# Formation, characterization and modeling of emergent synthetic microbial communities^☆^

**DOI:** 10.1016/j.csbj.2021.03.034

**Published:** 2021-04-09

**Authors:** Jia Wang, Dana L. Carper, Leah H. Burdick, Him K. Shrestha, Manasa R. Appidi, Paul E. Abraham, Collin M. Timm, Robert L. Hettich, Dale A. Pelletier, Mitchel J. Doktycz

**Affiliations:** a Biosciences Division, Oak Ridge National Laboratory, Oak Ridge, TN, USA; b Graduate School of Genome Science and Technology, University of Tennessee, Knoxville, TN, USA

**Keywords:** Microbial community, Rhizosphere bacteria, Genome-scale model, Flux balance analysis, Metabolic interaction, Metaproteomics

## Abstract

Microbial communities colonize plant tissues and contribute to host function. How these communities form and how individual members contribute to shaping the microbial community are not well understood. Synthetic microbial communities, where defined individual isolates are combined, can serve as valuable model systems for uncovering the organizational principles of communities. Using genome-defined organisms, systematic analysis by computationally-based network reconstruction can lead to mechanistic insights and the metabolic interactions between species. In this study, 10 bacterial strains isolated from the *Populus deltoides* rhizosphere were combined and passaged in two different media environments to form stable microbial communities. The membership and relative abundances of the strains stabilized after around 5 growth cycles and resulted in just a few dominant strains that depended on the medium. To unravel the underlying metabolic interactions, flux balance analysis was used to model microbial growth and identify potential metabolic exchanges involved in shaping the microbial communities. These analyses were complemented by growth curves of the individual isolates, pairwise interaction screens, and metaproteomics of the community. A fast growth rate is identified as one factor that can provide an advantage for maintaining presence in the community. Final community selection can also depend on selective antagonistic relationships and metabolic exchanges. Revealing the mechanisms of interaction among plant-associated microorganisms provides insights into strategies for engineering microbial communities that can potentially increase plant growth and disease resistance. Further, deciphering the membership and metabolic potentials of a bacterial community will enable the design of synthetic communities with desired biological functions.

## Graphical abstract

**Figure:**
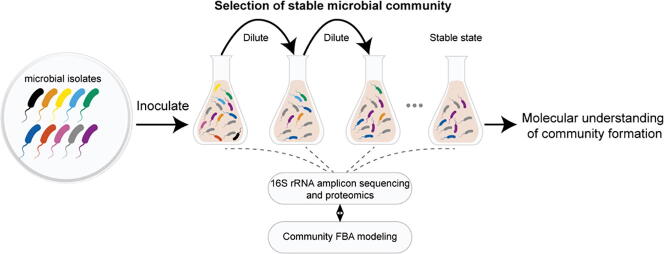

## 1 Introduction

Bacterial communities exert significant influence over a wide range of biological processes, such as human disease, plant interactions, biogeochemical cycles, and food fermentation [1], [2], [3]. An important challenge exists in understanding the underlying mechanisms that contribute to how bacteria shape their community and how the resulting structure depends on distinct environmental niches [2], [4], [5]. The interactions between species are dynamic and community membership depends on possessing the metabolic capabilities needed to survive in a particular environment [6]. Determining these trophic exchanges and interdependent metabolic processes is difficult in natural microbial communities comprised of hundreds of members. For example, phylogenetic marker and metagenomics analyses have revealed the extreme diversity of rhizosphere bacterial communities and the complex interplay among them [7], [8], [9], [10], [11]. It has been established that the composition and activity of root bacterial communities is spatially and temporally dynamic and can be influenced by both abiotic (e.g., soil nutrients, O_2_, pH, etc.) and biotic (e.g., host and microbe-microbe) factors [12], [13], [14], [15]. This complexity prevents tracking of metabolic fluxes from specific donor to acceptor strains or identifying competitive and cooperative relationships that leads to community structure [16], [17].

Simplified synthetic microbial communities are being considered as comprehensible systems for uncovering an in-depth view of community assembly principles. These systems are able to circumvent the complexity of natural ecosystems and allow the capture of community behaviors [17], [18], [19], [20], [21]. One approach is to reduce the complexity of natural communities by selection of microbial consortia under laboratory conditions from environmental samples [22]. This top-down approach can provide an overall co-occurrence correlation network but does not assess metabolic interactions in detail as individual genome and metabolic profiles are lacking [23]. A second approach is to construct synthetic bacterial communities from the bottom-up [24]. In the bottom-up approach, individual bacterial isolates are combined to give rise to a more complex microbial system where the original strains serve as sub-systems in an emergent community [25], [26], [27]. These easily manipulated bottom-up assemblies contribute to a promising approach for understanding interactions in natural communities [26], [28], [29]. Definition and characterization of each individual strain facilitates the study of potential synergistic effects in the synthetic community [25], [30]. The metabolites driving interspecies interactions can be determined and modeling of the metabolite exchange is possible [16], [19], [29]. This bottom-up approach can be used to experimentally select and investigate stable microbial community assembly [22].

The interplay among bacterial members in a consortium can be reconstructed using community-wide genome-scale metabolic models [31]. Recent applications of computational biology and genome-scale modeling approaches to the analysis of bottom-up assembled communities is providing mechanistic insights into the dynamic interactions occurring in defined bacterial communities [17], [23], [25]. For example, modeling studies have been applied for understanding biodegradation and bioproductivity [32]. In these models, it is often assumed that species interact in a pairwise manner [33], [34]. Two-species metabolic models assess cross-feeding networks and usually capture the positive interactions between the microorganisms [34], [35], [36]. Currently, metabolic exchanges among greater numbers of microbes are being modeled and found important for shaping community distribution [22], [37]. Modeling these higher-order interactions will be helpful for addressing questions regarding how and why a stable bacterial community forms.

**Fig. 1:**
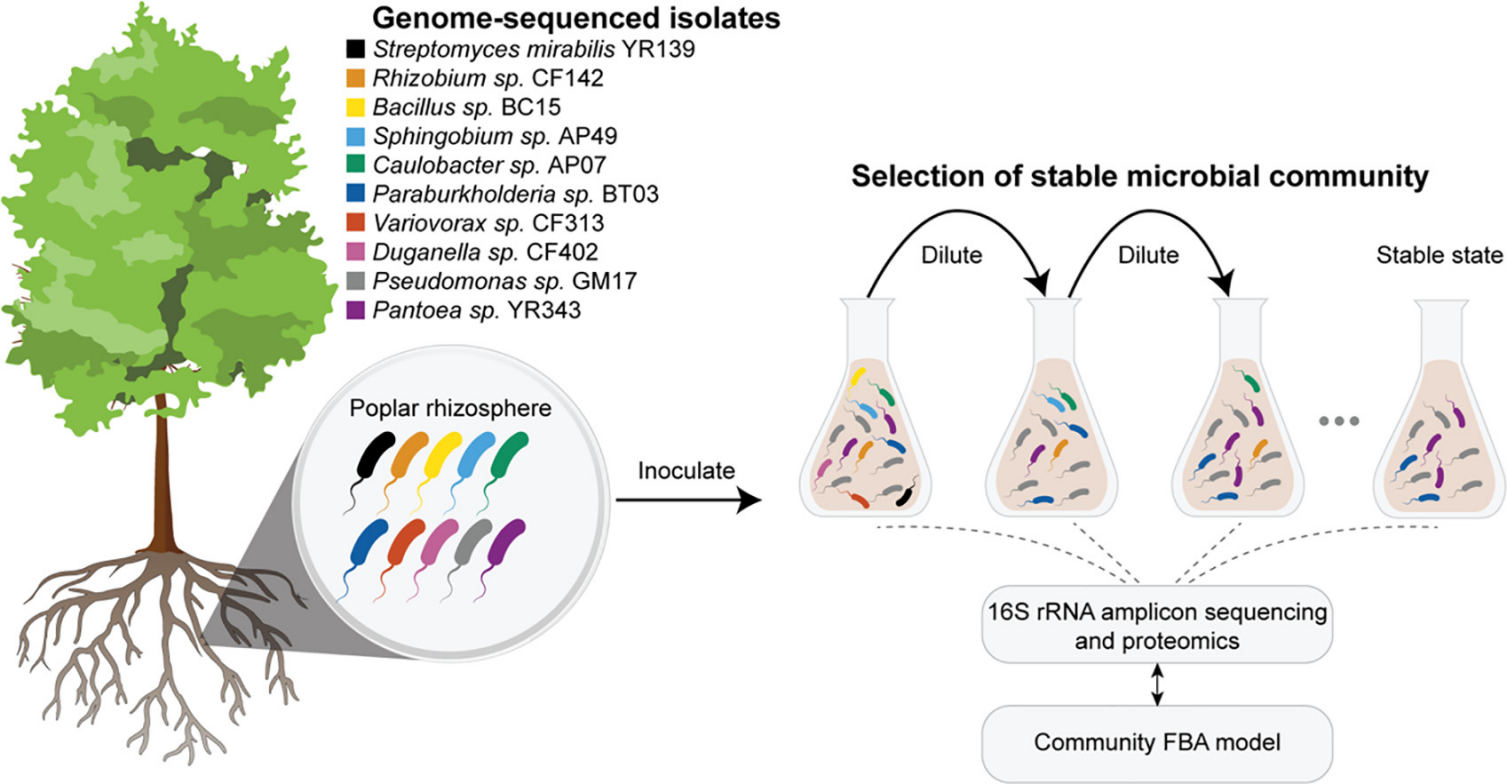
Overview of experimental design for the bottom-up assembly of stable communities utilizing defined bacterial isolates.

In this study, we describe the formation, characterization, and modeling of synthetic bacterial communities assembled from a highly characterized, phylogenetically diverse set of selected isolates in different media environments. The aim of these efforts is to define an approach to discovering simple, reproducible microbial communities, without predefined relationships, for detailed experimental studies that allow molecular and cellular level investigations into community structure. Using this discovery-based approach, we hypothesize that different community structures will result and depend on the media environment and the cooperative and competitive characteristics of the emergent community members. Ten bacterial strains (Table 1), isolated from *Populus deltoides* rhizosphere and with defined genome sequences, were co-cultured in either complex or minimal glucose media and serially transferred until a stable community structure formed. The resulting, reproducible system allows for understanding community assembly processes and investigation of causative molecular and cellular level events. To this end, a combination of marker gene profiling and metaproteomics characterization was carried out for tracking community structure and for gaining mechanistic insights into interactions between isolates (Fig. 1). These data are complemented by growth curve analyses and pairwise interaction screens. Different stable communities assemble in these environments and the higher-order interactions among community members are investigated. To unravel potential metabolic interactions among the surviving community members, genome-scale, community-level metabolic models were constructed for predicting potential metabolic processes involved in shaping the bacterial communities. The approach of discovering new microbial community structures under laboratory-defined conditions will facilitate understanding of the formation and dynamics of natural communities and the rational design of synthetic consortia with desired biological functions.

**Table 1:** General features of the bacterial isolates utilized for community experiments.

Strain	Phylogeny	Genome size (bp)	Number of coding sequence (CDS)	G + C content (%)	Reference
*Pantoea* sp. YR343	*γ-Proteobacteria*	5,391,843	4985	54.5	[7]
*Pseudomonas* sp. GM17	*γ-Proteobacteria*	6,866,808	6199	62.8	[7]
*Sphingobium* sp. AP49	*α-Proteobacteria*	4,506,188	4280	64.1	[7]
*Rhizobium* sp. CF142	*α-Proteobacteria*	6,068,985	5714	66.8	[7]
*Variovorax* sp. CF313	β*-Proteobacteria*	7,510,066	7608	60.1	[7]
*Bacillus* sp. BC15	*Firmicutes*	6,240,445	5413	62.9	[40]
*Caulobacter* sp. AP07	*α-Proteobacteria*	5,615,958	4915	68.9	[7]
*Duganella* sp. CF402	β*-Proteobacteria*	11,048,459	9632	61.9	[39]
*Streptomyces mirabilis* YR139	*Actinobacteria*	5,742,731	5635	34.8	[39]
*Paraburkholderia* sp. BT03	β*-Proteobacteria*	11,452,267	11,227	70.3	[38]

## 2 Materials and methods

### 2.1 Community construction

All 10 wild type bacterial strains (Table 1) were previously isolated from the rhizosphere of *Populus deltoides* [7], [38], [39], [40]. Two media were utilized in the experiments. A complex medium and a minimal, chemically defined medium were used in order to ascertain the influence of the chemical environment on microbial community selection. R2A was selected as the complex medium as this medium was used in the original environmental isolations of the strains. The medium contains essential amino acids and both simple and complex carbon sources that can potentially be utilized for growth (e.g., pyruvate, starch, dextrose). In contrast, the second medium, MOPS-glucose, is a defined mineral salts medium with a single carbon source that also supports growth of each of the individual strains. The MOPS minimal medium was prepared as described in [41] and 0.2% glucose was added as the carbon source. The R2A complex medium was prepared as described in [42]. The bacterial community was cultured through a serial dilution protocol. Each strain was inoculated from an agar plate and grown individually in a test tube with 10 mL R2A or MOPS liquid medium and cultivated in a shaking incubator at 30 °C and 200 rpm for 48 h. The growth of each strain was evaluated by measuring optical density at 600 nm (OD_600_) and then the cultures were normalized to the culture which had the lowest OD_600_ (0.228 in MOPS and 0.987 in R2A). Equal volumes of the normalized culture were mixed together, and the community was transferred into fresh medium using a 1:10 dilution. The starting inoculum for each medium contained a mixture of the ten bacterial strains at approximately equal concentrations as determined by OD_600_. Triplicate cultures, for both media, were incubated for 48 h at 30 °C with shaking at 200 rpm and passaged every 48 h at a 1:10 dilution for 15 passages. All bacterial strains in community are either obligate aerobes or facultative anaerobes, and shaking conditions ensured aeration required for growth. At the end of each passage, the bacterial cells were collected by centrifugation at 12,000 rpm for 15 min and stored at −80 °C.

### 2.2 DNA extraction and microbial relative abundance analysis

To determine the composition of the bacterial communities at each passage, genomic DNA was extracted from three replicate cell pellets collected at the end of each passage using the Qiagen DNeasy Blood and Tissue Kit (Qiagen, Valencia, CA) according to the manufacturer’s instructions. DNA concentrations were determined on an Invitrogen Qubit fluorometer (Thermo Fisher Scientific, Waltham, MA). The 16S rRNA gene was amplified using 515 forward and 806 reverse primers, barcoded, and prepared for sequencing using previously described methods [43]. The amplified products were then sequenced at Oak Ridge National Laboratory with a single 2 × 300 paired-end sequencing kit on Illumina MiSeq (Illumina Inc., San Diego, CA). Raw sequences were trimmed using cutadapt (v.1.18) to remove primers. The sequences were imported into QIIME2 (v. 2019.1) for further processing. Sequence variants were assigned using DADA2 implemented in QIIME2 plugin. Taxonomy was assigned using the consensus vsearch option in QIIME2 against a database of 16S sequences from the 10 community members [44], [45], [46], [47]. The resulting sequence variant table, mapping file, and taxonomy file were imported into Phyloseq (version 1.22.3) in R (version 3.4.4) for visualization. We corrected for 16S rRNA copy number using a custom R script (available at https://github.com/dlcarper/CopyNumberCorrection) and the number of copies obtained from the isolate genomes. Raw sequences were deposited in the NCBI SRA database under bioproject number PRJNA658537.

### 2.3 Maximum growth rate measurements

Individual bacterial strains were inoculated from R2A agar plates and grown in 10 mL of R2A or MOPS liquid media in test tubes for 48 h as seed cultures. These seed cultures were measured for OD_600_ and then normalized to the culture which had the lowest OD_600_ value. The seed culture of each strain was inoculated (5%, v/v) to the same liquid medium (10 mL) in a test tube and cultured at 30 °C with shaking at 200 rpm for 48 h. Optical density measurements at 600 nm were taken at 2-h intervals during the exponential phase of the growth curves. Bacterial cells were collected by centrifugation at 12,000 rpm and heated at 60 °C to measure the cell dry weight (CDW). A calibration curve for each strain was created by correlating the OD_600_ to the CDW and applied to transform the OD values to cell concentration. The maximum growth rates of bacterial strains were calculated as the slope of the plot between ln(X/X_0_) and time during the exponential growth phase. The X and X_0_ are the cell concentration (mg/mL) at time t and the time at the beginning of the exponential phase [48]. The length of lag phase was determined from a logarithmic plot of the growth curve and identified as the time point that results from extrapolating the slope of the exponential growth phase to the intersection with the initial inoculum OD value [49].

### 2.4 Pairwise interaction screens

Pairwise interactions were performed as described previously [11], briefly 5 μL of overnight R2A liquid culture of a selected microbe was spotted on R2A agar plate containing a lawn of another selected microbe and incubated at 25 °C for 48 h. Results were recorded as antagonistic when a zone of inhibition was observed, commensal when there was no obvious phenotype observed, or mutualistic when enhanced growth was observed around the test strain.

### 2.5 Individual and community modeling and flux balance analysis

The Department of Energy Systems Biology Knowledgebase (KBase) platform (www.kbase.us) was applied for the automated reconstruction of individual metabolic models and flux balance analysis (FBA) for the 10 bacterial isolates [50]. The FBA was carried out following previously described methods [51]. In brief, the genome of each strain was annotated by Annotate Microbial Genome app, which is based on Rapid Annotations using Subsystems Technology (RAST) toolkit to annotate prokaryotic genomes [52], [53], [54]. The metabolic modeling (Build Metabolic Model app) was performed using the annotated genome of each strain, and then the model was gapfilled (Gapfill Metabolic Model app) on R2A or MOPS media growth conditions to ensure they are qualified to simulate growth. The statistics on the constructed metabolic models for each strain are shown in Tables S1 and S2. Finally, FBA of the gapfilled model was predicted using biomass production as the objective value.

For community modeling, the individual models of the major surviving community members determined by 16S amplicon sequencing were merged into community metabolic model using the Merge Metabolic Models into Community Model app. The relative ratio of the community members was adjusted by the 16S rRNA experimentally determined ratio of the final passage of co-culture of 10 bacterial isolates (Table S3) using the method in [51]. Then, the adjusted community models were gapfilled and analyzed by FBA using the same procedure as individual models to estimate the fluxes of biochemical reactions and biomass production in the bacterial community. For constraining microbial growth in the community models, a calibration curve between the carbon uptake limit and growth rate was built using the FBA model of each individual strain, and the carbon uptake limit was calculated based on the experimental growth rate of each strain using the linear regression equation of the corresponding calibration curve. The carbon uptake limit of the community model was the summation of individual growth rate × relative percentage. The models used in this study are accessible online (https://narrative.kbase.us/narrative/73218 and https://narrative.kbase.us/narrative/73221).

### 2.6 Community proteomics analysis

#### 2.6.1 Cellular protein extraction

Cell pellets, collected at the end of the passage, were solubilized in 600 µL of lysis buffer (4% sodium dodecyl sulfate (SDS) in 100 mM Tris, pH 8.0) supplemented with 1 × Halt Protease Inhibitor Cocktail (Thermo Fisher Scientific, Waltham, MA). Samples were vortexed and then further disrupted with a Bullet Blender storm 24 (Next Advance) bead beater for 5 min using 0.15 mm Zirconium oxide beads at 3:1 sample to bead ratio. Samples were then placed in a heat-block for 10 min at 90 °C. Protein concentration was measured using a Nanodrop One spectrophotometer (Thermo Fisher Scientific, Waltham, MA). Protein disulfide bonds were reduced with 10 mM dithiothreitol (DTT) at 90 °C for 10 min and then alkylated with 30 mM iodoacetamide (IAA) for 15 min in the dark to prevent the reformation of disulfide bonds. As previously described [55], proteins were extracted by protein aggregation capture on Ser-Mag beads at 1:1 protein to beads ratio [55] and digested with sequencing grade Pierce trypsin (Thermo Fisher Scientific, Waltham, MA) at 1:75 (wt/wt) protein:trypsin ratio for overnight followed by a second 3 h digestion at 37 °C at constant shaking. Samples were vortexed and centrifuged at 12,000g for 15 min in 10 kDa molecular weight spin column filters (Vivaspin 500). Tryptic peptide flow-throughs were collected and then desalted using Pierce peptide desalting spin columns (Thermo Fisher Scientific, Waltham, MA) as per the manufacturer's instructions. Desalted peptides were vacuum dried with a SpeedVac Concentrator (Thermo Fisher Scientific, Waltham, MA) and then resolubilized in 0.1% formic acid. Peptide concentrations were measured using a nanodrop (Thermo Fisher Scientific, Waltham, MA) and transferred to the auto-sampler vials for LC-MS/MS measurement.

#### 2.6.2 Protein identification and quantification

Each sample was analyzed using two-dimensional (2D) liquid chromatography (LC) on an Ultimate 3000 RSLCnano system (Thermo Fisher Scientific, Waltham, MA) coupled with a Q Exactive Plus mass spectrometer (Thermo Fisher Scientific, Waltham, MA). For each sample, an aliquot of digested peptide mixture was injected across an in-house built strong cation exchange (SCX) Luna trap column (5 µm, 150 µm × 50 mm; Phenomenex, Torrance, CA) followed by a nanoEase symmetry reversed-phase (RP) C18 trap column (5 µm, 300 µm × 50 mm; Waters, Milford, MA) and washed with an aqueous solvent. Cellular peptide mixtures were separated and analyzed across three successive SCX fractions of increasing concentrations of ammonium acetate (35 mM, 50 mM, and 500 mM), each followed by a 100-minute organic gradient (25 nL/min flow rate) to separate peptides across an in-house pulled nanospray emitter analytical column (75 µm × 350 mm) packed with C18 Kinetex RP C18 resin (1.7 µm; Phenomenex, Torrance, CA). All MS data were acquired with Thermo Xcalibur (version 4.2.47) using the topN method where N could be up to 10. Target values for the full scan MS spectra were 3 × 10^6^ charges in the 300–1500 *m*/*z* range with a maximum injection time of 25 ms. Transient times corresponding to a resolution of 70,000 at *m*/*z* 200 were chosen. A 1.6 *m*/*z* isolation window and fragmentation of precursor ions was performed by higher-energy C-trap dissociation (HCD) with a normalized collision energy of 27 eV. MS/MS scans were performed at a resolution of 17,500 at *m*/*z* 200 with an ion target value of 1 × 10^5^ and a maximum injection time of 50 ms. Dynamic exclusion was set to 20 s to avoid repeated sequencing of peptides.

All MS raw data files were analyzed using the Proteome Discoverer software (version 2.3, Thermo Fischer Scientific, Waltham, MA). Raw files were processed by the SEQUEST HT database search algorithm [56] and confidence in peptide-to-spectrum (PSM) matching was evaluated by Percolator [57]. Peptide and PSMs were considered identified at q < 0.01 and proteins were required to have at least one unique peptide sequence. Protein relative abundance values were calculated by summing together peptide extracted ion chromatograms. Protein abundances were normalized by LOESS and mean central tendency procedures performed on log2-transformed values by InfernoRDN [58]. From this normalized dataset, protein abundances subset for each microbe were extracted and further mean-centered by InfernoRDN. All proteomics spectral data in this study were deposited at the ProteomeXchange Consortium via the MASSIVE repository (https://massive.ucsd.edu/). The data can be reviewed under the username “MSV000086551_reviewer” and password “PMI”.

Organism relative abundance (i.e., population size) was assessed using two approaches [59]: 1) total protein count per organism divided by the total count of proteins per community and 2) summed total relative protein abundance per organism divided by the summed total protein abundance per community. Note, it is important to evaluate and compare both approaches to account for biases in protein expression. In this study, we found these two approaches provided similar results and report relative organism abundances using the second approach.

## 3 Results and discussion

### 3.1 Stable community structure in minimal and complex media

**Fig. 2:**
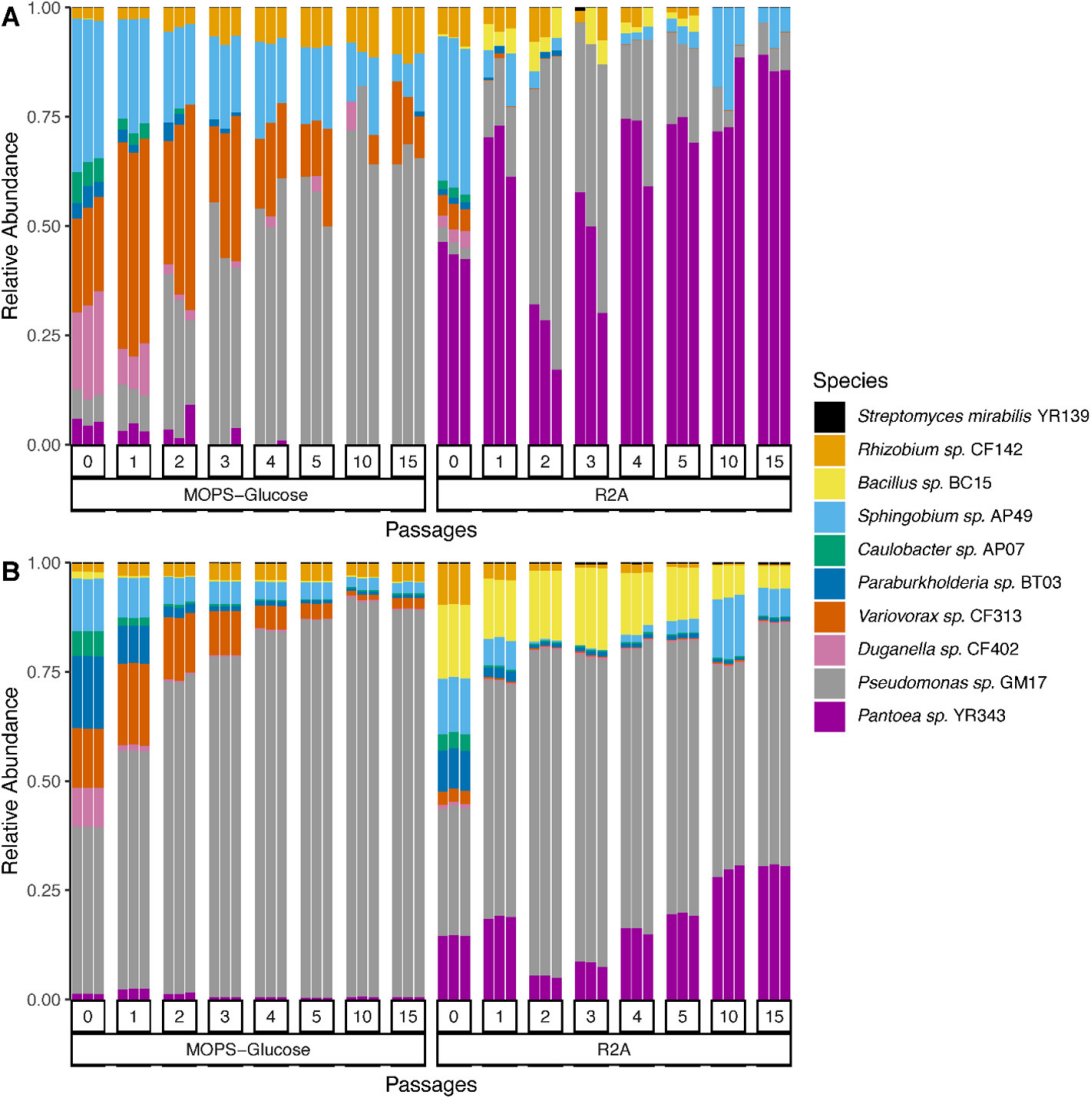
Analysis of the relative abundances of the 10 bacterial strains after sequential passages in MOPS minimal and R2A complex media. The relative abundances of each bacterial strain in the community are based on A) 16S rRNA gene amplicon sequencing results and B) metaproteomic results. Passage 0 represents the end of the first growth cycle after the initial inoculation. Each bar is a replicate, with three replicates per passage. The numbers shown on the bottom represent the passage number for those samples.

Stable microbial communities were formed by serial transfer of batch cultures containing a mixture of ten, phylogenetically diverse bacterial strains derived from the *Populus* rhizosphere (Table 1). These strains represent phyla that are abundant in the rhizosphere of plants [43], [60]. The use of batch cultures allows for effective exchange of metabolites and the preparation of samples for analytical measurements. The component strains’ genomes are sequenced and comprise three α-Proteobacteria, three β-Proteobacteria, two γ-Proteobacteria, one Firmicute and one Actinobacterium. These batch cultures were subsequently transferred to fresh medium every 48 h and the relative proportion of each member was analyzed by 16S rRNA gene amplicon Illumina sequencing and quantitative metaproteomics. They are in general agreement regarding the trends in membership of the stable microbial communities that are formed in the two different media environments (Fig. 2). In both environments, the bacterial diversity decreases from the initial inoculation, and both measurement approaches show changes in the relative proportions of the bacterial strains that settle into a similar distribution beginning with approximately the fifth dilution cycle (see Supplemental Fig. S1). This observation is consistent with the expectation that competition for local resources will reduce the genotypic diversity within a bacterial community [61]. In the MOPS minimal medium, four strains consistently dominate in abundance and stably coexist starting with passage No. 4. Organismal abundance trends in R2A show a fluctuation in organism relative abundances occurring between passages two and five that substantially alters the abundance of several members of the community until a community stably coexists starting with passage No. 10.

In general, 16S rRNA gene amplicon sequencing and metaproteomics results provide similar estimates of the relative microbial abundance distribution for each measured passage in the minimal MOPS medium but differ in the rich R2A medium. In MOPS medium, *Pseudomonas* sp. GM17 is the most abundant member along with three other strains, *Variovorax* sp. CF313, *Rhizobium* sp. CF142 and *Sphingobium* sp. AP49, that persist in consistent proportions (Fig. 2). Estimates of organism proportion in the R2A medium, on the other hand, are quite different between the two approaches. For 16S rRNA gene amplicon sequencing, three members dominate in abundance when grown in R2A complex medium, and *Pantoea* sp. YR343 is the member with highest content after 15 passages (Fig. 2A). The less abundant strains in R2A medium are *Pseudomonas* sp. GM17 and *Sphingobium* sp. AP49. Metaproteome analysis reveals the same dominant members, albeit the relative abundance of *Pantoea* sp. YR343 is strikingly different (Fig. 2B). Additionally, there is a notable abundance of *Bacillus* sp. BC15 not seen in 16S rRNA gene amplicon sequencing data. These differences can be attributed to measurement distinctions that result from using either a DNA- or protein-based approach to assess organism proportions in microbial communities. For example, examination of the proteins expressed by *Bacillus* sp. across the passages reveals an abundance of sporulation-related processes. Spores are notoriously challenging to measure by 16S rRNA gene amplicon sequencing because they are difficult to lyse, which negatively impacts DNA extraction efficiency [62]. Selective PCR amplification before amplicon sequencing may be another potential source of bias, since the designation of a perfectly matching universal primer is not possible [63], [64]. Nevertheless, there is clearly a benefit in using both approaches. For instance, the observed differences in the relative abundance of *Pantoea* sp. YR343 is likely because 16S rRNA gene amplicon sequencing measures DNA from viable cells as well as ‘relic’ DNA (i.e., DNA from dead cells), whereas metaproteomics is a more accurate estimate of biomass from viable, functioning cells [59], [65], [66]. Assimilating the results between these two measurements suggests that *Pantoea* sp. YR343 may experience a population dieback event prior to the timepoint of sampling.

### 3.2 Experimentally determined growth rates for the individual bacterial isolates

**Fig. 3:**
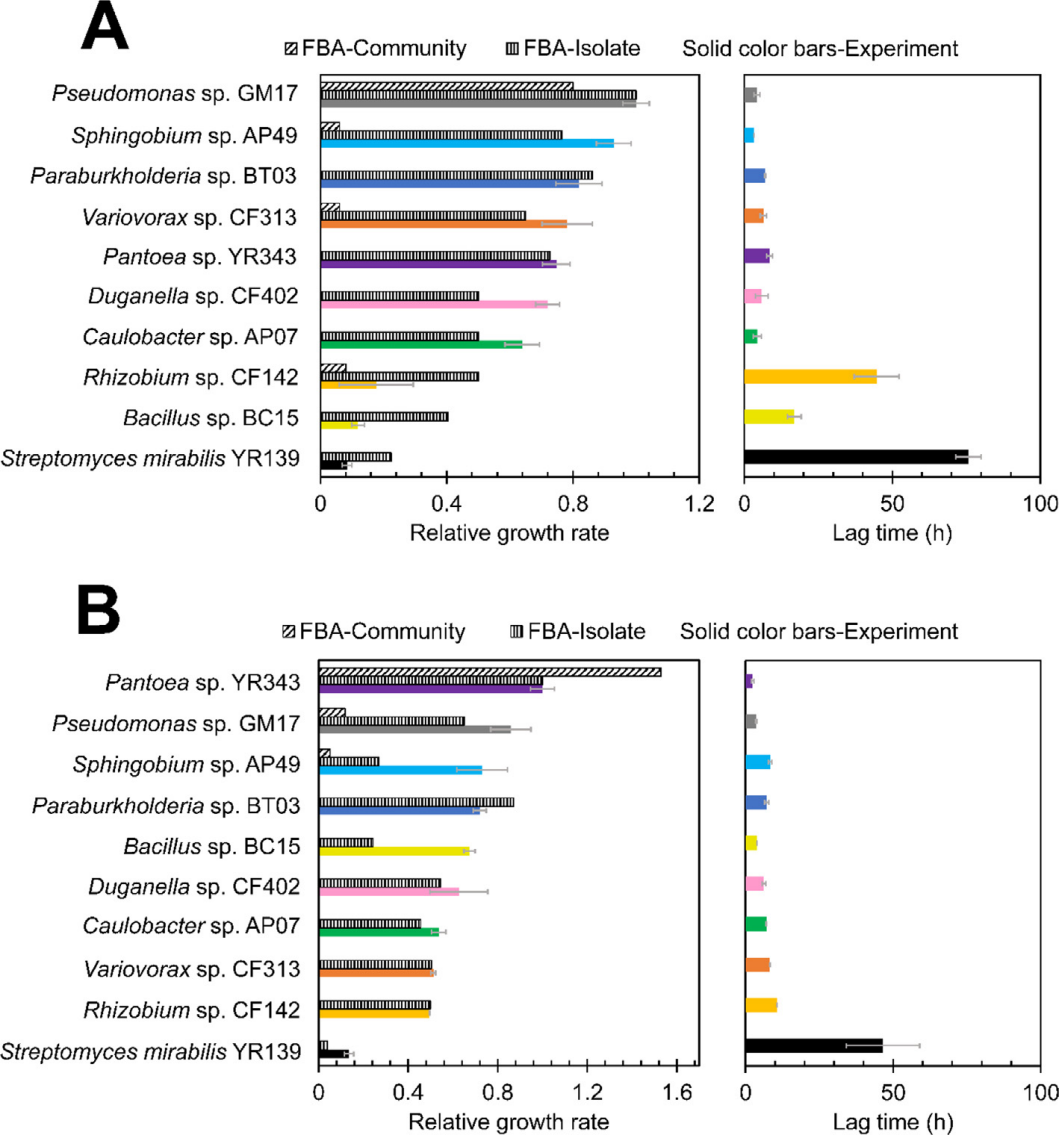
Comparison of experimentally determined growth parameters with FBA model predicted growth. A) Relative growth rates and lag times for the individual strains in MOPS medium; B) relative growth rates and lag times for the strains in R2A medium. Each data column of experimental results represents the mean and error bars are the standard deviation over three parallel experiments.

Individual microbial growth rates can impact interactions within a community [33], experimentally determined growth rates of the 10 individual strains were obtained for both media and show substantial differences. In the MOPS medium, strain *Pseudomonas* sp. GM17 has the highest growth rate (0.463 h^−1^) among the group (Fig. 3A). It is also the dominant strain in the community growth experiment (Fig. 2). In the same medium, *Sphingobium* sp. AP49 (0.430 h^−1^) has a maximum growth rate that is similar to that observed with the *Pseudomonas* sp. GM17 and also maintains a presence in the community. Compared to these two organisms, *Paraburkholderia* sp. BT03 (0.379 h^−1^), *Variovorax* sp. CF313 (0.362 h^−1^), *Pantoea* sp. YR343 (0.346 h^−1^), *Duganella* sp. CF402 (0.333 h^−1^) and *Caulobacter* sp. AP07 (0.296 h^−1^) have slightly lower maximum growth rates, but only *Variovorax* sp. CF313 persists in the community. Three organisms, *Rhizobium* sp. CF142 (0.082 h^−1^), *Bacillus* sp. BC15 (0.055 h^−1^) and *Streptomyces mirabilis* YR139 (0.039 h^−1^), have considerably lower growth rates when compared to the other organisms. Interestingly, despite having a relatively slow growth rate in the MOPS medium, *Rhizobium* sp. CF142 prevails as a dominant community member.

In R2A medium, there is a strong correlation between the growth rates of individual strains and community membership. Not surprisingly, the growth rates of all of the selected microbes are higher in this rich medium when compared to growth rates observed in MOPS medium. Overall, *Pantoea* sp. YR343 (0.656 h^−1^), *Pseudomonas* sp. GM17 (0.563 h^−1^) and *Sphingobium* sp. AP49 (0.479 h^−1^) are relatively fast growers in R2A and are significant components of the emergent community. In contrast, *Paraburkholderia* sp. BT03 (0.472 h^−1^), *Bacillus* sp. BC15 (0.442 h^−1^), and *Duganella* sp. CF402 (0.411 h^−1^), despite showing growth rates only slightly lower than *Sphingobium* sp. AP49, do not maintain a significant presence in successive community growth cycles (Fig. 3B).

Comparison of the individual member growth rates to the observed community composition indicates complex relationships among the community members. On the one hand, the presence of fast-growing microbes in the emerging community composition is expected and consistent with observations in other systems. The competitive exclusion principle predicts that when the bacterial members in a community compete for the same resources, the fitter strain will outcompete the other members [6] and has a greater opportunity for recurrent colonization that can allow for persistence in the community [67]. Yet, several fast-growing microbes are absent in the final passages of the community and slow growers are present. Often, slower growing microbes persist in communities and allow for maintenance of diversity. Here, the well-mixed conditions promote exchange of metabolites during bacterial growth and prevents spatial structuring that often allows for maintenance of slower growing organisms [22], [68]. Therefore, bacterial strains must collectively adjust their behavior and selectively cooperate in order to emerge into a community with stable proportions. The resulting supportive associations among community members likely proceeds through metabolic interactions such as the cross-feeding of essential nutrients [61]. One particularly interesting observation to this point is the persistence of *Rhizobium* sp. CF142 in the MOPS minimal medium community. When grown in monoculture in MOPS minimal medium, this strain has a slower growth rate and lower final OD when compared to the other strains. It could be speculated that metabolic cooperation emerges in these mixed microbial communities increasing the fitness of strain CF142, potentially by providing some missing nutrients made available by other community members [69], [70]. This is consistent with the existence of both cooperative and competitive associations between the component members that lead to the formation of a community network structure [71].

### 3.3 FBA-based growth prediction models for individual microbes

To gain a better understanding of individual strain metabolism and the potential variety of exchanged metabolites, FBA models of each of the component microbes were generated. By estimating the reaction fluxes to generate biomass constituents, the growth rate of the microorganism can be predicted [72]. The maximum relative growth rates of the ten bacterial isolates in R2A and MOPS media are displayed in Fig. 3. In these models, the objective value of growth is determined by setting a maximum glucose uptake flux of 100 mmol/g DCW/h. Among the 10 *Populus* bacterial isolates in MOPS medium, *Pseudomonas* sp. GM17 has the highest predicted and actual growth rate and was used for normalization. Similarly, among the 10 *Populus* bacterial isolates grown in R2A medium, *Pantoea* sp. YR343 has the maximal predicted and actual growth rate and was used as the reference standard.

In MOPS medium, the hierarchy of relative predicted growth rates generally matches the experimentally observed growth rates. In this medium, the maximal growth rate predictions for the slowest growing organisms, *Rhizobium* sp. CF142, *Bacillus* sp. BC15, and *Streptomyces mirabilis* YR139 are significantly overestimated and likely reflect imperfect understanding of metabolism in these organisms. When compared to the others, these organisms all have long lag phases (Fig. 3A) which likely reflects unknown adaptations to environmental conditions [73]. Further, these organisms may adopt different growth strategies that do not prioritize the conversion of glucose to biomass. In the case of *Streptomyces mirabilis* YR139, the unusual growth and morphological characteristics of this genus can contribute to experimental and predictive errors.

In R2A media, growth rate predictions show a different trend. In general, the predicted relative growth rates of the slower growing organisms match the experimentally observed maximal growth rates. In contrast, growth rate predictions are poor for several of the faster growing microbes (Fig. 3B). In particular, growth rate predictions are significantly underestimated for *Sphingobium* sp. AP49 and *Bacillus* sp. BC15 in this complex medium. Again, this may reflect unknown limits on metabolism for these species. The FBA predicted growth rates assume ideal conditions; all nutrients in the medium are made available at the maximum uptake flux. In the growth experiments of those individual strains, the growth rate could not be ideally as high as the FBA models.

In the FBA model using R2A medium, the dominant strain *Pantoea* sp. YR343 has the highest number of exchange reactions of nutrients (Table S4), and it has more transporters based on genome annotation compared with the other community members. For *Pantoea* sp. YR343, the higher number of transporter genes may be related to its stronger metabolic interaction potential encoded in the genome. In the nutrient rich complex medium, *Pantoea* sp. YR343 is capable of utilizing a variety of nutrients which is consistent with the observation that this organism has the highest simulated growth rate. In contrast, the number of exchange reactions identified in individual FBA models carried out in MOPS medium is similar (Table S5). This likely results from the minimal medium environment where nutrition is relatively limited compared with the complex R2A medium. In MOPS medium models, the medium-specific metabolic processes may override the strain-specific metabolisms, leading to much less difference in simulated growth rates among the 10 isolates when compared to models employing R2A medium [74]. The discrepancy of simulated growth rates of the same strain between MOPS and R2A media also corresponds to the experimental observation that bacteria utilize their metabolism for survival in the minimal medium, in contrast they tend to have more active growth in the complex medium [75].

### 3.4 Evaluation of microbial community models

#### 3.4.1 FBA-based growth prediction for community models

FBA-based community models were assembled in order to identify microbial features that account for the observed stability of the community and for assessing the suitability of these models and their use in understanding the molecular genetic bases for the resulting community structure. Flux changes of the metabolites involved in exchange may explain the interaction mechanisms between the component members. Compartmentalized models were created using the KBase platform to allow for the community members to secrete and take up metabolites from a shared environment [17], [51]. To create these models, the genome-scale metabolic models of the primary constituents of the final community were combined using the relative ratio of the community members determined from the 16S rRNA gene marker data for the final passage. These final, persistent strains are considered the best-performing species and as community drivers that affect dependent species and community organization [76], [77].

The FBA-based community models predict altered growth rates for the constituent members when compared to their individual growth rates. In MOPS medium, the dominant strain *Pseudomonas* sp. GM17 is predicted to have the fastest growth among the consortium members. However, this growth rate is lower when compared with its individual FBA model (Fig. 3A). This is most likely due to competition with other community members in this limited nutrient environment and thus a lower growth rate is not surprising. In comparison, the three other major strains in this consortium have significantly lower, but similar, predicted growth rates when compared to *Pseudomonas* sp. GM17 as well as to their individual models (Fig. 3A). Altered growth rates can result from competitive interactions and have been observed in other studies using minimal media for microbial community assembly [78], [79], [80]. These prior studies indicate that the metabolic capability associated with each strain can influence community composition and lead to the survival of the strongest competitors [22], [81].

When using R2A medium in the community metabolic model, the dominant strain *Pantoea* sp. YR343 has a much higher theoretical growth rate than that of its individual model. This suggests that it is beneficial for *Pantoea* sp. YR343 to grow in the presence of the other two members in the community. The higher predicted growth rate for *Pantoea* sp. YR343 in the community model may relate to the organism’s broad spectrum of transport reactions [82]. In a community, the bacterial members that are metabolic generalists have a better chance of survival compared to those that are adapted to specific substrates [22], [83]. For both media, the community-FBA models support the dominant presence of a fast-growing member and suggest that members of the community influence each other’s growth rate.

#### 3.4.2 FBA-based predictions of metabolic exchanges

Limiting the uptake flux of carbon is the foundation of a constraint-based FBA model and allows prediction of the distribution of metabolic fluxes that depend on the medium [84]. Without a carbon source uptake limitation, metabolite exchanges between community members will be overestimated. Therefore, a limitation of carbon uptake flux was added to the FBA models for the communities modeled in either MOPS or R2A medium. Calibration curves relating carbon source uptake flux and growth rate objective value were established for each community member, and the individual carbon uptake flux limitation of each strain was calculated based on the experimental growth rate. The carbon uptake limit for a community FBA model was calculated as the sum of the relative ratios of the individual microbial components multiplied by their individual carbon uptake rates.

**Fig. 4:**
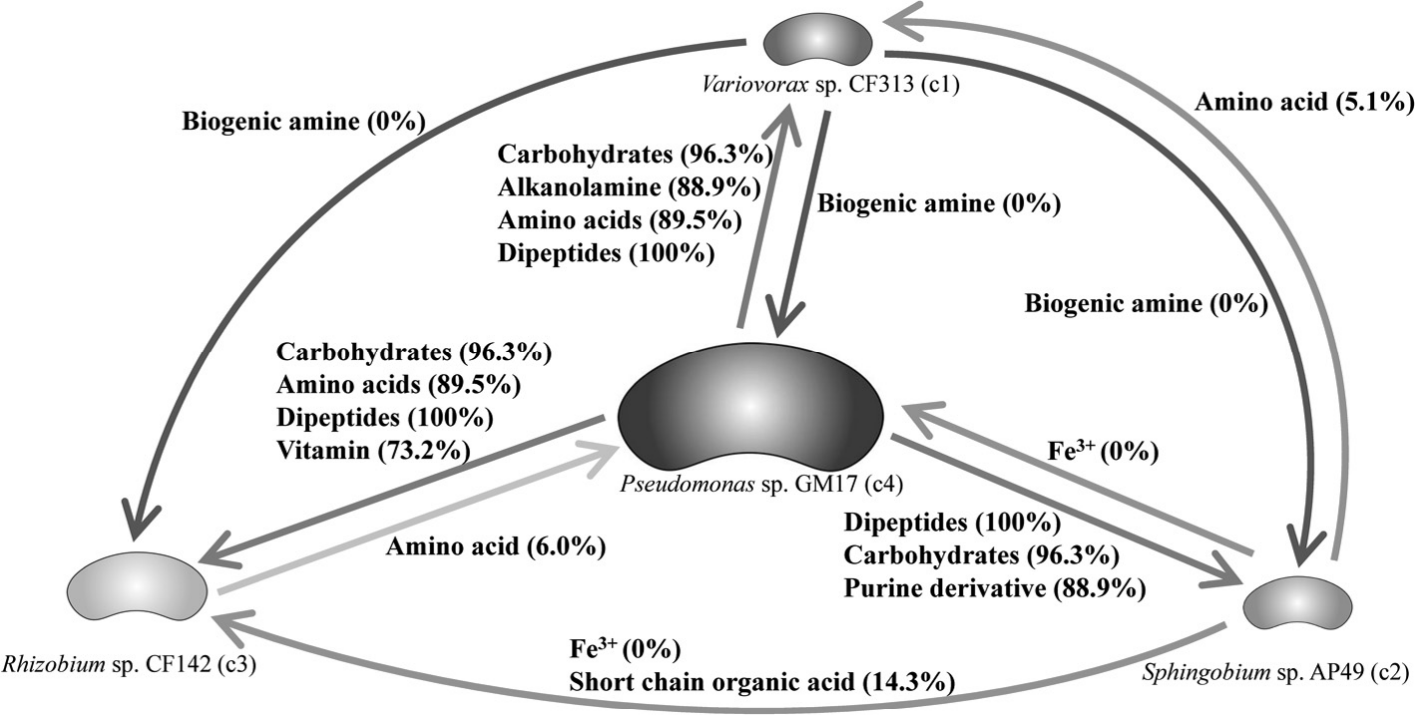
Predicted metabolite exchange among the four dominant members of the microbial community formed in MOPS medium as proposed by the community FBA model. The percentage of detected enzymes by metaproteomics analyses is shown in parentheses.

The extracellular metabolites involved in interspecies exchanges can be predicted by the community FBA models [51]. Fig. 4 illustrates the predicted metabolite exchanges among the four component members in MOPS medium. The dominant strain, *Pseudomonas* sp. GM17 is predicted to supply more metabolites to the other community members than it receives. Key among these predicted metabolites are amino acids, sugars and purine derivatives. In the minimal medium environment, cellular building materials must be wholly prepared from the glucose carbon source, or, in a community environment, scavenged from the excretions of other microbes. Accessing excreted metabolites may be vital for the maintenance of those members with minor content in the community. In turn, *Pseudomonas* sp. GM17 may rely on the production of metabolites from these minor community members as evidenced by its lower growth rate in the community FBA model when compared with its individual FBA model. The metaproteomics results provide an opportunity to assess whether the appropriate pathways are represented. The percentage of the detected enzymes in the metabolite supplier is related to the total protein involved in the KEGG pathways for the exchanged metabolites. Considering the dominant microbial component *Pseudomonas* sp. GM17, there is high representation of the enzymes related to the metabolites supplied by this organism (Fig. 4). In contrast, enzymes involved in the preparation of metabolites shared from the other organisms are low. This lower representation is a consequence of the relatively lower number of proteins detected from these organisms relative to *Pseudomonas* sp. GM17.

**Fig. 5:**
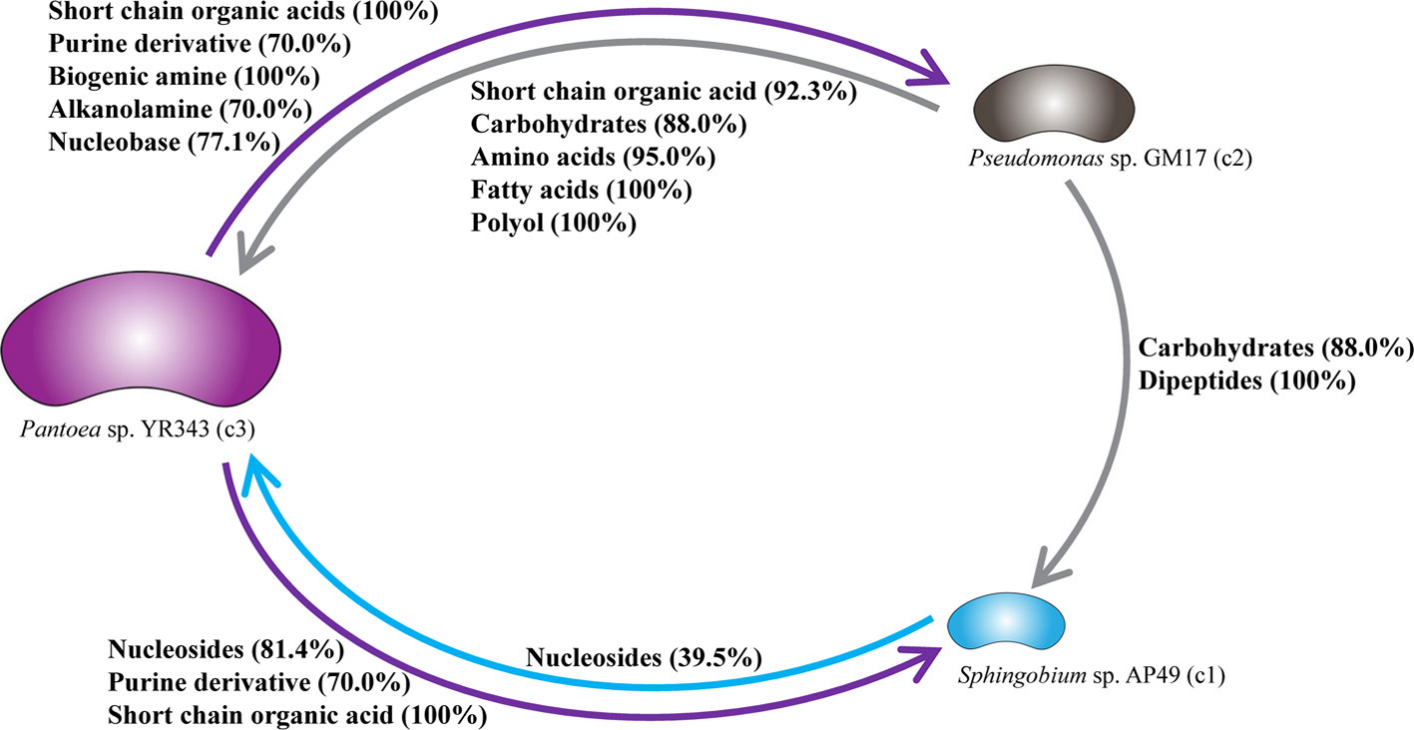
Predicted metabolites exchange among the three dominant members of the microbial community formed in R2A medium as proposed by the community FBA model. The percentage of detected enzymes by metaproteomics analyses is shown in parentheses.

In the community model using R2A medium, metabolite exchange can also be predicted. Here, the dominant strain *Pantoea* sp. YR343, based on the 16S rRNA marker data, is expected to export a greater range of metabolites than it receives from the other two members of the community (Fig. 5). Organic acids, purine derivatives and biogenic amines are predicted to be excreted and support the growth of the other community members. The community FBA model predicts a faster growth rate for *Pantoea* sp. YR343 in the community model when compared to the individual model and this may result from access to metabolites excreted by the other community members. Again, the community metaproteomics results can be used to assess the presence of relevant pathways. The high representation of both *Pantoea* sp. YR343 and *Pseudomonas* sp. GM17 in the metaproteomics data allows detection of most of the enzymes expected to participate in the FBA-based predictions. As in the case of the minimal medium environment, representation of the minor microbial components is poor, and confirmation of relevant components of metabolism is much lower when compared to the major microbial components.

The combination of omics data with FBA modeling can aid with understanding community function and stability. Here, the metaproteomics results help support predictions of the FBA model by providing evidence for the metabolic capabilities of each community member and making them more faithful representations of the biological system being interrogated [23]. While the coverage of enzymes implicated in the exchanged metabolites is relatively high for prominent members of the community, as communities grow in membership diversity, metaproteomic sequencing depth will need to improve for the more minor members, which may disclose their survival mechanisms. Additionally, the integration of metabolomics data will be valuable for constraining FBA models and for confirming predictions of interdependencies between community members.

The inherent complexity of metabolic interactions is a challenge in the modeling of microbial communities [85]. The genome-scale, mechanistic modeling provided by the FBA approach is presently insufficient to account for a large fraction of intracellular networks or assess dynamic, population level changes that likely lead to community structuring [72]. Integration with population-level dynamic models, such Lotka-Volterra (LV) [86], [87], [88], or r/K selection strategies [89] may help to describe the temporal progress of species abundances and community formation processes. Dynamic FBA, which simulates the dynamics of community growth and substrate consumption in time-dependent processes, can also extend current FBA approaches to temporal changes [90], [91], [92]. Effective application of these tools will require new, time dependent global sampling and measurement strategies for verifying the efficacy of dynamic models.

### 3.5 Pairwise interaction screens

While the present metabolic models help to understand interactions that support growth of a stable community, they provide only partial insight into the selection process that leads to community formation. In both tested environments, a fast-growing microbe emerges as a dominant component that is supportive of other community members. However, other relatively fast-growing microbes are out competed in early growth cycles, and their abundance fades from the composition of the community. Antagonistic interactions may be present that facilitate the community selection process. To assess this possibility, community members were screened for mutualistic, commensal, or antagonistic colony phenotypes in a pairwise interaction screen. While a majority of the interactions appear to be commensal, with no obvious phenotypes, the screen did identify both positive and negative interactions (Table 2). *Pseudomonas* sp. GM17 cells were antagonistic to the growth of the majority of the other community members, while *Pantoea* sp. YR343 demonstrated positive interactions with several strains indicating an obvious role for competition, antimicrobial production and or beneficial interactions in microbial community selection, structure and stability.

**Table 2:** Pairwise interaction screen results. Strain designations across top of table indicate lawn of microbes spread on R2A agar plate and designations on left indicate cells spotted on center of lawn. + indicates a positive interaction while - indicates an antagonistic interaction. Empty cells indicate no obvious colony phenotype change.

Genus	Strain	YR343	GM17	AP49	CF142	CF313	BC15	AP07	CF402	YR139*	BT03
*Pantoea*	YR343				+	+				ND	
*Pseudomonas*	GM17			–	–	–	–	–	–	ND	–
*Sphingobium*	AP49					–	+			ND	
*Rhizobium*	CF142						+			ND	
*Variovorax*	CF313				–					ND	–
*Bacillus*	BC15								–	ND	
*Caulobacter*	AP07			–						ND	
*Duganella*	CF402					+	+			ND	
*Streptomyces*	YR139			–	–			–		ND	
*Paraburkholderia*	BT03				–					ND	

* Due to the growth characteristics of *Streptomyces* sp. YR139, a lawn of bacteria could not be prepared and resulted in no data (ND).

Interestingly, although the growth of strain AP49, CF313 and CF142 were inhibited by strain GM17 in pairwise interaction screens, these strains still co-existed with strain GM17 during the 10-member community cultivation. This may be attributed to intertwined metabolic interactions among these four strains, in which the beneficial effect from the metabolites in a shared extracellular environment for growth overwhelms antagonistic effects by strain GM17. Alternatively, different experimental conditions may account for the unexpected result. The multi-member community was grown in a well-mixed liquid medium condition which is different from the static agar plate conditions of the pairwise interaction screen and may account for the observed discrepancy [93]. The integration of temporal modeling of microbial communities with time course of community composition within the growth period in a passage can be promising to accommodate these antagonistic interactions and the dynamic processes that shape community structure.

## 4 Conclusions

Distinct bacterial communities can be formed from a more complex mixture of microbial isolates. Diverse bacterial species, without previously known or expected obligate relationships, were combined and put through dilution cycles. After approximately five cycles, select members prevail and form fairly stable community structures that persist through successive cycles. Further, the resulting community structures depend on the media environment used for the dilution cycles. The described approach to discovering stable, media-dependent emergent communities takes advantage of genome-defined isolates to allow for effective implementation of systems biology tools. Growth curve analyses, metaproteomics and FBA analyses were employed to identify key factors that contribute to the resulting community structure. Growth rate was identified as providing an advantage for a microbial member to maintain presence in the community. Interestingly, some of the fastest growing organisms remain in the final community structure but other rapid growing organisms do not. Further, under minimal medium conditions, a relatively slow growing organism was found to persist. Pairwise interaction measurements highlight that selective antagonistic relationships may contribute to the final structuring of the community. In order to gain a molecular-level understanding of the resulting microbial organization, FBA analyses were performed. Metabolic exchanges between organisms can be identified and likely underpin the shaping of community membership. Metaproteomic results support general findings of the FBA models. However, presently accessible FBA tools primarily account for central metabolite fluxes with cell growth as the final objective. Understanding dynamic processes at the molecular and cell population levels will be required to understand community formation, dynamics, and structure. Improved modeling capabilities, coupled with time dependent measurements and the described, scalable approach to identifying stable communities will facilitate definition of the molecular events that result in microbial community structure and dynamics.

## Declaration of Competing Interest

The authors declare that they have no known competing financial interests or personal relationships that could have appeared to influence the work reported in this paper.
