# Supplementary material for: Formation, characterization and modeling of emergent synthetic microbial communities
Source: Comput Struct Biotechnol J. 2021 Apr 9;19:1917–27. doi: 10.1016/j.csbj.2021.03.034 (PMC8079826; doi:10.1016/j.csbj.2021.03.034)
Supplement: Supplementary data 1 [file mmc1.pdf]

Supplemental Material

Formation, characterization and modeling of emergent synthetic microbial communities

Jia Wang[a], Dana L. Carper[a], Leah H. Burdick[a], Him K. Shrestha[a,b], Manasa R. Appidi[a,b], Paul E. Abraham[a], Collin M. Timm[a,c], Robert L. Hettich[a], Dale A. Pelletier[a,*], Mitchel J. Doktycz[a,*]

[a]Biosciences Division, Oak Ridge National Laboratory, Oak Ridge, TN, USA

[b]Graduate School of Genome Science and Technology, University of Tennessee, Knoxville, TN, USA

[c]Current address: Research and Exploratory Development Department, Johns Hopkins University Applied Physics Laboratory, Laurel, MD, USA

* Corresponding authors

E-mail addresses: pelletierda@ornl.gov (D. A. Pelletier) and doktyczmj@ornl.gov (M. J. Doktycz)

Figures S1-S2 and Supplemental Tables S1-S6

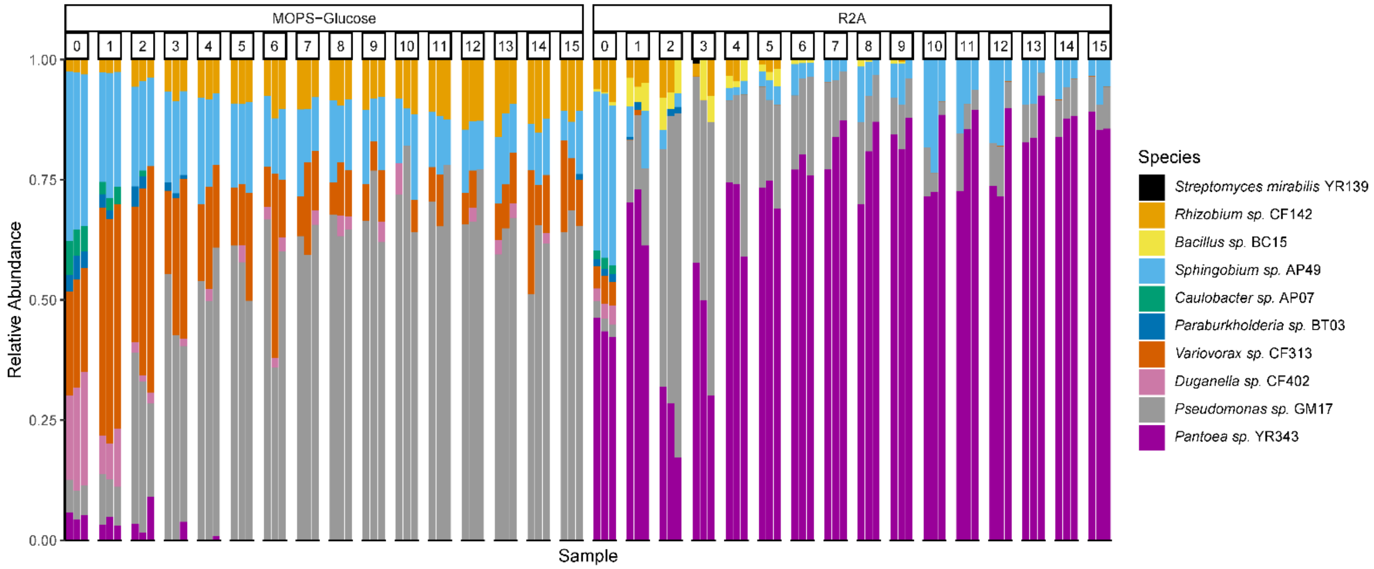

Figure S1. The relative abundances of each bacterial strain in the community in every passage based on the 16S rRNA gene amplicon sequencing results

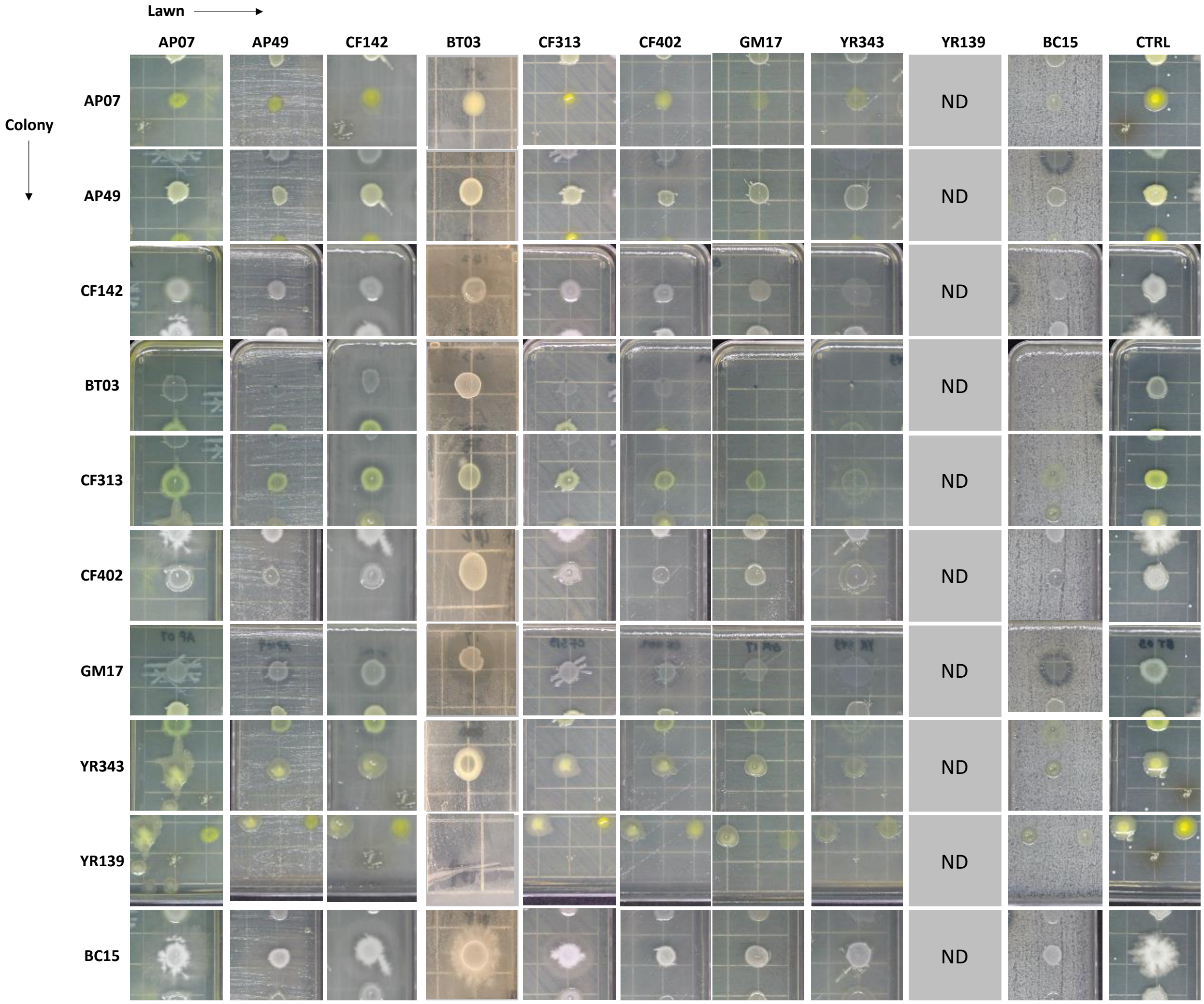

Figure S2. Pairwise interaction screen results. The bacterial strain designations listed along the top indicate the bacterial lawn. Due to the growth characteristics of *Streptomyces* sp. YR139, a lawn of bacteria could not be prepared and resulted in no data (ND). The bacterial strain designations listed along the left indicate the spotted bacterial strain

Table S1. General features of individual metabolic models using R2A medium

Strain	Number of reactions	Number of compounds	Number of genes	Number of added reactions for gapfill	Number of reversed reactions for gapfill	Number of proteins identified
Pantoea sp. YR343	1341	1262	1231	51	4	1727
Pseudomonas sp. GM17	1309	1260	1259	65	1	3139

Sphingobium sp. AP49	1086	1097	850	82	1	363
Variovorax sp. CF313	1233	1197	1292	59	1	11
Rhizobium sp. CF142	1285	1245	1551	63	1	21
Duganella sp. CF402	1235	1189	1030	59	2	21
Caulobacter sp. AP07	1106	1114	902	79	5	16
Burkholderia sp. BT03	1432	1303	1924	52	2	41
Bacillus sp. BC15	1274	1215	1094	51	4	291
Streptomyces mirabilis YR139	1251	1201	1657	74	2	25

Table S2. General features of individual metabolic models using MOPS medium

Strain	Number of reactions	Number of compounds	Number of genes	Number of added reactions for gapfill	Number of reversed reactions for gapfill	Number of proteins identified
Pantoea sp. YR343	1346	1264	1231	56	4	17
Pseudomonas sp. GM17	1313	1260	1259	69	1	2614
Sphingobium sp. AP49	1088	1096	850	84	0	74
Variovorax sp. CF313	1236	1198	1292	62	1	76
Rhizobium sp. CF142	1292	1247	1551	70	0	123
Duganella sp. CF402	1244	1189	1030	68	0	10
Caulobacter sp. AP07	1111	1111	902	84	2	14
Burkholderia sp. BT03	1435	1305	1924	55	3	20
Bacillus sp. BC15	1278	1215	1094	55	3	10
Streptomyces mirabilis YR139	1256	1203	1657	79	2	9

Table S3. General features of community metabolic models

Community model	Medium	Relative proportion in community model	Number of reactions	Number of compounds	Number of genes	Number of added reactions for gapfill	Number of reversed reactions for gapfill
YR343-GM17-AP49	R2A	90%-7%-3%	3728	3408	3340	190	11
GM17-CF142-AP49-CF313	MOPS	80%-8%-6%-6%	4909	4517	4952	265	11

Table S4. Exchange reactions of individual FBA models in R2A medium

Strain	Exchange reactions	Active import transport reaction	Active export transport reaction
Pantoea sp. YR343	158	35	13
Pseudomonas sp. GM17	131	30	11
Sphingobium sp. AP49	95	21	8
Variovorax sp. CF313	112	24	8
Rhizobium sp. CF142	113	23	8
Duganella sp. CF402	120	28	8
Caulobacter sp. AP07	101	29	11
Burkholderia sp. BT03	135	27	9
Bacillus sp. BC15	124	26	12
Streptomyces mirabilis YR139	112	24	7

Table S5. Exchange reactions of individual FBA models in MOPS medium

Strain	Exchange reactions	Active import transport reaction	Active export transport reaction
Pantoea sp. YR343	159	13	8
Pseudomonas sp. GM17	130	13	6
Sphingobium sp. AP49	93	13	7
Variovorax sp. CF313	112	13	5
Rhizobium sp. CF142	114	13	6
Duganella sp. CF402	118	13	5
Caulobacter sp. AP07	97	13	6
Burkholderia sp. BT03	136	13	6
Bacillus sp. BC15	123	13	7
Streptomyces mirabilis YR139	113	14	6
