# Supplementary material for: Formation, characterization and modeling of emergent synthetic microbial communities
Source: Comput Struct Biotechnol J. 2021 Apr 9;19:1917–27. doi: 10.1016/j.csbj.2021.03.034 (PMC8079826; doi:10.1016/j.csbj.2021.03.034)
Supplement: Supplementary data 2 [file mmc2.pdf]

Table S6. Enzymes involved in the KEGG pathways identified by metaproteomics analysis at passage No. 15

Reaction ID	KEGG Pathways	Enzyme name	Definition of reaction	Flux	Proteomics Identification
***Pseudomonas* sp. GM17, community in MOPS medium**					
rxn00555	Alanine, aspartate and glutamate metabolism|Amino sugar and nucleotide sugar metabolism	L-glutamine:D-fructose-6-phosphate isomerase (deaminating)	(1) L-Glutamine_c0[c4] + (1) D-fructose-6-phosphate_c0[c4] <-> (1) L-Glutamate_c0[c4] + (1) D-Glucosamine phosphate_c0[c4]	0.0365231	Yes
rxn00802	Alanine, aspartate and glutamate metabolism|Arginine and proline metabolism	2-(Nomega-L-arginino)succinate arginine-lyase (fumarate-forming)	(1) L-Argininosuccinate_c0[c4] <-> (1) L-Arginine_c0[c4] + (1) Fumarate_c0[c4]	0.089547	Yes
rxn00503	Alanine, aspartate and glutamate metabolism|Arginine and proline metabolism	(S)-1-pyrroline-5-carboxylate:NAD+ oxidoreductase	(2) H2O_c0[c4] + (1) NAD_c0[c4] + (1) 1-Pyrroline-5-carboxylate_c0[c4] <-> (1) NADH_c0[c4] + (1) L-Glutamate_c0[c4] + (1) H+_c0[c4]	-0.062608	Yes
rxn01434	Alanine, aspartate and glutamate metabolism|Arginine and proline metabolism	L-Citrulline:L-aspartate ligase (AMP-forming)	(1) ATP_c0[c4] + (1) L-Aspartate_c0[c4] + (1) Citrulline_c0[c4] <-> (1) PPi_c0[c4] + (1) AMP_c0[c4] + (2) H+_c0[c4] + (1) L-Argininosuccinate_c0[c4]	0.089547	Yes
rxn00182	Alanine, aspartate and glutamate metabolism|Arginine and proline metabolism|Taurine and hypotaurine metabolism|D-Glutamine and D-glutamate metabolism|Nitrogen metabolism	L-glutamate:NAD+ oxidoreductase (deaminating)	(1) H2O_c0[c4] + (1) NAD_c0[c4] + (1) L-Glutamate_c0[c4] <-> (1) NADH_c0[c4] + (1) NH3_c0[c4] + (1) 2-Oxoglutarate_c0[c4] + (1) H+_c0[c4]	-2.42879	Yes
rxn00260	Alanine, aspartate and glutamate metabolism|Carbon fixation in photosynthetic organisms	L-Aspartate:2-oxoglutarate aminotransferase	(1) 2-Oxoglutarate_c0[c4] + (1) L-Aspartate_c0[c4] <-> (1) L-Glutamate_c0[c4] + (1) Oxaloacetate_c0[c4]	-0.932139	Yes
rxn00416	Alanine, aspartate and glutamate metabolism|Nitrogen metabolism	L-aspartate:L-glutamine amido-ligase (AMP-forming)	(1) H2O_c0[c4] + (1) ATP_c0[c4] + (1) L-Aspartate_c0[c4] + (1) L-Glutamine_c0[c4] -> (1) PPi_c0[c4] + (1) AMP_c0[c4] + (1) L-Glutamate_c0[c4] + (2) H+_c0[c4] + (1) L-Asparagine_c0[c4]	0.0852542	Yes
rxn00347	Alanine, aspartate and glutamate metabolism|Nitrogen metabolism	L-Aspartate ammonia-lyase	(1) L-Aspartate_c0[c4] <-> (1) NH3_c0[c4] + (1) Fumarate_c0[c4]	0.0455833	Yes
rxn03638	Amino sugar and nucleotide sugar metabolism	Acetyl-CoA:D-glucosamine-1-phosphate N-acetyltransferase	(1) Acetyl-CoA_c0[c4] + (1) D-Glucosamine1-phosphate_c0[c4] -> (1) CoA_c0[c4] + (1) H+_c0[c4] + (1) N-Acetyl-D-glucosamine1-phosphate_c0[c4]	0.033975	Yes
rxn01485	Amino sugar and nucleotide sugar metabolism	D-Glucosamine 1-phosphate 1,6-phosphomutase	(1) D-Glucosamine1-phosphate_c0[c4] <-> (1) D-Glucosamine phosphate_c0[c4]	-0.033975	Yes
rxn00293	Amino sugar and nucleotide sugar metabolism	UTP:N-acetyl-alpha-D-glucosamine-1-phosphate uridylyltransferase	(1) UTP_c0[c4] + (1) N-Acetyl-D-glucosamine1-phosphate_c0[c4] <-> (1) PPi_c0[c4] + (1) UDP-N-acetylglucosamine_c0[c4]	-0.0533017	Yes
rxn01484	Amino sugar and nucleotide sugar metabolism	N-Acetyl-D-glucosamine-6-phosphate amidohydrolase	(1) H2O_c0[c4] + (1) N-Acetyl-D-glucosamine 6-phosphate_c0[c4] <->	-0.00254813	No

			(1) Acetate_c0[c4] + (1) D-Glucosamine phosphate_c0[c4]		
rxn02285	Amino sugar and nucleotide sugar metabolism|Peptidoglycan biosynthesis	UDP-N-acetylmuramate:NADP+ oxidoreductase	(1) NADP_c0[c4] + (1) UDP-MurNAc_c0[c4] <-> (1) NADPH_c0[c4] + (1) H+_c0[c4] + (1) UDP-N-acetylglucosamine enolpyruvate_c0[c4]	-0.00849375	Yes
rxn00461	Amino sugar and nucleotide sugar metabolism|Peptidoglycan biosynthesis	Phosphoenolpyruvate:UDP-N-acetyl-D-glucosamine 1-carboxyvinyl-transferase	(1) UDP-N-acetylglucosamine_c0[c4] + (1) Phosphoenolpyruvate_c0[c4] <-> (1) Phosphate_c0[c4] + (1) UDP-N-acetylglucosamine enolpyruvate_c0[c4]	0.00849375	Yes
rxn01636	Arginine and proline metabolism	N2-Acetyl-L-ornithine:L-glutamate N-acetyltransferase	(1) L-Glutamate_c0[c4] + (1) N-Acetylornithine_c0[c4] <-> (1) Ornithine_c0[c4] + (1) N-Acetyl-L-glutamate_c0[c4]	0.0837798	Yes
rxn01917	Arginine and proline metabolism	ATP:N-acetyl-L-glutamate 5-phosphotransferase	(1) ATP_c0[c4] + (1) N-Acetyl-L-glutamate_c0[c4] <-> (1) ADP_c0[c4] + (1) n-acetylglutamyl-phosphate_c0[c4]	0.0837798	Yes
rxn00929	Arginine and proline metabolism	L-Proline:NAD+ 5-oxidoreductase	(1) NAD_c0[c4] + (1) L-Proline_c0[c4] <-> (1) NADH_c0[c4] + (2) H+_c0[c4] + (1) 1-Pyrroline-5-carboxylate_c0[c4]	-0.244224	Yes
rxn02465	Arginine and proline metabolism	N-acetyl-L-glutamate-5-semialdehyde:NADP+ 5-oxidoreductase (phosphrylating)	(1) NADP_c0[c4] + (1) Phosphate_c0[c4] + (1) 2-Acetamido-5-oxopentanoate_c0[c4] <- (1) NADPH_c0[c4] + (1) H+_c0[c4] + (1) n-acetylglutamyl-phosphate_c0[c4]	-0.0837798	Yes
rxn01019	Arginine and proline metabolism	Carbamoyl-phosphate:L-ornithine carbamoyltransferase	(1) Ornithine_c0[c4] + (1) Carbamoylphosphate_c0[c4] -> (1) Phosphate_c0[c4] + (1) H+_c0[c4] + (1) Citrulline_c0[c4]	0.089547	Yes
rxn01637	Arginine and proline metabolism	N2-Acetyl-L-ornithine:2-oxoglutarate aminotransferase	(1) 2-Oxoglutarate_c0[c4] + (1) N-Acetylornithine_c0[c4] <-> (1) L-Glutamate_c0[c4] + (1) 2-Acetamido-5-oxopentanoate_c0[c4]	-0.0837798	No
rxn01791	beta-Alanine metabolism|Pantothenate and CoA biosynthesis	(R)-Pantoate:beta-alanine ligase (AMP-forming)	(1) ATP_c0[c4] + (1) beta-Alanine_c0[c4] + (1) Pantoate_c0[c4] -> (1) PPi_c0[c4] + (1) AMP_c0[c4] + (2) H+_c0[c4] + (1) PAN_c0[c4]	0.00210316	Yes
rxn00346	beta-Alanine metabolism|Pantothenate and CoA biosynthesis	L-aspartate 1-carboxy-lyase (beta-alanine-forming)	(1) L-Aspartate_c0[c4] + (1) H+_c0[c4] -> (1) CO2_c0[c4] + (1) beta-Alanine_c0[c4]	0.00210316	No
rxn02185	Butanoate metabolism	2-Acetolactate pyruvate-lyase (carboxylating)	(1) TPP_c0[c4] + (1) ALCTT_c0[c4] <-> (1) Pyruvate_c0[c4] + (1) 2-Hydroxyethyl-ThPP_c0[c4]	-0.121724	Yes
rxn00785	Carbon fixation in photosynthetic organisms	D-Fructose 6-phosphate:D-glyceraldehyde-3-phosphate glycolaldehyde transferase	(1) D-fructose-6-phosphate_c0[c4] + (1) Glyceraldehyde3-phosphate_c0[c4] <-> (1) D-Xylulose5-phosphate_c0[c4] + (1) D-Erythrose4-phosphate_c0[c4]	0.456266	Yes
rxn00285	Citrate cycle (TCA cycle)|Propanoate metabolism|Carbon fixation pathways in prokaryotes	Succinate:CoA ligase (ADP-forming)	(1) ATP_c0[c4] + (1) CoA_c0[c4] + (1) Succinate_c0[c4] <-> (1) ADP_c0[c4] + (1) Phosphate_c0[c4] + (1) Succinyl-CoA_c0[c4]	0.145207	Yes
rxn00126	Cysteine and methionine metabolism	ATP:L-methionine S-adenosyltransferase	(1) H2O_c0[c4] + (1) ATP_c0[c4] + (1) L-Methionine_c0[c4] -> (1) Phosphate_c0[c4] + (1) PPi_c0[c4] + (1) S-Adenosyl-L-methionine_c0[c4] + (1) H+_c0[c4]	0.00736105	Yes
rxn00952	Cysteine and methionine metabolism	O-acetyl-L-homoserine:hydrogen sulfide S-(3-amino-3-carboxypropyl)transferase	(1) H2S_c0[c4] + (1) O-Acetyl-L-homoserine_c0[c4] -> (1) Acetate_c0[c4] + (1) Homocysteine_c0[c4]	0.074625	Yes

rxn00141	Cysteine and methionine metabolism	S-Adenosyl-L-homocysteine hydrolase	(1) H2O_c0[c4] + (1) S-Adenosyl-homocysteine_c0[c4] <-> (1) Homocysteine_c0[c4] + (1) Adenosine_c0[c4]	0.00630948	Yes
rxn00693	Cysteine and methionine metabolism|One carbon pool by folate	5-Methyltetrahydrofolate:L-homocysteine S-methyltransferase	(1) Homocysteine_c0[c4] + (1) 5-Methyltetrahydrofolate_c0[c4] <-> (1) L-Methionine_c0[c4] + (1) Tetrahydrofolate_c0[c4]	0.0507632	Yes
rxn01303	Cysteine and methionine metabolism|Sulfur metabolism	Acetyl-CoA:L-homoserine O-acetyltransferase	(1) Acetyl-CoA_c0[c4] + (1) L-Homoserine_c0[c4] -> (1) CoA_c0[c4] + (1) O-Acetyl-L-homoserine_c0[c4]	0.074625	Yes
rxn00283	D-Alanine metabolism	alanine racemase	(1) L-Alanine_c0[c4] <-> (1) D-Alanine_c0[c4]	0.0169875	Yes
rxn00193	D-Glutamine and D-glutamate metabolism	glutamate racemase	(1) L-Glutamate_c0[c4] <-> (1) D-Glutamate_c0[c4]	0.00849375	Yes
rxn02286	D-Glutamine and D-glutamate metabolism|Peptidoglycan biosynthesis	UDP-N-acetylmuramate:L-alanine ligase (ADP-forming)	(1) ATP_c0[c4] + (1) L-Alanine_c0[c4] + (1) UDP-MurNAc_c0[c4] -> (1) ADP_c0[c4] + (1) Phosphate_c0[c4] + (1) H+_c0[c4] + (1) UDP-N-acetylmuramoyl-L-alanine_c0[c4]	0.00849375	Yes
rxn02008	D-Glutamine and D-glutamate metabolism|Peptidoglycan biosynthesis	UDP-N-acetylmuramoyl-L-alanine:D-glutamate ligase(ADP-forming)	(1) ATP_c0[c4] + (1) D-Glutamate_c0[c4] + (1) UDP-N-acetylmuramoyl-L-alanine_c0[c4] -> (1) ADP_c0[c4] + (1) Phosphate_c0[c4] + (1) H+_c0[c4] + (1) UDP-N-acetylmuramoyl-L-alanyl-D-glutamate_c0[c4]	0.00849375	Yes
rxn05345	Fatty acid biosynthesis	dodecanoyl-[acyl-carrier-protein]:malonyl-[acyl-carrier-protein] C-acyltransferase (decarboxylating)	(1) Dodecanoyl-ACP_c0[c4] + (1) Malonyl-acyl-carrierprotein-_c0[c4] -> (1) CO2_c0[c4] + (1) 3-oxotetradecanoyl-acp_c0[c4] + (1) ACP_c0[c4]	0.033975	Yes
rxn05465	Fatty acid biosynthesis	Malonyl-CoA:[acyl-carrier-protein] S-malonyltransferase	(1) H+_c0[c4] + (1) Malonyl-CoA_c0[c4] + (1) ACP_c0[c4] <-> (1) CoA_c0[c4] + (1) Malonyl-acyl-carrierprotein-_c0[c4]	0.338656	Yes
rxn05342	Fatty acid biosynthesis	(3R)-3-Hydroxytetradecanoyl-[acyl-carrier-protein]:NADP+ oxidoreductase	(1) NADP_c0[c4] + (1) HMA_c0[c4] <-> (1) NADPH_c0[c4] + (1) 3-oxotetradecanoyl-acp_c0[c4]	-0.033975	Yes
rxn00178	Fatty acid degradation|Synthesis and degradation of ketone bodies|Valine, leucine and isoleucine degradation|Lysine degradation|Benzoate degradation|Tryptophan metabolism|Pyruvate metabolism|Glyoxylate and dicarboxylate metabolism|Propanoate metabolism|Butanoate metabolism|Carbon fixation pathways in prokaryotes|Terpenoid backbone biosynthesis	Acetyl-CoA:acetyl-CoA C-acetyltransferase	(2) Acetyl-CoA_c0[c4] <-> (1) CoA_c0[c4] + (1) Acetoacetyl-CoA_c0[c4]	0.181616	Yes
rxn02504	Folate biosynthesis	2-amino-4-hydroxy-6-(D-erythro-1,2,3-trihydroxypropyl)-7,8-dihydropteridine glycolaldehyde-lyase	(1) Dihydroneopterin_c0[c4] <-> (1) Glycolaldehyde_c0[c4] + (1) 6-hydroxymethyl dihydropterin_c0[c4]	0.00315474	Yes

<table>
<tr><td>rxn02200</td><td>Folate biosynthesis</td><td>2-amino-4-hydroxy-6-hydroxymethyl-7,8-dihydropteridine:4-aminobenzoate 2-amino-4-hydroxydihydropteridine-6-methenyltransferase</td><td>(1) ABEE_c0[c4] + (1) 6-hydroxymethyl dihydropterin_c0[c4] <-> (1) H2O_c0[c4] + (1) Dihydropteroate_c0[c4]</td><td>0.00315474</td><td>Yes</td></tr>
<tr><td>rxn03841</td><td>Folate biosynthesis</td><td>4-amino-4-deoxychorismate pyruvate-lyase</td><td>(1) ADC_c0[c4] -> (1) Pyruvate_c0[c4] + (1) H+_c0[c4] + (1) ABEE_c0[c4]</td><td>0.00315474</td><td>Yes</td></tr>
<tr><td>rxn01257</td><td>Folate biosynthesis</td><td>chorismate:L-glutamine aminotransferase</td><td>(1) L-Glutamine_c0[c4] + (1) Chorismate_c0[c4] <-> (1) L-Glutamate_c0[c4] + (1) ADC_c0[c4]</td><td>0.00315474</td><td>Yes</td></tr>
<tr><td>rxn01603</td><td>Folate biosynthesis</td><td>7,8-dihydropteroate:L-glutamate ligase (ADP-forming)</td><td>(1) ATP_c0[c4] + (1) L-Glutamate_c0[c4] + (1) Dihydropteroate_c0[c4] -> (1) ADP_c0[c4] + (1) Phosphate_c0[c4] + (1) H+_c0[c4] + (1) Dihydrofolate_c0[c4]</td><td>0.00315474</td><td>Yes</td></tr>
<tr><td>rxn03167</td><td>Folate biosynthesis</td><td>2-Amino-4-hydroxy-6-(erythro-1,2,3-trihydroxypropyl) dihydropteridine triphosphate phosphohydrolase (alkaline optimum)</td><td>(3) H2O_c0[c4] + (1) 7,8-Dihydroneopterin 3'-triphosphate_c0[c4] -> (3) Phosphate_c0[c4] + (3) H+_c0[c4] + (1) Dihydroneopterin_c0[c4]</td><td>0.00315474</td><td>No</td></tr>
<tr><td>rxn00650</td><td>Glutathione metabolism</td><td>L-cysteinylglycine dipeptidase</td><td>(1) H2O_c0[c4] + (1) Cys-Gly_c0[c4] <-> (1) Glycine_c0[c4] + (1) L-Cysteine_c0[c4]</td><td>-0.00105158</td><td>Yes</td></tr>
<tr><td>rxn00350</td><td>Glutathione metabolism</td><td>glutathione gamma-glutamylaminopeptidase</td><td>(1) H2O_c0[c4] + (1) GSH_c0[c4] <-> (1) L-Glutamate_c0[c4] + (1) Cys-Gly_c0[c4]</td><td>-0.00105158</td><td>Yes</td></tr>
<tr><td>rxn00615</td><td>Glycerolipid metabolism</td><td>ATP:glycerol 3-phosphotransferase</td><td>(1) ATP_c0[c4] + (1) Glycerol_c0[c4] <-> (1) ADP_c0[c4] + (1) H+_c0[c4] + (1) Glycerol-3-phosphate_c0[c4]</td><td>0.0108484</td><td>Yes</td></tr>
<tr><td>rxn00611</td><td>Glycerophospholipid metabolism</td><td>sn-Glycerol-3-phosphate:NAD+ 2-oxidoreductase</td><td>(1) NAD_c0[c4] + (1) Glycerol-3-phosphate_c0[c4] <-> (1) NADH_c0[c4] + (1) H+_c0[c4] + (1) Glycerone-phosphate_c0[c4]</td><td>-0.0650905</td><td>Yes</td></tr>
<tr><td>rxn01300</td><td>Glycine, serine and threonine metabolism</td><td>ATP:L-homoserine O-phosphotransferase</td><td>(1) ATP_c0[c4] + (1) L-Homoserine_c0[c4] <-> (1) ADP_c0[c4] + (1) H+_c0[c4] + (1) O-Phospho-L-homoserine_c0[c4]</td><td>0.152556</td><td>Yes</td></tr>
<tr><td>rxn01069</td><td>Glycine, serine and threonine metabolism</td><td>O-phospho-L-homoserine phosphate-lyase (adding water;L-threonine-forming)</td><td>(1) H2O_c0[c4] + (1) O-Phospho-L-homoserine_c0[c4] -> (1) Phosphate_c0[c4] + (1) L-Threonine_c0[c4]</td><td>0.152556</td><td>Yes</td></tr>
<tr><td>rxn00692</td><td>Glycine, serine and threonine metabolism|Cyanoamino acid metabolism|Glyoxylate and dicarboxylate metabolism|One carbon pool by folate|Methane metabolism</td><td>5,10-Methylenetetrahydrofolate:glycine hydroxymethyltransferase</td><td>(1) H2O_c0[c4] + (1) Glycine_c0[c4] + (1) 5-10-Methylenetetrahydrofolate_c0[c4] <-> (1) L-Serine_c0[c4] + (1) Tetrahydrofolate_c0[c4]</td><td>-0.322689</td><td>Yes</td></tr>
<tr><td>rxn00742</td><td>Glycine, serine and threonine metabolism|Cysteine and methionine metabolism</td><td>L-cystathionine cysteine-lyase (deaminating; 2-oxobutanoate-forming)</td><td>(1) H2O_c0[c4] + (1) Cystathionine_c0[c4] -> (1) NH3_c0[c4] + (1) L-Cysteine_c0[c4] + (1) 2-Oxobutyrate_c0[c4]</td><td>0.0301713</td><td>Yes</td></tr>
<tr><td>rxn00337</td><td>Glycine, serine and threonine metabolism|Cysteine and methionine metabolism|Lysine biosynthesis</td><td>ATP:L-aspartate 4-phosphotransferase</td><td>(1) ATP_c0[c4] + (1) L-Aspartate_c0[c4] <-> (1) ADP_c0[c4] + (1) 4-Phospho-L-aspartate_c0[c4]</td><td>0.342305</td><td>Yes</td></tr>
</table>

rxn01301	Glycine, serine and threonine metabolism|Cysteine and methionine metabolism|Lysine biosynthesis	L-Homoserine:NAD+ oxidoreductase	(1) NAD_c0[c4] + (1) L-Homoserine_c0[c4] <-> (1) NADH_c0[c4] + (1) H+_c0[c4] + (1) L-Aspartate4-semialdehyde_c0[c4]	-0.227181	Yes
rxn01643	Glycine, serine and threonine metabolism|Cysteine and methionine metabolism|Lysine biosynthesis	L-Aspartate-4-semialdehyde:NADP+ oxidoreductase (phosphorylating)	(1) NADP_c0[c4] + (1) Phosphate_c0[c4] + (1) L-Aspartate4-semialdehyde_c0[c4] <- (1) NADPH_c0[c4] + (1) H+_c0[c4] + (1) 4-Phospho-L-aspartate_c0[c4]	-0.342305	Yes
rxn00420	Glycine, serine and threonine metabolism|Methane metabolism	O-phospho-L-serine phosphohydrolase	(1) H2O_c0[c4] + (1) phosphoserine_c0[c4] -> (1) Phosphate_c0[c4] + (1) L-Serine_c0[c4]	0.440683	Yes
rxn01101	Glycine, serine and threonine metabolism|Methane metabolism	3-Phospho-D-glycerate:NAD+ 2-oxidoreductase	(1) NAD_c0[c4] + (1) 3-Phosphoglycerate_c0[c4] <-> (1) NADH_c0[c4] + (1) H+_c0[c4] + (1) 3-Phosphonooxypyruvate_c0[c4]	0.440683	Yes
rxn02914	Glycine, serine and threonine metabolism|Methane metabolism	3-Phosphoserine:2-oxoglutarate aminotransferase	(1) 2-Oxoglutarate_c0[c4] + (1) phosphoserine_c0[c4] <-> (1) L-Glutamate_c0[c4] + (1) 3-Phosphonooxypyruvate_c0[c4]	-0.440683	Yes
rxn01964	Glycine, serine and threonine metabolism|Phenylalanine, tyrosine and tryptophan biosynthesis	L-serine hydro-lyase [adding 1-C-(indol-3-yl)glycerol 3-phosphate; L-tryptophan and glyceraldehyde-3-phosphate-forming]	(1) L-Serine_c0[c4] + (1) Indoleglycerol phosphate_c0[c4] -> (1) H2O_c0[c4] + (1) L-Tryptophan_c0[c4] + (1) Glyceraldehyde3-phosphate_c0[c4]	0.0160301	Yes
rxn00737	Glycine, serine and threonine metabolism|Valine, leucine and isoleucine biosynthesis	L-threonine ammonia-lyase (2-oxobutanoate-forming)	(1) L-Threonine_c0[c4] -> (1) NH3_c0[c4] + (1) 2-Oxobutyrate_c0[c4]	0.0808741	Yes
rxn00781	Glycolysis / Gluconeogenesis|Carbon fixation in photosynthetic organisms	D-glyceraldehyde-3-phosphate:NAD+ oxidoreductase (phosphorylating)	(1) NAD_c0[c4] + (1) Phosphate_c0[c4] + (1) Glyceraldehyde3-phosphate_c0[c4] <-> (1) NADH_c0[c4] + (1) H+_c0[c4] + (1) 1,3-Bisphospho-D-glycerate_c0[c4]	1.67516	Yes
rxn01100	Glycolysis / Gluconeogenesis|Carbon fixation in photosynthetic organisms	ATP:3-phospho-D-glycerate 1-phosphotransferase	(1) ATP_c0[c4] + (1) 3-Phosphoglycerate_c0[c4] <-> (1) ADP_c0[c4] + (1) 1,3-Bisphospho-D-glycerate_c0[c4]	-1.67516	Yes
rxn02342	Glycolysis / Gluconeogenesis|Citrate cycle (TCA cycle)|Pyruvate metabolism	R03270	(1) Lipoamide_c0[c4] + (1) 2-Hydroxyethyl-ThPP_c0[c4] <-> (1) TPP_c0[c4] + (1) S-Acetyldihydrolipoamide_c0[c4]	1.52014	Yes
rxn01871	Glycolysis / Gluconeogenesis|Citrate cycle (TCA cycle)|Pyruvate metabolism	acetyl-CoA:enzyme N6-(dihydrolipoyl)lysine S-acetyltransferase	(1) Acetyl-CoA_c0[c4] + (1) Dihydrolipoamide_c0[c4] <-> (1) CoA_c0[c4] + (1) S-Acetyldihydrolipoamide_c0[c4]	-1.52014	Yes
rxn00011	Glycolysis / Gluconeogenesis|Citrate cycle (TCA cycle)|Valine, leucine and isoleucine biosynthesis|Pyruvate metabolism|Butanoate metabolism	pyruvate:thiamin diphosphate acetaldehydetransferase (decarboxylating)	(1) CO2_c0[c4] + (1) 2-Hydroxyethyl-ThPP_c0[c4] <- (1) Pyruvate_c0[c4] + (1) TPP_c0[c4] + (1) H+_c0[c4]	-1.75291	Yes
rxn00747	Glycolysis / Gluconeogenesis|Fructose and mannose metabolism|Inositol phosphate metabolism|Carbon fixation in photosynthetic organisms	D-glyceraldehyde-3-phosphate aldose-ketose-isomerase	(1) Glyceraldehyde3-phosphate_c0[c4] <-> (1) Glycerone-phosphate_c0[c4]	0.0671937	Yes

rxn00704	Glycolysis / Gluconeogenesis|Galactose metabolism|Starch and sucrose metabolism|Amino sugar and nucleotide sugar metabolism	alpha-D-Glucose 1-phosphate 1,6-phosphomutase	(1) Glucose-1-phosphate_c0[c4] <-> (1) D-glucose-6-phosphate_c0[c4]	-0.0424688	Yes
rxn01106	Glycolysis / Gluconeogenesis|Glycine, serine and threonine metabolism|Methane metabolism	2-Phospho-D-glycerate 2,3-phosphomutase	(1) 2-Phospho-D-glycerate_c0[c4] <-> (1) 3-Phosphoglycerate_c0[c4]	-1.23447	Yes
rxn00459	Glycolysis / Gluconeogenesis|Methane metabolism	2-phospho-D-glycerate hydro-lyase (phosphoenolpyruvate-forming)	(1) 2-Phospho-D-glycerate_c0[c4] <-> (1) H2O_c0[c4] + (1) Phosphoenolpyruvate_c0[c4]	1.23447	Yes
rxn00175	Glycolysis / Gluconeogenesis|Methane metabolism|Carbon fixation pathways in prokaryotes	Acetate:CoA ligase (AMP-forming)	(1) ATP_c0[c4] + (1) CoA_c0[c4] + (1) Acetate_c0[c4] <-> (1) PPi_c0[c4] + (1) AMP_c0[c4] + (1) Acetyl-CoA_c0[c4] + (1) H+_c0[c4]	0.0890644	Yes
rxn00148	Glycolysis / Gluconeogenesis|Purine metabolism|Pyruvate metabolism|Carbon fixation in photosynthetic organisms	ATP:pyruvate 2-O-phosphotransferase	(1) ATP_c0[c4] + (1) Pyruvate_c0[c4] <-> (1) ADP_c0[c4] + (1) Phosphoenolpyruvate_c0[c4] + (1) H+_c0[c4]	2.37544	Yes
rxn00333	Glyoxylate and dicarboxylate metabolism	Glycolate:oxygen 2-oxidoreductase	(1) O2_c0[c4] + (1) Glycolate_c0[c4] -> (1) H2O2_c0[c4] + (1) Glyoxalate_c0[c4]	0.00220832	No
rxn00371	Glyoxylate and dicarboxylate metabolism|Methane metabolism	Formate:NAD+ oxidoreductase	(1) NAD_c0[c4] + (1) Formate_c0[c4] -> (1) NADH_c0[c4] + (1) CO2_c0[c4]	0.0236807	No
rxn12822	Glyoxylate and dicarboxylate metabolism|Nitrogen metabolism	L-glutamate:ferredoxin oxidoreductase (transaminating)	(2) L-Glutamate_c0[c4] + (2) Oxidizedferredoxin_c0[c4] <-> (1) 2-Oxoglutarate_c0[c4] + (1) L-Glutamine_c0[c4] + (2) H+_c0[c4] + (2) Reducedferredoxin_c0[c4]	0.502415	Yes
rxn00691	Glyoxylate and dicarboxylate metabolism|One carbon pool by folate	10-Formyltetrahydrofolate amidohydrolase	(1) H2O_c0[c4] + (1) 10-Formyltetrahydrofolate_c0[c4] -> (1) Formate_c0[c4] + (1) H+_c0[c4] + (1) Tetrahydrofolate_c0[c4]	0.0142165	Yes
rxn02320	Histidine metabolism	5-Amino-2-oxopentanoate:2-oxoglutarate aminotransferase	(1) 2-Oxoglutarate_c0[c4] + (1) L-histidinol-phosphate_c0[c4] <-> (1) L-Glutamate_c0[c4] + (1) imidazole acetol-phosphate_c0[c4]	-0.0269184	Yes
rxn00789	Histidine metabolism	1-(5-phospho-D-ribosyl)-ATP:diphosphate phospho-alpha-D-ribosyl-transferase	(1) PPi_c0[c4] + (1) H+_c0[c4] + (1) Phosphoribosyl-ATP_c0[c4] <- (1) ATP_c0[c4] + (1) PRPP_c0[c4]	-0.0269184	Yes
rxn02160	Histidine metabolism	L-Histidinol-phosphate phosphohydrolase	(1) H2O_c0[c4] + (1) L-histidinol-phosphate_c0[c4] -> (1) Phosphate_c0[c4] + (1) L-Histidinol_c0[c4]	0.0269184	Yes
rxn02159	Histidine metabolism	L-Histidinol:NAD+ oxidoreductase	(1) NAD_c0[c4] + (1) L-Histidinol_c0[c4] <-> (1) NADH_c0[c4] + (1) H+_c0[c4] + (1) L-Histidinal_c0[c4]	0.0269184	Yes
rxn02835	Histidine metabolism	1-(5-phospho-D-ribosyl)-AMP 1,6-hydrolase	(1) H2O_c0[c4] + (1) Phosphoribosyl-AMP_c0[c4] <-> (1) phosphoribosylformiminoaicar-phosphate_c0[c4]	0.0269184	Yes
rxn03175	Histidine metabolism	N-(5'-Phospho-D-ribosylformimino)-5-amino-1- (5''-phospho-D-ribosyl)-4-	(1) H+_c0[c4] + (1) phosphoribosylformiminoaicar-phosphate_c0[c4] <-> (1)	0.0269184	Yes

		imidazolecarboxamide ketol-isomerase	phosphoribulosylformimino-AICAR-phosphate_c0[c4]		
rxn03135	Histidine metabolism	R04558	(1) L-Glutamate_c0[c4] + (2) H+_c0[c4] + (1) D-erythro-imidazol-glycerol-phosphate_c0[c4] + (1) AICAR_c0[c4] <- (1) L-Glutamine_c0[c4] + (1) phosphoribulosylformimino-AICAR-phosphate_c0[c4]	-0.0269184	Yes
rxn00863	Histidine metabolism	L-histidinal:NAD+ oxidoreductase	(1) H2O_c0[c4] + (1) NAD_c0[c4] + (1) L-Histidinal_c0[c4] -> (1) NADH_c0[c4] + (2) H+_c0[c4] + (1) L-Histidine_c0[c4]	0.0269184	Yes
rxn02834	Histidine metabolism	Phosphoribosyl-ATP pyrophosphohydrolase	(1) H2O_c0[c4] + (1) Phosphoribosyl-ATP_c0[c4] -> (1) PPi_c0[c4] + (2) H+_c0[c4] + (1) Phosphoribosyl-AMP_c0[c4]	0.0269184	Yes
rxn02473	Histidine metabolism	D-erythro-1-(Imidazol-4-yl)glycerol 3-phosphate hydro-lyase	(1) D-erythro-imidazol-glycerol-phosphate_c0[c4] -> (1) H2O_c0[c4] + (1) imidazole acetol-phosphate_c0[c4]	0.0269184	Yes
rxn03181	Lipopolysaccharide biosynthesis	ATP:2,3,2',3'-tetrakis(3-hydroxytetradecanoyl)-D-glucosaminyl-beta-D-1,6-glucosaminyl-alpha-phosphate 4-O'-phosphotransferase	(1) ATP_c0[c4] + (1) Lipid A disaccharide_c0[c4] <-> (1) ADP_c0[c4] + (1) H+_c0[c4] + (1) Lipid IV(A)_c0[c4]	0.00849375	Yes
rxn02331	Lipopolysaccharide biosynthesis	phosphoenolpyruvate:D-arabinose-5-phosphate C-(1-carboxyvinyl)transferase (phosphate-hydrolysing, 2-carboxy-2-oxoethyl-forming)	(1) Phosphate_c0[c4] + (1) 3-Deoxy-D-manno-octulosonate8-phosphate_c0[c4] <- (1) H2O_c0[c4] + (1) Phosphoenolpyruvate_c0[c4] + (1) D-Arabinose5-phosphate_c0[c4]	-0.0254813	Yes
rxn02404	Lipopolysaccharide biosynthesis	3-Deoxy-D-manno-octulosonate-8-phosphate 8-phosphohydrolase	(1) H2O_c0[c4] + (1) 3-Deoxy-D-manno-octulosonate8-phosphate_c0[c4] -> (1) Phosphate_c0[c4] + (1) KDO_c0[c4]	0.0254813	Yes
rxn03919	Lipopolysaccharide biosynthesis	D-glycero-beta-D-manno-heptose 1,7-bisphosphate 7-phosphohydrolase	(1) H2O_c0[c4] + (1) D-Glycero-D-manno-heptose1-7-bisphosphate_c0[c4] -> (1) Phosphate_c0[c4] + (1) D-Glycero-D-manno-heptose1-phosphate_c0[c4]	0.033975	Yes
rxn06723	Lipopolysaccharide biosynthesis	(3R)-3-hydroxymyristoyl-[acyl-carrier protein]:UDP-3-O-[(3R)-3-hydroxymyristoyl]-alpha-D-glucosamine N-acetyltransferase	(1) UDP-2,3-bis(3-hydroxytetradecanoyl)glucosamine_c0[c4] + (1) ACP_c0[c4] <- (1) UDP-3-O-(beta-hydroxymyristoyl)-D-glucosamine_c0[c4] + (1) HMA_c0[c4]	-0.0169875	Yes
rxn03146	Lipopolysaccharide biosynthesis	UDP-3-O-[(3R)-3-hydroxymyristoyl]-N-acetylglucosamine amidohydrolase	(1) H2O_c0[c4] + (1) UDP-3-O-(beta-hydroxymyristoyl)-N-acetylglucosamine_c0[c4] <-> (1) Acetate_c0[c4] + (1) UDP-3-O-(beta-hydroxymyristoyl)-D-glucosamine_c0[c4]	0.0169875	Yes
rxn02405	Lipopolysaccharide biosynthesis	CTP:3-deoxy-D-manno-octulosonate cytidylyltransferase	(1) CTP_c0[c4] + (1) KDO_c0[c4] -> (1) PPi_c0[c4] + (1) H+_c0[c4] + (1) CMP-KDO_c0[c4]	0.0254813	Yes
rxn06865	Lipopolysaccharide biosynthesis	R05146	(1) Lauroyl-KDO2-lipid IV(A)_c0[c4] + (1) ACP_c0[c4] <- (1) kdo2-lipid iva_c0[c4] + (1) Dodecanoyl-ACP_c0[c4]	-0.00849375	Yes
rxn06729	Lipopolysaccharide biosynthesis	(R)-3-Hydroxytetradecanoly-[acyl-carrier-protein]:UDP-	(1) UDP-N-acetylglucosamine_c0[c4] + (1) H+_c0[c4] + (1) HMA_c0[c4] -> (1) UDP-3-O-(beta-hydroxymyristoyl)-N-	0.0169875	Yes

		N-acetyl-glucosamine 3-O-(3-hydroxytetradecanoyl)transferase	acetylglucosamine_c0[c4] + (1) ACP_c0[c4]		
rxn03130	Lipopolysaccharide biosynthesis	UDP-2,3-bis[(3R)-3-hydroxymyristoyl]-alpha-D-glucosamine 2,3-bis[(3R)-3-hydroxymyristoyl]-beta-D-glucosaminyl 1-phosphate phosphohydrolase	(1) H2O_c0[c4] + (1) UDP-2,3-bis(3-hydroxytetradecanoyl)glucosamine_c0[c4] -> (2) H+_c0[c4] + (1) UMP_c0[c4] + (1) Lipid X_c0[c4]	0.00849375	Yes
rxn03159	Lipopolysaccharide biosynthesis	UDP-2,3-bis(3-hydroxytetradecanoyl)glucosamine:2,3-bis-(3-hydroxytetradecanoyl)-alpha-D-glucosaminyl-1-phosphate 2,3-bis(3-hydroxytetradecanoyl)-glucosaminyltransferase	(1) UDP-2,3-bis(3-hydroxytetradecanoyl)glucosamine_c0[c4] + (1) Lipid X_c0[c4] <-> (1) UDP_c0[c4] + (1) Lipid A disaccharide_c0[c4]	0.00849375	No
rxn03916	Lipopolysaccharide biosynthesis	ATP:D-glycero-beta-D-manno-heptose 1-phosphate adenylyltransferase	(1) ATP_c0[c4] + (1) D-Glycero-D-manno-heptose1-phosphate_c0[c4] -> (1) PPi_c0[c4] + (1) ADP-D-glycero-D-manno-heptose[c4]	0.033975	No
rxn03182	Lipopolysaccharide biosynthesis	CMP-3-deoxy-D-manno-oct-2-ulosonate:lipid IVA 3-deoxy-D-manno-oct-2-ulosonate transferase	(1) CMP-KDO_c0[c4] + (1) Lipid IV(A)_c0[c4] -> (1) CMP_c0[c4] + (1) H+_c0[c4] + (1) KDO-lipid IV(A)[c4]	0.00849375	No
rxn06848	Lipopolysaccharide biosynthesis	R05075	(1) kdo2-lipid a_c0[c4] + (1) ACP_c0[c4] <- (1) Lauroyl-KDO2-lipid IV(A)_c0[c4] + (1) Myristoyl-ACP_c0[c4]	-0.00849375	No
rxn03511	Lipopolysaccharide biosynthesis	ADP-D-glycero-D-manno-heptose 6-epimerase	(1) ADP-D-glycero-D-manno-heptose[c4] -> (1) ADP-L-glycero-D-manno-heptose_c0[c4]	0.033975	No
rxn03918	Lipopolysaccharide biosynthesis	ATP:D-glycero-beta-D-manno-heptose 7-phosphate 1-phosphotransferase	(1) ATP_c0[c4] + (1) D-Glycero-D-manno-heptose7-phosphate[c4] -> (1) ADP_c0[c4] + (1) H+_c0[c4] + (1) D-Glycero-D-manno-heptose1-7-bisphosphate_c0[c4]	0.033975	No
rxn03439	Lipopolysaccharide biosynthesis	CMP-3-deoxy-D-manno-oct-2-ulosonate:(KDO)-lipid IVA 3-deoxy-D-manno-oct-2-ulosonate transferase	(1) CMP-KDO_c0[c4] + (1) KDO-lipid IV(A)[c4] -> (1) CMP_c0[c4] + (1) H+_c0[c4] + (1) kdo2-lipid iva_c0[c4]	0.00849375	No
rxn03917	Lipopolysaccharide biosynthesis	D-glycero-beta-D-manno-heptose-7-phosphate aldose-ketose-isomerase	(1) Sedoheptulose7-phosphate_c0[c4] -> (1) D-Glycero-D-manno-heptose7-phosphate[c4]	0.033975	No
rxn00313	Lysine biosynthesis	meso-2,6-diaminoheptanedioate carboxy-lyase (L-lysine-forming)	(1) H+_c0[c4] + (1) meso-2,6-Diaminopimelate_c0[c4] -> (1) CO2_c0[c4] + (1) L-Lysine_c0[c4]	0.10663	Yes
rxn01644	Lysine biosynthesis	L-Aspartate-4-semialdehyde hydro-lyase (adding pyruvate and cyclizing)	(1) Pyruvate_c0[c4] + (1) L-Aspartate4-semialdehyde_c0[c4] -> (2) H2O_c0[c4] + (1) H+_c0[c4] + (1) Dihydrodipicolinate[c4]	0.115124	No
rxn02928	Lysine biosynthesis	2,3,4,5-Tetrahydrodipicolinate:NAD+ oxidoreductase	(1) NAD_c0[c4] + (1) tetrahydrodipicolinate_c0[c4] <- (1) NADH_c0[c4] + (1) H+_c0[c4] + (1) Dihydrodipicolinate[c4]	-0.115124	No
rxn02011	Lysine biosynthesis|Peptidoglycan biosynthesis	UDP-N-acetylmuramoyl-L-alanyl-D-glutamate:(L)-meso-2,6-diaminoheptanedioate	(1) ATP_c0[c4] + (1) meso-2,6-Diaminopimelate_c0[c4] + (1) UDP-N-acetylmuramoyl-L-alanyl-D-glutamate_c0[c4] -> (1) ADP_c0[c4] +	0.00849375	Yes

		gamma-ligase (ADP-forming)	(1) Phosphate_c0[c4] + (1) H+_c0[c4] + (1) UDP-N-acetylmuramoyl-L-alanyl-D-gamma-glutamyl-meso-2-6-diaminopimelate_c0[c4]		
rxn03164	Lysine biosynthesis|Peptidoglycan biosynthesis	UDP-N-acetylmuramoyl-L-alanyl-D-glutamyl-meso-2,6-diaminoheptanedioate:D-alanyl-D-alanine ligase(ADP-forming)	(1) ATP_c0[c4] + (1) Ala-Ala[c4] + (1) UDP-N-acetylmuramoyl-L-alanyl-D-gamma-glutamyl-meso-2-6-diaminopimelate_c0[c4] -> (1) ADP_c0[c4] + (1) Phosphate_c0[c4] + (1) H+_c0[c4] + (1) UDP-N-acetylmuramoyl-L-alanyl-D-glutamyl-6-carboxy-L-lysyl-D-alanyl- D-alanine_c0[c4]	0.00849375	No
rxn02988	Nicotinate and nicotinamide metabolism	glycerone phosphate:iminosuccinate alkyltransferase (cyclizing)	(2) H2O_c0[c4] + (1) Phosphate_c0[c4] + (1) Quinolinate_c0[c4] <- (1) Glycerone-phosphate_c0[c4] + (1) Iminoaspartate_c0[c4]	-0.00210316	Yes
rxn02402	Nicotinate and nicotinamide metabolism	Nicotinate-nucleotide:pyrophosphate phosphoribosyltransferase (carboxylating)	(1) CO2_c0[c4] + (1) PPi_c0[c4] + (1) Nicotinate ribonucleotide_c0[c4] <- (1) H+_c0[c4] + (1) PRPP_c0[c4] + (1) Quinolinate_c0[c4]	-0.00210316	Yes
rxn00083	Nicotinate and nicotinamide metabolism	NADPH:NAD+ oxidoreductase	(1) NAD_c0[c4] + (1) NADPH_c0[c4] <-> (1) NADH_c0[c4] + (1) NADP_c0[c4]	-0.147993	Yes
rxn01265	Nicotinate and nicotinamide metabolism	Nicotinate D-ribonucleotide:diphosphate phosphoribosyltransferase	(1) PPi_c0[c4] + (1) Nicotinate ribonucleotide_c0[c4] <-> (1) PRPP_c0[c4] + (1) Niacin_c0[c4]	-0.00105158	Yes
rxn00077	Nicotinate and nicotinamide metabolism	ATP:NAD+ 2'-phosphotransferase	(1) ATP_c0[c4] + (1) NAD_c0[c4] <-> (1) NADP_c0[c4] + (1) ADP_c0[c4] + (1) H+_c0[c4]	0.00105158	Yes
rxn00338	Nicotinate and nicotinamide metabolism	L-aspartate:oxygen oxidoreductase	(1) O2_c0[c4] + (1) L-Aspartate_c0[c4] -> (1) H2O2_c0[c4] + (1) H+_c0[c4] + (1) Iminoaspartate_c0[c4]	0.00210316	Yes
rxn00138	Nicotinate and nicotinamide metabolism	deamido-NAD+:ammonia ligase (AMP-forming)	(1) ATP_c0[c4] + (1) NH3_c0[c4] + (1) Deamido-NAD_c0[c4] -> (1) NAD_c0[c4] + (1) PPi_c0[c4] + (1) AMP_c0[c4] + (2) H+_c0[c4]	0.00210316	Yes
rxn02155	Nicotinate and nicotinamide metabolism	ATP:nicotinamide-nucleotide adenylyltransferase	(1) ATP_c0[c4] + (1) Nicotinate ribonucleotide_c0[c4] <-> (1) PPi_c0[c4] + (1) Deamido-NAD_c0[c4]	0.00210316	No
rxn00102	Nitrogen metabolism	carbonate hydro-lyase (carbon-dioxide-forming)	(1) H+_c0[c4] + (1) H2CO3_c0[c4] <-> (1) H2O_c0[c4] + (1) CO2_c0[c4]	-0.804936	Yes
rxn00907	One carbon pool by folate|Carbon fixation pathways in prokaryotes	5,10-methylenetetrahydrofolate:NADP+ oxidoreductase	(1) NADP_c0[c4] + (1) 5-10-Methylenetetrahydrofolate_c0[c4] <-> (1) NADPH_c0[c4] + (1) 5-10-Methenyltetrahydrofolate_c0[c4]	0.264719	Yes
rxn01211	One carbon pool by folate|Carbon fixation pathways in prokaryotes	5,10-Methenyltetrahydrofolate 5-hydrolase (decyclizing)	(1) H2O_c0[c4] + (1) 5-10-Methenyltetrahydrofolate_c0[c4] <-> (1) H+_c0[c4] + (1) 10-Formyltetrahydrofolate_c0[c4]	0.264719	Yes
rxn00686	One carbon pool by folate|Folate biosynthesis	5,6,7,8-tetrahydrofolate:NADP+ oxidoreductase	(1) NADP_c0[c4] + (1) Tetrahydrofolate_c0[c4] <-> (1) NADPH_c0[c4] + (1) H+_c0[c4] + (1) Dihydrofolate_c0[c4]	-0.00720645	Yes
rxn04954	One carbon pool by folate|Methane metabolism|Carbon fixation pathways in prokaryotes	5-methyltetrahydrofolate:NAD+ oxidoreductase	(1) NAD_c0[c4] + (1) 5-Methyltetrahydrofolate_c0[c4] <-> (1) NADH_c0[c4] + (1) H+_c0[c4] + (1) 5-10-Methylenetetrahydrofolate_c0[c4]	-0.0518148	Yes
rxn00898	Pantothenate and CoA biosynthesis	2,3-Dihydroxy-3-methylbutanoate hydro-lyase	(1) 2,3-Dihydroxy-isovalerate_c0[c4] -> (1) H2O_c0[c4] + (1) 3-Methyl-2-oxobutanoate_c0[c4]	0.121724	Yes

rxn12512	Pantothenate and CoA biosynthesis	(R)-4'-Phosphopantothenate:L-cysteine ligase	(1) ATP_c0[c4] + (1) L-Cysteine_c0[c4] + (1) 4-phosphopantothenate_c0[c4] -> (1) PPi_c0[c4] + (1) AMP_c0[c4] + (2) H+_c0[c4] + (1) (R)-4'-Phosphopantothenoyl-L-cysteine_c0[c4]	0.00210316	Yes
rxn02175	Pantothenate and CoA biosynthesis	ATP:pantetheine-4'-phosphate adenylyltransferase	(1) ATP_c0[c4] + (1) Phosphopantetheine_c0[c4] <-> (1) PPi_c0[c4] + (1) Dephospho-CoA_c0[c4]	0.00210316	Yes
rxn01790	Pantothenate and CoA biosynthesis	(R)-Pantoate:NADP+ 2-oxidoreductase	(1) NADP_c0[c4] + (1) Pantoate_c0[c4] <-> (1) NADPH_c0[c4] + (1) H+_c0[c4] + (1) 2-Dehydropantoate_c0[c4]	-0.00210316	Yes
rxn00912	Pantothenate and CoA biosynthesis	5,10-Methylenetetrahydrofolate:3-methyl-2-oxobutanoate hydroxymethyltransferase	(1) H2O_c0[c4] + (1) 3-Methyl-2-oxobutanoate_c0[c4] + (1) 5-10-Methylenetetrahydrofolate_c0[c4] <-> (1) Tetrahydrofolate_c0[c4] + (1) 2-Dehydropantoate_c0[c4]	0.00210316	Yes
rxn02341	Pantothenate and CoA biosynthesis	N-[(R)-4'-Phosphopantothenoyl]-L-cysteine carboxy-lyase	(1) H+_c0[c4] + (1) (R)-4'-Phosphopantothenoyl-L-cysteine_c0[c4] -> (1) CO2_c0[c4] + (1) Phosphopantetheine_c0[c4]	0.00210316	Yes
rxn02186	Pantothenate and CoA biosynthesis	2,3-Dihydroxy-3-methylbutanoate:NADP+ oxidoreductase (isomerizing)	(1) NADPH_c0[c4] + (1) H+_c0[c4] + (1) ALCTT_c0[c4] <-> (1) NADP_c0[c4] + (1) 2,3-Dihydroxy-isovalerate_c0[c4]	0.121724	Yes
rxn00100	Pantothenate and CoA biosynthesis	ATP:dephospho-CoA 3'-phosphotransferase	(1) ATP_c0[c4] + (1) Dephospho-CoA_c0[c4] -> (1) ADP_c0[c4] + (1) CoA_c0[c4] + (1) H+_c0[c4]	0.00210316	No
rxn06023	Pantothenate and CoA biosynthesis	CoA:apo-[acyl-carrier-protein] pantetheinephosphotransferase	(1) CoA_c0[c4] + (1) apo-ACP_c0[c4] <-> (1) Adenosine 3-5-bisphosphate_c0[c4] + (1) ACP_c0[c4]	0.00105158	No
rxn12510	Pantothenate and CoA biosynthesis	ATP:pantothenate 4'-phosphotransferase	(1) ATP_c0[c4] + (1) PAN_c0[c4] <-> (1) ADP_c0[c4] + (1) H+_c0[c4] + (1) 4-phosphopantothenate_c0[c4]	0.00210316	No
rxn00213	Pentose and glucuronate interconversions|Galactose metabolism|Starch and sucrose metabolism|Amino sugar and nucleotide sugar metabolism	UTP:alpha-D-glucose-1-phosphate uridylyltransferase	(1) UTP_c0[c4] + (1) Glucose-1-phosphate_c0[c4] <-> (1) PPi_c0[c4] + (1) UDP-glucose_c0[c4]	0.033975	Yes
rxn01477	Pentose phosphate pathway	6-Phospho-D-gluconate hydro-lyase(2-dehydro-3-deoxy-6-phospho-D-gluconate-forming)	(1) 6-Phospho-D-gluconate_c0[c4] -> (1) H2O_c0[c4] + (1) 2-Keto-3-deoxy-6-phosphogluconate_c0[c4]	2.26949	Yes
rxn01476	Pentose phosphate pathway	6-Phospho-D-glucono-1,5-lactone lactonohydrolase	(1) H2O_c0[c4] + (1) 6-phospho-D-glucono-1-5-lactone_c0[c4] -> (1) H+_c0[c4] + (1) 6-Phospho-D-gluconate_c0[c4]	1.50373	Yes
rxn01333	Pentose phosphate pathway	sedoheptulose-7-phosphate:D-glyceraldehyde-3-phosphate glyceronetransferase	(1) Glyceraldehyde3-phosphate_c0[c4] + (1) Sedoheptulose7-phosphate_c0[c4] <-> (1) D-fructose-6-phosphate_c0[c4] + (1) D-Erythrose4-phosphate_c0[c4]	-0.315789	Yes
rxn00772	Pentose phosphate pathway	ATP:D-ribose 5-phosphotransferase	(1) ATP_c0[c4] + (1) D-Ribose_c0[c4] <-> (1) ADP_c0[c4] + (1) H+_c0[c4] + (1) ribose-5-phosphate_c0[c4]	0.0629103	No
rxn00777	Pentose phosphate pathway|Carbon fixation in photosynthetic organisms	D-ribose-5-phosphate aldose-ketose-isomerase	(1) ribose-5-phosphate_c0[c4] <-> (1) D-Ribulose5-phosphate_c0[c4]	0.0584158	Yes

rxn01200	Pentose phosphate pathway|Carbon fixation in photosynthetic organisms	Sedoheptulose-7-phosphate:D-glyceraldehyde-3-phosphate glycolaldehyde transferase	(1) Glyceraldehyde3-phosphate_c0[c4] + (1) Sedoheptulose7-phosphate_c0[c4] <-> (1) ribose-5-phosphate_c0[c4] + (1) D-Xylulose5-phosphate_c0[c4]	0.281814	Yes
rxn03884	Pentose phosphate pathway|Pentose and glucuronate interconversions	2-dehydro-3-deoxy-D-gluconate-6-phosphate D-glyceraldehyde-3-phosphate-lyase	(1) 2-Keto-3-deoxy-6-phosphogluconate_c0[c4] <-> (1) Pyruvate_c0[c4] + (1) Glyceraldehyde3-phosphate_c0[c4]	2.26949	Yes
rxn01116	Pentose phosphate pathway|Pentose and glucuronate interconversions|Carbon fixation in photosynthetic organisms	D-Ribulose-5-phosphate 3-epimerase	(1) D-Ribulose5-phosphate_c0[c4] <-> (1) D-Xylulose5-phosphate_c0[c4]	-0.738079	Yes
rxn00770	Pentose phosphate pathway|Purine metabolism	ATP:D-ribose-5-phosphate diphosphotransferase	(1) ATP_c0[c4] + (1) ribose-5-phosphate_c0[c4] <-> (1) AMP_c0[c4] + (1) H+_c0[c4] + (1) PRPP_c0[c4]	0.286308	Yes
rxn03904	Peptidoglycan biosynthesis	UDP-N-acetylmuramoyl-L-alanyl-gamma-D-glutamyl-meso-2,6-diaminopimeloyl-D-alanyl-D-alanine:undecaprenyl-phosphate phospho-N-acetylmuramoyl-pentapeptide-transferase	(1) Undecaprenylphosphate_c0[c4] + (1) UDP-N-acetylmuramoyl-L-alanyl-D-glutamyl-6-carboxy-L-lysyl-D-alanyl- D-alanine_c0[c4] <-> (1) UMP_c0[c4] + (1) Undecaprenyl-diphospho-N-acetylmuramoyl-L-alanyl-D-glutamyl-meso-2-6-diaminopimeloyl-D-alanyl-D-alanine_c0[c4]	0.00849375	Yes
rxn03901	Peptidoglycan biosynthesis	undecaprenyl-diphosphate phosphohydrolase	(1) H2O_c0[c4] + (1) Bactoprenyl diphosphate_c0[c4] -> (1) Phosphate_c0[c4] + (2) H+_c0[c4] + (1) Undecaprenylphosphate_c0[c4]	0.00849375	Yes
rxn03408	Peptidoglycan biosynthesis	UDP-N-acetyl-D-glucosamine:undecaprenyl-diphospho-N-acetylmuramoyl-L-alanyl-gamma-D-glutamyl-meso-2,6-diaminopimeloyl-D-alanyl-D-alanine 4-beta-N-acetylglucosaminlytransferase	(1) UDP-N-acetylglucosamine_c0[c4] + (1) Undecaprenyl-diphospho-N-acetylmuramoyl-L-alanyl-D-glutamyl-meso-2-6-diaminopimeloyl-D-alanyl-D-alanine_c0[c4] <-> (1) UDP_c0[c4] + (1) Undecaprenyl-diphospho-N-acetylmuramoyl--N-acetylglucosamine-L-ala-D-glu-meso-2-6-diaminopimeloyl-D-ala-D-ala_c0[c4]	0.00849375	No
rxn00493	Phenylalanine metabolism|Phenylalanine, tyrosine and tryptophan biosynthesis|Tropane, piperidine and pyridine alkaloid biosynthesis	L-Phenylalanine:2-oxoglutarate aminotransferase	(1) 2-Oxoglutarate_c0[c4] + (1) L-Phenylalanine_c0[c4] <-> (1) L-Glutamate_c0[c4] + (1) Phenylpyruvate_c0[c4]	-0.0655947	Yes
rxn02212	Phenylalanine, tyrosine and tryptophan biosynthesis	2-Dehydro-3-deoxy-D-arabino-heptonate 7-phosphate phosphate-lyase (cyclyzing)	(1) DAHP_c0[c4] -> (1) Phosphate_c0[c4] + (1) 5-Dehydroquinate_c0[c4]	0.140477	Yes
rxn00727	Phenylalanine, tyrosine and tryptophan biosynthesis	Chorismate pyruvate-lyase (amino-accepting)	(1) L-Glutamine_c0[c4] + (1) Chorismate_c0[c4] -> (1) Pyruvate_c0[c4] + (1) L-Glutamate_c0[c4] + (1) H+_c0[c4] + (1) Anthranilate_c0[c4]	0.0160301	Yes
rxn01269	Phenylalanine, tyrosine and tryptophan biosynthesis	Prephenate:NADP+ oxidoreductase(decarboxylating)	(1) NADP_c0[c4] + (1) Prephenate_c0[c4] -> (1) NADPH_c0[c4] + (1) CO2_c0[c4] + (1) p-hydroxyphenylpyruvate_c0[c4]	0.0525426	Yes
rxn01740	Phenylalanine, tyrosine and tryptophan biosynthesis	Shikimate:NADP+ 3-oxidoreductase	(1) NADP_c0[c4] + (1) Shikimate_c0[c4] <-> (1) NADPH_c0[c4] + (1) H+_c0[c4] + (1) 3-Dehydroshikimate_c0[c4]	-0.140477	Yes

rxn01000	Phenylalanine, tyrosine and tryptophan biosynthesis	prephenate hydro-lyase (decarboxylating; phenylpyruvate-forming)	(1) H+_c0[c4] + (1) Prephenate_c0[c4] -> (1) H2O_c0[c4] + (1) CO2_c0[c4] + (1) Phenylpyruvate_c0[c4]	0.0655947	Yes
rxn01255	Phenylalanine, tyrosine and tryptophan biosynthesis	5-O-(1-Carboxyvinyl)-3-phosphoshikimate phosphate-lyase (chorismate-forming)	(1) 5-O--1-Carboxyvinyl-3-phosphoshikimate_c0[c4] -> (1) Phosphate_c0[c4] + (1) Chorismate_c0[c4]	0.140477	Yes
rxn00791	Phenylalanine, tyrosine and tryptophan biosynthesis	N-(5-Phospho-D-ribosyl)anthranilate:pyrophosphate phosphoribosyl-transferase	(1) PPi_c0[c4] + (1) H+_c0[c4] + (1) N-5-phosphoribosyl-anthranilate_c0[c4] <- (1) Anthranilate_c0[c4] + (1) PRPP_c0[c4]	-0.0160301	Yes
rxn02476	Phenylalanine, tyrosine and tryptophan biosynthesis	Phosphoenolpyruvate:3-phosphoshikimate 5-O-(1-carboxyvinyl)-transferase	(1) Phosphoenolpyruvate_c0[c4] + (1) 3-phosphoshikimate_c0[c4] <-> (1) Phosphate_c0[c4] + (1) 5-O--1-Carboxyvinyl-3-phosphoshikimate_c0[c4]	0.140477	Yes
rxn01332	Phenylalanine, tyrosine and tryptophan biosynthesis	Phosphoenolpyruvate:D-erythrose-4-phosphate C-(1-carboxyvinyl)transferase (phosphate hydrolysing, 2-carboxy-2-oxoethyl-forming)	(1) H2O_c0[c4] + (1) Phosphoenolpyruvate_c0[c4] + (1) D-Erythrose4-phosphate_c0[c4] -> (1) Phosphate_c0[c4] + (1) DAHP_c0[c4]	0.140477	Yes
rxn02507	Phenylalanine, tyrosine and tryptophan biosynthesis	1-(2-Carboxyphenylamino)-1-deoxy-D-ribulose-5-phosphate carboxy-lyase(cyclizing)	(1) H+_c0[c4] + (1) 1-(2-carboxyphenylamino)-1-deoxyribulose 5-phosphate_c0[c4] -> (1) H2O_c0[c4] + (1) CO2_c0[c4] + (1) Indoleglycerol phosphate_c0[c4]	0.0160301	Yes
rxn01739	Phenylalanine, tyrosine and tryptophan biosynthesis	ATP:shikimate 3-phosphotransferase	(1) ATP_c0[c4] + (1) Shikimate_c0[c4] <-> (1) ADP_c0[c4] + (1) H+_c0[c4] + (1) 3-phosphoshikimate_c0[c4]	0.140477	No
rxn01256	Phenylalanine, tyrosine and tryptophan biosynthesis	Chorismate pyruvatemutase	(1) Chorismate_c0[c4] -> (1) Prephenate_c0[c4]	0.118137	No
rxn02508	Phenylalanine, tyrosine and tryptophan biosynthesis	N-(5-Phospho-beta-D-ribosyl)anthranilate ketol-isomerase	(1) N-5-phosphoribosyl-anthranilate_c0[c4] <-> (1) 1-(2-carboxyphenylamino)-1-deoxyribulose 5-phosphate_c0[c4]	0.0160301	No
rxn02213	Phenylalanine, tyrosine and tryptophan biosynthesis	3-Dehydroquinate hydro-lyase	(1) 5-Dehydroquinate_c0[c4] -> (1) H2O_c0[c4] + (1) 3-Dehydroshikimate_c0[c4]	0.140477	No
rxn00060	Porphyrin and chlorophyll metabolism	porphobilinogen:(4-[2-carboxyethyl]-3-[carboxymethyl]pyrrol-2-yl)methyltransferase (hydrolysing)	(1) H2O_c0[c4] + (4) Porphobilinogen_c0[c4] -> (4) NH3_c0[c4] + (1) Hydroxymethylbilane_c0[c4]	0.00210316	Yes
rxn00029	Porphyrin and chlorophyll metabolism	5-aminolevulinate hydro-lyase (adding 5-aminolevulinate and cyclizing; porphobilinogen-forming)	(2) 5-Aminolevulinate_c0[c4] -> (2) H2O_c0[c4] + (1) H+_c0[c4] + (1) Porphobilinogen_c0[c4]	0.00841263	Yes
rxn01629	Porphyrin and chlorophyll metabolism	(S)-4-Amino-5-oxopentanoate 4,5-aminomutase	(1) 5-Aminolevulinate_c0[c4] <-> (1) L-Glutamate1-semialdehyde_c0[c4]	-0.0168253	Yes
rxn03150	Porphyrin and chlorophyll metabolism	R04594	(1) H2O_c0[c4] + (1) alpha-Ribazole 5'-phosphate_c0[c4] -> (1) Phosphate_c0[c4] + (1) alpha-Ribazole_c0[c4]	0.00105158	Yes
rxn05029	Porphyrin and chlorophyll metabolism	ATP:cobinamide Cobeta-adenosyltransferase	(1) ATP_c0[c4] + (1) H+_c0[c4] + (1) Cobinamide_c0[c4] <-> (1) Triphosphate_c0[c4] + (1) Adenosyl cobinamide_c0[c4]	0.00105158	Yes
rxn02303	Porphyrin and chlorophyll metabolism	Coproporphyrinogen:oxygen	(1) O2_c0[c4] + (2) H+_c0[c4] + (1) CoproporphyrinogenIII_c0[c4] <-> (2)	0.00105158	Yes

		oxidoreductase(decarboxylating)	H2O_c0[c4] + (2) CO2_c0[c4] + (1) ProtoporphyrinogenIX_c0[c4]		
rxn00056	Porphyrin and chlorophyll metabolism	Fe(II):oxygen oxidoreductase	(1) O2_c0[c4] + (4) H+_c0[c4] + (4) Fe2+_c0[c4] <-> (2) H2O_c0[c4] + (4) fe3_c0[c4]	-0.000788684	Yes
rxn02897	Porphyrin and chlorophyll metabolism	Nicotinate-nucleotide:dimethylbenzimidazole phospho-D-ribosyltransferase	(1) Nicotinate ribonucleotide_c0[c4] + (1) Dimethylbenzimidazole_c0[c4] <-> (1) H+_c0[c4] + (1) Niacin_c0[c4] + (1) alpha-Ribazole 5'-phosphate_c0[c4]	0.00105158	Yes
rxn02056	Porphyrin and chlorophyll metabolism	S-Adenosyl-L-methionine:uroporphyrin-III C-methyltransferase	(2) H+_c0[c4] + (1) Siroheme_c0[c4] <-> (1) Sirohydrochlorin_c0[c4] + (1) Fe2+_c0[c4]	-0.00105158	Yes
rxn02288	Porphyrin and chlorophyll metabolism	Uroporphyrinogen-III carboxy-lyase	(4) H+_c0[c4] + (1) UroporphyrinogenIII_c0[c4] <-> (4) CO2_c0[c4] + (1) CoproporphyrinogenIII_c0[c4]	0.00105158	Yes
rxn06591	Porphyrin and chlorophyll metabolism	L-glutamate-semialdehyde: NADP+ oxidoreductase(L-glutamyl-tRNA(Glu)-forming)	(1) NADPH_c0[c4] + (1) H+_c0[c4] + (1) L-Glutamyl-tRNA-Glu_c0[c4] <-> (1) NADP_c0[c4] + (1) L-Glutamate1-semialdehyde_c0[c4] + (1) tRNA-Glu_c0[c4]	0.0168253	Yes
rxn00224	Porphyrin and chlorophyll metabolism	protoheme ferro-lyase (protoporphyrin-forming)	(1) Protoporphyrin_c0[c4] + (1) Fe2+_c0[c4] <-> (1) Heme_c0[c4] + (2) H+_c0[c4]	0.00105158	Yes
rxn02264	Porphyrin and chlorophyll metabolism	Hydroxymethylbilane hydro-lyase(cyclizing)	(1) Hydroxymethylbilane_c0[c4] <-> (1) H2O_c0[c4] + (1) UroporphyrinogenIII_c0[c4]	0.00210316	No
rxn03537	Porphyrin and chlorophyll metabolism	R05222	(1) GTP_c0[c4] + (1) Adenosyl cobinamide phosphate_c0[c4] <-> (1) PPi_c0[c4] + (1) H+_c0[c4] + (1) Adenosylcobinamide-GDP_c0[c4]	0.00105158	No
rxn04413	Porphyrin and chlorophyll metabolism	R06558	(1) GTP_c0[c4] + (1) Adenosyl cobinamide_c0[c4] <-> (1) GDP_c0[c4] + (1) H+_c0[c4] + (1) Adenosyl cobinamide phosphate_c0[c4]	0.00105158	No
rxn03538	Porphyrin and chlorophyll metabolism	R05223	(1) alpha-Ribazole_c0[c4] + (1) Adenosylcobinamide-GDP_c0[c4] <-> (1) H+_c0[c4] + (1) GMP_c0[c4] + (1) Calomide_c0[c4]	0.00105158	No
rxn02304	Porphyrin and chlorophyll metabolism	protoporphyrinogen-IX:oxygen oxidoreductase	(3) O2_c0[c4] + (2) ProtoporphyrinogenIX_c0[c4] -> (6) H2O_c0[c4] + (2) Protoporphyrin_c0[c4]	0.00052579	No
rxn02774	Porphyrin and chlorophyll metabolism	S-Adenosyl-L-methionine:uroporphyrin-III C-methyltransferase	(1) NAD_c0[c4] + (1) Precorrin 2_c0[c4] -> (1) NADH_c0[c4] + (2) H+_c0[c4] + (1) Sirohydrochlorin_c0[c4]	0.00105158	No
rxn06937	Porphyrin and chlorophyll metabolism|Aminoacyl-tRNA biosynthesis	L-glutamate:tRNA(Glu) ligase (AMP-forming)	(1) ATP_c0[c4] + (1) L-Glutamate_c0[c4] + (1) tRNA-Glu_c0[c4] -> (1) PPi_c0[c4] + (1) AMP_c0[c4] + (1) H+_c0[c4] + (1) L-Glutamyl-tRNA-Glu_c0[c4]	0.0168253	Yes
rxn01297	Purine metabolism	hypoxanthine:NAD+ oxidoreductase	(1) H2O_c0[c4] + (1) NAD_c0[c4] + (1) HYXN_c0[c4] <-> (1) NADH_c0[c4] + (1) H+_c0[c4] + (1) XAN_c0[c4]	0.00147033	Yes
rxn00097	Purine metabolism	ATP:AMP phosphotransferase	(1) ATP_c0[c4] + (1) AMP_c0[c4] + (1) H+_c0[c4] <-> (2) ADP_c0[c4]	0.797626	Yes
rxn01545	Purine metabolism	Xanthosine ribohydrolase	(1) H2O_c0[c4] + (1) Xanthosine_c0[c4] <-> (1) D-Ribose_c0[c4] + (1) XAN_c0[c4]	0.0566008	Yes

rxn00915	Purine metabolism	GMP:diphosphate 5-phospho-alpha-D-ribosyltransferase	(1) PPi_c0[c4] + (1) H+_c0[c4] + (1) GMP_c0[c4] <- (1) PRPP_c0[c4] + (1) Guanine_c0[c4]	-0.0580712	Yes
rxn03147	Purine metabolism	1-(5-Phosphoribosyl)-5-amino-4-carboxyimidazole:L-aspartate ligase (ADP-forming)	(1) ATP_c0[c4] + (1) L-Aspartate_c0[c4] + (1) 5'-Phosphoribosyl-4-carboxy-5-aminoimidazole_c0[c4] -> (1) ADP_c0[c4] + (1) Phosphate_c0[c4] + (1) H+_c0[c4] + (1) SAICAR_c0[c4]	0.110688	Yes
rxn00832	Purine metabolism	IMP 1,2-hydrolase (decyclizing)	(1) H2O_c0[c4] + (1) IMP_c0[c4] <-> (1) FAICAR_c0[c4]	-0.137606	Yes
rxn02895	Purine metabolism	5-Phospho-D-ribosylamine:glycine ligase (ADP-forming)	(1) ATP_c0[c4] + (1) Glycine_c0[c4] + (1) 5-Phosphoribosylamine_c0[c4] -> (1) ADP_c0[c4] + (1) Phosphate_c0[c4] + (1) H+_c0[c4] + (1) GAR_c0[c4]	0.111845	Yes
rxn03136	Purine metabolism	1-(5'-Phosphoribosyl)-5-amino-4-(N-succinocarboxamide)-imidazole AMP-lyase	(1) SAICAR_c0[c4] <-> (1) H+_c0[c4] + (1) Fumarate_c0[c4] + (1) AICAR_c0[c4]	0.110688	Yes
rxn02937	Purine metabolism	2-(Formamido)-N1-(5-phosphoribosyl)acetamidine cyclo-ligase (ADP-forming)	(1) ATP_c0[c4] + (1) 5'-Phosphoribosylformylglycinamidine_c0[c4] <-> (1) ADP_c0[c4] + (1) Phosphate_c0[c4] + (1) H+_c0[c4] + (1) AIR_c0[c4]	0.111845	Yes
rxn01961	Purine metabolism	Xanthosine 5'-phosphate phosphohydrolase	(1) H2O_c0[c4] + (1) XMP_c0[c4] -> (1) Phosphate_c0[c4] + (1) Xanthosine_c0[c4]	0.0566008	Yes
rxn01299	Purine metabolism	Inosine ribohydrolase	(1) H2O_c0[c4] + (1) Inosine_c0[c4] <-> (1) D-Ribose_c0[c4] + (1) HYXN_c0[c4]	0.00630948	Yes
rxn00834	Purine metabolism	IMP:NAD+ oxidoreductase	(1) H2O_c0[c4] + (1) NAD_c0[c4] + (1) IMP_c0[c4] <-> (1) NADH_c0[c4] + (1) H+_c0[c4] + (1) XMP_c0[c4]	0.0566008	Yes
rxn03084	Purine metabolism	5'-Phosphoribosylformylglycinamide:L-glutamine amido-ligase (ADP-forming)	(1) H2O_c0[c4] + (1) ATP_c0[c4] + (1) L-Glutamine_c0[c4] + (1) N-Formyl-GAR_c0[c4] -> (1) ADP_c0[c4] + (1) Phosphate_c0[c4] + (1) L-Glutamate_c0[c4] + (1) H+_c0[c4] + (1) 5'-Phosphoribosylformylglycinamidine_c0[c4]	0.111845	Yes
rxn01225	Purine metabolism	Guanine aminohydrolase	(1) H2O_c0[c4] + (1) H+_c0[c4] + (1) Guanine_c0[c4] <-> (1) NH3_c0[c4] + (1) XAN_c0[c4]	-0.0580712	Yes
rxn00239	Purine metabolism	ATP:GMP phosphotransferase	(1) ATP_c0[c4] + (1) H+_c0[c4] + (1) GMP_c0[c4] <-> (1) ADP_c0[c4] + (1) GDP_c0[c4]	0.0591228	Yes
rxn01137	Purine metabolism	Adenosine aminohydrolase	(1) H2O_c0[c4] + (1) H+_c0[c4] + (1) Adenosine_c0[c4] -> (1) NH3_c0[c4] + (1) Inosine_c0[c4]	0.00630948	Yes
rxn05234	Purine metabolism	2'-Deoxyguanosine 5'-triphosphate:oxydized-thioredoxin 2'-oxidoreductase	(1) GTP_c0[c4] + (1) trdrd_c0[c4] -> (1) H2O_c0[c4] + (1) dGTP_c0[c4] + (1) trdox_c0[c4]	0.00682829	No
rxn05232	Purine metabolism	2'-Deoxyadenosine 5'-triphosphate:oxydized-thioredoxin 2'-oxidoreductase	(1) ATP_c0[c4] + (1) trdrd_c0[c4] -> (1) H2O_c0[c4] + (1) dATP_c0[c4] + (1) trdox_c0[c4]	0.00405171	No
rxn00800	Purine metabolism|Alanine, aspartate and glutamate metabolism	N6-(1,2-dicarboxyethyl)AMP AMP-lyase (fumarate-forming)	(1) Adenylosuccinate_c0[c4] <-> (1) AMP_c0[c4] + (1) Fumarate_c0[c4]	0.0810054	Yes

rxn00790	Purine metabolism|Alanine, aspartate and glutamate metabolism	5-phosphoribosylamine:diphosphate phospho-alpha-D-ribosyltransferase (glutamate-amidating)	(1) PPi_c0[c4] + (1) L-Glutamate_c0[c4] + (1) H+_c0[c4] + (1) 5-Phosphoribosylamine_c0[c4] <- (1) H2O_c0[c4] + (1) L-Glutamine_c0[c4] + (1) PRPP_c0[c4]	-0.111845	Yes
rxn00838	Purine metabolism|Alanine, aspartate and glutamate metabolism	IMP:L-aspartate ligase (GDP-forming)	(1) GTP_c0[c4] + (1) L-Aspartate_c0[c4] + (1) IMP_c0[c4] -> (1) Phosphate_c0[c4] + (1) GDP_c0[c4] + (2) H+_c0[c4] + (1) Adenylosuccinate_c0[c4]	0.0810054	Yes
rxn00114	Purine metabolism|Arginine and proline metabolism|Nitrogen metabolism	ATP:carbamate phosphotransferase	(1) ATP_c0[c4] + (1) CO2_c0[c4] + (1) NH3_c0[c4] <-> (1) ADP_c0[c4] + (2) H+_c0[c4] + (1) Carbamoylphosphate_c0[c4]	0.159836	Yes
rxn03137	Purine metabolism|One carbon pool by folate	10-Formyltetrahydrofolate:5'-phosphoribosyl-5-amino-4-imidazolecarboxamide formyltransferase	(1) 10-Formyltetrahydrofolate_c0[c4] + (1) AICAR_c0[c4] <-> (1) Tetrahydrofolate_c0[c4] + (1) FAICAR_c0[c4]	0.137606	Yes
rxn03004	Purine metabolism|One carbon pool by folate	10-Formyltetrahydrofolate:5'-phosphoribosylglycinamide formyltransferase	(1) 10-Formyltetrahydrofolate_c0[c4] + (1) GAR_c0[c4] <-> (1) H+_c0[c4] + (1) Tetrahydrofolate_c0[c4] + (1) N-Formyl-GAR_c0[c4]	0.111845	No
rxn00379	Purine metabolism|Sulfur metabolism	ATP:sulfate adenylyltransferase	(1) ATP_c0[c4] + (1) Sulfate_c0[c4] <-> (1) PPi_c0[c4] + (1) APS_c0[c4]	0.215805	Yes
rxn00364	Pyrimidine metabolism	ATP:CMP phosphotransferase	(1) ATP_c0[c4] + (1) CMP_c0[c4] + (1) H+_c0[c4] <-> (1) ADP_c0[c4] + (1) CDP_c0[c4]	0.187544	Yes
rxn00710	Pyrimidine metabolism	orotidine-5'-phosphate carboxy-lyase (UMP-forming)	(1) H+_c0[c4] + (1) Orotidylic acid_c0[c4] -> (1) CO2_c0[c4] + (1) UMP_c0[c4]	0.0702892	Yes
rxn01362	Pyrimidine metabolism	Orotidine-5'-phosphate:diphosphate phospho-alpha-D-ribosyl-transferase	(1) PPi_c0[c4] + (1) H+_c0[c4] + (1) Orotidylic acid_c0[c4] <- (1) PRPP_c0[c4] + (1) Orotate_c0[c4]	-0.0702892	Yes
rxn01465	Pyrimidine metabolism	(S)-dihydroorotate amidohydrolase	(1) H2O_c0[c4] + (1) S-Dihydroorotate_c0[c4] <-> (1) H+_c0[c4] + (1) N-Carbamoyl-L-aspartate_c0[c4]	-0.0702892	Yes
rxn01678	Pyrimidine metabolism	ATP:dUDP phosphotransferase	(1) ATP_c0[c4] + (1) dUDP_c0[c4] <-> (1) ADP_c0[c4] + (1) dUTP_c0[c4]	0.00405171	Yes
rxn00412	Pyrimidine metabolism	UTP:L-glutamine amido-ligase (ADP-forming)	(1) H2O_c0[c4] + (1) ATP_c0[c4] + (1) L-Glutamine_c0[c4] + (1) UTP_c0[c4] -> (1) ADP_c0[c4] + (1) Phosphate_c0[c4] + (1) L-Glutamate_c0[c4] + (1) CTP_c0[c4] + (2) H+_c0[c4]	0.0353904	Yes
rxn01512	Pyrimidine metabolism	ATP:dTDP phosphotransferase	(1) ATP_c0[c4] + (1) dTDP_c0[c4] <-> (1) ADP_c0[c4] + (1) TTP_c0[c4]	0.0125455	Yes
rxn00409	Pyrimidine metabolism	ATP:CDP phosphotransferase	(1) ATP_c0[c4] + (1) CDP_c0[c4] <-> (1) ADP_c0[c4] + (1) CTP_c0[c4]	0.187544	Yes
rxn01519	Pyrimidine metabolism	dUTP nucleotidohydrolase	(1) H2O_c0[c4] + (1) dUTP_c0[c4] -> (1) PPi_c0[c4] + (2) H+_c0[c4] + (1) dUMP_c0[c4]	0.00405171	Yes
rxn01513	Pyrimidine metabolism	ATP:dTMP phosphotransferase	(1) ATP_c0[c4] + (1) H+_c0[c4] + (1) dTMP_c0[c4] <-> (1) ADP_c0[c4] + (1) dTDP_c0[c4]	0.00405171	Yes
rxn06075	Pyrimidine metabolism	2'-Deoxyuridine 5'-diphosphate:oxidized-thioredoxin 2'-oxidoreductase	(1) H2O_c0[c4] + (1) dUDP_c0[c4] + (1) trdox_c0[c4] <- (1) UDP_c0[c4] + (1) trdrd_c0[c4]	-0.00405171	Yes

rxn05289	Pyrimidine metabolism	NADPH:oxidized-thioredoxin oxidoreductase	(1) NADPH_c0[c4] + (1) H+_c0[c4] + (1) trdox_c0[c4] <-> (1) NADP_c0[c4] + (1) trdrd_c0[c4]	0.096385	Yes
rxn00117	Pyrimidine metabolism	ATP:UDP phosphotransferase	(1) ATP_c0[c4] + (1) UDP_c0[c4] <-> (1) ADP_c0[c4] + (1) UTP_c0[c4]	0.0469108	Yes
rxn05235	Pyrimidine metabolism	2'-Deoxyuridine 5'-triphosphate:oxydized-thioredoxin 2'-oxidoreductase	(1) CTP_c0[c4] + (1) trdrd_c0[c4] -> (1) H2O_c0[c4] + (1) dCTP_c0[c4] + (1) trdox_c0[c4]	0.00682829	No
rxn01018	Pyrimidine metabolism|Alanine, aspartate and glutamate metabolism	carbamoyl-phosphate:L-aspartate carbamoyltransferase	(1) L-Aspartate_c0[c4] + (1) Carbamoylphosphate_c0[c4] -> (1) Phosphate_c0[c4] + (1) H+_c0[c4] + (1) N-Carbamoyl-L-aspartate_c0[c4]	0.0702892	Yes
rxn01520	Pyrimidine metabolism|One carbon pool by folate	5,10-Methylenetetrahydrofolate: dUMP C-methyltransferase	(1) 5-10-Methylenetetrahydrofolate_c0[c4] + (1) dUMP_c0[c4] -> (1) dTMP_c0[c4] + (1) Dihydrofolate_c0[c4]	0.00405171	No
rxn00146	Pyruvate metabolism	(R)-Lactate:ferricytochrome-c 2-oxidoreductase	(2) Cytochrome c3+_c0[c4] + (1) D-Lactate_c0[c4] <-> (1) Pyruvate_c0[c4] + (2) H+_c0[c4] + (2) Cytochrome c2+_c0[c4]	-1.92032	Yes
rxn00500	Pyruvate metabolism	(R)-Lactate:NAD+ oxidoreductase	(1) NAD_c0[c4] + (1) D-Lactate_c0[c4] <-> (1) NADH_c0[c4] + (1) Pyruvate_c0[c4] + (1) H+_c0[c4]	1.92032	Yes
rxn00251	Pyruvate metabolism|Methane metabolism|Carbon fixation in photosynthetic organisms|Carbon fixation pathways in prokaryotes	phosphate:oxaloacetate carboxy-lyase (adding phosphate;phosphoenolpyruvate-forming)	(1) Phosphate_c0[c4] + (1) Oxaloacetate_c0[c4] + (1) H+_c0[c4] <-> (1) H2O_c0[c4] + (1) CO2_c0[c4] + (1) Phosphoenolpyruvate_c0[c4]	-0.932139	Yes
rxn05040	Riboflavin metabolism	D-ribulose 5-phosphate formate-lyase (L-3,4-dihydroxybutan-2-one 4-phosphate-forming)	(1) D-Ribulose5-phosphate_c0[c4] -> (1) Formate_c0[c4] + (1) H+_c0[c4] + (1) 3-4-dihydroxy-2-butanone4-phosphate_c0[c4]	0.00420632	Yes
rxn00300	Riboflavin metabolism	GTP 7,8-8,9-dihydrolase (diphosphate-forming)	(3) H2O_c0[c4] + (1) GTP_c0[c4] -> (1) PPi_c0[c4] + (1) Formate_c0[c4] + (3) H+_c0[c4] + (1) 2,5-Diamino-6-(5'-phosphoribosylamino)-4-pyrimidineone_c0[c4]	0.00210316	Yes
rxn00392	Riboflavin metabolism	ATP:riboflavin 5'-phosphotransferase	(1) ATP_c0[c4] + (1) Riboflavin_c0[c4] <-> (1) ADP_c0[c4] + (1) FMN_c0[c4] + (1) H+_c0[c4]	0.00105158	Yes
rxn03080	Riboflavin metabolism	5-amino-6-(D-ribitylamino)uracil butanedionetransferase	(1) 4--1-D-Ribitylamino-5-aminouracil_c0[c4] + (1) 3-4-dihydroxy-2-butanone4-phosphate_c0[c4] <-> (2) H2O_c0[c4] + (1) Phosphate_c0[c4] + (1) H+_c0[c4] + (1) 6-7-Dimethyl-8--1-D-ribityllumazine_c0[c4]	0.00420632	Yes
rxn00048	Riboflavin metabolism	6,7-Dimethyl-8-(1-D-ribityl)lumazine:6,7-dimethyl-8-(1-D-ribityl)lumazine 2,3-butanediyltransferase	(1) H+_c0[c4] + (2) 6-7-Dimethyl-8--1-D-ribityllumazine_c0[c4] -> (1) Riboflavin_c0[c4] + (1) 4--1-D-Ribitylamino-5-aminouracil_c0[c4]	0.00210316	Yes
rxn02475	Riboflavin metabolism	2,5-Diamino-6-hydroxy-4-(5-phosphoribosylamino)-pyrimidine 2-aminohydrolase	(1) H2O_c0[c4] + (1) H+_c0[c4] + (1) 2,5-Diamino-6-(5'-phosphoribosylamino)-4-pyrimidineone_c0[c4] -> (1) NH3_c0[c4] + (1) 5-Amino-6--5-phosphoribosylaminouracil_c0[c4]	0.00210316	Yes
rxn00122	Riboflavin metabolism	ATP:FMN adenylyltransferase	(1) ATP_c0[c4] + (1) FMN_c0[c4] -> (1) PPi_c0[c4] + (1) FAD_c0[c4]	0.00105158	Yes

rxn02474	Riboflavin metabolism	5-amino-6-(5-phosphoribitylamino)uracil: NADP+ 1'-oxidoreductase	(1) NADP_c0[c4] + (1) 5-Amino-6--5-phosphoribitylaminouracil_c0[c4] <-> (1) NADPH_c0[c4] + (1) H+_c0[c4] + (1) 5-Amino-6--5-phosphoribosylaminouracil_c0[c4]	-0.00210316	Yes
rxn05039	Riboflavin metabolism	R07280	(1) H2O_c0[c4] + (1) 5-Amino-6--5-phosphoribitylaminouracil_c0[c4] -> (1) Phosphate_c0[c4] + (1) 4--1-D-Ribitylamino-5-aminouracil_c0[c4]	0.00210316	No
rxn00606	Starch and sucrose metabolism	alpha,alpha-Trehalose-6-phosphate phosphoglucohydrolase	(1) H2O_c0[c4] + (1) Trehalose 6-phosphate_c0[c4] <-> (1) D-Glucose_c0[c4] + (1) D-glucose-6-phosphate_c0[c4]	-0.0106172	No
rxn00216	Streptomycin biosynthesis|Butirosin and neomycin biosynthesis	ATP:D-glucose 6-phosphotransferase	(1) ATP_c0[c4] + (1) D-Glucose_c0[c4] <-> (1) ADP_c0[c4] + (1) H+_c0[c4] + (1) D-glucose-6-phosphate_c0[c4]	-0.0106172	Yes
rxn02000	Streptomycin biosynthesis|Polyketide sugar unit biosynthesis	dTDP-4-dehydro-6-deoxy-D-glucose 3,5-epimerase	(1) dTDP-4-oxo-6-deoxy-D-glucose_c0[c4] <-> (1) dTDP-4-oxo-L-rhamnose_c0[c4]	0.00849375	Yes
rxn01675	Streptomycin biosynthesis|Polyketide sugar unit biosynthesis	dTTP:alpha-D-glucose-1-phosphate thymidylyltransferase	(1) Glucose-1-phosphate_c0[c4] + (1) TTP_c0[c4] <-> (1) PPi_c0[c4] + (1) dTDPglucose_c0[c4]	0.00849375	Yes
rxn02003	Streptomycin biosynthesis|Polyketide sugar unit biosynthesis	dTDP-6-deoxy-L-mannose:NADP+ 4-oxidoreductase	(1) NADP_c0[c4] + (1) dTDP-rhamnose_c0[c4] <-> (1) NADPH_c0[c4] + (1) H+_c0[c4] + (1) dTDP-4-oxo-L-rhamnose_c0[c4]	-0.00849375	Yes
rxn01997	Streptomycin biosynthesis|Polyketide sugar unit biosynthesis|Biosynthesis of vancomycin group antibiotics	dTDPglucose 4,6-hydro-lyase	(1) dTDPglucose_c0[c4] -> (1) H2O_c0[c4] + (1) dTDP-4-oxo-6-deoxy-D-glucose_c0[c4]	0.00849375	Yes
rxn00623	Sulfur metabolism	hydrogen-sulfide:NADP+ oxidoreductase	(3) H2O_c0[c4] + (3) NADP_c0[c4] + (1) H2S_c0[c4] <-> (3) NADPH_c0[c4] + (3) H+_c0[c4] + (1) Sulfite_c0[c4]	-0.074625	Yes
rxn00991	Synthesis and degradation of ketone bodies|Valine, leucine and isoleucine degradation|Butanoate metabolism	(S)-3-hydroxy-3-methylglutaryl-CoA acetoacetate-lyase (acetyl-CoA-forming)	(1) HMG-CoA_c0[c4] <-> (1) Acetyl-CoA_c0[c4] + (1) Acetoacetate_c0[c4]	-0.181616	Yes
rxn00290	Synthesis and degradation of ketone bodies|Valine, leucine and isoleucine degradation|Butanoate metabolism	succinyl-CoA:acetoacetate CoA-transferase	(1) Succinyl-CoA_c0[c4] + (1) Acetoacetate_c0[c4] <-> (1) Succinate_c0[c4] + (1) Acetoacetyl-CoA_c0[c4]	-0.181616	No
rxn03910	Terpenoid backbone biosynthesis	2-Phospho-4-(cytidine 5'-diphospho)-2-C-methyl-D-erythritol CMP-lyase (cyclizing)	(1) 2-phospho-4--cytidine5-diphospho-2-C-methyl-D-erythritol_c0[c4] <-> (1) CMP_c0[c4] + (1) 2-C-methyl-D-erythritol2-4-cyclodiphosphate_c0[c4]	0.118669	Yes
rxn03908	Terpenoid backbone biosynthesis	ATP:4-(Cytidine 5'-diphospho)-2-C-methyl-D-erythritol 2-phosphotransferase	(1) ATP_c0[c4] + (1) 4--cytidine5-diphospho-2-C-methyl-D-erythritol_c0[c4] <-> (1) ADP_c0[c4] + (1) H+_c0[c4] + (1) 2-phospho-4--cytidine5-diphospho-2-C-methyl-D-erythritol_c0[c4]	0.118669	Yes
rxn08352	Terpenoid backbone biosynthesis	R08210	(1) NADH_c0[c4] + (1) H+_c0[c4] + (1) 1-Hydroxy-2-methyl-2-butenyl 4-diphosphate_c0[c4] -> (1) H2O_c0[c4] + (1) NAD_c0[c4] + (1) DMAPP_c0[c4]	0.0116485	Yes
rxn08756	Terpenoid backbone biosynthesis	isopentenyl-diphosphate:NAD+ oxidoreductase	(1) NADH_c0[c4] + (1) H+_c0[c4] + (1) 1-Hydroxy-2-methyl-2-butenyl 4-diphosphate_c0[c4] -> (1) H2O_c0[c4]	0.107021	Yes

			+ (1) NAD_c0[c4] + (1) Isopentenyldiphosphate_c0[c4]		
rxn01213	Terpenoid backbone biosynthesis	GPPSYN-RXN	(1) Isopentenyldiphosphate_c0[c4] + (1) DMAPP_c0[c4] -> (1) PPi_c0[c4] + (1) H+_c0[c4] + (1) Geranyldiphosphate_c0[c4]	0.0116485	Yes
rxn03909	Terpenoid backbone biosynthesis	1-Deoxy-D-xylulose-5-phosphate pyruvate-lyase (carboxylating)	(1) Pyruvate_c0[c4] + (1) H+_c0[c4] + (1) Glyceraldehyde3-phosphate_c0[c4] -> (1) CO2_c0[c4] + (1) 1-deoxy-D-xylulose5-phosphate_c0[c4]	0.119826	Yes
rxn01466	Terpenoid backbone biosynthesis	Geranyl-diphosphate:isopentenyl-diphosphate geranyltrans-transferase	(1) Isopentenyldiphosphate_c0[c4] + (1) Geranyldiphosphate_c0[c4] -> (1) PPi_c0[c4] + (1) H+_c0[c4] + (1) Farnesyldiphosphate_c0[c4]	0.0116485	Yes
rxn03958	Terpenoid backbone biosynthesis	1-Deoxy-D-xylulose-5-phosphate isomeroreductase	(1) NADP_c0[c4] + (1) 2-C-methyl-D-erythritol4-phosphate_c0[c4] <-> (1) NADPH_c0[c4] + (1) H+_c0[c4] + (1) 1-deoxy-D-xylulose5-phosphate_c0[c4]	-0.118669	No
rxn03907	Terpenoid backbone biosynthesis	CTP: 2-C-Methyl-D-erythritol 4-phosphate cytidylyltransferase	(1) CTP_c0[c4] + (1) 2-C-methyl-D-erythritol4-phosphate_c0[c4] <-> (1) PPi_c0[c4] + (1) 4--cytidine5-diphospho-2-C-methyl-D-erythritol_c0[c4]	0.118669	No
rxn13477	Terpenoid backbone biosynthesis	(2E,6E)-farnesyl-diphosphate:isopentenyl-diphosphate farnesyltranstransferase (adding 3 isopentenyl units)	(3) Isopentenyldiphosphate_c0[c4] + (1) Farnesyldiphosphate_c0[c4] -> (3) PPi_c0[c4] + (3) H+_c0[c4] + (1) all-trans-Hexaprenyl diphosphate[c4]	0.00315474	No
rxn00533	Tetracycline biosynthesis|Pyruvate metabolism|Propanoate metabolism|Carbon fixation pathways in prokaryotes	Acetyl-CoA:carbon-dioxide ligase (ADP-forming)	(1) ATP_c0[c4] + (1) Acetyl-CoA_c0[c4] + (1) H2CO3_c0[c4] <-> (1) ADP_c0[c4] + (1) Phosphate_c0[c4] + (1) H+_c0[c4] + (1) Malonyl-CoA_c0[c4]	0.875864	Yes
rxn02305	Thiamine metabolism	2-methyl-4-amino-5-hydroxymethylpyrimidine-diphosphate:4-methyl-5-(2-phosphoethyl)-thiazole 2-methyl-4-aminopyridine-5-methenyltransferase	(1) 4-Methyl-5--2-phosphoethyl-thiazole_c0[c4] + (1) 4-Amino-2-methyl-5-diphosphomethylpyrimidine_c0[c4] <-> (1) PPi_c0[c4] + (1) Thiamine phosphate_c0[c4]	0.00115674	Yes
rxn02484	Thiamine metabolism	ATP:4-amino-5-hydroxymethyl-2-methylpyrimidine 5-phosphotransferase	(1) ATP_c0[c4] + (1) Toxopyrimidine_c0[c4] <-> (1) ADP_c0[c4] + (1) H+_c0[c4] + (1) 4-Amino-5-phosphomethyl-2-methylpyrimidine_c0[c4]	0.00115674	No
rxn03108	Thiamine metabolism	ATP:4-amino-2-methyl-5-phosphomethylpyrimidine phosphotransferase	(1) ATP_c0[c4] + (1) H+_c0[c4] + (1) 4-Amino-5-phosphomethyl-2-methylpyrimidine_c0[c4] <-> (1) ADP_c0[c4] + (1) 4-Amino-2-methyl-5-diphosphomethylpyrimidine_c0[c4]	0.00115674	No
rxn00438	Thiamine metabolism	ATP:thiamin-phosphate phosphotransferase	(1) ATP_c0[c4] + (1) H+_c0[c4] + (1) Thiamine phosphate_c0[c4] <-> (1) ADP_c0[c4] + (1) TPP_c0[c4]	0.00105158	No
rxn01538	Thiamine metabolism	ATP:thiamine phosphotransferase	(1) ATP_c0[c4] + (1) Thiamin_c0[c4] <- (1) ADP_c0[c4] + (1) H+_c0[c4] + (1) Thiamine phosphate_c0[c4]	-0.000105158	No
rxn11946	Ubiquinone and other terpenoid-quinone biosynthesis	R05614	(1) S-Adenosyl-L-methionine_c0[c4] + (1) 2-Octaprenyl-3-methyl-5-hydroxy-6-methoxy-1,4-benzoquinone_c0[c4] <-> (1) S-Adenosyl-homocysteine_c0[c4] + (1) H+_c0[c4] + (1) Ubiquinone-8_c0[c4]	0.00105158	Yes

rxn04139	Ubiquinone and other terpenoid-quinone biosynthesis	2-Octaprenyl-3-methyl-6-methoxy-1,4-benzoquinone ,NADPH2:oxygen oxidoreductase	(1) NADPH_c0[c4] + (1) O2_c0[c4] + (1) H+_c0[c4] + (1) 2-Octaprenyl-3-methyl-6-methoxy-1,4-benzoquinone_c0[c4] -> (1) H2O_c0[c4] + (1) NADP_c0[c4] + (1) 2-Octaprenyl-3-methyl-5-hydroxy-6-methoxy-1,4-benzoquinone_c0[c4]	0.00105158	Yes
rxn03893	Ubiquinone and other terpenoid-quinone biosynthesis	all-trans-octaprenyl-diphosphate:4-hydroxybenzoate 3-octaprenyltransferase	(1) 4-Hydroxybenzoate_c0[c4] + (1) Farnesylfarnesylgeraniol_c0[c4] -> (1) PPi_c0[c4] + (1) H+_c0[c4] + (1) 3-Octaprenyl-4-hydroxybenzoate_c0[c4]	0.00105158	Yes
rxn03393	Ubiquinone and other terpenoid-quinone biosynthesis	3-octaprenyl-4-hydroxybenzoate carboxy-lyase	(1) H+_c0[c4] + (1) 3-Octaprenyl-4-hydroxybenzoate_c0[c4] -> (1) CO2_c0[c4] + (1) 2-Octaprenylphenol_c0[c4]	0.00105158	Yes
rxn03394	Ubiquinone and other terpenoid-quinone biosynthesis	R04987	(1) NADPH_c0[c4] + (1) O2_c0[c4] + (1) H+_c0[c4] + (1) 2-Octaprenylphenol_c0[c4] -> (1) H2O_c0[c4] + (1) NADP_c0[c4] + (1) 2-Octaprenyl-6-hydroxyphenol_c0[c4]	0.00105158	Yes
rxn03395	Ubiquinone and other terpenoid-quinone biosynthesis	S-adenosyl-L-methionine:3-(all-trans-octaprenyl)benzene-1,2-diol 2-O-methyltransferase	(1) S-Adenosyl-L-methionine_c0[c4] + (1) 2-Octaprenyl-6-hydroxyphenol_c0[c4] <-> (1) S-Adenosyl-homocysteine_c0[c4] + (1) H+_c0[c4] + (1) 2-Octaprenyl-6-methoxyphenol_c0[c4]	0.00105158	Yes
rxn00966	Ubiquinone and other terpenoid-quinone biosynthesis	chorismate pyruvate-lyase (4-hydroxybenzoate-forming)	(1) Pyruvate_c0[c4] + (1) 4-Hydroxybenzoate_c0[c4] <- (1) Chorismate_c0[c4]	-0.00105158	No
rxn02832	Ubiquinone and other terpenoid-quinone biosynthesis	(1R,6R)-6-hydroxy-2-succinylcyclohexa-2,4-diene-1-carboxylate hydrolyase (2-succinylbenzoate-forming)	(1) H2O_c0[c4] + (1) Succinylbenzoate[c4] <- (1) SHCHC[c4]	-0.00210316	No
rxn03397	Ubiquinone and other terpenoid-quinone biosynthesis	UDP-L-rhamnose:flavonol-3-O-D-glucoside L-rhamnosyltransferase	(1) S-Adenosyl-L-methionine_c0[c4] + (1) 2-Octaprenyl-6-methoxy-1,4-benzoquinone_c0[c4] -> (1) S-Adenosyl-homocysteine_c0[c4] + (1) H+_c0[c4] + (1) 2-Octaprenyl-3-methyl-6-methoxy-1,4-benzoquinone_c0[c4]	0.00105158	No
rxn02831	Ubiquinone and other terpenoid-quinone biosynthesis	O-Succinylbenzoate:CoA ligase (AMP-forming)	(1) ATP_c0[c4] + (1) CoA_c0[c4] + (1) Succinylbenzoate[c4] -> (1) PPi_c0[c4] + (1) AMP_c0[c4] + (1) H+_c0[c4] + (1) Succinylbenzoyl-CoA[c4]	0.00210316	No
rxn01258	Ubiquinone and other terpenoid-quinone biosynthesis|Biosynthesis of siderophore group nonribosomal peptides	chorismate hydroxymutase	(1) Chorismate_c0[c4] -> (1) Isochorismate_c0[c4]	0.00210316	No
rxn00527	Ubiquinone and other terpenoid-quinone biosynthesis|Tyrosine metabolism|Phenylalanine, tyrosine and tryptophan biosynthesis|Novobiocin biosynthesis|Isoquinoline alkaloid biosynthesis	L-tyrosine:2-oxoglutarate aminotransferase	(1) 2-Oxoglutarate_c0[c4] + (1) L-Tyrosine_c0[c4] <-> (1) L-Glutamate_c0[c4] + (1) p-hydroxyphenylpyruvate_c0[c4]	-0.0525426	Yes
rxn03436	Valine, leucine and isoleucine biosynthesis	(S)-2-Aceto-2-hydroxybutanoate:NADP+ oxidoreductase (isomerizing)	(1) 2-Aceto-2-hydroxybutanoate_c0[c4] <-> (1) (R)-3-Hydroxy-3-methyl-2-oxopentanoate_c0[c4]	0.111045	Yes

rxn03435	Valine, leucine and isoleucine biosynthesis	(R)-2,3-Dihydroxy-3-methylpentanoate:NADP+ oxidoreductase (isomerizing)	(1) NADP_c0[c4] + (1) 2,3-Dihydroxy-3-methylvalerate_c0[c4] <-> (1) NADPH_c0[c4] + (1) H+_c0[c4] + (1) (R)-3-Hydroxy-3-methyl-2-oxopentanoate_c0[c4]	-0.111045	Yes
rxn01045	Valine, leucine and isoleucine biosynthesis	L-Valine:NAD+ oxidoreductase(deaminating)	(1) H2O_c0[c4] + (1) NAD_c0[c4] + (1) L-Valine_c0[c4] <-> (1) NADH_c0[c4] + (1) NH3_c0[c4] + (1) H+_c0[c4] + (1) 3-Methyl-2-oxobutanoate_c0[c4]	-0.289274	Yes
rxn01573	Valine, leucine and isoleucine biosynthesis	L-Isoleucine:NAD+ oxidoreductase(deaminating)	(1) H2O_c0[c4] + (1) NAD_c0[c4] + (1) L-Isoleucine_c0[c4] <-> (1) NADH_c0[c4] + (1) NH3_c0[c4] + (1) H+_c0[c4] + (1) 3MOP_c0[c4]	-0.0821163	Yes
rxn03437	Valine, leucine and isoleucine biosynthesis	(R)-2,3-Dihydroxy-3-methylpentanoate hydro-lyase	(1) 2,3-Dihydroxy-3-methylvalerate_c0[c4] -> (1) H2O_c0[c4] + (1) 3MOP_c0[c4]	0.111045	Yes
rxn03194	Valine, leucine and isoleucine biosynthesis	(S)-2-Aceto-2-hydroxybutanoate pyruvate-lyase (carboxylating)	(1) 2-Oxobutyrate_c0[c4] + (1) 2-Hydroxyethyl-ThPP_c0[c4] <-> (1) TPP_c0[c4] + (1) 2-Aceto-2-hydroxybutanoate_c0[c4]	0.111045	Yes
rxn00904	Valine, leucine and isoleucine biosynthesis	L-Valine:pyruvate aminotransferase	(1) Pyruvate_c0[c4] + (1) L-Valine_c0[c4] <-> (1) L-Alanine_c0[c4] + (1) 3-Methyl-2-oxobutanoate_c0[c4]	0.169654	No
rxn07434	Valine, leucine and isoleucine degradation	R07603	(1) TPP_c0[c4] + (1) H+_c0[c4] + (1) 3MOP_c0[c4] -> (1) CO2_c0[c4] + (1) 2-Methyl-1-hydroxybutyl-TPP_c0[c4]	0.0289291	Yes
rxn01504	Valine, leucine and isoleucine degradation	(S)-3-Hydroxy-3-methylglutaryl-CoA hydro-lyase (trans-3-methylglutaconyl-CoA-forming)	(1) HMG-CoA_c0[c4] <-> (1) H2O_c0[c4] + (1) 3-Methylglutaconyl-CoA_c0[c4]	0.181616	Yes
rxn07433	Valine, leucine and isoleucine degradation	R07602	(1) Lipoamide_c0[c4] + (1) 3-Methyl-1-hydroxybutyl-TPP_c0[c4] <-> (1) TPP_c0[c4] + (1) S-(3-Methylbutanoyl)-dihydrolipoamide-E_c0[c4]	-0.152687	Yes
rxn07435	Valine, leucine and isoleucine degradation	R07604	(1) Lipoamide_c0[c4] + (1) 2-Methyl-1-hydroxybutyl-TPP_c0[c4] <-> (1) TPP_c0[c4] + (1) S-(2-Methylbutanoyl)-dihydrolipoamide-E_c0[c4]	0.0289291	Yes
rxn06586	Valine, leucine and isoleucine degradation	3-methylbutanoyl-CoA:enzyme N6-(dihydrolipoyl)lysine S-(3-methylbutanoyl)transferase	(1) Dihydrolipoamide_c0[c4] + (1) Isovaleryl-CoA_c0[c4] <-> (1) CoA_c0[c4] + (1) S-(3-Methylbutanoyl)-dihydrolipoamide-E_c0[c4]	0.152687	Yes
rxn02889	Valine, leucine and isoleucine degradation	3-Methylcrotonoyl-CoA:carbon-dioxide ligase (ADP-forming)	(1) ATP_c0[c4] + (1) H2CO3_c0[c4] + (1) Dimethylacryloyl-CoA_c0[c4] <-> (1) ADP_c0[c4] + (1) Phosphate_c0[c4] + (1) H+_c0[c4] + (1) 3-Methylglutaconyl-CoA_c0[c4]	-0.181616	Yes
rxn07432	Valine, leucine and isoleucine degradation	R07601	(1) TPP_c0[c4] + (1) H+_c0[c4] + (1) 4MOP_c0[c4] <-> (1) CO2_c0[c4] + (1) 3-Methyl-1-hydroxybutyl-TPP_c0[c4]	-0.152687	Yes
rxn06335	Valine, leucine and isoleucine degradation	(S)-2-methylbutanoyl-CoA:enzyme N6-(dihydrolipoyl)lysine S-(2-methylbutanoyl)transferase	(1) Dihydrolipoamide_c0[c4] + (1) 2-Methylbutyryl-CoA_c0[c4] <-> (1) CoA_c0[c4] + (1) S-(2-Methylbutanoyl)-dihydrolipoamide-E_c0[c4]	-0.0289291	Yes

rxn02866	Valine, leucine and isoleucine degradation	3-methylbutanoyl-CoA:(acceptor) 2,3-oxidoreductase	(1) FAD_c0[c4] + (1) H+_c0[c4] + (1) Isovaleryl-CoA_c0[c4] <- (1) FADH2_c0[c4] + (1) Dimethylacryloyl-CoA_c0[c4]	-0.181616	No
rxn00804	Valine, leucine and isoleucine degradation|Valine, leucine and isoleucine biosynthesis	L-leucine:NAD+ oxidoreductase (deaminating)	(1) H2O_c0[c4] + (1) NAD_c0[c4] + (1) L-Leucine_c0[c4] <-> (1) NADH_c0[c4] + (1) NH3_c0[c4] + (1) H+_c0[c4] + (1) 4MOP_c0[c4]	-0.152687	Yes
***Sphingobium* sp. AP49, community in MOPS medium**					
rxn00802	Alanine, aspartate and glutamate metabolism|Arginine and proline metabolism	2-(Nomega-L-arginino)succinate arginine-lyase (fumarate-forming)	(1) L-Argininosuccinate_c0[c2] <-> (1) L-Arginine_c0[c2] + (1) Fumarate_c0[c2]	0.00628348	No
rxn00503	Alanine, aspartate and glutamate metabolism|Arginine and proline metabolism	(S)-1-pyrroline-5-carboxylate:NAD+ oxidoreductase	(2) H2O_c0[c2] + (1) NAD_c0[c2] + (1) 1-Pyrroline-5-carboxylate_c0[c2] <-> (1) NADH_c0[c2] + (1) L-Glutamate_c0[c2] + (1) H+_c0[c2]	-0.0140308	No
rxn01434	Alanine, aspartate and glutamate metabolism|Arginine and proline metabolism	L-Citrulline:L-aspartate ligase (AMP-forming)	(1) ATP_c0[c2] + (1) L-Aspartate_c0[c2] + (1) Citrulline_c0[c2] <-> (1) PPi_c0[c2] + (1) AMP_c0[c2] + (2) H+_c0[c2] + (1) L-Argininosuccinate_c0[c2]	0.00628348	No
rxn00182	Alanine, aspartate and glutamate metabolism|Arginine and proline metabolism|Taurine and hypotaurine metabolism|D-Glutamine and D-glutamate metabolism|Nitrogen metabolism	L-glutamate:NAD+ oxidoreductase (deaminating)	(1) H2O_c0[c2] + (1) NAD_c0[c2] + (1) L-Glutamate_c0[c2] <-> (1) NADH_c0[c2] + (1) NH3_c0[c2] + (1) 2-Oxoglutarate_c0[c2] + (1) H+_c0[c2]	-0.141524	No
rxn00260	Alanine, aspartate and glutamate metabolism|Carbon fixation in photosynthetic organisms	L-Aspartate:2-oxoglutarate aminotransferase	(1) 2-Oxoglutarate_c0[c2] + (1) L-Aspartate_c0[c2] <-> (1) L-Glutamate_c0[c2] + (1) Oxaloacetate_c0[c2]	-0.0509383	Yes
rxn00085	Alanine, aspartate and glutamate metabolism|Nitrogen metabolism	L-Glutamate:NADP+ oxidoreductase (transaminating)	(1) NADP_c0[c2] + (2) L-Glutamate_c0[c2] <-> (1) NADPH_c0[c2] + (1) 2-Oxoglutarate_c0[c2] + (1) L-Glutamine_c0[c2] + (1) H+_c0[c2]	0.0311389	No
rxn00278	Alanine, aspartate and glutamate metabolism|Taurine and hypotaurine metabolism	L-Alanine:NAD+ oxidoreductase (deaminating)	(1) H2O_c0[c2] + (1) NAD_c0[c2] + (1) L-Alanine_c0[c2] <-> (1) NADH_c0[c2] + (1) NH3_c0[c2] + (1) Pyruvate_c0[c2] + (1) H+_c0[c2]	-0.0128896	No
rxn01484	Amino sugar and nucleotide sugar metabolism	N-Acetyl-D-glucosamine-6-phosphate amidohydrolase	(1) H2O_c0[c2] + (1) N-Acetyl-D-glucosamine 6-phosphate_c0[c2] <-> (1) Acetate_c0[c2] + (1) D-Glucosamine phosphate_c0[c2]	0.00254813	No
rxn03638	Amino sugar and nucleotide sugar metabolism	Acetyl-CoA:D-glucosamine-1-phosphate N-acetyltransferase	(1) Acetyl-CoA_c0[c2] + (1) D-Glucosamine1-phosphate_c0[c2] -> (1) CoA_c0[c2] + (1) H+_c0[c2] + (1) N-Acetyl-D-glucosamine1-phosphate_c0[c2]	0.00254813	No
rxn01485	Amino sugar and nucleotide sugar metabolism	D-Glucosamine 1-phosphate 1,6-phosphomutase	(1) D-Glucosamine1-phosphate_c0[c2] <-> (1) D-Glucosamine phosphate_c0[c2]	-0.00254813	No
rxn00293	Amino sugar and nucleotide sugar metabolism	UTP:N-acetyl-alpha-D-glucosamine-1-phosphate uridylyltransferase	(1) UTP_c0[c2] + (1) N-Acetyl-D-glucosamine1-phosphate_c0[c2] <-> (1) PPi_c0[c2] + (1) UDP-N-acetylglucosamine_c0[c2]	0.00254813	No

rxn02285	Amino sugar and nucleotide sugar metabolism|Peptidoglycan biosynthesis	UDP-N-acetylmuramate:NADP+ oxidoreductase	(1) NADP_c0[c2] + (1) UDP-MurNAc_c0[c2] <-> (1) NADPH_c0[c2] + (1) H+_c0[c2] + (1) UDP-N-acetylglucosamine enolpyruvate_c0[c2]	-0.000637032	No
rxn00461	Amino sugar and nucleotide sugar metabolism|Peptidoglycan biosynthesis	Phosphoenolpyruvate:UDP-N-acetyl-D-glucosamine 1-carboxyvinyl-transferase	(1) UDP-N-acetylglucosamine_c0[c2] + (1) Phosphoenolpyruvate_c0[c2] <-> (1) Phosphate_c0[c2] + (1) UDP-N-acetylglucosamine enolpyruvate_c0[c2]	0.000637032	No
rxn01636	Arginine and proline metabolism	N2-Acetyl-L-ornithine:L-glutamate N-acetyltransferase	(1) L-Glutamate_c0[c2] + (1) N-Acetylornithine_c0[c2] <-> (1) Ornithine_c0[c2] + (1) N-Acetyl-L-glutamate_c0[c2]	0.00636235	No
rxn01637	Arginine and proline metabolism	N2-Acetyl-L-ornithine:2-oxoglutarate aminotransferase	(1) 2-Oxoglutarate_c0[c2] + (1) N-Acetylornithine_c0[c2] <-> (1) L-Glutamate_c0[c2] + (1) 2-Acetamido-5-oxopentanoate_c0[c2]	-0.00636235	No
rxn01917	Arginine and proline metabolism	ATP:N-acetyl-L-glutamate 5-phosphotransferase	(1) ATP_c0[c2] + (1) N-Acetyl-L-glutamate_c0[c2] <-> (1) ADP_c0[c2] + (1) n-acetylglutamyl-phosphate_c0[c2]	0.00636235	No
rxn00929	Arginine and proline metabolism	L-Proline:NAD+ 5-oxidoreductase	(1) NAD_c0[c2] + (1) L-Proline_c0[c2] <-> (1) NADH_c0[c2] + (2) H+_c0[c2] + (1) 1-Pyrroline-5-carboxylate_c0[c2]	-0.0140308	No
rxn02465	Arginine and proline metabolism	N-acetyl-L-glutamate-5-semialdehyde:NADP+ 5-oxidoreductase (phosphrylating)	(1) NADP_c0[c2] + (1) Phosphate_c0[c2] + (1) 2-Acetamido-5-oxopentanoate_c0[c2] <- (1) NADPH_c0[c2] + (1) H+_c0[c2] + (1) n-acetylglutamyl-phosphate_c0[c2]	-0.00636235	No
rxn01019	Arginine and proline metabolism	Carbamoyl-phosphate:L-ornithine carbamoyltransferase	(1) Ornithine_c0[c2] + (1) Carbamoylphosphate_c0[c2] -> (1) Phosphate_c0[c2] + (1) H+_c0[c2] + (1) Citrulline_c0[c2]	0.00628348	No
rxn00501	beta-Alanine metabolism|Inositol phosphate metabolism|Propanoate metabolism	3-Oxopropanoate:NAD+ oxidoreductase (decarboxylating, CoA-acetylating)	(1) NAD_c0[c2] + (1) CoA_c0[c2] + (1) 3-Oxopropanoate_c0[c2] <-> (1) NADH_c0[c2] + (1) CO2_c0[c2] + (1) Acetyl-CoA_c0[c2]	-0.000157737	Yes
rxn01791	beta-Alanine metabolism|Pantothenate and CoA biosynthesis	(R)-Pantoate:beta-alanine ligase (AMP-forming)	(1) ATP_c0[c2] + (1) beta-Alanine_c0[c2] + (1) Pantoate_c0[c2] -> (1) PPi_c0[c2] + (1) AMP_c0[c2] + (2) H+_c0[c2] + (1) PAN_c0[c2]	0.000157737	No
rxn02185	Butanoate metabolism	2-Acetolactate pyruvate-lyase (carboxylating)	(1) TPP_c0[c2] + (1) ALCTT_c0[c2] <-> (1) Pyruvate_c0[c2] + (1) 2-Hydroxyethyl-ThPP_c0[c2]	-0.0208603	No
rxn00785	Carbon fixation in photosynthetic organisms	D-Fructose 6-phosphate:D-glyceraldehyde-3-phosphate glycolaldehyde transferase	(1) D-fructose-6-phosphate_c0[c2] + (1) Glyceraldehyde3-phosphate_c0[c2] <-> (1) D-Xylulose5-phosphate_c0[c2] + (1) D-Erythrose4-phosphate_c0[c2]	0.00927167	No
rxn00799	Citrate cycle (TCA cycle)|Carbon fixation pathways in prokaryotes	(S)-malate hydro-lyase (fumarate-forming)	(1) L-Malate_c0[c2] <-> (1) H2O_c0[c2] + (1) Fumarate_c0[c2]	0.018478	No
rxn00285	Citrate cycle (TCA cycle)|Propanoate metabolism|Carbon fixation pathways in prokaryotes	Succinate:CoA ligase (ADP-forming)	(1) ATP_c0[c2] + (1) CoA_c0[c2] + (1) Succinate_c0[c2] <-> (1) ADP_c0[c2] + (1) Phosphate_c0[c2] + (1) Succinyl-CoA_c0[c2]	0.0339258	No
rxn00248	Citrate cycle (TCA cycle)|Pyruvate metabolism|Glyoxylate and dicarboxylate metabolism|Methane metabolism|Carbon fixation in photosynthetic	(S)-malate:NAD+ oxidoreductase	(1) NAD_c0[c2] + (1) L-Malate_c0[c2] <-> (1) NADH_c0[c2] + (1) Oxaloacetate_c0[c2] + (1) H+_c0[c2]	-0.0234687	No

	organisms|Carbon fixation pathways in prokaryotes				
rxn00126	Cysteine and methionine metabolism	ATP:L-methionine S-adenosyltransferase	(1) H2O_c0[c2] + (1) ATP_c0[c2] + (1) L-Methionine_c0[c2] -> (1) Phosphate_c0[c2] + (1) PPi_c0[c2] + (1) S-Adenosyl-L-methionine_c0[c2] + (1) H+_c0[c2]	0.000552079	No
rxn00952	Cysteine and methionine metabolism	O-acetyl-L-homoserine:hydrogen sulfide S-(3-amino-3-carboxypropyl)transferase	(1) H2S_c0[c2] + (1) O-Acetyl-L-homoserine_c0[c2] -> (1) Acetate_c0[c2] + (1) Homocysteine_c0[c2]	0.00333403	No
rxn00141	Cysteine and methionine metabolism	S-Adenosyl-L-homocysteine hydrolase	(1) H2O_c0[c2] + (1) S-Adenosyl-homocysteine_c0[c2] <-> (1) Homocysteine_c0[c2] + (1) Adenosine_c0[c2]	0.000473211	No
rxn00693	Cysteine and methionine metabolism|One carbon pool by folate	5-Methyltetrahydrofolate:L-homocysteine S-methyltransferase	(1) Homocysteine_c0[c2] + (1) 5-Methyltetrahydrofolate_c0[c2] <-> (1) L-Methionine_c0[c2] + (1) Tetrahydrofolate_c0[c2]	0.00380724	No
rxn00423	Cysteine and methionine metabolism|Sulfur metabolism	acetyl-CoA:L-serine O-acetyltransferase	(1) Acetyl-CoA_c0[c2] + (1) L-Serine_c0[c2] -> (1) CoA_c0[c2] + (1) O-Acetyl-L-serine_c0[c2]	0.00225496	No
rxn00649	Cysteine and methionine metabolism|Sulfur metabolism	O3-acetyl-L-serine:hydrogen-sulfide 2-amino-2-carboxyethyltransferase	(1) H2S_c0[c2] + (1) O-Acetyl-L-serine_c0[c2] -> (1) Acetate_c0[c2] + (1) L-Cysteine_c0[c2]	0.00225496	No
rxn01303	Cysteine and methionine metabolism|Sulfur metabolism	Acetyl-CoA:L-homoserine O-acetyltransferase	(1) Acetyl-CoA_c0[c2] + (1) L-Homoserine_c0[c2] -> (1) CoA_c0[c2] + (1) O-Acetyl-L-homoserine_c0[c2]	0.00333403	No
rxn00283	D-Alanine metabolism	alanine racemase	(1) L-Alanine_c0[c2] <-> (1) D-Alanine_c0[c2]	0.00127406	No
rxn00193	D-Glutamine and D-glutamate metabolism	glutamate racemase	(1) L-Glutamate_c0[c2] <-> (1) D-Glutamate_c0[c2]	0.000637032	No
rxn02286	D-Glutamine and D-glutamate metabolism|Peptidoglycan biosynthesis	UDP-N-acetylmuramate:L-alanine ligase (ADP-forming)	(1) ATP_c0[c2] + (1) L-Alanine_c0[c2] + (1) UDP-MurNAc_c0[c2] -> (1) ADP_c0[c2] + (1) Phosphate_c0[c2] + (1) H+_c0[c2] + (1) UDP-N-acetylmuramoyl-L-alanine_c0[c2]	0.000637032	No
rxn02008	D-Glutamine and D-glutamate metabolism|Peptidoglycan biosynthesis	UDP-N-acetylmuramoyl-L-alanine:D-glutamate ligase(ADP-forming)	(1) ATP_c0[c2] + (1) D-Glutamate_c0[c2] + (1) UDP-N-acetylmuramoyl-L-alanine_c0[c2] -> (1) ADP_c0[c2] + (1) Phosphate_c0[c2] + (1) H+_c0[c2] + (1) UDP-N-acetylmuramoyl-L-alanyl-D-glutamate_c0[c2]	0.000637032	No
rxn05345	Fatty acid biosynthesis	dodecanoyl-[acyl-carrier-protein]:malonyl-[acyl-carrier-protein] C-acyltransferase (decarboxylating)	(1) Dodecanoyl-ACP_c0[c2] + (1) Malonyl-acyl-carrierprotein-_c0[c2] -> (1) CO2_c0[c2] + (1) 3-oxotetradecanoyl-acp_c0[c2] + (1) ACP_c0[c2]	0.00254813	No
rxn05465	Fatty acid biosynthesis	Malonyl-CoA:[acyl-carrier-protein] S-malonyltransferase	(1) H+_c0[c2] + (1) Malonyl-CoA_c0[c2] + (1) ACP_c0[c2] <-> (1) CoA_c0[c2] + (1) Malonyl-acyl-carrierprotein-_c0[c2]	0.0253992	No
rxn05342	Fatty acid biosynthesis	(3R)-3-Hydroxytetradecanoyl-[acyl-carrier-protein]:NADP+ oxidoreductase	(1) NADP_c0[c2] + (1) HMA_c0[c2] <-> (1) NADPH_c0[c2] + (1) 3-oxotetradecanoyl-acp_c0[c2]	-0.00254813	No
rxn02504	Folate biosynthesis	2-amino-4-hydroxy-6-(D-erythro-1,2,3-trihydroxypropyl)-7,8-	(1) Dihydroneopterin_c0[c2] <-> (1) Glycolaldehyde_c0[c2] + (1) 6-hydroxymethyl dihydropterin_c0[c2]	0.000236605	No

		dihydropteridine glycolaldehyde-lyase			
rxn03167	Folate biosynthesis	2-Amino-4-hydroxy-6-(erythro-1,2,3-trihydroxypropyl) dihydropteridine triphosphate phosphohydrolase (alkaline optimum)	(3) H2O_c0[c2] + (1) 7,8-Dihydroneopterin 3'-triphosphate_c0[c2] -> (3) Phosphate_c0[c2] + (3) H+_c0[c2] + (1) Dihydroneopterin_c0[c2]	0.000236605	No
rxn02200	Folate biosynthesis	2-amino-4-hydroxy-6-hydroxymethyl-7,8-dihydropteridine:4-aminobenzoate 2-amino-4-hydroxydihydropteridine-6-methenyltransferase	(1) ABEE_c0[c2] + (1) 6-hydroxymethyl dihydropterin_c0[c2] <-> (1) H2O_c0[c2] + (1) Dihydropteroate_c0[c2]	0.000236605	No
rxn03841	Folate biosynthesis	4-amino-4-deoxychorismate pyruvate-lyase	(1) ADC_c0[c2] -> (1) Pyruvate_c0[c2] + (1) H+_c0[c2] + (1) ABEE_c0[c2]	0.000236605	No
rxn01257	Folate biosynthesis	chorismate:L-glutamine aminotransferase	(1) L-Glutamine_c0[c2] + (1) Chorismate_c0[c2] <-> (1) L-Glutamate_c0[c2] + (1) ADC_c0[c2]	0.000236605	No
rxn01603	Folate biosynthesis	7,8-dihydropteroate:L-glutamate ligase (ADP-forming)	(1) ATP_c0[c2] + (1) L-Glutamate_c0[c2] + (1) Dihydropteroate_c0[c2] -> (1) ADP_c0[c2] + (1) Phosphate_c0[c2] + (1) H+_c0[c2] + (1) Dihydrofolate_c0[c2]	0.000236605	No
rxn00650	Glutathione metabolism	L-cysteinylglycine dipeptidase	(1) H2O_c0[c2] + (1) Cys-Gly_c0[c2] <-> (1) Glycine_c0[c2] + (1) L-Cysteine_c0[c2]	-7.88684E-05	No
rxn00350	Glutathione metabolism	glutathione gamma-glutamylaminopeptidase	(1) H2O_c0[c2] + (1) GSH_c0[c2] <-> (1) L-Glutamate_c0[c2] + (1) Cys-Gly_c0[c2]	-7.88684E-05	No
rxn00615	Glycerolipid metabolism	ATP:glycerol 3-phosphotransferase	(1) ATP_c0[c2] + (1) Glycerol_c0[c2] <-> (1) ADP_c0[c2] + (1) H+_c0[c2] + (1) Glycerol-3-phosphate_c0[c2]	0.000813632	No
rxn00611	Glycerophospholipid metabolism	sn-Glycerol-3-phosphate:NAD+ 2-oxidoreductase	(1) NAD_c0[c2] + (1) Glycerol-3-phosphate_c0[c2] <-> (1) NADH_c0[c2] + (1) H+_c0[c2] + (1) Glycerone-phosphate_c0[c2]	-0.00488179	No
rxn06493	Glycine, serine and threonine metabolism	dihydrolipoylprotein:NAD+ oxidoreductase	(1) NAD_c0[c2] + (1) Dihydrolipolprotein_c0[c2] <-> (1) NADH_c0[c2] + (1) H+_c0[c2] + (1) Lipoylprotein_c0[c2]	0.00394185	No
rxn01300	Glycine, serine and threonine metabolism	ATP:L-homoserine O-phosphotransferase	(1) ATP_c0[c2] + (1) L-Homoserine_c0[c2] <-> (1) ADP_c0[c2] + (1) H+_c0[c2] + (1) O-Phospho-L-homoserine_c0[c2]	0.0137045	No
rxn01069	Glycine, serine and threonine metabolism	O-phospho-L-homoserine phosphate-lyase (adding water;L-threonine-forming)	(1) H2O_c0[c2] + (1) O-Phospho-L-homoserine_c0[c2] -> (1) Phosphate_c0[c2] + (1) L-Threonine_c0[c2]	0.0137045	No
rxn06377	Glycine, serine and threonine metabolism	glycine:lipoylprotein oxidoreductase (decarboxylating and acceptor-aminomethylating)	(1) Glycine_c0[c2] + (1) H+_c0[c2] + (1) Lipoylprotein_c0[c2] -> (1) CO2_c0[c2] + (1) S-Aminomethyldihydrolipoylprotein_c0[c2]	0.00394185	No
rxn00692	Glycine, serine and threonine metabolism|Cyanoamino acid metabolism|Glyoxylate and dicarboxylate metabolism|One	5,10-Methylenetetrahydrofolate:glycine hydroxymethyltransferase	(1) H2O_c0[c2] + (1) Glycine_c0[c2] + (1) 5-10-Methylenetetrahydrofolate_c0[c2] <-> (1) L-Serine_c0[c2] + (1) Tetrahydrofolate_c0[c2]	-0.00971732	No

	carbon pool by folate|Methane metabolism				
rxn00337	Glycine, serine and threonine metabolism|Cysteine and methionine metabolism|Lysine biosynthesis	ATP:L-aspartate 4-phosphotransferase	(1) ATP_c0[c2] + (1) L-Aspartate_c0[c2] <-> (1) ADP_c0[c2] + (1) 4-Phospho-L-aspartate_c0[c2]	0.0249458	No
rxn01301	Glycine, serine and threonine metabolism|Cysteine and methionine metabolism|Lysine biosynthesis	L-Homoserine:NAD+ oxidoreductase	(1) NAD_c0[c2] + (1) L-Homoserine_c0[c2] <-> (1) NADH_c0[c2] + (1) H+_c0[c2] + (1) L-Aspartate4-semialdehyde_c0[c2]	-0.0170386	No
rxn01643	Glycine, serine and threonine metabolism|Cysteine and methionine metabolism|Lysine biosynthesis	L-Aspartate-4-semialdehyde:NADP+ oxidoreductase (phosphorylating)	(1) NADP_c0[c2] + (1) Phosphate_c0[c2] + (1) L-Aspartate4-semialdehyde_c0[c2] <- (1) NADPH_c0[c2] + (1) H+_c0[c2] + (1) 4-Phospho-L-aspartate_c0[c2]	-0.0249458	No
rxn00420	Glycine, serine and threonine metabolism|Methane metabolism	O-phospho-L-serine phosphohydrolase	(1) H2O_c0[c2] + (1) phosphoserine_c0[c2] -> (1) Phosphate_c0[c2] + (1) L-Serine_c0[c2]	0.018559	No
rxn01101	Glycine, serine and threonine metabolism|Methane metabolism	3-Phospho-D-glycerate:NAD+ 2-oxidoreductase	(1) NAD_c0[c2] + (1) 3-Phosphoglycerate_c0[c2] <-> (1) NADH_c0[c2] + (1) H+_c0[c2] + (1) 3-Phosphonooxypyruvate_c0[c2]	0.018559	No
rxn02914	Glycine, serine and threonine metabolism|Methane metabolism	3-Phosphoserine:2-oxoglutarate aminotransferase	(1) 2-Oxoglutarate_c0[c2] + (1) phosphoserine_c0[c2] <-> (1) L-Glutamate_c0[c2] + (1) 3-Phosphonooxypyruvate_c0[c2]	-0.018559	No
rxn06600	Glycine, serine and threonine metabolism|One carbon pool by folate	S-aminomethyldihydrolipoylprotein:(6S)-tetrahydrofolate aminomethyltransferase (ammonia-forming)	(1) Tetrahydrofolate_c0[c2] + (1) S-Aminomethyldihydrolipoylprotein_c0[c2] -> (1) NH3_c0[c2] + (1) 5-10-Methylenetetrahydrofolate_c0[c2] + (1) Dihydrolipolprotein_c0[c2]	0.00394185	No
rxn01964	Glycine, serine and threonine metabolism|Phenylalanine, tyrosine and tryptophan biosynthesis	L-serine hydro-lyase [adding 1-C-(indol-3-yl)glycerol 3-phosphate; L-tryptophan and glyceraldehyde-3-phosphate-forming]	(1) L-Serine_c0[c2] + (1) Indoleglycerol phosphate_c0[c2] -> (1) H2O_c0[c2] + (1) L-Tryptophan_c0[c2] + (1) Glyceraldehyde3-phosphate_c0[c2]	0.00120225	No
rxn00599	Glycine, serine and threonine metabolism|Porphyrin and chlorophyll metabolism	succinyl-CoA:glycine C-succinyltransferase (decarboxylating)	(1) Glycine_c0[c2] + (1) H+_c0[c2] + (1) Succinyl-CoA_c0[c2] -> (1) CoA_c0[c2] + (1) CO2_c0[c2] + (1) 5-Aminolevulinate_c0[c2]	0.0012619	No
rxn00737	Glycine, serine and threonine metabolism|Valine, leucine and isoleucine biosynthesis	L-threonine ammonia-lyase (2-oxobutanoate-forming)	(1) L-Threonine_c0[c2] -> (1) NH3_c0[c2] + (1) 2-Oxobutyrate_c0[c2]	0.0083284	No
rxn00781	Glycolysis / Gluconeogenesis|Carbon fixation in photosynthetic organisms	D-glyceraldehyde-3-phosphate:NAD+ oxidoreductase (phosphorylating)	(1) NAD_c0[c2] + (1) Phosphate_c0[c2] + (1) Glyceraldehyde3-phosphate_c0[c2] <-> (1) NADH_c0[c2] + (1) H+_c0[c2] + (1) 1,3-Bisphospho-D-glycerate_c0[c2]	0.119728	No
rxn01100	Glycolysis / Gluconeogenesis|Carbon fixation in photosynthetic organisms	ATP:3-phospho-D-glycerate 1-phosphotransferase	(1) ATP_c0[c2] + (1) 3-Phosphoglycerate_c0[c2] <-> (1) ADP_c0[c2] + (1) 1,3-Bisphospho-D-glycerate_c0[c2]	-0.119728	No
rxn02342	Glycolysis / Gluconeogenesis|Citrate cycle (TCA cycle)|Pyruvate metabolism	R03270	(1) Lipoamide_c0[c2] + (1) 2-Hydroxyethyl-ThPP_c0[c2] <-> (1) TPP_c0[c2] + (1) S-Acetyldihydrolipoamide_c0[c2]	0.0822964	Yes
rxn01871	Glycolysis / Gluconeogenesis|Citrate cycle	acetyl-CoA:enzyme N6-(dihydrolipoyl)lysine S-acetyltransferase	(1) Acetyl-CoA_c0[c2] + (1) Dihydrolipoamide_c0[c2] <-> (1)	-0.0822964	No

	(TCA cycle)|Pyruvate metabolism		CoA_c0[c2] + (1) S-Acetyldihydrolipoamide_c0[c2]		
rxn00011	Glycolysis / Gluconeogenesis|Citrate cycle (TCA cycle)|Valine, leucine and isoleucine biosynthesis|Pyruvate metabolism|Butanoate metabolism	pyruvate:thiamin diphosphate acetaldehydetransferase (decarboxylating)	(1) CO2_c0[c2] + (1) 2-Hydroxyethyl-ThPP_c0[c2] <- (1) Pyruvate_c0[c2] + (1) TPP_c0[c2] + (1) H+_c0[c2]	-0.111485	Yes
rxn00747	Glycolysis / Gluconeogenesis|Fructose and mannose metabolism|Inositol phosphate metabolism|Carbon fixation in photosynthetic organisms	D-glyceraldehyde-3-phosphate aldose-ketose-isomerase	(1) Glyceraldehyde3-phosphate_c0[c2] <-> (1) Glycerone-phosphate_c0[c2]	0.00503953	No
rxn01106	Glycolysis / Gluconeogenesis|Glycine, serine and threonine metabolism|Methane metabolism	2-Phospho-D-glycerate 2,3-phosphomutase	(1) 2-Phospho-D-glycerate_c0[c2] <-> (1) 3-Phosphoglycerate_c0[c2]	-0.101169	No
rxn00459	Glycolysis / Gluconeogenesis|Methane metabolism	2-phospho-D-glycerate hydro-lyase (phosphoenolpyruvate-forming)	(1) 2-Phospho-D-glycerate_c0[c2] <-> (1) H2O_c0[c2] + (1) Phosphoenolpyruvate_c0[c2]	0.101169	No
rxn00175	Glycolysis / Gluconeogenesis|Methane metabolism|Carbon fixation pathways in prokaryotes	Acetate:CoA ligase (AMP-forming)	(1) ATP_c0[c2] + (1) CoA_c0[c2] + (1) Acetate_c0[c2] <-> (1) PPi_c0[c2] + (1) AMP_c0[c2] + (1) Acetyl-CoA_c0[c2] + (1) H+_c0[c2]	0.00941118	No
rxn00148	Glycolysis / Gluconeogenesis|Purine metabolism|Pyruvate metabolism|Carbon fixation in photosynthetic organisms	ATP:pyruvate 2-O-phosphotransferase	(1) ATP_c0[c2] + (1) Pyruvate_c0[c2] <-> (1) ADP_c0[c2] + (1) Phosphoenolpyruvate_c0[c2] + (1) H+_c0[c2]	0.191899	No
rxn00006	Glyoxylate and dicarboxylate metabolism	hydrogen-peroxide:hydrogen-peroxide oxidoreductase	(2) H2O2_c0[c2] -> (2) H2O_c0[c2] + (1) O2_c0[c2]	7.88684E-05	No
rxn00371	Glyoxylate and dicarboxylate metabolism|Methane metabolism	Formate:NAD+ oxidoreductase	(1) NAD_c0[c2] + (1) Formate_c0[c2] -> (1) NADH_c0[c2] + (1) CO2_c0[c2]	0.00134076	No
rxn02320	Histidine metabolism	5-Amino-2-oxopentanoate:2-oxoglutarate aminotransferase	(1) 2-Oxoglutarate_c0[c2] + (1) L-histidinol-phosphate_c0[c2] <-> (1) L-Glutamate_c0[c2] + (1) imidazole acetol-phosphate_c0[c2]	-0.00201888	No
rxn00789	Histidine metabolism	1-(5-phospho-D-ribosyl)-ATP:diphosphate phospho-alpha-D-ribosyl-transferase	(1) PPi_c0[c2] + (1) H+_c0[c2] + (1) Phosphoribosyl-ATP_c0[c2] <- (1) ATP_c0[c2] + (1) PRPP_c0[c2]	-0.00201888	No
rxn02160	Histidine metabolism	L-Histidinol-phosphate phosphohydrolase	(1) H2O_c0[c2] + (1) L-histidinol-phosphate_c0[c2] -> (1) Phosphate_c0[c2] + (1) L-Histidinol_c0[c2]	0.00201888	No
rxn02159	Histidine metabolism	L-Histidinol:NAD+ oxidoreductase	(1) NAD_c0[c2] + (1) L-Histidinol_c0[c2] <-> (1) NADH_c0[c2] + (1) H+_c0[c2] + (1) L-Histidinal_c0[c2]	0.00201888	No
rxn02835	Histidine metabolism	1-(5-phospho-D-ribosyl)-AMP 1,6-hydrolase	(1) H2O_c0[c2] + (1) Phosphoribosyl-AMP_c0[c2] <-> (1) phosphoribosylformiminoaicar-phosphate_c0[c2]	0.00201888	No
rxn03175	Histidine metabolism	N-(5'-Phospho-D-ribosylformimino)-5-amino-1- (5''-phospho-D-ribosyl)-4-	(1) H+_c0[c2] + (1) phosphoribosylformiminoaicar-phosphate_c0[c2] <-> (1)	0.00201888	No

		imidazolecarboxamide ketol-isomerase	phosphoribulosylformimino-AICAR-phosphate_c0[c2]		
rxn03135	Histidine metabolism	R04558	(1) L-Glutamate_c0[c2] + (2) H+_c0[c2] + (1) D-erythro-imidazol-glycerol-phosphate_c0[c2] + (1) AICAR_c0[c2] <- (1) L-Glutamine_c0[c2] + (1) phosphoribulosylformimino-AICAR-phosphate_c0[c2]	-0.00201888	No
rxn00863	Histidine metabolism	L-histidinal:NAD+ oxidoreductase	(1) H2O_c0[c2] + (1) NAD_c0[c2] + (1) L-Histidinal_c0[c2] -> (1) NADH_c0[c2] + (2) H+_c0[c2] + (1) L-Histidine_c0[c2]	0.00201888	No
rxn02834	Histidine metabolism	Phosphoribosyl-ATP pyrophosphohydrolase	(1) H2O_c0[c2] + (1) Phosphoribosyl-ATP_c0[c2] -> (1) PPi_c0[c2] + (2) H+_c0[c2] + (1) Phosphoribosyl-AMP_c0[c2]	0.00201888	No
rxn02473	Histidine metabolism	D-erythro-1-(Imidazol-4-yl)glycerol 3-phosphate hydro-lyase	(1) D-erythro-imidazol-glycerol-phosphate_c0[c2] -> (1) H2O_c0[c2] + (1) imidazole acetol-phosphate_c0[c2]	0.00201888	No
rxn02331	Lipopolysaccharide biosynthesis	phosphoenolpyruvate:D-arabinose-5-phosphate C-(1-carboxyvinyl)transferase (phosphate-hydrolysing, 2-carboxy-2-oxoethyl-forming)	(1) Phosphate_c0[c2] + (1) 3-Deoxy-D-manno-octulosonate8-phosphate_c0[c2] <- (1) H2O_c0[c2] + (1) Phosphoenolpyruvate_c0[c2] + (1) D-Arabinose5-phosphate_c0[c2]	-0.00191109	No
rxn02405	Lipopolysaccharide biosynthesis	CTP:3-deoxy-D-manno-octulosonate cytidylyltransferase	(1) CTP_c0[c2] + (1) KDO_c0[c2] -> (1) PPi_c0[c2] + (1) H+_c0[c2] + (1) CMP-KDO_c0[c2]	0.00191109	No
rxn06865	Lipopolysaccharide biosynthesis	R05146	(1) Lauroyl-KDO2-lipid IV(A)_c0[c2] + (1) ACP_c0[c2] <- (1) kdo2-lipid iva_c0[c2] + (1) Dodecanoyl-ACP_c0[c2]	-0.000637032	No
rxn00313	Lysine biosynthesis	meso-2,6-diaminoheptanedioate carboxy-lyase (L-lysine-forming)	(1) H+_c0[c2] + (1) meso-2,6-Diaminopimelate_c0[c2] -> (1) CO2_c0[c2] + (1) L-Lysine_c0[c2]	0.00727024	No
rxn02011	Lysine biosynthesis|Peptidoglycan biosynthesis	UDP-N-acetylmuramoyl-L-alanyl-D-glutamate:(L)-meso-2,6-diaminoheptanedioate gamma-ligase (ADP-forming)	(1) ATP_c0[c2] + (1) meso-2,6-Diaminopimelate_c0[c2] + (1) UDP-N-acetylmuramoyl-L-alanyl-D-glutamate_c0[c2] -> (1) ADP_c0[c2] + (1) Phosphate_c0[c2] + (1) H+_c0[c2] + (1) UDP-N-acetylmuramoyl-L-alanyl-D-gamma-glutamyl-meso-2-6-diaminopimelate_c0[c2]	0.000637032	No
rxn02988	Nicotinate and nicotinamide metabolism	glycerone phosphate:iminosuccinate alkyltransferase (cyclizing)	(2) H2O_c0[c2] + (1) Phosphate_c0[c2] + (1) Quinolinate_c0[c2] <- (1) Glycerone-phosphate_c0[c2] + (1) Iminoaspartate_c0[c2]	-0.000157737	No
rxn02402	Nicotinate and nicotinamide metabolism	Nicotinate-nucleotide:pyrophosphate phosphoribosyltransferase (carboxylating)	(1) CO2_c0[c2] + (1) PPi_c0[c2] + (1) Nicotinate ribonucleotide_c0[c2] <- (1) H+_c0[c2] + (1) PRPP_c0[c2] + (1) Quinolinate_c0[c2]	-0.000157737	No
rxn02155	Nicotinate and nicotinamide metabolism	ATP:nicotinamide-nucleotide adenylyltransferase	(1) ATP_c0[c2] + (1) Nicotinate ribonucleotide_c0[c2] <-> (1) PPi_c0[c2] + (1) Deamido-NAD_c0[c2]	0.000157737	No
rxn00083	Nicotinate and nicotinamide metabolism	NADPH:NAD+ oxidoreductase	(1) NAD_c0[c2] + (1) NADPH_c0[c2] <-> (1) NADH_c0[c2] + (1) NADP_c0[c2]	-0.0258264	No
rxn00077	Nicotinate and nicotinamide metabolism	ATP:NAD+ 2'-phosphotransferase	(1) ATP_c0[c2] + (1) NAD_c0[c2] <-> (1) NADP_c0[c2] + (1) ADP_c0[c2] + (1) H+_c0[c2]	7.88684E-05	No

rxn00338	Nicotinate and nicotinamide metabolism	L-aspartate:oxygen oxidoreductase	(1) O2_c0[c2] + (1) L-Aspartate_c0[c2] -> (1) H2O2_c0[c2] + (1) H+_c0[c2] + (1) Iminoaspartate_c0[c2]	0.000157737	No
rxn00138	Nicotinate and nicotinamide metabolism	deamido-NAD+:ammonia ligase (AMP-forming)	(1) ATP_c0[c2] + (1) NH3_c0[c2] + (1) Deamido-NAD_c0[c2] -> (1) NAD_c0[c2] + (1) PPi_c0[c2] + (1) AMP_c0[c2] + (2) H+_c0[c2]	0.000157737	No
rxn00102	Nitrogen metabolism	carbonate hydro-lyase (carbon-dioxide-forming)	(1) H+_c0[c2] + (1) H2CO3_c0[c2] <-> (1) H2O_c0[c2] + (1) CO2_c0[c2]	-0.0808177	Yes
rxn00907	One carbon pool by folate|Carbon fixation pathways in prokaryotes	5,10-methylenetetrahydrofolate: NADP+ oxidoreductase	(1) NADP_c0[c2] + (1) 5-10-Methylenetetrahydrofolate_c0[c2] <-> (1) NADPH_c0[c2] + (1) 5-10-Methenyltetrahydrofolate_c0[c2]	0.00932204	Yes
rxn01211	One carbon pool by folate|Carbon fixation pathways in prokaryotes	5,10-Methenyltetrahydrofolate 5-hydrolase (decyclizing)	(1) H2O_c0[c2] + (1) 5-10-Methenyltetrahydrofolate_c0[c2] <-> (1) H+_c0[c2] + (1) 10-Formyltetrahydrofolate_c0[c2]	0.00932204	Yes
rxn00686	One carbon pool by folate|Folate biosynthesis	5,6,7,8-tetrahydrofolate:NADP+ oxidoreductase	(1) NADP_c0[c2] + (1) Tetrahydrofolate_c0[c2] <-> (1) NADPH_c0[c2] + (1) H+_c0[c2] + (1) Dihydrofolate_c0[c2]	-0.00052988	No
rxn04954	One carbon pool by folate|Methane metabolism|Carbon fixation pathways in prokaryotes	5-methyltetrahydrofolate:NAD+ oxidoreductase	(1) NAD_c0[c2] + (1) 5-Methyltetrahydrofolate_c0[c2] <-> (1) NADH_c0[c2] + (1) H+_c0[c2] + (1) 5-10-Methylenetetrahydrofolate_c0[c2]	-0.00388611	No
rxn00898	Pantothenate and CoA biosynthesis	2,3-Dihydroxy-3-methylbutanoate hydro-lyase	(1) 2,3-Dihydroxy-isovalerate_c0[c2] -> (1) H2O_c0[c2] + (1) 3-Methyl-2-oxobutanoate_c0[c2]	0.0208603	No
rxn12512	Pantothenate and CoA biosynthesis	(R)-4'-Phosphopantothenate:L-cysteine ligase	(1) ATP_c0[c2] + (1) L-Cysteine_c0[c2] + (1) 4-phosphopantothenate_c0[c2] -> (1) PPi_c0[c2] + (1) AMP_c0[c2] + (2) H+_c0[c2] + (1) (R)-4'-Phosphopantothenoyl-L-cysteine_c0[c2]	0.000157737	No
rxn00100	Pantothenate and CoA biosynthesis	ATP:dephospho-CoA 3'-phosphotransferase	(1) ATP_c0[c2] + (1) Dephospho-CoA_c0[c2] -> (1) ADP_c0[c2] + (1) CoA_c0[c2] + (1) H+_c0[c2]	0.000157737	No
rxn02175	Pantothenate and CoA biosynthesis	ATP:pantetheine-4'-phosphate adenylyltransferase	(1) ATP_c0[c2] + (1) Phosphopantetheine_c0[c2] <-> (1) PPi_c0[c2] + (1) Dephospho-CoA_c0[c2]	0.000157737	No
rxn06023	Pantothenate and CoA biosynthesis	CoA:apo-[acyl-carrier-protein] pantetheinephosphotransferase	(1) CoA_c0[c2] + (1) apo-ACP_c0[c2] <-> (1) Adenosine 3-5-bisphosphate_c0[c2] + (1) ACP_c0[c2]	7.88684E-05	No
rxn12510	Pantothenate and CoA biosynthesis	ATP:pantothenate 4'-phosphotransferase	(1) ATP_c0[c2] + (1) PAN_c0[c2] <-> (1) ADP_c0[c2] + (1) H+_c0[c2] + (1) 4-phosphopantothenate_c0[c2]	0.000157737	No
rxn01790	Pantothenate and CoA biosynthesis	(R)-Pantoate:NADP+ 2-oxidoreductase	(1) NADP_c0[c2] + (1) Pantoate_c0[c2] <-> (1) NADPH_c0[c2] + (1) H+_c0[c2] + (1) 2-Dehydropantoate_c0[c2]	-0.000157737	Yes
rxn00912	Pantothenate and CoA biosynthesis	5,10-Methylenetetrahydrofolate: 3-methyl-2-oxobutanoate hydroxymethyltransferase	(1) H2O_c0[c2] + (1) 3-Methyl-2-oxobutanoate_c0[c2] + (1) 5-10-Methylenetetrahydrofolate_c0[c2] <-> (1) Tetrahydrofolate_c0[c2] + (1) 2-Dehydropantoate_c0[c2]	0.000157737	No
rxn02341	Pantothenate and CoA biosynthesis	N-[(R)-4'-Phosphopantothenoyl]-L-cysteine carboxy-lyase	(1) H+_c0[c2] + (1) (R)-4'-Phosphopantothenoyl-L-cysteine_c0[c2] -> (1) CO2_c0[c2] + (1) Phosphopantetheine_c0[c2]	0.000157737	No

rxn02186	Pantothenate and CoA biosynthesis	2,3-Dihydroxy-3-methylbutanoate:NADP+ oxidoreductase (isomerizing)	(1) NADPH_c0[c2] + (1) H+_c0[c2] + (1) ALCTT_c0[c2] <-> (1) NADP_c0[c2] + (1) 2,3-Dihydroxy-isovalerate_c0[c2]	0.0208603	Yes
rxn00213	Pentose and glucuronate interconversions|Galactose metabolism|Starch and sucrose metabolism|Amino sugar and nucleotide sugar metabolism	UTP:alpha-D-glucose-1-phosphate uridylyltransferase	(1) UTP_c0[c2] + (1) Glucose-1-phosphate_c0[c2] <-> (1) PPi_c0[c2] + (1) UDP-glucose_c0[c2]	-0.000637032	No
rxn01477	Pentose phosphate pathway	6-Phospho-D-gluconate hydro-lyase(2-dehydro-3-deoxy-6-phospho-D-gluconate-forming)	(1) 6-Phospho-D-gluconate_c0[c2] -> (1) H2O_c0[c2] + (1) 2-Keto-3-deoxy-6-phosphogluconate_c0[c2]	0.139347	No
rxn01476	Pentose phosphate pathway	6-Phospho-D-glucono-1,5-lactone lactonohydrolase	(1) H2O_c0[c2] + (1) 6-phospho-D-glucono-1-5-lactone_c0[c2] -> (1) H+_c0[c2] + (1) 6-Phospho-D-gluconate_c0[c2]	0.139347	No
rxn01333	Pentose phosphate pathway	sedoheptulose-7-phosphate:D-glyceraldehyde-3-phosphate glyceronetransferase	(1) Glyceraldehyde3-phosphate_c0[c2] + (1) Sedoheptulose7-phosphate_c0[c2] <-> (1) D-fructose-6-phosphate_c0[c2] + (1) D-Erythrose4-phosphate_c0[c2]	-0.00767508	No
rxn00777	Pentose phosphate pathway|Carbon fixation in photosynthetic organisms	D-ribose-5-phosphate aldose-ketose-isomerase	(1) ribose-5-phosphate_c0[c2] <-> (1) D-Ribulose5-phosphate_c0[c2]	-0.0120932	No
rxn01200	Pentose phosphate pathway|Carbon fixation in photosynthetic organisms	Sedoheptulose-7-phosphate:D-glyceraldehyde-3-phosphate glycolaldehyde transferase	(1) Glyceraldehyde3-phosphate_c0[c2] + (1) Sedoheptulose7-phosphate_c0[c2] <-> (1) ribose-5-phosphate_c0[c2] + (1) D-Xylulose5-phosphate_c0[c2]	0.00512695	No
rxn03884	Pentose phosphate pathway|Pentose and glucuronate interconversions	2-dehydro-3-deoxy-D-gluconate-6-phosphate D-glyceraldehyde-3-phosphate-lyase	(1) 2-Keto-3-deoxy-6-phosphogluconate_c0[c2] <-> (1) Pyruvate_c0[c2] + (1) Glyceraldehyde3-phosphate_c0[c2]	0.139347	No
rxn01116	Pentose phosphate pathway|Pentose and glucuronate interconversions|Carbon fixation in photosynthetic organisms	D-Ribulose-5-phosphate 3-epimerase	(1) D-Ribulose5-phosphate_c0[c2] <-> (1) D-Xylulose5-phosphate_c0[c2]	-0.0143986	No
rxn00770	Pentose phosphate pathway|Purine metabolism	ATP:D-ribose-5-phosphate diphosphotransferase	(1) ATP_c0[c2] + (1) ribose-5-phosphate_c0[c2] <-> (1) AMP_c0[c2] + (1) H+_c0[c2] + (1) PRPP_c0[c2]	0.0167469	No
rxn03408	Peptidoglycan biosynthesis	UDP-N-acetyl-D-glucosamine:undecaprenyl-diphospho-N-acetylmuramoyl-L-alanyl-gamma-D-glutamyl-meso-2,6-diaminopimeloyl-D-alanyl-D-alanine 4-beta-N-acetylglucosaminlytransferase	(1) UDP-N-acetylglucosamine_c0[c2] + (1) Undecaprenyl-diphospho-N-acetylmuramoyl-L-alanyl-D-glutamyl-meso-2-6-diaminopimeloyl-D-alanyl-D-alanine_c0[c2] <-> (1) UDP_c0[c2] + (1) Undecaprenyl-diphospho-N-acetylmuramoyl--N-acetylglucosamine-L-ala-D-glu-meso-2-6-diaminopimeloyl-D-ala-D-ala_c0[c2]	0.000637032	No
rxn03904	Peptidoglycan biosynthesis	UDP-N-acetylmuramoyl-L-alanyl-gamma-D-glutamyl-meso-2,6-diaminopimeloyl-D-alanyl-D-alanine:undecaprenyl-phosphate phospho-N-acetylmuramoyl-pentapeptide-transferase	(1) Undecaprenylphosphate_c0[c2] + (1) UDP-N-acetylmuramoyl-L-alanyl-D-glutamyl-6-carboxy-L-lysyl-D-alanyl- D-alanine_c0[c2] <-> (1) UMP_c0[c2] + (1) Undecaprenyl-diphospho-N-acetylmuramoyl-L-alanyl-D-glutamyl-meso-2-6-diaminopimeloyl-D-alanyl-D-alanine_c0[c2]	0.000637032	No
rxn03901	Peptidoglycan biosynthesis	undecaprenyl-diphosphate phosphohydrolase	(1) H2O_c0[c2] + (1) Bactoprenyl diphosphate_c0[c2] -> (1)	0.000637032	No

			Phosphate_c0[c2] + (2) H+_c0[c2] + (1) Undecaprenylphosphate_c0[c2]		
rxn01739	Phenylalanine, tyrosine and tryptophan biosynthesis	ATP:shikimate 3-phosphotransferase	(1) ATP_c0[c2] + (1) Shikimate_c0[c2] <-> (1) ADP_c0[c2] + (1) H+_c0[c2] + (1) 3-phosphoshikimate_c0[c2]	0.0015966	No
rxn02212	Phenylalanine, tyrosine and tryptophan biosynthesis	2-Dehydro-3-deoxy-D-arabino-heptonate 7-phosphate phosphate-lyase (cyclyzing)	(1) DAHP_c0[c2] -> (1) Phosphate_c0[c2] + (1) 5-Dehydroquinate_c0[c2]	0.0015966	No
rxn01740	Phenylalanine, tyrosine and tryptophan biosynthesis	Shikimate:NADP+ 3-oxidoreductase	(1) NADP_c0[c2] + (1) Shikimate_c0[c2] <-> (1) NADPH_c0[c2] + (1) H+_c0[c2] + (1) 3-Dehydroshikimate_c0[c2]	-0.0015966	No
rxn01255	Phenylalanine, tyrosine and tryptophan biosynthesis	5-O-(1-Carboxyvinyl)-3-phosphoshikimate phosphate-lyase (chorismate-forming)	(1) 5-O--1-Carboxyvinyl-3-phosphoshikimate_c0[c2] -> (1) Phosphate_c0[c2] + (1) Chorismate_c0[c2]	0.0015966	No
rxn02508	Phenylalanine, tyrosine and tryptophan biosynthesis	N-(5-Phospho-beta-D-ribosyl)anthranilate ketol-isomerase	(1) N-5-phosphoribosyl-anthranilate_c0[c2] <-> (1) 1-(2-carboxyphenylamino)-1-deoxyribulose 5-phosphate_c0[c2]	0.00120225	No
rxn00726	Phenylalanine, tyrosine and tryptophan biosynthesis	chorismate pyruvate-lyase (amino-accepting; anthranilate-forming)	(1) NH3_c0[c2] + (1) Chorismate_c0[c2] -> (1) H2O_c0[c2] + (1) Pyruvate_c0[c2] + (1) H+_c0[c2] + (1) Anthranilate_c0[c2]	0.00120225	No
rxn00791	Phenylalanine, tyrosine and tryptophan biosynthesis	N-(5-Phospho-D-ribosyl)anthranilate:pyrophosphate phosphoribosyl-transferase	(1) PPi_c0[c2] + (1) H+_c0[c2] + (1) N-5-phosphoribosyl-anthranilate_c0[c2] <- (1) Anthranilate_c0[c2] + (1) PRPP_c0[c2]	-0.00120225	No
rxn02476	Phenylalanine, tyrosine and tryptophan biosynthesis	Phosphoenolpyruvate:3-phosphoshikimate 5-O-(1-carboxyvinyl)-transferase	(1) Phosphoenolpyruvate_c0[c2] + (1) 3-phosphoshikimate_c0[c2] <-> (1) Phosphate_c0[c2] + (1) 5-O--1-Carboxyvinyl-3-phosphoshikimate_c0[c2]	0.0015966	No
rxn02213	Phenylalanine, tyrosine and tryptophan biosynthesis	3-Dehydroquinate hydro-lyase	(1) 5-Dehydroquinate_c0[c2] -> (1) H2O_c0[c2] + (1) 3-Dehydroshikimate_c0[c2]	0.0015966	No
rxn01332	Phenylalanine, tyrosine and tryptophan biosynthesis	Phosphoenolpyruvate:D-erythrose-4-phosphate C-(1-carboxyvinyl)transferase (phosphate hydrolysing, 2-carboxy-2-oxoethyl-forming)	(1) H2O_c0[c2] + (1) Phosphoenolpyruvate_c0[c2] + (1) D-Erythrose4-phosphate_c0[c2] -> (1) Phosphate_c0[c2] + (1) DAHP_c0[c2]	0.0015966	No
rxn02507	Phenylalanine, tyrosine and tryptophan biosynthesis	1-(2-Carboxyphenylamino)-1-deoxy-D-ribulose-5-phosphate carboxy-lyase(cyclizing)	(1) H+_c0[c2] + (1) 1-(2-carboxyphenylamino)-1-deoxyribulose 5-phosphate_c0[c2] -> (1) H2O_c0[c2] + (1) CO2_c0[c2] + (1) Indoleglycerol phosphate_c0[c2]	0.00120225	No
rxn00060	Porphyrin and chlorophyll metabolism	porphobilinogen:(4-[2-carboxyethyl]-3-[carboxymethyl]pyrrol-2-yl)methyltransferase (hydrolysing)	(1) H2O_c0[c2] + (4) Porphobilinogen_c0[c2] -> (4) NH3_c0[c2] + (1) Hydroxymethylbilane_c0[c2]	0.000157737	No
rxn00029	Porphyrin and chlorophyll metabolism	5-aminolevulinate hydro-lyase (adding 5-aminolevulinate and cyclizing; porphobilinogen-forming)	(2) 5-Aminolevulinate_c0[c2] -> (2) H2O_c0[c2] + (1) H+_c0[c2] + (1) Porphobilinogen_c0[c2]	0.000630948	No
rxn03537	Porphyrin and chlorophyll metabolism	R05222	(1) GTP_c0[c2] + (1) Adenosyl cobinamide phosphate_c0[c2] <-> (1) PPi_c0[c2] + (1) H+_c0[c2] + (1) Adenosylcobinamide-GDP_c0[c2]	7.88684E-05	No

rxn04413	Porphyrin and chlorophyll metabolism	R06558	(1) GTP_c0[c2] + (1) Adenosyl cobinamide_c0[c2] <-> (1) GDP_c0[c2] + (1) H+_c0[c2] + (1) Adenosyl cobinamide phosphate_c0[c2]	7.88684E-05	No
rxn03150	Porphyrin and chlorophyll metabolism	R04594	(1) H2O_c0[c2] + (1) alpha-Ribazole 5'-phosphate_c0[c2] -> (1) Phosphate_c0[c2] + (1) alpha-Ribazole_c0[c2]	7.88684E-05	No
rxn05029	Porphyrin and chlorophyll metabolism	ATP:cobinamide Cobeta-adenosyltransferase	(1) ATP_c0[c2] + (1) H+_c0[c2] + (1) Cobinamide_c0[c2] <-> (1) Triphosphate_c0[c2] + (1) Adenosyl cobinamide_c0[c2]	7.88684E-05	No
rxn02303	Porphyrin and chlorophyll metabolism	Coproporphyrinogen:oxygen oxidoreductase(decarboxylating)	(1) O2_c0[c2] + (2) H+_c0[c2] + (1) CoproporphyrinogenIII_c0[c2] <-> (2) H2O_c0[c2] + (2) CO2_c0[c2] + (1) ProtoporphyrinogenIX_c0[c2]	7.88684E-05	No
rxn03538	Porphyrin and chlorophyll metabolism	R05223	(1) alpha-Ribazole_c0[c2] + (1) Adenosylcobinamide-GDP_c0[c2] <-> (1) H+_c0[c2] + (1) GMP_c0[c2] + (1) Calomide_c0[c2]	7.88684E-05	No
rxn00056	Porphyrin and chlorophyll metabolism	Fe(II):oxygen oxidoreductase	(1) O2_c0[c2] + (4) H+_c0[c2] + (4) Fe2+_c0[c2] <-> (2) H2O_c0[c2] + (4) fe3_c0[c2]	0.00117645	No
rxn02897	Porphyrin and chlorophyll metabolism	Nicotinate-nucleotide:dimethylbenzimidazole phospho-D-ribosyltransferase	(1) Nicotinate ribonucleotide_c0[c2] + (1) Dimethylbenzimidazole_c0[c2] <-> (1) H+_c0[c2] + (1) Niacin_c0[c2] + (1) alpha-Ribazole 5'-phosphate_c0[c2]	7.88684E-05	No
rxn02056	Porphyrin and chlorophyll metabolism	S-Adenosyl-L-methionine:uroporphyrin-III C-methyltransferase	(2) H+_c0[c2] + (1) Siroheme_c0[c2] <-> (1) Sirohydrochlorin_c0[c2] + (1) Fe2+_c0[c2]	-7.88684E-05	No
rxn02288	Porphyrin and chlorophyll metabolism	Uroporphyrinogen-III carboxy-lyase	(4) H+_c0[c2] + (1) UroporphyrinogenIII_c0[c2] <-> (4) CO2_c0[c2] + (1) CoproporphyrinogenIII_c0[c2]	7.88684E-05	No
rxn00224	Porphyrin and chlorophyll metabolism	protoheme ferro-lyase (protoporphyrin-forming)	(1) Protoporphyrin_c0[c2] + (1) Fe2+_c0[c2] <-> (1) Heme_c0[c2] + (2) H+_c0[c2]	7.88684E-05	No
rxn01297	Purine metabolism	hypoxanthine:NAD+ oxidoreductase	(1) H2O_c0[c2] + (1) NAD_c0[c2] + (1) HYXN_c0[c2] <-> (1) NADH_c0[c2] + (1) H+_c0[c2] + (1) XAN_c0[c2]	0.00436593	No
rxn00131	Purine metabolism	AMP phosphoribohydrolase	(1) H2O_c0[c2] + (1) AMP_c0[c2] <-> (1) ribose-5-phosphate_c0[c2] + (1) Adenine_c0[c2]	-0.000473211	No
rxn00917	Purine metabolism	Xanthosine-5'-phosphate:L-glutamine amido-ligase (AMP-forming)	(1) H2O_c0[c2] + (1) ATP_c0[c2] + (1) L-Glutamine_c0[c2] + (1) XMP_c0[c2] -> (1) PPi_c0[c2] + (1) AMP_c0[c2] + (1) L-Glutamate_c0[c2] + (3) H+_c0[c2] + (1) GMP_c0[c2]	0.00436593	No
rxn00927	Purine metabolism	Adenosine ribohydrolase	(1) H2O_c0[c2] + (1) Adenosine_c0[c2] <-> (1) D-Ribose_c0[c2] + (1) Adenine_c0[c2]	0.000473211	No
rxn03147	Purine metabolism	1-(5-Phosphoribosyl)-5-amino-4-carboxyimidazole:L-aspartate ligase (ADP-forming)	(1) ATP_c0[c2] + (1) L-Aspartate_c0[c2] + (1) 5'-Phosphoribosyl-4-carboxy-5-aminoimidazole_c0[c2] -> (1) ADP_c0[c2] + (1) Phosphate_c0[c2] + (1) H+_c0[c2] + (1) SAICAR_c0[c2]	0.00357271	No
rxn01544	Purine metabolism	XMP:pyrophosphate phosphoribosyltransferase	(1) PPi_c0[c2] + (1) H+_c0[c2] + (1) XMP_c0[c2] <- (1) PRPP_c0[c2] + (1) XAN_c0[c2]	-0.00436593	No

rxn00832	Purine metabolism	IMP 1,2-hydrolase (decyclizing)	(1) H2O_c0[c2] + (1) IMP_c0[c2] <-> (1) FAICAR_c0[c2]	-0.00559159	No
rxn01353	Purine metabolism	ATP:dGDP phosphotransferase	(1) ATP_c0[c2] + (1) dGDP_c0[c2] <-> (1) ADP_c0[c2] + (1) dGTP_c0[c2]	0.000522715	No
rxn02895	Purine metabolism	5-Phospho-D-ribosylamine:glycine ligase (ADP-forming)	(1) ATP_c0[c2] + (1) Glycine_c0[c2] + (1) 5-Phosphoribosylamine_c0[c2] -> (1) ADP_c0[c2] + (1) Phosphate_c0[c2] + (1) H+_c0[c2] + (1) GAR_c0[c2]	0.00365158	No
rxn05231	Purine metabolism	2'-Deoxyadenosine 5'-diphosphate:oxidized-thioredoxin 2'-oxidoreductase	(1) ADP_c0[c2] + (1) trdrd_c0[c2] -> (1) H2O_c0[c2] + (1) dADP_c0[c2] + (1) trdox_c0[c2]	0.000293275	No
rxn03136	Purine metabolism	1-(5'-Phosphoribosyl)-5-amino-4-(N-succinocarboxamide)-imidazole AMP-lyase	(1) SAICAR_c0[c2] <-> (1) H+_c0[c2] + (1) Fumarate_c0[c2] + (1) AICAR_c0[c2]	0.00357271	No
rxn02937	Purine metabolism	2-(Formamido)-N1-(5-phosphoribosyl)acetamidine cyclo-ligase (ADP-forming)	(1) ATP_c0[c2] + (1) 5'-Phosphoribosylformylglycinamidine_c0[c2] <-> (1) ADP_c0[c2] + (1) Phosphate_c0[c2] + (1) H+_c0[c2] + (1) AIR_c0[c2]	0.00365158	No
rxn01299	Purine metabolism	Inosine ribohydrolase	(1) H2O_c0[c2] + (1) Inosine_c0[c2] <-> (1) D-Ribose_c0[c2] + (1) HYXN_c0[c2]	-0.000473211	No
rxn03084	Purine metabolism	5'-Phosphoribosylformylglycinamide:L-glutamine amido-ligase (ADP-forming)	(1) H2O_c0[c2] + (1) ATP_c0[c2] + (1) L-Glutamine_c0[c2] + (1) N-Formyl-GAR_c0[c2] -> (1) ADP_c0[c2] + (1) Phosphate_c0[c2] + (1) L-Glutamate_c0[c2] + (1) H+_c0[c2] + (1) 5'-Phosphoribosylformylglycinamidine_c0[c2]	0.00365158	No
rxn05233	Purine metabolism	2'-Deoxyguanosine 5'-diphosphate:oxidized-thioredoxin 2'-oxidoreductase	(1) GDP_c0[c2] + (1) trdrd_c0[c2] -> (1) H2O_c0[c2] + (1) dGDP_c0[c2] + (1) trdox_c0[c2]	0.000522715	No
rxn00239	Purine metabolism	ATP:GMP phosphotransferase	(1) ATP_c0[c2] + (1) H+_c0[c2] + (1) GMP_c0[c2] <-> (1) ADP_c0[c2] + (1) GDP_c0[c2]	0.0044448	No
rxn00839	Purine metabolism	ATP:dADP phosphotransferase	(1) ATP_c0[c2] + (1) dADP_c0[c2] <-> (1) ADP_c0[c2] + (1) dATP_c0[c2]	0.000293275	No
rxn00800	Purine metabolism|Alanine, aspartate and glutamate metabolism	N6-(1,2-dicarboxyethyl)AMP AMP-lyase (fumarate-forming)	(1) Adenylosuccinate_c0[c2] <-> (1) AMP_c0[c2] + (1) Fumarate_c0[c2]	0.00559159	No
rxn00790	Purine metabolism|Alanine, aspartate and glutamate metabolism	5-phosphoribosylamine:diphosphate phospho-alpha-D-ribosyltransferase (glutamate-amidating)	(1) PPi_c0[c2] + (1) L-Glutamate_c0[c2] + (1) H+_c0[c2] + (1) 5-Phosphoribosylamine_c0[c2] <- (1) H2O_c0[c2] + (1) L-Glutamine_c0[c2] + (1) PRPP_c0[c2]	-0.00365158	No
rxn00838	Purine metabolism|Alanine, aspartate and glutamate metabolism	IMP:L-aspartate ligase (GDP-forming)	(1) GTP_c0[c2] + (1) L-Aspartate_c0[c2] + (1) IMP_c0[c2] -> (1) Phosphate_c0[c2] + (1) GDP_c0[c2] + (2) H+_c0[c2] + (1) Adenylosuccinate_c0[c2]	0.00559159	No
rxn03137	Purine metabolism|One carbon pool by folate	10-Formyltetrahydrofolate:5'-phosphoribosyl-5-amino-4-imidazolecarboxamide formyltransferase	(1) 10-Formyltetrahydrofolate_c0[c2] + (1) AICAR_c0[c2] <-> (1) Tetrahydrofolate_c0[c2] + (1) FAICAR_c0[c2]	0.00559159	No
rxn03004	Purine metabolism|One carbon pool by folate	10-Formyltetrahydrofolate:5'-	(1) 10-Formyltetrahydrofolate_c0[c2] + (1) GAR_c0[c2] <-> (1) H+_c0[c2] +	0.00365158	No

		phosphoribosylglycinamide formyltransferase	(1) Tetrahydrofolate_c0[c2] + (1) N-Formyl-GAR_c0[c2]		
rxn00361	Purine metabolism|Sulfur metabolism	ATP:adenylylsulfate 3'-phosphotransferase	(1) ATP_c0[c2] + (1) APS_c0[c2] <-> (1) ADP_c0[c2] + (1) 3-phosphoadenylylsulfate_c0[c2] + (1) H+_c0[c2]	-7.88684E-05	No
rxn00379	Purine metabolism|Sulfur metabolism	ATP:sulfate adenylyltransferase	(1) ATP_c0[c2] + (1) Sulfate_c0[c2] <-> (1) PPi_c0[c2] + (1) APS_c0[c2]	0.0151815	No
rxn00364	Pyrimidine metabolism	ATP:CMP phosphotransferase	(1) ATP_c0[c2] + (1) CMP_c0[c2] + (1) H+_c0[c2] <-> (1) ADP_c0[c2] + (1) CDP_c0[c2]	0.0140658	No
rxn01673	Pyrimidine metabolism	ATP:dCDP phosphotransferase	(1) ATP_c0[c2] + (1) dCDP_c0[c2] <-> (1) ADP_c0[c2] + (1) dCTP_c0[c2]	0.000522715	No
rxn00710	Pyrimidine metabolism	orotidine-5'-phosphate carboxy-lyase (UMP-forming)	(1) H+_c0[c2] + (1) Orotidylic acid_c0[c2] -> (1) CO2_c0[c2] + (1) UMP_c0[c2]	0.00527168	No
rxn01362	Pyrimidine metabolism	Orotidine-5'-phosphate:diphosphate phospho-alpha-D-ribosyl-transferase	(1) PPi_c0[c2] + (1) H+_c0[c2] + (1) Orotidylic acid_c0[c2] <- (1) PRPP_c0[c2] + (1) Orotate_c0[c2]	-0.00527168	No
rxn01465	Pyrimidine metabolism	(S)-dihydroorotate amidohydrolase	(1) H2O_c0[c2] + (1) S-Dihydroorotate_c0[c2] <-> (1) H+_c0[c2] + (1) N-Carbamoyl-L-aspartate_c0[c2]	-0.00527168	No
rxn01678	Pyrimidine metabolism	ATP:dUDP phosphotransferase	(1) ATP_c0[c2] + (1) dUDP_c0[c2] <-> (1) ADP_c0[c2] + (1) dUTP_c0[c2]	0.000293275	No
rxn00410	Pyrimidine metabolism	UTP:ammonia ligase (ADP-forming)	(1) ATP_c0[c2] + (1) NH3_c0[c2] + (1) UTP_c0[c2] <-> (1) ADP_c0[c2] + (1) Phosphate_c0[c2] + (1) CTP_c0[c2] + (2) H+_c0[c2]	0.00266487	No
rxn01512	Pyrimidine metabolism	ATP:dTDP phosphotransferase	(1) ATP_c0[c2] + (1) dTDP_c0[c2] <-> (1) ADP_c0[c2] + (1) TTP_c0[c2]	0.000930306	No
rxn06076	Pyrimidine metabolism	2'-Deoxycytidine diphosphate:oxidized-thioredoxin 2'-oxidoreductase	(1) H2O_c0[c2] + (1) dCDP_c0[c2] + (1) trdox_c0[c2] <- (1) CDP_c0[c2] + (1) trdrd_c0[c2]	-0.000522715	No
rxn00409	Pyrimidine metabolism	ATP:CDP phosphotransferase	(1) ATP_c0[c2] + (1) CDP_c0[c2] <-> (1) ADP_c0[c2] + (1) CTP_c0[c2]	0.0135431	No
rxn01519	Pyrimidine metabolism	dUTP nucleotidohydrolase	(1) H2O_c0[c2] + (1) dUTP_c0[c2] -> (1) PPi_c0[c2] + (2) H+_c0[c2] + (1) dUMP_c0[c2]	0.000293275	No
rxn01513	Pyrimidine metabolism	ATP:dTMP phosphotransferase	(1) ATP_c0[c2] + (1) H+_c0[c2] + (1) dTMP_c0[c2] <-> (1) ADP_c0[c2] + (1) dTDP_c0[c2]	0.000293275	No
rxn06075	Pyrimidine metabolism	2'-Deoxyuridine 5'-diphosphate:oxidized-thioredoxin 2'-oxidoreductase	(1) H2O_c0[c2] + (1) dUDP_c0[c2] + (1) trdox_c0[c2] <- (1) UDP_c0[c2] + (1) trdrd_c0[c2]	-0.000293275	No
rxn05289	Pyrimidine metabolism	NADPH:oxidized-thioredoxin oxidoreductase	(1) NADPH_c0[c2] + (1) H+_c0[c2] + (1) trdox_c0[c2] <-> (1) NADP_c0[c2] + (1) trdrd_c0[c2]	0.00722097	No
rxn00117	Pyrimidine metabolism	ATP:UDP phosphotransferase	(1) ATP_c0[c2] + (1) UDP_c0[c2] <-> (1) ADP_c0[c2] + (1) UTP_c0[c2]	0.000343757	No
rxn01018	Pyrimidine metabolism|Alanine, aspartate and glutamate metabolism	carbamoyl-phosphate:L-aspartate carbamoyltransferase	(1) L-Aspartate_c0[c2] + (1) Carbamoylphosphate_c0[c2] -> (1) Phosphate_c0[c2] + (1) H+_c0[c2] + (1) N-Carbamoyl-L-aspartate_c0[c2]	0.00527168	No
rxn00414	Pyrimidine metabolism|Alanine, aspartate and glutamate metabolism	hydrogen-carbonate:L-glutamine amido-ligase (ADP-forming, carbamate-phosphorylating)	(1) H2O_c0[c2] + (2) ATP_c0[c2] + (1) L-Glutamine_c0[c2] + (1) H2CO3_c0[c2] -> (2) ADP_c0[c2] + (1) Phosphate_c0[c2] + (1) L-	0.0115552	No

			Glutamate_c0[c2] + (2) H+_c0[c2] + (1) Carbamoylphosphate_c0[c2]		
rxn01520	Pyrimidine metabolism|One carbon pool by folate	5,10-Methylenetetrahydrofolate: dUMP C-methyltransferase	(1) 5-10-Methylenetetrahydrofolate_c0[c2] + (1) dUMP_c0[c2] -> (1) dTMP_c0[c2] + (1) Dihydrofolate_c0[c2]	0.000293275	No
rxn00146	Pyruvate metabolism	(R)-Lactate:ferricytochrome-c 2-oxidoreductase	(2) Cytochrome c3+_c0[c2] + (1) D-Lactate_c0[c2] <-> (1) Pyruvate_c0[c2] + (2) H+_c0[c2] + (2) Cytochrome c2+_c0[c2]	-0.144596	No
rxn00500	Pyruvate metabolism	(R)-Lactate:NAD+ oxidoreductase	(1) NAD_c0[c2] + (1) D-Lactate_c0[c2] <-> (1) NADH_c0[c2] + (1) Pyruvate_c0[c2] + (1) H+_c0[c2]	0.144596	No
rxn00151	Pyruvate metabolism|Carbon fixation in photosynthetic organisms	ATP:pyruvate,phosphate phosphotransferase	(1) ATP_c0[c2] + (1) Phosphate_c0[c2] + (1) Pyruvate_c0[c2] <-> (1) PPi_c0[c2] + (1) AMP_c0[c2] + (1) Phosphoenolpyruvate_c0[c2] + (2) H+_c0[c2]	-0.0540782	No
rxn00251	Pyruvate metabolism|Methane metabolism|Carbon fixation in photosynthetic organisms|Carbon fixation pathways in prokaryotes	phosphate:oxaloacetate carboxy-lyase (adding phosphate;phosphoenolpyruvate-forming)	(1) Phosphate_c0[c2] + (1) Oxaloacetate_c0[c2] + (1) H+_c0[c2] <-> (1) H2O_c0[c2] + (1) CO2_c0[c2] + (1) Phosphoenolpyruvate_c0[c2]	-0.074407	No
rxn05040	Riboflavin metabolism	D-ribulose 5-phosphate formate-lyase (L-3,4-dihydroxybutan-2-one 4-phosphate-forming)	(1) D-Ribulose5-phosphate_c0[c2] -> (1) Formate_c0[c2] + (1) H+_c0[c2] + (1) 3-4-dihydroxy-2-butanone4-phosphate_c0[c2]	0.000315474	No
rxn00300	Riboflavin metabolism	GTP 7,8-8,9-dihydrolase (diphosphate-forming)	(3) H2O_c0[c2] + (1) GTP_c0[c2] -> (1) PPi_c0[c2] + (1) Formate_c0[c2] + (3) H+_c0[c2] + (1) 2,5-Diamino-6-(5'-phosphoribosylamino)-4-pyrimidineone_c0[c2]	0.000157737	No
rxn00392	Riboflavin metabolism	ATP:riboflavin 5'-phosphotransferase	(1) ATP_c0[c2] + (1) Riboflavin_c0[c2] <-> (1) ADP_c0[c2] + (1) FMN_c0[c2] + (1) H+_c0[c2]	7.88684E-05	No
rxn03080	Riboflavin metabolism	5-amino-6-(D-ribitylamino)uracil butanedionetransferase	(1) 4--1-D-Ribitylamino-5-aminouracil_c0[c2] + (1) 3-4-dihydroxy-2-butanone4-phosphate_c0[c2] <-> (2) H2O_c0[c2] + (1) Phosphate_c0[c2] + (1) H+_c0[c2] + (1) 6-7-Dimethyl-8--1-D-ribityllumazine_c0[c2]	0.000315474	No
rxn00048	Riboflavin metabolism	6,7-Dimethyl-8-(1-D-ribityl)lumazine:6,7-dimethyl-8-(1-D-ribityl)lumazine 2,3-butanediyltransferase	(1) H+_c0[c2] + (2) 6-7-Dimethyl-8--1-D-ribityllumazine_c0[c2] -> (1) Riboflavin_c0[c2] + (1) 4--1-D-Ribitylamino-5-aminouracil_c0[c2]	0.000157737	No
rxn02475	Riboflavin metabolism	2,5-Diamino-6-hydroxy-4-(5-phosphoribosylamino)-pyrimidine 2-aminohydrolase	(1) H2O_c0[c2] + (1) H+_c0[c2] + (1) 2,5-Diamino-6-(5'-phosphoribosylamino)-4-pyrimidineone_c0[c2] -> (1) NH3_c0[c2] + (1) 5-Amino-6--5-phosphoribosylaminouracil_c0[c2]	0.000157737	No
rxn00122	Riboflavin metabolism	ATP:FMN adenylyltransferase	(1) ATP_c0[c2] + (1) FMN_c0[c2] -> (1) PPi_c0[c2] + (1) FAD_c0[c2]	7.88684E-05	No
rxn02474	Riboflavin metabolism	5-amino-6-(5-phosphoribitylamino)uracil: NADP+ 1'-oxidoreductase	(1) NADP_c0[c2] + (1) 5-Amino-6--5-phosphoribitylaminouracil_c0[c2] <-> (1) NADPH_c0[c2] + (1) H+_c0[c2] + (1) 5-Amino-6--5-phosphoribosylaminouracil_c0[c2]	-0.000157737	No

rxn00695	Starch and sucrose metabolism|Amino sugar and nucleotide sugar metabolism	ATP:alpha-D-glucose-1-phosphate adenylyltransferase	(1) ATP_c0[c2] + (1) Glucose-1-phosphate_c0[c2] <-> (1) PPi_c0[c2] + (1) ADPglucose_c0[c2]	-0.0373561	No
rxn01997	Streptomycin biosynthesis|Polyketide sugar unit biosynthesis|Biosynthesis of vancomycin group antibiotics	dTDPglucose 4,6-hydro-lyase	(1) dTDPglucose_c0[c2] -> (1) H2O_c0[c2] + (1) dTDP-4-oxo-6-deoxy-D-glucose_c0[c2]	0.000637032	No
rxn05239	Sulfur metabolism	adenosine 3',5'-bisphosphate,sulfite:oxidized-thioredoxin oxidoreductase (3'-phosphoadenosine-5'-phosphosulfate -forming)	(1) 3-phosphoadenylylsulfate_c0[c2] + (1) trdrd_c0[c2] <-> (1) Adenosine 3-5-bisphosphate_c0[c2] + (1) H+_c0[c2] + (1) Sulfite_c0[c2] + (1) trdox_c0[c2]	-7.88684E-05	No
rxn00623	Sulfur metabolism	hydrogen-sulfide:NADP+ oxidoreductase	(3) H2O_c0[c2] + (3) NADP_c0[c2] + (1) H2S_c0[c2] <-> (3) NADPH_c0[c2] + (3) H+_c0[c2] + (1) Sulfite_c0[c2]	-0.00558899	No
rxn03958	Terpenoid backbone biosynthesis	1-Deoxy-D-xylulose-5-phosphate isomeroreductase	(1) NADP_c0[c2] + (1) 2-C-methyl-D-erythritol4-phosphate_c0[c2] <-> (1) NADPH_c0[c2] + (1) H+_c0[c2] + (1) 1-deoxy-D-xylulose5-phosphate_c0[c2]	-0.00890019	No
rxn03910	Terpenoid backbone biosynthesis	2-Phospho-4-(cytidine 5'-diphospho)-2-C-methyl-D-erythritol CMP-lyase (cyclizing)	(1) 2-phospho-4--cytidine5-diphospho-2-C-methyl-D-erythritol_c0[c2] <-> (1) CMP_c0[c2] + (1) 2-C-methyl-D-erythritol2-4-cyclodiphosphate_c0[c2]	0.00890019	No
rxn03908	Terpenoid backbone biosynthesis	ATP:4-(Cytidine 5'-diphospho)-2-C-methyl-D-erythritol 2-phosphotransferase	(1) ATP_c0[c2] + (1) 4--cytidine5-diphospho-2-C-methyl-D-erythritol_c0[c2] <-> (1) ADP_c0[c2] + (1) H+_c0[c2] + (1) 2-phospho-4--cytidine5-diphospho-2-C-methyl-D-erythritol_c0[c2]	0.00890019	No
rxn08352	Terpenoid backbone biosynthesis	R08210	(1) NADH_c0[c2] + (1) H+_c0[c2] + (1) 1-Hydroxy-2-methyl-2-butenyl 4-diphosphate_c0[c2] -> (1) H2O_c0[c2] + (1) NAD_c0[c2] + (1) DMAPP_c0[c2]	0.000873637	No
rxn08756	Terpenoid backbone biosynthesis	isopentenyl-diphosphate:NAD+ oxidoreductase	(1) NADH_c0[c2] + (1) H+_c0[c2] + (1) 1-Hydroxy-2-methyl-2-butenyl 4-diphosphate_c0[c2] -> (1) H2O_c0[c2] + (1) NAD_c0[c2] + (1) Isopentenyldiphosphate_c0[c2]	0.00802655	No
rxn03907	Terpenoid backbone biosynthesis	CTP: 2-C-Methyl-D-erythritol 4-phosphate cytidylyltransferase	(1) CTP_c0[c2] + (1) 2-C-methyl-D-erythritol4-phosphate_c0[c2] <-> (1) PPi_c0[c2] + (1) 4--cytidine5-diphospho-2-C-methyl-D-erythritol_c0[c2]	0.00890019	No
rxn01213	Terpenoid backbone biosynthesis	GPPSYN-RXN	(1) Isopentenyldiphosphate_c0[c2] + (1) DMAPP_c0[c2] -> (1) PPi_c0[c2] + (1) H+_c0[c2] + (1) Geranyldiphosphate_c0[c2]	0.000873637	No
rxn03909	Terpenoid backbone biosynthesis	1-Deoxy-D-xylulose-5-phosphate pyruvate-lyase (carboxylating)	(1) Pyruvate_c0[c2] + (1) H+_c0[c2] + (1) Glyceraldehyde3-phosphate_c0[c2] -> (1) CO2_c0[c2] + (1) 1-deoxy-D-xylulose5-phosphate_c0[c2]	0.00897906	No
rxn01466	Terpenoid backbone biosynthesis	Geranyl-diphosphate:isopentenyl-diphosphate geranyltrans-transferase	(1) Isopentenyldiphosphate_c0[c2] + (1) Geranyldiphosphate_c0[c2] -> (1) PPi_c0[c2] + (1) H+_c0[c2] + (1) Farnesyldiphosphate_c0[c2]	0.000873637	No
rxn00533	Tetracycline biosynthesis|Pyruvate metabolism|Propanoate	Acetyl-CoA:carbon-dioxide ligase (ADP-forming)	(1) ATP_c0[c2] + (1) Acetyl-CoA_c0[c2] + (1) H2CO3_c0[c2] <-> (1) ADP_c0[c2] + (1) Phosphate_c0[c2]	0.0656898	No

	metabolism|Carbon fixation pathways in prokaryotes		+ (1) H+_c0[c2] + (1) Malonyl-CoA_c0[c2]		
rxn02305	Thiamine metabolism	2-methyl-4-amino-5-hydroxymethylpyrimidine-diphosphate:4-methyl-5-(2-phosphoethyl)-thiazole 2-methyl-4-aminopyridine-5-methenyltransferase	(1) 4-Methyl-5--2-phosphoethyl-thiazole_c0[c2] + (1) 4-Amino-2-methyl-5-diphosphomethylpyrimidine_c0[c2] <-> (1) PPi_c0[c2] + (1) Thiamine phosphate_c0[c2]	7.88684E-05	No
rxn02484	Thiamine metabolism	ATP:4-amino-5-hydroxymethyl-2-methylpyrimidine 5-phosphotransferase	(1) ATP_c0[c2] + (1) Toxopyrimidine_c0[c2] <-> (1) ADP_c0[c2] + (1) H+_c0[c2] + (1) 4-Amino-5-phosphomethyl-2-methylpyrimidine_c0[c2]	-0.000236605	No
rxn03108	Thiamine metabolism	ATP:4-amino-2-methyl-5-phosphomethylpyrimidine phosphotransferase	(1) ATP_c0[c2] + (1) H+_c0[c2] + (1) 4-Amino-5-phosphomethyl-2-methylpyrimidine_c0[c2] <-> (1) ADP_c0[c2] + (1) 4-Amino-2-methyl-5-diphosphomethylpyrimidine_c0[c2]	7.88684E-05	No
rxn00438	Thiamine metabolism	ATP:thiamin-phosphate phosphotransferase	(1) ATP_c0[c2] + (1) H+_c0[c2] + (1) Thiamine phosphate_c0[c2] <-> (1) ADP_c0[c2] + (1) TPP_c0[c2]	7.88684E-05	No
rxn11946	Ubiquinone and other terpenoid-quinone biosynthesis	R05614	(1) S-Adenosyl-L-methionine_c0[c2] + (1) 2-Octaprenyl-3-methyl-5-hydroxy-6-methoxy-1,4-benzoquinone_c0[c2] <-> (1) S-Adenosyl-homocysteine_c0[c2] + (1) H+_c0[c2] + (1) Ubiquinone-8_c0[c2]	7.88684E-05	No
rxn04139	Ubiquinone and other terpenoid-quinone biosynthesis	2-Octaprenyl-3-methyl-6-methoxy-1,4-benzoquinone ,NADPH2:oxygen oxidoreductase	(1) NADPH_c0[c2] + (1) O2_c0[c2] + (1) H+_c0[c2] + (1) 2-Octaprenyl-3-methyl-6-methoxy-1,4-benzoquinone_c0[c2] -> (1) H2O_c0[c2] + (1) NADP_c0[c2] + (1) 2-Octaprenyl-3-methyl-5-hydroxy-6-methoxy-1,4-benzoquinone_c0[c2]	7.88684E-05	No
rxn03893	Ubiquinone and other terpenoid-quinone biosynthesis	all-trans-octaprenyl-diphosphate:4-hydroxybenzoate 3-octaprenyltransferase	(1) 4-Hydroxybenzoate_c0[c2] + (1) Farnesylfarnesylgeraniol_c0[c2] -> (1) PPi_c0[c2] + (1) H+_c0[c2] + (1) 3-Octaprenyl-4-hydroxybenzoate_c0[c2]	7.88684E-05	No
rxn03394	Ubiquinone and other terpenoid-quinone biosynthesis	R04987	(1) NADPH_c0[c2] + (1) O2_c0[c2] + (1) H+_c0[c2] + (1) 2-Octaprenylphenol_c0[c2] -> (1) H2O_c0[c2] + (1) NADP_c0[c2] + (1) 2-Octaprenyl-6-hydroxyphenol_c0[c2]	7.88684E-05	No
rxn03395	Ubiquinone and other terpenoid-quinone biosynthesis	S-adenosyl-L-methionine:3-(all-trans-octaprenyl)benzene-1,2-diol 2-O-methyltransferase	(1) S-Adenosyl-L-methionine_c0[c2] + (1) 2-Octaprenyl-6-hydroxyphenol_c0[c2] <-> (1) S-Adenosyl-homocysteine_c0[c2] + (1) H+_c0[c2] + (1) 2-Octaprenyl-6-methoxyphenol_c0[c2]	7.88684E-05	No
rxn03436	Valine, leucine and isoleucine biosynthesis	(S)-2-Aceto-2-hydroxybutanoate:NADP+ oxidoreductase (isomerizing)	(1) 2-Aceto-2-hydroxybutanoate_c0[c2] <-> (1) (R)-3-Hydroxy-3-methyl-2-oxopentanoate_c0[c2]	0.0083284	Yes
rxn03435	Valine, leucine and isoleucine biosynthesis	(R)-2,3-Dihydroxy-3-methylpentanoate:NADP+ oxidoreductase (isomerizing)	(1) NADP_c0[c2] + (1) 2,3-Dihydroxy-3-methylvalerate_c0[c2] <-> (1) NADPH_c0[c2] + (1) H+_c0[c2] + (1) (R)-3-Hydroxy-3-methyl-2-oxopentanoate_c0[c2]	-0.0083284	Yes
rxn01045	Valine, leucine and isoleucine biosynthesis	L-Valine:NAD+ oxidoreductase(deaminating)	(1) H2O_c0[c2] + (1) NAD_c0[c2] + (1) L-Valine_c0[c2] <-> (1) NADH_c0[c2] + (1) NH3_c0[c2] + (1) H+_c0[c2] + (1) 3-Methyl-2-oxobutanoate_c0[c2]	-0.00897154	No

rxn01573	Valine, leucine and isoleucine biosynthesis	L-Isoleucine:NAD+ oxidoreductase(deaminating)	(1) H2O_c0[c2] + (1) NAD_c0[c2] + (1) L-Isoleucine_c0[c2] <-> (1) NADH_c0[c2] + (1) NH3_c0[c2] + (1) H+_c0[c2] + (1) 3MOP_c0[c2]	-0.00615872	No
rxn03437	Valine, leucine and isoleucine biosynthesis	(R)-2,3-Dihydroxy-3-methylpentanoate hydro-lyase	(1) 2,3-Dihydroxy-3-methylvalerate_c0[c2] -> (1) H2O_c0[c2] + (1) 3MOP_c0[c2]	0.0083284	No
rxn03194	Valine, leucine and isoleucine biosynthesis	(S)-2-Aceto-2-hydroxybutanoate pyruvate-lyase (carboxylating)	(1) 2-Oxobutyrate_c0[c2] + (1) 2-Hydroxyethyl-ThPP_c0[c2] <-> (1) TPP_c0[c2] + (1) 2-Aceto-2-hydroxybutanoate_c0[c2]	0.0083284	No
rxn00902	Valine, leucine and isoleucine biosynthesis|Pyruvate metabolism	acetyl-CoA:3-methyl-2-oxobutanoate C-acetyltransferase (thioester-hydrolysing, carboxymethyl-forming)	(1) CoA_c0[c2] + (1) H+_c0[c2] + (1) 2-Isopropylmalate_c0[c2] <- (1) H2O_c0[c2] + (1) Acetyl-CoA_c0[c2] + (1) 3-Methyl-2-oxobutanoate_c0[c2]	-0.011731	No
rxn07434	Valine, leucine and isoleucine degradation	R07603	(1) TPP_c0[c2] + (1) H+_c0[c2] + (1) 3MOP_c0[c2] -> (1) CO2_c0[c2] + (1) 2-Methyl-1-hydroxybutyl-TPP_c0[c2]	0.00216968	No
rxn07433	Valine, leucine and isoleucine degradation	R07602	(1) Lipoamide_c0[c2] + (1) 3-Methyl-1-hydroxybutyl-TPP_c0[c2] <-> (1) TPP_c0[c2] + (1) S-(3-Methylbutanoyl)-dihydrolipoamide-E_c0[c2]	0.00216968	No
rxn07435	Valine, leucine and isoleucine degradation	R07604	(1) Lipoamide_c0[c2] + (1) 2-Methyl-1-hydroxybutyl-TPP_c0[c2] <-> (1) TPP_c0[c2] + (1) S-(2-Methylbutanoyl)-dihydrolipoamide-E_c0[c2]	0.00216968	No
rxn06586	Valine, leucine and isoleucine degradation	3-methylbutanoyl-CoA:enzyme N6-(dihydrolipoyl)lysine S-(3-methylbutanoyl)transferase	(1) Dihydrolipoamide_c0[c2] + (1) Isovaleryl-CoA_c0[c2] <-> (1) CoA_c0[c2] + (1) S-(3-Methylbutanoyl)-dihydrolipoamide-E_c0[c2]	-0.00216968	No
rxn07432	Valine, leucine and isoleucine degradation	R07601	(1) TPP_c0[c2] + (1) H+_c0[c2] + (1) 4MOP_c0[c2] -> (1) CO2_c0[c2] + (1) 3-Methyl-1-hydroxybutyl-TPP_c0[c2]	0.00216968	No
rxn06335	Valine, leucine and isoleucine degradation	(S)-2-methylbutanoyl-CoA:enzyme N6-(dihydrolipoyl)lysine S-(2-methylbutanoyl)transferase	(1) Dihydrolipoamide_c0[c2] + (1) 2-Methylbutyryl-CoA_c0[c2] <-> (1) CoA_c0[c2] + (1) S-(2-Methylbutanoyl)-dihydrolipoamide-E_c0[c2]	-0.00216968	No
rxn00804	Valine, leucine and isoleucine degradation|Valine, leucine and isoleucine biosynthesis	L-leucine:NAD+ oxidoreductase (deaminating)	(1) H2O_c0[c2] + (1) NAD_c0[c2] + (1) L-Leucine_c0[c2] <-> (1) NADH_c0[c2] + (1) NH3_c0[c2] + (1) H+_c0[c2] + (1) 4MOP_c0[c2]	-0.00956133	No
***Variovorax* sp. CF313, community in MOPS medium**					
rxn00555	Alanine, aspartate and glutamate metabolism|Amino sugar and nucleotide sugar metabolism	L-glutamine:D-fructose-6-phosphate isomerase (deaminating)	(1) L-Glutamine_c0[c1] + (1) D-fructose-6-phosphate_c0[c1] <-> (1) L-Glutamate_c0[c1] + (1) D-Glucosamine phosphate_c0[c1]	0.00254813	No
rxn00802	Alanine, aspartate and glutamate metabolism|Arginine and proline metabolism	2-(Nomega-L-arginino)succinate arginine-lyase (fumarate-forming)	(1) L-Argininosuccinate_c0[c1] <-> (1) L-Arginine_c0[c1] + (1) Fumarate_c0[c1]	0.00628348	No
rxn00503	Alanine, aspartate and glutamate metabolism|Arginine and proline metabolism	(S)-1-pyrroline-5-carboxylate:NAD+ oxidoreductase	(2) H2O_c0[c1] + (1) NAD_c0[c1] + (1) 1-Pyrroline-5-carboxylate_c0[c1] <-> (1) NADH_c0[c1] + (1) L-Glutamate_c0[c1] + (1) H+_c0[c1]	0.00463958	No

rxn01434	Alanine, aspartate and glutamate metabolism|Arginine and proline metabolism	L-Citrulline:L-aspartate ligase (AMP-forming)	(1) ATP_c0[c1] + (1) L-Aspartate_c0[c1] + (1) Citrulline_c0[c1] <-> (1) PPi_c0[c1] + (1) AMP_c0[c1] + (2) H+_c0[c1] + (1) L-Argininosuccinate_c0[c1]	0.00628348	No
rxn00182	Alanine, aspartate and glutamate metabolism|Arginine and proline metabolism|Taurine and hypotaurine metabolism|D-Glutamine and D-glutamate metabolism|Nitrogen metabolism	L-glutamate:NAD+ oxidoreductase (deaminating)	(1) H2O_c0[c1] + (1) NAD_c0[c1] + (1) L-Glutamate_c0[c1] <-> (1) NADH_c0[c1] + (1) NH3_c0[c1] + (1) 2-Oxoglutarate_c0[c1] + (1) H+_c0[c1]	-0.180611	No
rxn00260	Alanine, aspartate and glutamate metabolism|Carbon fixation in photosynthetic organisms	L-Aspartate:2-oxoglutarate aminotransferase	(1) 2-Oxoglutarate_c0[c1] + (1) L-Aspartate_c0[c1] <-> (1) L-Glutamate_c0[c1] + (1) Oxaloacetate_c0[c1]	-0.0499572	No
rxn03638	Amino sugar and nucleotide sugar metabolism	Acetyl-CoA:D-glucosamine-1-phosphate N-acetyltransferase	(1) Acetyl-CoA_c0[c1] + (1) D-Glucosamine1-phosphate_c0[c1] -> (1) CoA_c0[c1] + (1) H+_c0[c1] + (1) N-Acetyl-D-glucosamine1-phosphate_c0[c1]	0.00254813	No
rxn01485	Amino sugar and nucleotide sugar metabolism	D-Glucosamine 1-phosphate 1,6-phosphomutase	(1) D-Glucosamine1-phosphate_c0[c1] <-> (1) D-Glucosamine phosphate_c0[c1]	-0.00254813	No
rxn00293	Amino sugar and nucleotide sugar metabolism	UTP:N-acetyl-alpha-D-glucosamine-1-phosphate uridylyltransferase	(1) UTP_c0[c1] + (1) N-Acetyl-D-glucosamine1-phosphate_c0[c1] <-> (1) PPi_c0[c1] + (1) UDP-N-acetylglucosamine_c0[c1]	0.00254813	No
rxn02285	Amino sugar and nucleotide sugar metabolism|Peptidoglycan biosynthesis	UDP-N-acetylmuramate:NADP+ oxidoreductase	(1) NADP_c0[c1] + (1) UDP-MurNAc_c0[c1] <-> (1) NADPH_c0[c1] + (1) H+_c0[c1] + (1) UDP-N-acetylglucosamine enolpyruvate_c0[c1]	-0.00063703	No
rxn00461	Amino sugar and nucleotide sugar metabolism|Peptidoglycan biosynthesis	Phosphoenolpyruvate:UDP-N-acetyl-D-glucosamine 1-carboxyvinyl-transferase	(1) UDP-N-acetylglucosamine_c0[c1] + (1) Phosphoenolpyruvate_c0[c1] <-> (1) Phosphate_c0[c1] + (1) UDP-N-acetylglucosamine enolpyruvate_c0[c1]	0.000637032	No
rxn01636	Arginine and proline metabolism	N2-Acetyl-L-ornithine:L-glutamate N-acetyltransferase	(1) L-Glutamate_c0[c1] + (1) N-Acetylornithine_c0[c1] <-> (1) Ornithine_c0[c1] + (1) N-Acetyl-L-glutamate_c0[c1]	0.0087284	No
rxn01637	Arginine and proline metabolism	N2-Acetyl-L-ornithine:2-oxoglutarate aminotransferase	(1) 2-Oxoglutarate_c0[c1] + (1) N-Acetylornithine_c0[c1] <-> (1) L-Glutamate_c0[c1] + (1) 2-Acetamido-5-oxopentanoate_c0[c1]	-0.0087284	No
rxn01917	Arginine and proline metabolism	ATP:N-acetyl-L-glutamate 5-phosphotransferase	(1) ATP_c0[c1] + (1) N-Acetyl-L-glutamate_c0[c1] <-> (1) ADP_c0[c1] + (1) n-acetylglutamyl-phosphate_c0[c1]	0.0087284	No
rxn02465	Arginine and proline metabolism	N-acetyl-L-glutamate-5-semialdehyde:NADP+ 5-oxidoreductase (phosphrylating)	(1) NADP_c0[c1] + (1) Phosphate_c0[c1] + (1) 2-Acetamido-5-oxopentanoate_c0[c1] <- (1) NADPH_c0[c1] + (1) H+_c0[c1] + (1) n-acetylglutamyl-phosphate_c0[c1]	-0.0087284	No
rxn01019	Arginine and proline metabolism	Carbamoyl-phosphate:L-ornithine carbamoyltransferase	(1) Ornithine_c0[c1] + (1) Carbamoylphosphate_c0[c1] -> (1) Phosphate_c0[c1] + (1) H+_c0[c1] + (1) Citrulline_c0[c1]	0.00628348	No
rxn00470	Arginine and proline metabolism|Glutathione metabolism	L-ornithine carboxy-lyase (putrescine-forming)	(1) Ornithine_c0[c1] + (1) H+_c0[c1] -> (1) CO2_c0[c1] + (1) Putrescine_c0[c1]	0.00244492	No

rxn00346	beta-Alanine metabolism|Pantothenate and CoA biosynthesis	L-aspartate 1-carboxy-lyase (beta-alanine-forming)	(1) L-Aspartate_c0[c1] + (1) H+_c0[c1] -> (1) CO2_c0[c1] + (1) beta-Alanine_c0[c1]	0.000157737	No
rxn01791	beta-Alanine metabolism|Pantothenate and CoA biosynthesis	(R)-Pantoate:beta-alanine ligase (AMP-forming)	(1) ATP_c0[c1] + (1) beta-Alanine_c0[c1] + (1) Pantoate_c0[c1] -> (1) PPi_c0[c1] + (1) AMP_c0[c1] + (2) H+_c0[c1] + (1) PAN_c0[c1]	0.000157737	No
rxn02185	Butanoate metabolism	2-Acetolactate pyruvate-lyase (carboxylating)	(1) TPP_c0[c1] + (1) ALCTT_c0[c1] <-> (1) Pyruvate_c0[c1] + (1) 2-Hydroxyethyl-ThPP_c0[c1]	-0.00912928	No
rxn00548	Carbon fixation in photosynthetic organisms	D-fructose-6-phosphate D-erythrose-4-phosphate-lyase (adding phosphate; acetyl-phosphate-forming)	(1) Phosphate_c0[c1] + (1) D-fructose-6-phosphate_c0[c1] -> (1) H2O_c0[c1] + (1) Acetylphosphate_c0[c1] + (1) D-Erythrose4-phosphate_c0[c1]	0.0363254	No
rxn00285	Citrate cycle (TCA cycle)|Propanoate metabolism|Carbon fixation pathways in prokaryotes	Succinate:CoA ligase (ADP-forming)	(1) ATP_c0[c1] + (1) CoA_c0[c1] + (1) Succinate_c0[c1] <-> (1) ADP_c0[c1] + (1) Phosphate_c0[c1] + (1) Succinyl-CoA_c0[c1]	0.106119	No
rxn00126	Cysteine and methionine metabolism	ATP:L-methionine S-adenosyltransferase	(1) H2O_c0[c1] + (1) ATP_c0[c1] + (1) L-Methionine_c0[c1] -> (1) Phosphate_c0[c1] + (1) PPi_c0[c1] + (1) S-Adenosyl-L-methionine_c0[c1] + (1) H+_c0[c1]	0.000552079	No
rxn00952	Cysteine and methionine metabolism	O-acetyl-L-homoserine:hydrogen sulfide S-(3-amino-3-carboxypropyl)transferase	(1) H2S_c0[c1] + (1) O-Acetyl-L-homoserine_c0[c1] -> (1) Acetate_c0[c1] + (1) Homocysteine_c0[c1]	0.00333403	No
rxn00141	Cysteine and methionine metabolism	S-Adenosyl-L-homocysteine hydrolase	(1) H2O_c0[c1] + (1) S-Adenosyl-homocysteine_c0[c1] <-> (1) Homocysteine_c0[c1] + (1) Adenosine_c0[c1]	0.000473211	No
rxn00127	Cysteine and methionine metabolism|Arginine and proline metabolism	S-adenosyl-L-methionine carboxy-lyase [(5-deoxy-5-adenosyl)(3-aminopropyl)methylsulfonium-forming]	(1) S-Adenosyl-L-methionine_c0[c1] + (1) H+_c0[c1] -> (1) CO2_c0[c1] + (1) S-Adenosylmethioninamine_c0[c1]	0.00131447	No
rxn01406	Cysteine and methionine metabolism|Arginine and proline metabolism|Glutathione metabolism	S-adenosylmethioninamine:putrescine 3-aminopropyltransferase	(1) Putrescine_c0[c1] + (1) S-Adenosylmethioninamine_c0[c1] -> (1) H+_c0[c1] + (1) 5-Methylthioadenosine_c0[c1] + (1) Spermidine_c0[c1]	0.00131447	No
rxn00693	Cysteine and methionine metabolism|One carbon pool by folate	5-Methyltetrahydrofolate:L-homocysteine S-methyltransferase	(1) Homocysteine_c0[c1] + (1) 5-Methyltetrahydrofolate_c0[c1] <-> (1) L-Methionine_c0[c1] + (1) Tetrahydrofolate_c0[c1]	0.00380724	No
rxn00423	Cysteine and methionine metabolism|Sulfur metabolism	acetyl-CoA:L-serine O-acetyltransferase	(1) Acetyl-CoA_c0[c1] + (1) L-Serine_c0[c1] -> (1) CoA_c0[c1] + (1) O-Acetyl-L-serine_c0[c1]	0.00225496	No
rxn00649	Cysteine and methionine metabolism|Sulfur metabolism	O3-acetyl-L-serine:hydrogen-sulfide 2-amino-2-carboxyethyltransferase	(1) H2S_c0[c1] + (1) O-Acetyl-L-serine_c0[c1] -> (1) Acetate_c0[c1] + (1) L-Cysteine_c0[c1]	0.00225496	No
rxn01303	Cysteine and methionine metabolism|Sulfur metabolism	Acetyl-CoA:L-homoserine O-acetyltransferase	(1) Acetyl-CoA_c0[c1] + (1) L-Homoserine_c0[c1] -> (1) CoA_c0[c1] + (1) O-Acetyl-L-homoserine_c0[c1]	0.00333403	No
rxn00849	D-Alanine metabolism	D-Alanine:2-oxoglutarate aminotransferase	(1) 2-Oxoglutarate_c0[c1] + (1) D-Alanine_c0[c1] <-> (1) Pyruvate_c0[c1] + (1) D-Glutamate_c0[c1]	-0.00097385	No
rxn00193	D-Glutamine and D-glutamate metabolism	glutamate racemase	(1) L-Glutamate_c0[c1] <-> (1) D-Glutamate_c0[c1]	0.00161088	No

rxn02286	D-Glutamine and D-glutamate metabolism|Peptidoglycan biosynthesis	UDP-N-acetylmuramate:L-alanine ligase (ADP-forming)	(1) ATP_c0[c1] + (1) L-Alanine_c0[c1] + (1) UDP-MurNAc_c0[c1] -> (1) ADP_c0[c1] + (1) Phosphate_c0[c1] + (1) H+_c0[c1] + (1) UDP-N-acetylmuramoyl-L-alanine_c0[c1]	0.000637032	No
rxn02008	D-Glutamine and D-glutamate metabolism|Peptidoglycan biosynthesis	UDP-N-acetylmuramoyl-L-alanine:D-glutamate ligase(ADP-forming)	(1) ATP_c0[c1] + (1) D-Glutamate_c0[c1] + (1) UDP-N-acetylmuramoyl-L-alanine_c0[c1] -> (1) ADP_c0[c1] + (1) Phosphate_c0[c1] + (1) H+_c0[c1] + (1) UDP-N-acetylmuramoyl-L-alanyl-D-glutamate_c0[c1]	0.000637032	No
rxn05345	Fatty acid biosynthesis	dodecanoyl-[acyl-carrier-protein]:malonyl-[acyl-carrier-protein] C-acyltransferase (decarboxylating)	(1) Dodecanoyl-ACP_c0[c1] + (1) Malonyl-acyl-carrierprotein-_c0[c1] -> (1) CO2_c0[c1] + (1) 3-oxotetradecanoyl-acp_c0[c1] + (1) ACP_c0[c1]	0.00254813	No
rxn05465	Fatty acid biosynthesis	Malonyl-CoA:[acyl-carrier-protein] S-malonyltransferase	(1) H+_c0[c1] + (1) Malonyl-CoA_c0[c1] + (1) ACP_c0[c1] <-> (1) CoA_c0[c1] + (1) Malonyl-acyl-carrierprotein-_c0[c1]	0.0253992	No
rxn05342	Fatty acid biosynthesis	(3R)-3-Hydroxytetradecanoyl-[acyl-carrier-protein]:NADP+ oxidoreductase	(1) NADP_c0[c1] + (1) HMA_c0[c1] <-> (1) NADPH_c0[c1] + (1) 3-oxotetradecanoyl-acp_c0[c1]	-0.00254813	No
rxn00178	Fatty acid degradation|Synthesis and degradation of ketone bodies|Valine, leucine and isoleucine degradation|Lysine degradation|Benzoate degradation|Tryptophan metabolism|Pyruvate metabolism|Glyoxylate and dicarboxylate metabolism|Propanoate metabolism|Butanoate metabolism|Carbon fixation pathways in prokaryotes|Terpenoid backbone biosynthesis	Acetyl-CoA:acetyl-CoA C-acetyltransferase	(2) Acetyl-CoA_c0[c1] <-> (1) CoA_c0[c1] + (1) Acetoacetyl-CoA_c0[c1]	0.00216968	Yes
rxn03245	Fatty acid elongation|Fatty acid degradation	(S)-Hydroxydecanoyl-CoA hydro-lyase	(1) (S)-Hydroxydecanoyl-CoA_c0[c1] <-> (1) H2O_c0[c1] + (1) (2E)-Decenoyl-CoA_c0[c1]	0.00382219	No
rxn02911	Fatty acid elongation|Fatty acid degradation	(S)-3-Hydroxydodecanoyl-CoA hydro-lyase	(1) (S)-3-Hydroxydodecanoyl-CoA_c0[c1] <-> (1) H2O_c0[c1] + (1) (2E)-Dodecenoyl-CoA_c0[c1]	0.00382219	No
rxn06777	Fatty acid elongation|Fatty acid degradation	(S)-3-Hydroxytetradecanoyl-CoA:NAD+ oxidoreductase	(1) NAD_c0[c1] + (1) (S)-3-Hydroxytetradecanoyl-CoA_c0[c1] <-> (1) NADH_c0[c1] + (1) H+_c0[c1] + (1) 3-Oxotetradecanoyl-CoA_c0[c1]	-0.00382219	No
rxn03244	Fatty acid elongation|Fatty acid degradation	(S)-hydroxydecanoyl-CoA:NAD+ oxidoreductase	(1) NAD_c0[c1] + (1) (S)-Hydroxydecanoyl-CoA_c0[c1] <-> (1) NADH_c0[c1] + (1) H+_c0[c1] + (1) 3-Oxodecanoyl-CoA_c0[c1]	-0.00382219	No
rxn02680	Fatty acid elongation|Fatty acid degradation	Octanoyl-CoA:acetyl-CoA C-acyltransferase	(1) Acetyl-CoA_c0[c1] + (1) Octanoyl-CoA_c0[c1] <-> (1) CoA_c0[c1] + (1) 3-Oxodecanoyl-CoA_c0[c1]	0.00382219	Yes
rxn03243	Fatty acid elongation|Fatty acid degradation	Decanoyl-CoA:acetyl-CoA C-acyltransferase	(1) Acetyl-CoA_c0[c1] + (1) Decanoyl-CoA_c0[c1] <-> (1) CoA_c0[c1] + (1) 3-Oxododecanoyl-CoA_c0[c1]	0.00382219	Yes

rxn03242	Fatty acid elongation|Fatty acid degradation	(S)-3-hydroxydodecanoyl-CoA:NAD+ oxidoreductase	(1) NAD_c0[c1] + (1) (S)-3-Hydroxydodecanoyl-CoA_c0[c1] <-> (1) NADH_c0[c1] + (1) H+_c0[c1] + (1) 3-Oxododecanoyl-CoA_c0[c1]	-0.00382219	No
rxn03241	Fatty acid elongation|Fatty acid degradation	(S)-3-Hydroxytetradecanoyl-CoA hydro-lyase	(1) (S)-3-Hydroxytetradecanoyl-CoA_c0[c1] <-> (1) H2O_c0[c1] + (1) (2E)-Tetradecenoyl-CoA_c0[c1]	0.00382219	No
rxn06510	Fatty acid elongation|Fatty acid degradation	Lauroyl-CoA:acetyl-CoA C-acyltransferase	(1) Acetyl-CoA_c0[c1] + (1) Lauroyl-CoA_c0[c1] <-> (1) CoA_c0[c1] + (1) 3-Oxotetradecanoyl-CoA_c0[c1]	0.00382219	Yes
rxn02504	Folate biosynthesis	2-amino-4-hydroxy-6-(D-erythro-1,2,3-trihydroxypropyl)-7,8-dihydropteridine glycolaldehyde-lyase	(1) Dihydroneopterin_c0[c1] <-> (1) Glycolaldehyde_c0[c1] + (1) 6-hydroxymethyl dihydropterin_c0[c1]	0.000236605	No
rxn03167	Folate biosynthesis	2-Amino-4-hydroxy-6-(erythro-1,2,3-trihydroxypropyl) dihydropteridine triphosphate phosphohydrolase (alkaline optimum)	(3) H2O_c0[c1] + (1) 7,8-Dihydroneopterin 3'-triphosphate_c0[c1] -> (3) Phosphate_c0[c1] + (3) H+_c0[c1] + (1) Dihydroneopterin_c0[c1]	0.000236605	No
rxn02200	Folate biosynthesis	2-amino-4-hydroxy-6-hydroxymethyl-7,8-dihydropteridine:4-aminobenzoate 2-amino-4-hydroxydihydropteridine-6-methenyltransferase	(1) ABEE_c0[c1] + (1) 6-hydroxymethyl dihydropterin_c0[c1] <-> (1) H2O_c0[c1] + (1) Dihydropteroate_c0[c1]	0.000236605	No
rxn03841	Folate biosynthesis	4-amino-4-deoxychorismate pyruvate-lyase	(1) ADC_c0[c1] -> (1) Pyruvate_c0[c1] + (1) H+_c0[c1] + (1) ABEE_c0[c1]	0.000236605	No
rxn01257	Folate biosynthesis	chorismate:L-glutamine aminotransferase	(1) L-Glutamine_c0[c1] + (1) Chorismate_c0[c1] <-> (1) L-Glutamate_c0[c1] + (1) ADC_c0[c1]	0.000236605	No
rxn01603	Folate biosynthesis	7,8-dihydropteroate:L-glutamate ligase (ADP-forming)	(1) ATP_c0[c1] + (1) L-Glutamate_c0[c1] + (1) Dihydropteroate_c0[c1] -> (1) ADP_c0[c1] + (1) Phosphate_c0[c1] + (1) H+_c0[c1] + (1) Dihydrofolate_c0[c1]	0.000236605	No
rxn00650	Glutathione metabolism	L-cysteinylglycine dipeptidase	(1) H2O_c0[c1] + (1) Cys-Gly_c0[c1] <-> (1) Glycine_c0[c1] + (1) L-Cysteine_c0[c1]	-7.8868E-05	No
rxn00350	Glutathione metabolism	glutathione gamma-glutamylaminopeptidase	(1) H2O_c0[c1] + (1) GSH_c0[c1] <-> (1) L-Glutamate_c0[c1] + (1) Cys-Gly_c0[c1]	-7.8868E-05	No
rxn00615	Glycerolipid metabolism	ATP:glycerol 3-phosphotransferase	(1) ATP_c0[c1] + (1) Glycerol_c0[c1] <-> (1) ADP_c0[c1] + (1) H+_c0[c1] + (1) Glycerol-3-phosphate_c0[c1]	0.000813632	No
rxn00539	Glycerophospholipid metabolism	ethanolamine ammonia-lyase (acetaldehyde-forming)	(1) Aminoethanol_c0[c1] -> (1) NH3_c0[c1] + (1) Acetaldehyde_c0[c1]	0.00462695	No
rxn00611	Glycerophospholipid metabolism	sn-Glycerol-3-phosphate:NAD+ 2-oxidoreductase	(1) NAD_c0[c1] + (1) Glycerol-3-phosphate_c0[c1] <-> (1) NADH_c0[c1] + (1) H+_c0[c1] + (1) Glycerone-phosphate_c0[c1]	-0.00488179	No
rxn06493	Glycine, serine and threonine metabolism	dihydrolipoylprotein:NAD+ oxidoreductase	(1) NAD_c0[c1] + (1) Dihydrolipolprotein_c0[c1] <-> (1) NADH_c0[c1] + (1) H+_c0[c1] + (1) Lipoylprotein_c0[c1]	0.00210828	No
rxn01300	Glycine, serine and threonine metabolism	ATP:L-homoserine O-phosphotransferase	(1) ATP_c0[c1] + (1) L-Homoserine_c0[c1] <-> (1)	0.00690789	No

			ADP_c0[c1] + (1) H+_c0[c1] + (1) O-Phospho-L-homoserine_c0[c1]		
rxn01069	Glycine, serine and threonine metabolism	O-phospho-L-homoserine phosphate-lyase (adding water;L-threonine-forming)	(1) H2O_c0[c1] + (1) O-Phospho-L-homoserine_c0[c1] -> (1) Phosphate_c0[c1] + (1) L-Threonine_c0[c1]	0.00690789	No
rxn06377	Glycine, serine and threonine metabolism	glycine:lipoylprotein oxidoreductase (decarboxylating and acceptor-aminomethylating)	(1) Glycine_c0[c1] + (1) H+_c0[c1] + (1) Lipoylprotein_c0[c1] -> (1) CO2_c0[c1] + (1) S-Aminomethyldihydrolipoylprotein_c0[c1]	0.00210828	No
rxn00541	Glycine, serine and threonine metabolism	L-threonine acetaldehyde-lyase (glycine-forming)	(1) L-Threonine_c0[c1] <-> (1) Glycine_c0[c1] + (1) Acetaldehyde_c0[c1]	-0.00462695	No
rxn00692	Glycine, serine and threonine metabolism|Cyanoamino acid metabolism|Glyoxylate and dicarboxylate metabolism|One carbon pool by folate|Methane metabolism	5,10-Methylenetetrahydrofolate:glycine hydroxymethyltransferase	(1) H2O_c0[c1] + (1) Glycine_c0[c1] + (1) 5-10-Methylenetetrahydrofolate_c0[c1] <-> (1) L-Serine_c0[c1] + (1) Tetrahydrofolate_c0[c1]	-0.0195504	No
rxn00337	Glycine, serine and threonine metabolism|Cysteine and methionine metabolism|Lysine biosynthesis	ATP:L-aspartate 4-phosphotransferase	(1) ATP_c0[c1] + (1) L-Aspartate_c0[c1] <-> (1) ADP_c0[c1] + (1) 4-Phospho-L-aspartate_c0[c1]	0.0194637	No
rxn01301	Glycine, serine and threonine metabolism|Cysteine and methionine metabolism|Lysine biosynthesis	L-Homoserine:NAD+ oxidoreductase	(1) NAD_c0[c1] + (1) L-Homoserine_c0[c1] <-> (1) NADH_c0[c1] + (1) H+_c0[c1] + (1) L-Aspartate4-semialdehyde_c0[c1]	-0.0115564	No
rxn01643	Glycine, serine and threonine metabolism|Cysteine and methionine metabolism|Lysine biosynthesis	L-Aspartate-4-semialdehyde:NADP+ oxidoreductase (phosphorylating)	(1) NADP_c0[c1] + (1) Phosphate_c0[c1] + (1) L-Aspartate4-semialdehyde_c0[c1] <- (1) NADPH_c0[c1] + (1) H+_c0[c1] + (1) 4-Phospho-L-aspartate_c0[c1]	-0.0194637	No
rxn00420	Glycine, serine and threonine metabolism|Methane metabolism	O-phospho-L-serine phosphohydrolase	(1) H2O_c0[c1] + (1) phosphoserine_c0[c1] -> (1) Phosphate_c0[c1] + (1) L-Serine_c0[c1]	0.0283921	No
rxn01101	Glycine, serine and threonine metabolism|Methane metabolism	3-Phospho-D-glycerate:NAD+ 2-oxidoreductase	(1) NAD_c0[c1] + (1) 3-Phosphoglycerate_c0[c1] <-> (1) NADH_c0[c1] + (1) H+_c0[c1] + (1) 3-Phosphonooxypyruvate_c0[c1]	0.0283921	No
rxn02914	Glycine, serine and threonine metabolism|Methane metabolism	3-Phosphoserine:2-oxoglutarate aminotransferase	(1) 2-Oxoglutarate_c0[c1] + (1) phosphoserine_c0[c1] <-> (1) L-Glutamate_c0[c1] + (1) 3-Phosphonooxypyruvate_c0[c1]	-0.0283921	No
rxn06600	Glycine, serine and threonine metabolism|One carbon pool by folate	S-aminomethyldihydrolipoylprotein:(6S)-tetrahydrofolate aminomethyltransferase (ammonia-forming)	(1) Tetrahydrofolate_c0[c1] + (1) S-Aminomethyldihydrolipoylprotein_c0[c1] -> (1) NH3_c0[c1] + (1) 5-10-Methylenetetrahydrofolate_c0[c1] + (1) Dihydrolipolprotein_c0[c1]	0.00210828	No
rxn01964	Glycine, serine and threonine metabolism|Phenylalanine, tyrosine and tryptophan biosynthesis	L-serine hydro-lyase [adding 1-C-(indol-3-yl)glycerol 3-phosphate; L-tryptophan and glyceraldehyde-3-phosphate-forming]	(1) L-Serine_c0[c1] + (1) Indoleglycerol phosphate_c0[c1] -> (1) H2O_c0[c1] + (1) L-Tryptophan_c0[c1] + (1) Glyceraldehyde3-phosphate_c0[c1]	0.00120225	No
rxn00737	Glycine, serine and threonine metabolism|Valine, leucine and isoleucine biosynthesis	L-threonine ammonia-lyase (2-oxobutanoate-forming)	(1) L-Threonine_c0[c1] -> (1) NH3_c0[c1] + (1) 2-Oxobutyrate_c0[c1]	0.00615872	No
rxn00781	Glycolysis / Gluconeogenesis|Carbon	D-glyceraldehyde-3-phosphate:NAD+	(1) NAD_c0[c1] + (1) Phosphate_c0[c1] + (1) Glyceraldehyde3-	0.0907853	No

	fixation in photosynthetic organisms	oxidoreductase (phosphorylating)	phosphate_c0[c1] <-> (1) NADH_c0[c1] + (1) H+_c0[c1] + (1) 1,3-Bisphospho-D-glycerate_c0[c1]		
rxn01100	Glycolysis / Gluconeogenesis|Carbon fixation in photosynthetic organisms	ATP:3-phospho-D-glycerate 1-phosphotransferase	(1) ATP_c0[c1] + (1) 3-Phosphoglycerate_c0[c1] <-> (1) ADP_c0[c1] + (1) 1,3-Bisphospho-D-glycerate_c0[c1]	-0.0907853	No
rxn00011	Glycolysis / Gluconeogenesis|Citrate cycle (TCA cycle)|Valine, leucine and isoleucine biosynthesis|Pyruvate metabolism|Butanoate metabolism	pyruvate:thiamin diphosphate acetaldehydetransferase (decarboxylating)	(1) CO2_c0[c1] + (1) 2-Hydroxyethyl-ThPP_c0[c1] <- (1) Pyruvate_c0[c1] + (1) TPP_c0[c1] + (1) H+_c0[c1]	-0.015288	No
rxn01106	Glycolysis / Gluconeogenesis|Glycine, serine and threonine metabolism|Methane metabolism	2-Phospho-D-glycerate 2,3-phosphomutase	(1) 2-Phospho-D-glycerate_c0[c1] <-> (1) 3-Phosphoglycerate_c0[c1]	-0.0623932	No
rxn00459	Glycolysis / Gluconeogenesis|Methane metabolism	2-phospho-D-glycerate hydro-lyase (phosphoenolpyruvate-forming)	(1) 2-Phospho-D-glycerate_c0[c1] <-> (1) H2O_c0[c1] + (1) Phosphoenolpyruvate_c0[c1]	0.0623932	No
rxn00148	Glycolysis / Gluconeogenesis|Purine metabolism|Pyruvate metabolism|Carbon fixation in photosynthetic organisms	ATP:pyruvate 2-O-phosphotransferase	(1) ATP_c0[c1] + (1) Pyruvate_c0[c1] <-> (1) ADP_c0[c1] + (1) Phosphoenolpyruvate_c0[c1] + (1) H+_c0[c1]	0.117691	No
rxn00499	Glycolysis / Gluconeogenesis|Pyruvate metabolism	(S)-Lactate:NAD+ oxidoreductase	(1) NAD_c0[c1] + (1) L-Lactate_c0[c1] <-> (1) NADH_c0[c1] + (1) Pyruvate_c0[c1] + (1) H+_c0[c1]	0.18635	No
rxn00333	Glyoxylate and dicarboxylate metabolism	Glycolate:oxygen 2-oxidoreductase	(1) O2_c0[c1] + (1) Glycolate_c0[c1] -> (1) H2O2_c0[c1] + (1) Glyoxalate_c0[c1]	0.0116243	No
rxn12822	Glyoxylate and dicarboxylate metabolism|Nitrogen metabolism	L-glutamate:ferredoxin oxidoreductase (transaminating)	(2) L-Glutamate_c0[c1] + (2) Oxidizedferredoxin_c0[c1] <-> (1) 2-Oxoglutarate_c0[c1] + (1) L-Glutamine_c0[c1] + (2) H+_c0[c1] + (2) Reducedferredoxin_c0[c1]	0.0464886	No
rxn02320	Histidine metabolism	5-Amino-2-oxopentanoate:2-oxoglutarate aminotransferase	(1) 2-Oxoglutarate_c0[c1] + (1) L-histidinol-phosphate_c0[c1] <-> (1) L-Glutamate_c0[c1] + (1) imidazole acetol-phosphate_c0[c1]	-0.00201888	No
rxn00789	Histidine metabolism	1-(5-phospho-D-ribosyl)-ATP:diphosphate phospho-alpha-D-ribosyl-transferase	(1) PPi_c0[c1] + (1) H+_c0[c1] + (1) Phosphoribosyl-ATP_c0[c1] <- (1) ATP_c0[c1] + (1) PRPP_c0[c1]	-0.00201888	No
rxn02160	Histidine metabolism	L-Histidinol-phosphate phosphohydrolase	(1) H2O_c0[c1] + (1) L-histidinol-phosphate_c0[c1] -> (1) Phosphate_c0[c1] + (1) L-Histidinol_c0[c1]	0.00201888	No
rxn02159	Histidine metabolism	L-Histidinol:NAD+ oxidoreductase	(1) NAD_c0[c1] + (1) L-Histidinol_c0[c1] <-> (1) NADH_c0[c1] + (1) H+_c0[c1] + (1) L-Histidinal_c0[c1]	0.00201888	No
rxn02835	Histidine metabolism	1-(5-phospho-D-ribosyl)-AMP 1,6-hydrolase	(1) H2O_c0[c1] + (1) Phosphoribosyl-AMP_c0[c1] <-> (1) phosphoribosylformiminoaicar-phosphate_c0[c1]	0.00201888	No
rxn03175	Histidine metabolism	N-(5'-Phospho-D-ribosylformimino)-5-amino-1- (5''-phospho-D-ribosyl)-4-	(1) H+_c0[c1] + (1) phosphoribosylformiminoaicar-phosphate_c0[c1] <-> (1)	0.00201888	No

		imidazolecarboxamide ketol-isomerase	phosphoribulosylformimino-AICAR-phosphate_c0[c1]		
rxn03135	Histidine metabolism	R04558	(1) L-Glutamate_c0[c1] + (2) H+_c0[c1] + (1) D-erythro-imidazol-glycerol-phosphate_c0[c1] + (1) AICAR_c0[c1] <- (1) L-Glutamine_c0[c1] + (1) phosphoribulosylformimino-AICAR-phosphate_c0[c1]	-0.00201888	No
rxn00863	Histidine metabolism	L-histidinal:NAD+ oxidoreductase	(1) H2O_c0[c1] + (1) NAD_c0[c1] + (1) L-Histidinal_c0[c1] -> (1) NADH_c0[c1] + (2) H+_c0[c1] + (1) L-Histidine_c0[c1]	0.00201888	No
rxn02834	Histidine metabolism	Phosphoribosyl-ATP pyrophosphohydrolase	(1) H2O_c0[c1] + (1) Phosphoribosyl-ATP_c0[c1] -> (1) PPi_c0[c1] + (2) H+_c0[c1] + (1) Phosphoribosyl-AMP_c0[c1]	0.00201888	No
rxn02473	Histidine metabolism	D-erythro-1-(Imidazol-4-yl)glycerol 3-phosphate hydro-lyase	(1) D-erythro-imidazol-glycerol-phosphate_c0[c1] -> (1) H2O_c0[c1] + (1) imidazole acetol-phosphate_c0[c1]	0.00201888	No
rxn03181	Lipopolysaccharide biosynthesis	ATP:2,3,2',3'-tetrakis(3-hydroxytetradecanoyl)-D-glucosaminyl-beta-D-1,6-glucosaminyl-alpha-phosphate 4-O'-phosphotransferase	(1) ATP_c0[c1] + (1) Lipid A disaccharide_c0[c1] <-> (1) ADP_c0[c1] + (1) H+_c0[c1] + (1) Lipid IV(A)_c0[c1]	0.000637032	No
rxn02331	Lipopolysaccharide biosynthesis	phosphoenolpyruvate:D-arabinose-5-phosphate C-(1-carboxyvinyl)transferase (phosphate-hydrolysing, 2-carboxy-2-oxoethyl-forming)	(1) Phosphate_c0[c1] + (1) 3-Deoxy-D-manno-octulosonate8-phosphate_c0[c1] <- (1) H2O_c0[c1] + (1) Phosphoenolpyruvate_c0[c1] + (1) D-Arabinose5-phosphate_c0[c1]	-0.00191109	No
rxn02404	Lipopolysaccharide biosynthesis	3-Deoxy-D-manno-octulosonate-8-phosphate 8-phosphohydrolase	(1) H2O_c0[c1] + (1) 3-Deoxy-D-manno-octulosonate8-phosphate_c0[c1] -> (1) Phosphate_c0[c1] + (1) KDO_c0[c1]	0.00191109	No
rxn03919	Lipopolysaccharide biosynthesis	D-glycero-beta-D-manno-heptose 1,7-bisphosphate 7-phosphohydrolase	(1) H2O_c0[c1] + (1) D-Glycero-D-manno-heptose1-7-bisphosphate_c0[c1] -> (1) Phosphate_c0[c1] + (1) D-Glycero-D-manno-heptose1-phosphate_c0[c1]	0.00254813	No
rxn03159	Lipopolysaccharide biosynthesis	UDP-2,3-bis(3-hydroxytetradecanoyl)glucosamine:2,3-bis-(3-hydroxytetradecanoyl)-alpha-D-glucosaminyl-1-phosphate 2,3-bis(3-hydroxytetradecanoyl)-glucosaminyltransferase	(1) UDP-2,3-bis(3-hydroxytetradecanoyl)glucosamine_c0[c1] + (1) Lipid X_c0[c1] <-> (1) UDP_c0[c1] + (1) Lipid A disaccharide_c0[c1]	0.000637032	No
rxn06723	Lipopolysaccharide biosynthesis	(3R)-3-hydroxymyristoyl-[acyl-carrier protein]:UDP-3-O-[(3R)-3-hydroxymyristoyl]-alpha-D-glucosamine N-acetyltransferase	(1) UDP-2,3-bis(3-hydroxytetradecanoyl)glucosamine_c0[c1] + (1) ACP_c0[c1] <- (1) UDP-3-O-(beta-hydroxymyristoyl)-D-glucosamine_c0[c1] + (1) HMA_c0[c1]	-0.00127406	No
rxn03146	Lipopolysaccharide biosynthesis	UDP-3-O-[(3R)-3-hydroxymyristoyl]-N-acetylglucosamine amidohydrolase	(1) H2O_c0[c1] + (1) UDP-3-O-(beta-hydroxymyristoyl)-N-acetylglucosamine_c0[c1] <-> (1) Acetate_c0[c1] + (1) UDP-3-O-(beta-hydroxymyristoyl)-D-glucosamine_c0[c1]	0.00127406	No

rxn02405	Lipopolysaccharide biosynthesis	CTP:3-deoxy-D-manno-octulosonate cytidylyltransferase	(1) CTP_c0[c1] + (1) KDO_c0[c1] -> (1) PPi_c0[c1] + (1) H+_c0[c1] + (1) CMP-KDO_c0[c1]	0.00191109	No
rxn06865	Lipopolysaccharide biosynthesis	R05146	(1) Lauroyl-KDO2-lipid IV(A)_c0[c1] + (1) ACP_c0[c1] <- (1) kdo2-lipid iva_c0[c1] + (1) Dodecanoyl-ACP_c0[c1]	-0.00063703	No
rxn03439	Lipopolysaccharide biosynthesis	CMP-3-deoxy-D-manno-oct-2-ulosonate:(KDO)-lipid IVA 3-deoxy-D-manno-oct-2-ulosonate transferase	(1) CMP-KDO_c0[c1] + (1) KDO-lipid IV(A)_c0[c1] <-> (1) CMP_c0[c1] + (1) H+_c0[c1] + (1) kdo2-lipid iva_c0[c1]	0.000637032	No
rxn06729	Lipopolysaccharide biosynthesis	(R)-3-Hydroxytetradecanoly-[acyl-carrier-protein]:UDP-N-acetyl-glucosamine 3-O-(3-hydroxytetradecanoyl)transferase	(1) UDP-N-acetylglucosamine_c0[c1] + (1) H+_c0[c1] + (1) HMA_c0[c1] -> (1) UDP-3-O-(beta-hydroxymyristoyl)-N-acetylglucosamine_c0[c1] + (1) ACP_c0[c1]	0.00127406	No
rxn03130	Lipopolysaccharide biosynthesis	UDP-2,3-bis[(3R)-3-hydroxymyristoyl]-alpha-D-glucosamine 2,3-bis[(3R)-3-hydroxymyristoyl]-beta-D-glucosaminyl 1-phosphate phosphohydrolase	(1) H2O_c0[c1] + (1) UDP-2,3-bis(3-hydroxytetradecanoyl)glucosamine_c0[c1] -> (2) H+_c0[c1] + (1) UMP_c0[c1] + (1) Lipid X_c0[c1]	0.000637032	No
rxn03182	Lipopolysaccharide biosynthesis	CMP-3-deoxy-D-manno-oct-2-ulosonate:lipid IVA 3-deoxy-D-manno-oct-2-ulosonate transferase	(1) CMP-KDO_c0[c1] + (1) Lipid IV(A)_c0[c1] <-> (1) CMP_c0[c1] + (1) H+_c0[c1] + (1) KDO-lipid IV(A)_c0[c1]	0.000637032	No
rxn00313	Lysine biosynthesis	meso-2,6-diaminoheptanedioate carboxy-lyase (L-lysine-forming)	(1) H+_c0[c1] + (1) meso-2,6-Diaminopimelate_c0[c1] -> (1) CO2_c0[c1] + (1) L-Lysine_c0[c1]	0.00727024	No
rxn02011	Lysine biosynthesis|Peptidoglycan biosynthesis	UDP-N-acetylmuramoyl-L-alanyl-D-glutamate:(L)-meso-2,6-diaminoheptanedioate gamma-ligase (ADP-forming)	(1) ATP_c0[c1] + (1) meso-2,6-Diaminopimelate_c0[c1] + (1) UDP-N-acetylmuramoyl-L-alanyl-D-glutamate_c0[c1] -> (1) ADP_c0[c1] + (1) Phosphate_c0[c1] + (1) H+_c0[c1] + (1) UDP-N-acetylmuramoyl-L-alanyl-D-gamma-glutamyl-meso-2-6-diaminopimelate_c0[c1]	0.000637032	No
rxn00786	Methane metabolism|Carbon fixation in photosynthetic organisms	D-fructose-1,6-bisphosphate D-glyceraldehyde-3-phosphate-lyase (glycerone-phosphate-forming)	(1) D-fructose-1,6-bisphosphate_c0[c1] <-> (1) Glycerone-phosphate_c0[c1] + (1) Glyceraldehyde3-phosphate_c0[c1]	0.00503953	No
rxn02988	Nicotinate and nicotinamide metabolism	glycerone phosphate:iminosuccinate alkyltransferase (cyclizing)	(2) H2O_c0[c1] + (1) Phosphate_c0[c1] + (1) Quinolinate_c0[c1] <- (1) Glycerone-phosphate_c0[c1] + (1) Iminoaspartate_c0[c1]	-0.00015774	No
rxn00190	Nicotinate and nicotinamide metabolism	Deamido-NAD+:L-glutamine amido-ligase (AMP-forming)	(1) H2O_c0[c1] + (1) ATP_c0[c1] + (1) L-Glutamine_c0[c1] + (1) Deamido-NAD_c0[c1] -> (1) NAD_c0[c1] + (1) PPi_c0[c1] + (1) AMP_c0[c1] + (1) L-Glutamate_c0[c1] + (2) H+_c0[c1]	0.000157737	No
rxn02402	Nicotinate and nicotinamide metabolism	Nicotinate-nucleotide:pyrophosphate phosphoribosyltransferase (carboxylating)	(1) CO2_c0[c1] + (1) PPi_c0[c1] + (1) Nicotinate ribonucleotide_c0[c1] <- (1) H+_c0[c1] + (1) PRPP_c0[c1] + (1) Quinolinate_c0[c1]	-0.00015774	No

rxn02155	Nicotinate and nicotinamide metabolism	ATP:nicotinamide-nucleotide adenylyltransferase	(1) ATP_c0[c1] + (1) Nicotinate ribonucleotide_c0[c1] <-> (1) PPi_c0[c1] + (1) Deamido-NAD_c0[c1]	0.000157737	No
rxn00083	Nicotinate and nicotinamide metabolism	NADPH:NAD+ oxidoreductase	(1) NAD_c0[c1] + (1) NADPH_c0[c1] <-> (1) NADH_c0[c1] + (1) NADP_c0[c1]	-0.00554611	No
rxn01265	Nicotinate and nicotinamide metabolism	Nicotinate D-ribonucleotide:diphosphate phosphoribosyltransferase	(1) PPi_c0[c1] + (1) Nicotinate ribonucleotide_c0[c1] <-> (1) PRPP_c0[c1] + (1) Niacin_c0[c1]	-7.8868E-05	No
rxn00077	Nicotinate and nicotinamide metabolism	ATP:NAD+ 2'-phosphotransferase	(1) ATP_c0[c1] + (1) NAD_c0[c1] <-> (1) NADP_c0[c1] + (1) ADP_c0[c1] + (1) H+_c0[c1]	7.88684E-05	No
rxn00338	Nicotinate and nicotinamide metabolism	L-aspartate:oxygen oxidoreductase	(1) O2_c0[c1] + (1) L-Aspartate_c0[c1] -> (1) H2O2_c0[c1] + (1) H+_c0[c1] + (1) Iminoaspartate_c0[c1]	0.000157737	No
rxn00102	Nitrogen metabolism	carbonate hydro-lyase (carbon-dioxide-forming)	(1) H+_c0[c1] + (1) H2CO3_c0[c1] <-> (1) H2O_c0[c1] + (1) CO2_c0[c1]	-0.057911	No
rxn00907	One carbon pool by folate|Carbon fixation pathways in prokaryotes	5,10-methylenetetrahydrofolate:NADP+ oxidoreductase	(1) NADP_c0[c1] + (1) 5-10-Methylenetetrahydrofolate_c0[c1] <-> (1) NADPH_c0[c1] + (1) 5-10-Methenyltetrahydrofolate_c0[c1]	0.017344	No
rxn01211	One carbon pool by folate|Carbon fixation pathways in prokaryotes	5,10-Methenyltetrahydrofolate 5-hydrolase (decyclizing)	(1) H2O_c0[c1] + (1) 5-10-Methenyltetrahydrofolate_c0[c1] <-> (1) H+_c0[c1] + (1) 10-Formyltetrahydrofolate_c0[c1]	0.017344	No
rxn00686	One carbon pool by folate|Folate biosynthesis	5,6,7,8-tetrahydrofolate:NADP+ oxidoreductase	(1) NADP_c0[c1] + (1) Tetrahydrofolate_c0[c1] <-> (1) NADPH_c0[c1] + (1) H+_c0[c1] + (1) Dihydrofolate_c0[c1]	-0.00050738	No
rxn04954	One carbon pool by folate|Methane metabolism|Carbon fixation pathways in prokaryotes	5-methyltetrahydrofolate:NAD+ oxidoreductase	(1) NAD_c0[c1] + (1) 5-Methyltetrahydrofolate_c0[c1] <-> (1) NADH_c0[c1] + (1) H+_c0[c1] + (1) 5-10-Methylenetetrahydrofolate_c0[c1]	-0.00388611	No
rxn00898	Pantothenate and CoA biosynthesis	2,3-Dihydroxy-3-methylbutanoate hydro-lyase	(1) 2,3-Dihydroxy-isovalerate_c0[c1] -> (1) H2O_c0[c1] + (1) 3-Methyl-2-oxobutanoate_c0[c1]	0.00912928	No
rxn12512	Pantothenate and CoA biosynthesis	(R)-4'-Phosphopantothenate:L-cysteine ligase	(1) ATP_c0[c1] + (1) L-Cysteine_c0[c1] + (1) 4-phosphopantothenate_c0[c1] -> (1) PPi_c0[c1] + (1) AMP_c0[c1] + (2) H+_c0[c1] + (1) (R)-4'-Phosphopantothenoyl-L-cysteine_c0[c1]	0.000157737	No
rxn00100	Pantothenate and CoA biosynthesis	ATP:dephospho-CoA 3'-phosphotransferase	(1) ATP_c0[c1] + (1) Dephospho-CoA_c0[c1] -> (1) ADP_c0[c1] + (1) CoA_c0[c1] + (1) H+_c0[c1]	7.88684E-05	No
rxn02175	Pantothenate and CoA biosynthesis	ATP:pantetheine-4'-phosphate adenylyltransferase	(1) ATP_c0[c1] + (1) Phosphopantetheine_c0[c1] <-> (1) PPi_c0[c1] + (1) Dephospho-CoA_c0[c1]	7.88684E-05	No
rxn06023	Pantothenate and CoA biosynthesis	CoA:apo-[acyl-carrier-protein] pantetheinephosphotransferase	(1) CoA_c0[c1] + (1) apo-ACP_c0[c1] <-> (1) Adenosine 3-5-bisphosphate_c0[c1] + (1) ACP_c0[c1]	7.88684E-05	No
rxn12510	Pantothenate and CoA biosynthesis	ATP:pantothenate 4'-phosphotransferase	(1) ATP_c0[c1] + (1) PAN_c0[c1] <-> (1) ADP_c0[c1] + (1) H+_c0[c1] + (1) 4-phosphopantothenate_c0[c1]	0.000157737	No
rxn01790	Pantothenate and CoA biosynthesis	(R)-Pantoate:NADP+ 2-oxidoreductase	(1) NADP_c0[c1] + (1) Pantoate_c0[c1] <-> (1) NADPH_c0[c1] + (1) H+_c0[c1] + (1) 2-Dehydropantoate_c0[c1]	-0.00015774	No

rxn00912	Pantothenate and CoA biosynthesis	5,10-Methylenetetrahydrofolate: 3-methyl-2-oxobutanoate hydroxymethyltransferase	(1) H2O_c0[c1] + (1) 3-Methyl-2-oxobutanoate_c0[c1] + (1) 5-10-Methylenetetrahydrofolate_c0[c1] <-> (1) Tetrahydrofolate_c0[c1] + (1) 2-Dehydropantoate_c0[c1]	0.000157737	No
rxn02341	Pantothenate and CoA biosynthesis	N-[(R)-4'-Phosphopantothenoyl]-L-cysteine carboxy-lyase	(1) H+_c0[c1] + (1) (R)-4'-Phosphopantothenoyl-L-cysteine_c0[c1] -> (1) CO2_c0[c1] + (1) Phosphopantetheine_c0[c1]	0.000157737	No
rxn02186	Pantothenate and CoA biosynthesis	2,3-Dihydroxy-3-methylbutanoate:NADP+ oxidoreductase (isomerizing)	(1) NADPH_c0[c1] + (1) H+_c0[c1] + (1) ALCTT_c0[c1] <-> (1) NADP_c0[c1] + (1) 2,3-Dihydroxy-isovalerate_c0[c1]	0.00912928	No
rxn00213	Pentose and glucuronate interconversions|Galactose metabolism|Starch and sucrose metabolism|Amino sugar and nucleotide sugar metabolism	UTP:alpha-D-glucose-1-phosphate uridylyltransferase	(1) UTP_c0[c1] + (1) Glucose-1-phosphate_c0[c1] <-> (1) PPi_c0[c1] + (1) UDP-glucose_c0[c1]	-0.00063703	No
rxn00772	Pentose phosphate pathway	ATP:D-ribose 5-phosphotransferase	(1) ATP_c0[c1] + (1) D-Ribose_c0[c1] <-> (1) ADP_c0[c1] + (1) H+_c0[c1] + (1) ribose-5-phosphate_c0[c1]	0.000473211	No
rxn01477	Pentose phosphate pathway	6-Phospho-D-gluconate hydro-lyase(2-dehydro-3-deoxy-6-phospho-D-gluconate-forming)	(1) 6-Phospho-D-gluconate_c0[c1] -> (1) H2O_c0[c1] + (1) 2-Keto-3-deoxy-6-phosphogluconate_c0[c1]	0.0457443	No
rxn01476	Pentose phosphate pathway	6-Phospho-D-glucono-1,5-lactone lactonohydrolase	(1) H2O_c0[c1] + (1) 6-phospho-D-glucono-1-5-lactone_c0[c1] -> (1) H+_c0[c1] + (1) 6-Phospho-D-gluconate_c0[c1]	0.0457443	No
rxn01333	Pentose phosphate pathway	sedoheptulose-7-phosphate:D-glyceraldehyde-3-phosphate glyceronetransferase	(1) Glyceraldehyde3-phosphate_c0[c1] + (1) Sedoheptulose7-phosphate_c0[c1] <-> (1) D-fructose-6-phosphate_c0[c1] + (1) D-Erythrose4-phosphate_c0[c1]	-0.0347288	No
rxn00777	Pentose phosphate pathway|Carbon fixation in photosynthetic organisms	D-ribose-5-phosphate aldose-ketose-isomerase	(1) ribose-5-phosphate_c0[c1] <-> (1) D-Ribulose5-phosphate_c0[c1]	0.0154338	No
rxn01200	Pentose phosphate pathway|Carbon fixation in photosynthetic organisms	Sedoheptulose-7-phosphate:D-glyceraldehyde-3-phosphate glycolaldehyde transferase	(1) Glyceraldehyde3-phosphate_c0[c1] + (1) Sedoheptulose7-phosphate_c0[c1] <-> (1) ribose-5-phosphate_c0[c1] + (1) D-Xylulose5-phosphate_c0[c1]	0.0321807	Yes
rxn01187	Pentose phosphate pathway|Methane metabolism|Carbon fixation in photosynthetic organisms	D-Xylulose 5-phosphate D-glyceraldehyde-3-phosphate-lyase (adding phosphate; acetyl-phosphate-forming)	(1) Phosphate_c0[c1] + (1) D-Xylulose5-phosphate_c0[c1] -> (1) H2O_c0[c1] + (1) Glyceraldehyde3-phosphate_c0[c1] + (1) Acetylphosphate_c0[c1]	0.0453091	No
rxn03884	Pentose phosphate pathway|Pentose and glucuronate interconversions	2-dehydro-3-deoxy-D-gluconate-6-phosphate D-glyceraldehyde-3-phosphate-lyase	(1) 2-Keto-3-deoxy-6-phosphogluconate_c0[c1] <-> (1) Pyruvate_c0[c1] + (1) Glyceraldehyde3-phosphate_c0[c1]	0.0457443	No
rxn01116	Pentose phosphate pathway|Pentose and glucuronate interconversions|Carbon fixation in photosynthetic organisms	D-Ribulose-5-phosphate 3-epimerase	(1) D-Ribulose5-phosphate_c0[c1] <-> (1) D-Xylulose5-phosphate_c0[c1]	0.0131284	No
rxn00770	Pentose phosphate pathway|Purine metabolism	ATP:D-ribose-5-phosphate diphosphotransferase	(1) ATP_c0[c1] + (1) ribose-5-phosphate_c0[c1] <-> (1) AMP_c0[c1] + (1) H+_c0[c1] + (1) PRPP_c0[c1]	0.0167469	No

rxn03408	Peptidoglycan biosynthesis	UDP-N-acetyl-D-glucosamine:undecaprenyl-diphospho-N-acetylmuramoyl-L-alanyl-gamma-D-glutamyl-meso-2,6-diaminopimeloyl-D-alanyl-D-alanine 4-beta-N-acetylglucosaminlytransferase	(1) UDP-N-acetylglucosamine_c0[c1] + (1) Undecaprenyl-diphospho-N-acetylmuramoyl-L-alanyl-D-glutamyl-meso-2-6-diaminopimeloyl-D-alanyl-D-alanine_c0[c1] <-> (1) UDP_c0[c1] + (1) Undecaprenyl-diphospho-N-acetylmuramoyl--N-acetylglucosamine-L-ala-D-glu-meso-2-6-diaminopimeloyl-D-ala-D-ala_c0[c1]	0.000637032	No
rxn03904	Peptidoglycan biosynthesis	UDP-N-acetylmuramoyl-L-alanyl-gamma-D-glutamyl-meso-2,6-diaminopimeloyl-D-alanyl-D-alanine:undecaprenyl-phosphate phospho-N-acetylmuramoyl-pentapeptide-transferase	(1) Undecaprenylphosphate_c0[c1] + (1) UDP-N-acetylmuramoyl-L-alanyl-D-glutamyl-6-carboxy-L-lysyl-D-alanyl- D-alanine_c0[c1] <-> (1) UMP_c0[c1] + (1) Undecaprenyl-diphospho-N-acetylmuramoyl-L-alanyl-D-glutamyl-meso-2-6-diaminopimeloyl-D-alanyl-D-alanine_c0[c1]	0.000637032	No
rxn03901	Peptidoglycan biosynthesis	undecaprenyl-diphosphate phosphohydrolase	(1) H2O_c0[c1] + (1) Bactoprenyl diphosphate_c0[c1] -> (1) Phosphate_c0[c1] + (2) H+_c0[c1] + (1) Undecaprenylphosphate_c0[c1]	0.000637032	No
rxn01739	Phenylalanine, tyrosine and tryptophan biosynthesis	ATP:shikimate 3-phosphotransferase	(1) ATP_c0[c1] + (1) Shikimate_c0[c1] <-> (1) ADP_c0[c1] + (1) H+_c0[c1] + (1) 3-phosphoshikimate_c0[c1]	0.0015966	No
rxn02212	Phenylalanine, tyrosine and tryptophan biosynthesis	2-Dehydro-3-deoxy-D-arabino-heptonate 7-phosphate phosphate-lyase (cyclyzing)	(1) DAHP_c0[c1] -> (1) Phosphate_c0[c1] + (1) 5-Dehydroquinate_c0[c1]	0.0015966	No
rxn00727	Phenylalanine, tyrosine and tryptophan biosynthesis	Chorismate pyruvate-lyase (amino-accepting)	(1) L-Glutamine_c0[c1] + (1) Chorismate_c0[c1] -> (1) Pyruvate_c0[c1] + (1) L-Glutamate_c0[c1] + (1) H+_c0[c1] + (1) Anthranilate_c0[c1]	0.00120225	No
rxn01740	Phenylalanine, tyrosine and tryptophan biosynthesis	Shikimate:NADP+ 3-oxidoreductase	(1) NADP_c0[c1] + (1) Shikimate_c0[c1] <-> (1) NADPH_c0[c1] + (1) H+_c0[c1] + (1) 3-Dehydroshikimate_c0[c1]	-0.0015966	No
rxn01255	Phenylalanine, tyrosine and tryptophan biosynthesis	5-O-(1-Carboxyvinyl)-3-phosphoshikimate phosphate-lyase (chorismate-forming)	(1) 5-O--1-Carboxyvinyl-3-phosphoshikimate_c0[c1] -> (1) Phosphate_c0[c1] + (1) Chorismate_c0[c1]	0.0015966	No
rxn02508	Phenylalanine, tyrosine and tryptophan biosynthesis	N-(5-Phospho-beta-D-ribosyl)anthranilate ketol-isomerase	(1) N-5-phosphoribosyl-anthranilate_c0[c1] <-> (1) 1-(2-carboxyphenylamino)-1-deoxyribulose 5-phosphate_c0[c1]	0.00120225	No
rxn00791	Phenylalanine, tyrosine and tryptophan biosynthesis	N-(5-Phospho-D-ribosyl)anthranilate:pyrophosphate phosphoribosyl-transferase	(1) PPi_c0[c1] + (1) H+_c0[c1] + (1) N-5-phosphoribosyl-anthranilate_c0[c1] <- (1) Anthranilate_c0[c1] + (1) PRPP_c0[c1]	-0.00120225	No
rxn02476	Phenylalanine, tyrosine and tryptophan biosynthesis	Phosphoenolpyruvate:3-phosphoshikimate 5-O-(1-carboxyvinyl)-transferase	(1) Phosphoenolpyruvate_c0[c1] + (1) 3-phosphoshikimate_c0[c1] <-> (1) Phosphate_c0[c1] + (1) 5-O--1-Carboxyvinyl-3-phosphoshikimate_c0[c1]	0.0015966	No
rxn02213	Phenylalanine, tyrosine and tryptophan biosynthesis	3-Dehydroquinate hydro-lyase	(1) 5-Dehydroquinate_c0[c1] -> (1) H2O_c0[c1] + (1) 3-Dehydroshikimate_c0[c1]	0.0015966	No
rxn01332	Phenylalanine, tyrosine and tryptophan biosynthesis	Phosphoenolpyruvate:D-erythrose-4-phosphate C-(1-carboxyvinyl)transferase (phosphate hydrolysing, 2-carboxy-2-oxoethyl-forming)	(1) H2O_c0[c1] + (1) Phosphoenolpyruvate_c0[c1] + (1) D-Erythrose4-phosphate_c0[c1] -> (1) Phosphate_c0[c1] + (1) DAHP_c0[c1]	0.0015966	No

rxn02507	Phenylalanine, tyrosine and tryptophan biosynthesis	1-(2-Carboxyphenylamino)-1-deoxy-D-ribulose-5-phosphate carboxy-lyase(cyclizing)	(1) H+_c0[c1] + (1) 1-(2-carboxyphenylamino)-1-deoxyribulose 5-phosphate_c0[c1] -> (1) H2O_c0[c1] + (1) CO2_c0[c1] + (1) Indoleglycerol phosphate_c0[c1]	0.00120225	No
rxn00060	Porphyrin and chlorophyll metabolism	porphobilinogen:(4-[2-carboxyethyl]-3-[carboxymethyl]pyrrol-2-yl)methyltransferase (hydrolysing)	(1) H2O_c0[c1] + (4) Porphobilinogen_c0[c1] -> (4) NH3_c0[c1] + (1) Hydroxymethylbilane_c0[c1]	0.000157737	No
rxn02264	Porphyrin and chlorophyll metabolism	Hydroxymethylbilane hydro-lyase(cyclizing)	(1) Hydroxymethylbilane_c0[c1] <-> (1) H2O_c0[c1] + (1) UroporphyrinogenIII_c0[c1]	0.000157737	No
rxn00029	Porphyrin and chlorophyll metabolism	5-aminolevulinate hydro-lyase (adding 5-aminolevulinate and cyclizing; porphobilinogen-forming)	(2) 5-Aminolevulinate_c0[c1] -> (2) H2O_c0[c1] + (1) H+_c0[c1] + (1) Porphobilinogen_c0[c1]	0.000630948	No
rxn01629	Porphyrin and chlorophyll metabolism	(S)-4-Amino-5-oxopentanoate 4,5-aminomutase	(1) 5-Aminolevulinate_c0[c1] <-> (1) L-Glutamate1-semialdehyde_c0[c1]	-0.0012619	No
rxn03537	Porphyrin and chlorophyll metabolism	R05222	(1) GTP_c0[c1] + (1) Adenosyl cobinamide phosphate_c0[c1] <-> (1) PPi_c0[c1] + (1) H+_c0[c1] + (1) Adenosylcobinamide-GDP_c0[c1]	7.88684E-05	No
rxn04413	Porphyrin and chlorophyll metabolism	R06558	(1) GTP_c0[c1] + (1) Adenosyl cobinamide_c0[c1] <-> (1) GDP_c0[c1] + (1) H+_c0[c1] + (1) Adenosyl cobinamide phosphate_c0[c1]	7.88684E-05	No
rxn03150	Porphyrin and chlorophyll metabolism	R04594	(1) H2O_c0[c1] + (1) alpha-Ribazole 5'-phosphate_c0[c1] -> (1) Phosphate_c0[c1] + (1) alpha-Ribazole_c0[c1]	7.88684E-05	No
rxn05029	Porphyrin and chlorophyll metabolism	ATP:cobinamide Cobeta-adenosyltransferase	(1) ATP_c0[c1] + (1) H+_c0[c1] + (1) Cobinamide_c0[c1] <-> (1) Triphosphate_c0[c1] + (1) Adenosyl cobinamide_c0[c1]	7.88684E-05	No
rxn02303	Porphyrin and chlorophyll metabolism	Coproporphyrinogen:oxyge n oxidoreductase(decarboxyla ting)	(1) O2_c0[c1] + (2) H+_c0[c1] + (1) CoproporphyrinogenIII_c0[c1] <-> (2) H2O_c0[c1] + (2) CO2_c0[c1] + (1) ProtoporphyrinogenIX_c0[c1]	7.88684E-05	No
rxn03538	Porphyrin and chlorophyll metabolism	R05223	(1) alpha-Ribazole_c0[c1] + (1) Adenosylcobinamide-GDP_c0[c1] <-> (1) H+_c0[c1] + (1) GMP_c0[c1] + (1) Calomide_c0[c1]	7.88684E-05	No
rxn00056	Porphyrin and chlorophyll metabolism	Fe(II):oxygen oxidoreductase	(1) O2_c0[c1] + (4) H+_c0[c1] + (4) Fe2+_c0[c1] <-> (2) H2O_c0[c1] + (4) fe3_c0[c1]	1.97171E-05	No
rxn02897	Porphyrin and chlorophyll metabolism	Nicotinate-nucleotide:dimethylbenzimi dazole phospho-D-ribosyltransferase	(1) Nicotinate ribonucleotide_c0[c1] + (1) Dimethylbenzimidazole_c0[c1] <-> (1) H+_c0[c1] + (1) Niacin_c0[c1] + (1) alpha-Ribazole 5'-phosphate_c0[c1]	7.88684E-05	No
rxn02288	Porphyrin and chlorophyll metabolism	Uroporphyrinogen-III carboxy-lyase	(4) H+_c0[c1] + (1) UroporphyrinogenIII_c0[c1] <-> (4) CO2_c0[c1] + (1) CoproporphyrinogenIII_c0[c1]	7.88684E-05	No
rxn06591	Porphyrin and chlorophyll metabolism	L-glutamate-semialdehyde: NADP+ oxidoreductase(L-glutamyl-tRNA(Glu)-forming)	(1) NADPH_c0[c1] + (1) H+_c0[c1] + (1) L-Glutamyl-tRNA-Glu_c0[c1] <-> (1) NADP_c0[c1] + (1) L-Glutamate1-semialdehyde_c0[c1] + (1) tRNA-Glu_c0[c1]	0.0012619	No

rxn00224	Porphyrin and chlorophyll metabolism	protoheme ferro-lyase (protoporphyrin-forming)	(1) Protoporphyrin_c0[c1] + (1) Fe2+_c0[c1] <-> (1) Heme_c0[c1] + (2) H+_c0[c1]	7.88684E-05	No
rxn06937	Porphyrin and chlorophyll metabolism|Aminoacyl-tRNA biosynthesis	L-glutamate:tRNA(Glu) ligase (AMP-forming)	(1) ATP_c0[c1] + (1) L-Glutamate_c0[c1] + (1) tRNA-Glu_c0[c1] -> (1) PPi_c0[c1] + (1) AMP_c0[c1] + (1) H+_c0[c1] + (1) L-Glutamyl-tRNA-Glu_c0[c1]	0.0012619	No
rxn00669	Propanoate metabolism	Propanoate:CoA ligase (ADP-forming)	(1) ATP_c0[c1] + (1) CoA_c0[c1] + (1) Propionate_c0[c1] <-> (1) ADP_c0[c1] + (1) Phosphate_c0[c1] + (1) Propionyl-CoA_c0[c1]	0.0788386	No
rxn00990	Propanoate metabolism	Acetoacetyl-CoA:acetate CoA-transferase	(1) Acetate_c0[c1] + (1) Acetoacetyl-CoA_c0[c1] <-> (1) Acetyl-CoA_c0[c1] + (1) Acetoacetate_c0[c1]	0.0884975	No
rxn00097	Purine metabolism	ATP:AMP phosphotransferase	(1) ATP_c0[c1] + (1) AMP_c0[c1] + (1) H+_c0[c1] <-> (2) ADP_c0[c1]	-0.0362657	No
rxn00131	Purine metabolism	AMP phosphoribohydrolase	(1) H2O_c0[c1] + (1) AMP_c0[c1] <-> (1) ribose-5-phosphate_c0[c1] + (1) Adenine_c0[c1]	-0.00047321	No
rxn01445	Purine metabolism	2'-Deoxyguanosine 5'-monophosphate phosphohydrolase	(1) H2O_c0[c1] + (1) dGMP_c0[c1] -> (1) Phosphate_c0[c1] + (1) Deoxyguanosine_c0[c1]	0.000545188	No
rxn00917	Purine metabolism	Xanthosine-5'-phosphate:L-glutamine amido-ligase (AMP-forming)	(1) H2O_c0[c1] + (1) ATP_c0[c1] + (1) L-Glutamine_c0[c1] + (1) XMP_c0[c1] -> (1) PPi_c0[c1] + (1) AMP_c0[c1] + (1) L-Glutamate_c0[c1] + (3) H+_c0[c1] + (1) GMP_c0[c1]	0.0043884	No
rxn00927	Purine metabolism	Adenosine ribohydrolase	(1) H2O_c0[c1] + (1) Adenosine_c0[c1] <-> (1) D-Ribose_c0[c1] + (1) Adenine_c0[c1]	0.000473211	No
rxn01352	Purine metabolism	dGTP triphosphohydrolase	(1) H2O_c0[c1] + (1) dGTP_c0[c1] <-> (1) Deoxyguanosine_c0[c1] + (1) Triphosphate_c0[c1]	-0.00054519	No
rxn01509	Purine metabolism	ATP:dGMP phosphotransferase	(1) ATP_c0[c1] + (1) H+_c0[c1] + (1) dGMP_c0[c1] <-> (1) ADP_c0[c1] + (1) dGDP_c0[c1]	-0.00054519	No
rxn03147	Purine metabolism	1-(5-Phosphoribosyl)-5-amino-4-carboxyimidazole:L-aspartate ligase (ADP-forming)	(1) ATP_c0[c1] + (1) L-Aspartate_c0[c1] + (1) 5'-Phosphoribosyl-4-carboxy-5-aminoimidazole_c0[c1] -> (1) ADP_c0[c1] + (1) Phosphate_c0[c1] + (1) H+_c0[c1] + (1) SAICAR_c0[c1]	0.00793862	No
rxn00832	Purine metabolism	IMP 1,2-hydrolase (decyclizing)	(1) H2O_c0[c1] + (1) IMP_c0[c1] <-> (1) FAICAR_c0[c1]	-0.0099575	No
rxn02895	Purine metabolism	5-Phospho-D-ribosylamine:glycine ligase (ADP-forming)	(1) ATP_c0[c1] + (1) Glycine_c0[c1] + (1) 5-Phosphoribosylamine_c0[c1] -> (1) ADP_c0[c1] + (1) Phosphate_c0[c1] + (1) H+_c0[c1] + (1) GAR_c0[c1]	0.00801749	No
rxn05231	Purine metabolism	2'-Deoxyadenosine 5'-diphosphate:oxidized-thioredoxin 2'-oxidoreductase	(1) ADP_c0[c1] + (1) trdrd_c0[c1] -> (1) H2O_c0[c1] + (1) dADP_c0[c1] + (1) trdox_c0[c1]	0.000270778	No
rxn03136	Purine metabolism	1-(5'-Phosphoribosyl)-5-amino-4-(N-succinocarboxamide)-imidazole AMP-lyase	(1) SAICAR_c0[c1] <-> (1) H+_c0[c1] + (1) Fumarate_c0[c1] + (1) AICAR_c0[c1]	0.00793862	No
rxn02937	Purine metabolism	2-(Formamido)-N1-(5-phosphoribosyl)acetamidine cyclo-ligase (ADP-forming)	(1) ATP_c0[c1] + (1) 5'-Phosphoribosylformylglycinamidine_c0[c1] <-> (1) ADP_c0[c1] + (1) Phosphate_c0[c1] + (1) H+_c0[c1] + (1) AIR_c0[c1]	0.00801749	No

rxn00834	Purine metabolism	IMP:NAD+ oxidoreductase	(1) H2O_c0[c1] + (1) NAD_c0[c1] + (1) IMP_c0[c1] <-> (1) NADH_c0[c1] + (1) H+_c0[c1] + (1) XMP_c0[c1]	0.0043884	No
rxn03084	Purine metabolism	5'-Phosphoribosylformylglycinamide:L-glutamine amido-ligase (ADP-forming)	(1) H2O_c0[c1] + (1) ATP_c0[c1] + (1) L-Glutamine_c0[c1] + (1) N-Formyl-GAR_c0[c1] -> (1) ADP_c0[c1] + (1) Phosphate_c0[c1] + (1) L-Glutamate_c0[c1] + (1) H+_c0[c1] + (1) 5'-Phosphoribosylformylglycinamidine_c0[c1]	0.00801749	No
rxn05233	Purine metabolism	2'-Deoxyguanosine 5'-diphosphate:oxidized-thioredoxin 2'-oxidoreductase	(1) GDP_c0[c1] + (1) trdrd_c0[c1] -> (1) H2O_c0[c1] + (1) dGDP_c0[c1] + (1) trdox_c0[c1]	0.000545188	No
rxn00239	Purine metabolism	ATP:GMP phosphotransferase	(1) ATP_c0[c1] + (1) H+_c0[c1] + (1) GMP_c0[c1] <-> (1) ADP_c0[c1] + (1) GDP_c0[c1]	0.00446727	No
rxn00839	Purine metabolism	ATP:dADP phosphotransferase	(1) ATP_c0[c1] + (1) dADP_c0[c1] <-> (1) ADP_c0[c1] + (1) dATP_c0[c1]	0.000270778	No
rxn00800	Purine metabolism|Alanine, aspartate and glutamate metabolism	N6-(1,2-dicarboxyethyl)AMP AMP-lyase (fumarate-forming)	(1) Adenylosuccinate_c0[c1] <-> (1) AMP_c0[c1] + (1) Fumarate_c0[c1]	0.0055691	No
rxn00790	Purine metabolism|Alanine, aspartate and glutamate metabolism	5-phosphoribosylamine:diphosphate phospho-alpha-D-ribosyltransferase (glutamate-amidating)	(1) PPi_c0[c1] + (1) L-Glutamate_c0[c1] + (1) H+_c0[c1] + (1) 5-Phosphoribosylamine_c0[c1] <- (1) H2O_c0[c1] + (1) L-Glutamine_c0[c1] + (1) PRPP_c0[c1]	-0.00801749	No
rxn00838	Purine metabolism|Alanine, aspartate and glutamate metabolism	IMP:L-aspartate ligase (GDP-forming)	(1) GTP_c0[c1] + (1) L-Aspartate_c0[c1] + (1) IMP_c0[c1] -> (1) Phosphate_c0[c1] + (1) GDP_c0[c1] + (2) H+_c0[c1] + (1) Adenylosuccinate_c0[c1]	0.0055691	No
rxn03137	Purine metabolism|One carbon pool by folate	10-Formyltetrahydrofolate:5'-phosphoribosyl-5-amino-4-imidazolecarboxamide formyltransferase	(1) 10-Formyltetrahydrofolate_c0[c1] + (1) AICAR_c0[c1] <-> (1) Tetrahydrofolate_c0[c1] + (1) FAICAR_c0[c1]	0.0099575	No
rxn03004	Purine metabolism|One carbon pool by folate	10-Formyltetrahydrofolate:5'-phosphoribosylglycinamide formyltransferase	(1) 10-Formyltetrahydrofolate_c0[c1] + (1) GAR_c0[c1] <-> (1) H+_c0[c1] + (1) Tetrahydrofolate_c0[c1] + (1) N-Formyl-GAR_c0[c1]	0.00730767	No
rxn00379	Purine metabolism|Sulfur metabolism	ATP:sulfate adenylyltransferase	(1) ATP_c0[c1] + (1) Sulfate_c0[c1] <-> (1) PPi_c0[c1] + (1) APS_c0[c1]	0.015159	No
rxn00364	Pyrimidine metabolism	ATP:CMP phosphotransferase	(1) ATP_c0[c1] + (1) CMP_c0[c1] + (1) H+_c0[c1] <-> (1) ADP_c0[c1] + (1) CDP_c0[c1]	0.0140658	No
rxn01673	Pyrimidine metabolism	ATP:dCDP phosphotransferase	(1) ATP_c0[c1] + (1) dCDP_c0[c1] <-> (1) ADP_c0[c1] + (1) dCTP_c0[c1]	0.000545188	No
rxn00710	Pyrimidine metabolism	orotidine-5'-phosphate carboxy-lyase (UMP-forming)	(1) H+_c0[c1] + (1) Orotidylic acid_c0[c1] -> (1) CO2_c0[c1] + (1) UMP_c0[c1]	0.00527165	No
rxn01362	Pyrimidine metabolism	Orotidine-5'-phosphate:diphosphate phospho-alpha-D-ribosyl-transferase	(1) PPi_c0[c1] + (1) H+_c0[c1] + (1) Orotidylic acid_c0[c1] <- (1) PRPP_c0[c1] + (1) Orotate_c0[c1]	-0.00527165	No
rxn01465	Pyrimidine metabolism	(S)-dihydroorotate amidohydrolase	(1) H2O_c0[c1] + (1) S-Dihydroorotate_c0[c1] <-> (1) H+_c0[c1] + (1) N-Carbamoyl-L-aspartate_c0[c1]	-0.00527165	No
rxn01678	Pyrimidine metabolism	ATP:dUDP phosphotransferase	(1) ATP_c0[c1] + (1) dUDP_c0[c1] <-> (1) ADP_c0[c1] + (1) dUTP_c0[c1]	0.000270778	No

rxn00412	Pyrimidine metabolism	UTP:L-glutamine amido-ligase (ADP-forming)	(1) H2O_c0[c1] + (1) ATP_c0[c1] + (1) L-Glutamine_c0[c1] + (1) UTP_c0[c1] -> (1) ADP_c0[c1] + (1) Phosphate_c0[c1] + (1) L-Glutamate_c0[c1] + (1) CTP_c0[c1] + (2) H+_c0[c1]	0.00268735	No
rxn01512	Pyrimidine metabolism	ATP:dTDP phosphotransferase	(1) ATP_c0[c1] + (1) dTDP_c0[c1] <-> (1) ADP_c0[c1] + (1) TTP_c0[c1]	0.00090781	No
rxn06076	Pyrimidine metabolism	2'-Deoxycytidine diphosphate:oxidized-thioredoxin 2'-oxidoreductase	(1) H2O_c0[c1] + (1) dCDP_c0[c1] + (1) trdox_c0[c1] <- (1) CDP_c0[c1] + (1) trdrd_c0[c1]	-0.00054519	No
rxn00409	Pyrimidine metabolism	ATP:CDP phosphotransferase	(1) ATP_c0[c1] + (1) CDP_c0[c1] <-> (1) ADP_c0[c1] + (1) CTP_c0[c1]	0.0135206	No
rxn01519	Pyrimidine metabolism	dUTP nucleotidohydrolase	(1) H2O_c0[c1] + (1) dUTP_c0[c1] -> (1) PPi_c0[c1] + (2) H+_c0[c1] + (1) dUMP_c0[c1]	0.000270778	No
rxn01513	Pyrimidine metabolism	ATP:dTMP phosphotransferase	(1) ATP_c0[c1] + (1) H+_c0[c1] + (1) dTMP_c0[c1] <-> (1) ADP_c0[c1] + (1) dTDP_c0[c1]	0.000270778	No
rxn06075	Pyrimidine metabolism	2'-Deoxyuridine 5'-diphosphate:oxidized-thioredoxin 2'-oxidoreductase	(1) H2O_c0[c1] + (1) dUDP_c0[c1] + (1) trdox_c0[c1] <- (1) UDP_c0[c1] + (1) trdrd_c0[c1]	-0.00027078	No
rxn05289	Pyrimidine metabolism	NADPH:oxidized-thioredoxin oxidoreductase	(1) NADPH_c0[c1] + (1) H+_c0[c1] + (1) trdox_c0[c1] <-> (1) NADP_c0[c1] + (1) trdrd_c0[c1]	0.00722092	No
rxn00117	Pyrimidine metabolism	ATP:UDP phosphotransferase	(1) ATP_c0[c1] + (1) UDP_c0[c1] <-> (1) ADP_c0[c1] + (1) UTP_c0[c1]	0.000366253	No
rxn01018	Pyrimidine metabolism|Alanine, aspartate and glutamate metabolism	carbamoyl-phosphate:L-aspartate carbamoyltransferase	(1) L-Aspartate_c0[c1] + (1) Carbamoylphosphate_c0[c1] -> (1) Phosphate_c0[c1] + (1) H+_c0[c1] + (1) N-Carbamoyl-L-aspartate_c0[c1]	0.00527165	No
rxn00414	Pyrimidine metabolism|Alanine, aspartate and glutamate metabolism	hydrogen-carbonate:L-glutamine amido-ligase (ADP-forming, carbamate-phosphorylating)	(1) H2O_c0[c1] + (2) ATP_c0[c1] + (1) L-Glutamine_c0[c1] + (1) H2CO3_c0[c1] -> (2) ADP_c0[c1] + (1) Phosphate_c0[c1] + (1) L-Glutamate_c0[c1] + (2) H+_c0[c1] + (1) Carbamoylphosphate_c0[c1]	0.0115551	No
rxn01520	Pyrimidine metabolism|One carbon pool by folate	5,10-Methylenetetrahydrofolate:dUMP C-methyltransferase	(1) 5-10-Methylenetetrahydrofolate_c0[c1] + (1) dUMP_c0[c1] -> (1) dTMP_c0[c1] + (1) Dihydrofolate_c0[c1]	0.000270778	No
rxn00145	Pyruvate metabolism	(S)-Lactate:ferricytochrome-c 2-oxidoreductase	(2) Cytochrome c3+_c0[c1] + (1) L-Lactate_c0[c1] <-> (1) Pyruvate_c0[c1] + (2) H+_c0[c1] + (2) Cytochrome c2+_c0[c1]	-0.18635	No
rxn00251	Pyruvate metabolism|Methane metabolism|Carbon fixation in photosynthetic organisms|Carbon fixation pathways in prokaryotes	phosphate:oxaloacetate carboxy-lyase (adding phosphate;phosphoenolpyruvate-forming)	(1) Phosphate_c0[c1] + (1) Oxaloacetate_c0[c1] + (1) H+_c0[c1] <-> (1) H2O_c0[c1] + (1) CO2_c0[c1] + (1) Phosphoenolpyruvate_c0[c1]	-0.0499572	No
rxn05040	Riboflavin metabolism	D-ribulose 5-phosphate formate-lyase (L-3,4-dihydroxybutan-2-one 4-phosphate-forming)	(1) D-Ribulose5-phosphate_c0[c1] -> (1) Formate_c0[c1] + (1) H+_c0[c1] + (1) 3-4-dihydroxy-2-butanone4-phosphate_c0[c1]	0.000315474	No
rxn00300	Riboflavin metabolism	GTP 7,8-8,9-dihydrolase (diphosphate-forming)	(3) H2O_c0[c1] + (1) GTP_c0[c1] -> (1) PPi_c0[c1] + (1) Formate_c0[c1] + (3) H+_c0[c1] + (1) 2,5-Diamino-6-(5'-phosphoribosylamino)-4-pyrimidineone_c0[c1]	0.000157737	No

rxn00392	Riboflavin metabolism	ATP:riboflavin 5'-phosphotransferase	(1) ATP_c0[c1] + (1) Riboflavin_c0[c1] <-> (1) ADP_c0[c1] + (1) FMN_c0[c1] + (1) H+_c0[c1]	7.88684E-05	No
rxn03080	Riboflavin metabolism	5-amino-6-(D-ribitylamino)uracil butanedionetransferase	(1) 4--1-D-Ribitylamino-5-aminouracil_c0[c1] + (1) 3-4-dihydroxy-2-butanone4-phosphate_c0[c1] <-> (2) H2O_c0[c1] + (1) Phosphate_c0[c1] + (1) H+_c0[c1] + (1) 6-7-Dimethyl-8--1-D-ribityllumazine_c0[c1]	0.000315474	No
rxn00048	Riboflavin metabolism	6,7-Dimethyl-8-(1-D-ribityl)lumazine:6,7-dimethyl-8-(1-D-ribityl)lumazine 2,3-butanediyltransferase	(1) H+_c0[c1] + (2) 6-7-Dimethyl-8--1-D-ribityllumazine_c0[c1] -> (1) Riboflavin_c0[c1] + (1) 4--1-D-Ribitylamino-5-aminouracil_c0[c1]	0.000157737	No
rxn02475	Riboflavin metabolism	2,5-Diamino-6-hydroxy-4-(5-phosphoribosylamino)-pyrimidine 2-aminohydrolase	(1) H2O_c0[c1] + (1) H+_c0[c1] + (1) 2,5-Diamino-6-(5'-phosphoribosylamino)-4-pyrimidineone_c0[c1] -> (1) NH3_c0[c1] + (1) 5-Amino-6--5-phosphoribosylaminouracil_c0[c1]	0.000157737	No
rxn00122	Riboflavin metabolism	ATP:FMN adenylyltransferase	(1) ATP_c0[c1] + (1) FMN_c0[c1] -> (1) PPi_c0[c1] + (1) FAD_c0[c1]	7.88684E-05	No
rxn02474	Riboflavin metabolism	5-amino-6-(5-phosphoribitylamino)uracil:NADP+ 1'-oxidoreductase	(1) NADP_c0[c1] + (1) 5-Amino-6--5-phosphoribitylaminouracil_c0[c1] <-> (1) NADPH_c0[c1] + (1) H+_c0[c1] + (1) 5-Amino-6--5-phosphoribosylaminouracil_c0[c1]	-0.00015774	No
rxn02000	Streptomycin biosynthesis|Polyketide sugar unit biosynthesis	dTDP-4-dehydro-6-deoxy-D-glucose 3,5-epimerase	(1) dTDP-4-oxo-6-deoxy-D-glucose_c0[c1] <-> (1) dTDP-4-oxo-L-rhamnose_c0[c1]	0.000637032	No
rxn01675	Streptomycin biosynthesis|Polyketide sugar unit biosynthesis	dTTP:alpha-D-glucose-1-phosphate thymidylyltransferase	(1) Glucose-1-phosphate_c0[c1] + (1) TTP_c0[c1] <-> (1) PPi_c0[c1] + (1) dTDPglucose_c0[c1]	0.000637032	No
rxn02003	Streptomycin biosynthesis|Polyketide sugar unit biosynthesis	dTDP-6-deoxy-L-mannose:NADP+ 4-oxidoreductase	(1) NADP_c0[c1] + (1) dTDP-rhamnose_c0[c1] <-> (1) NADPH_c0[c1] + (1) H+_c0[c1] + (1) dTDP-4-oxo-L-rhamnose_c0[c1]	-0.00063703	No
rxn01997	Streptomycin biosynthesis|Polyketide sugar unit biosynthesis|Biosynthesis of vancomycin group antibiotics	dTDPglucose 4,6-hydro-lyase	(1) dTDPglucose_c0[c1] -> (1) H2O_c0[c1] + (1) dTDP-4-oxo-6-deoxy-D-glucose_c0[c1]	0.000637032	No
rxn00623	Sulfur metabolism	hydrogen-sulfide:NADP+ oxidoreductase	(3) H2O_c0[c1] + (3) NADP_c0[c1] + (1) H2S_c0[c1] <-> (3) NADPH_c0[c1] + (3) H+_c0[c1] + (1) Sulfite_c0[c1]	-0.00558899	No
rxn00290	Synthesis and degradation of ketone bodies|Valine, leucine and isoleucine degradation|Butanoate metabolism	succinyl-CoA:acetoacetate CoA-transferase	(1) Succinyl-CoA_c0[c1] + (1) Acetoacetate_c0[c1] <-> (1) Succinate_c0[c1] + (1) Acetoacetyl-CoA_c0[c1]	0.0863278	No
rxn00991	Synthesis and degradation of ketone bodies|Valine, leucine and isoleucine degradation|Butanoate metabolism	(S)-3-hydroxy-3-methylglutaryl-CoA acetoacetate-lyase (acetyl-CoA-forming)	(1) HMG-CoA_c0[c1] <-> (1) Acetyl-CoA_c0[c1] + (1) Acetoacetate_c0[c1]	-0.00216968	No
rxn00225	Taurine and hypotaurine metabolism|Pyruvate metabolism|Methane metabolism|Carbon fixation pathways in prokaryotes	ATP:acetate phosphotransferase	(1) ATP_c0[c1] + (1) Acetate_c0[c1] <-> (1) ADP_c0[c1] + (1) Acetylphosphate_c0[c1]	-0.0816345	No

rxn01486	Terpenoid backbone biosynthesis	trans,trans-Farnesyl-diphosphate:isopentenyl-diphosphate farnesyltranstransferase	(1) Isopentenyldiphosphate_c0[c1] + (1) Farnesyldiphosphate_c0[c1] -> (1) PPi_c0[c1] + (1) H+_c0[c1] + (1) Geranylgeranyl diphosphate_c0[c1]	0.000236605	No
rxn03958	Terpenoid backbone biosynthesis	1-Deoxy-D-xylulose-5-phosphate isomeroreductase	(1) NADP_c0[c1] + (1) 2-C-methyl-D-erythritol4-phosphate_c0[c1] <-> (1) NADPH_c0[c1] + (1) H+_c0[c1] + (1) 1-deoxy-D-xylulose5-phosphate_c0[c1]	-0.00890019	No
rxn03910	Terpenoid backbone biosynthesis	2-Phospho-4-(cytidine 5'-diphospho)-2-C-methyl-D-erythritol CMP-lyase (cyclizing)	(1) 2-phospho-4--cytidine5-diphospho-2-C-methyl-D-erythritol_c0[c1] <-> (1) CMP_c0[c1] + (1) 2-C-methyl-D-erythritol2-4-cyclodiphosphate_c0[c1]	0.00890019	No
rxn03908	Terpenoid backbone biosynthesis	ATP:4-(Cytidine 5'-diphospho)-2-C-methyl-D-erythritol 2-phosphotransferase	(1) ATP_c0[c1] + (1) 4--cytidine5-diphospho-2-C-methyl-D-erythritol_c0[c1] <-> (1) ADP_c0[c1] + (1) H+_c0[c1] + (1) 2-phospho-4--cytidine5-diphospho-2-C-methyl-D-erythritol_c0[c1]	0.00890019	No
rxn08352	Terpenoid backbone biosynthesis	R08210	(1) NADH_c0[c1] + (1) H+_c0[c1] + (1) 1-Hydroxy-2-methyl-2-butenyl 4-diphosphate_c0[c1] -> (1) H2O_c0[c1] + (1) NAD_c0[c1] + (1) DMAPP_c0[c1]	0.000873637	No
rxn08756	Terpenoid backbone biosynthesis	isopentenyl-diphosphate:NAD+ oxidoreductase	(1) NADH_c0[c1] + (1) H+_c0[c1] + (1) 1-Hydroxy-2-methyl-2-butenyl 4-diphosphate_c0[c1] -> (1) H2O_c0[c1] + (1) NAD_c0[c1] + (1) Isopentenyldiphosphate_c0[c1]	0.00802655	No
rxn03907	Terpenoid backbone biosynthesis	CTP: 2-C-Methyl-D-erythritol 4-phosphate cytidylyltransferase	(1) CTP_c0[c1] + (1) 2-C-methyl-D-erythritol4-phosphate_c0[c1] <-> (1) PPi_c0[c1] + (1) 4--cytidine5-diphospho-2-C-methyl-D-erythritol_c0[c1]	0.00890019	No
rxn01213	Terpenoid backbone biosynthesis	GPPSYN-RXN	(1) Isopentenyldiphosphate_c0[c1] + (1) DMAPP_c0[c1] -> (1) PPi_c0[c1] + (1) H+_c0[c1] + (1) Geranyldiphosphate_c0[c1]	0.000873637	No
rxn03909	Terpenoid backbone biosynthesis	1-Deoxy-D-xylulose-5-phosphate pyruvate-lyase (carboxylating)	(1) Pyruvate_c0[c1] + (1) H+_c0[c1] + (1) Glyceraldehyde3-phosphate_c0[c1] -> (1) CO2_c0[c1] + (1) 1-deoxy-D-xylulose5-phosphate_c0[c1]	0.00897906	No
rxn01466	Terpenoid backbone biosynthesis	Geranyl-diphosphate:isopentenyl-diphosphate geranyltrans-transferase	(1) Isopentenyldiphosphate_c0[c1] + (1) Geranyldiphosphate_c0[c1] -> (1) PPi_c0[c1] + (1) H+_c0[c1] + (1) Farnesyldiphosphate_c0[c1]	0.000873637	No
rxn00533	Tetracycline biosynthesis|Pyruvate metabolism|Propanoate metabolism|Carbon fixation pathways in prokaryotes	Acetyl-CoA:carbon-dioxide ligase (ADP-forming)	(1) ATP_c0[c1] + (1) Acetyl-CoA_c0[c1] + (1) H2CO3_c0[c1] <-> (1) ADP_c0[c1] + (1) Phosphate_c0[c1] + (1) H+_c0[c1] + (1) Malonyl-CoA_c0[c1]	0.0427567	No
rxn02305	Thiamine metabolism	2-methyl-4-amino-5-hydroxymethylpyrimidine-diphosphate:4-methyl-5-(2-phosphoethyl)-thiazole 2-methyl-4-aminopyridine-5-methenyltransferase	(1) 4-Methyl-5--2-phosphoethyl-thiazole_c0[c1] + (1) 4-Amino-2-methyl-5-diphosphomethylpyrimidine_c0[c1] <-> (1) PPi_c0[c1] + (1) Thiamine phosphate_c0[c1]	7.88684E-05	No
rxn02484	Thiamine metabolism	ATP:4-amino-5-hydroxymethyl-2-methylpyrimidine 5-phosphotransferase	(1) ATP_c0[c1] + (1) Toxopyrimidine_c0[c1] <-> (1) ADP_c0[c1] + (1) H+_c0[c1] + (1) 4-Amino-5-phosphomethyl-2-methylpyrimidine_c0[c1]	7.88684E-05	No

rxn03108	Thiamine metabolism	ATP:4-amino-2-methyl-5-phosphomethylpyrimidine phosphotransferase	(1) ATP_c0[c1] + (1) H+_c0[c1] + (1) 4-Amino-5-phosphomethyl-2-methylpyrimidine_c0[c1] <-> (1) ADP_c0[c1] + (1) 4-Amino-2-methyl-5-diphosphomethylpyrimidine_c0[c1]	7.88684E-05	No
rxn00438	Thiamine metabolism	ATP:thiamin-phosphate phosphotransferase	(1) ATP_c0[c1] + (1) H+_c0[c1] + (1) Thiamine phosphate_c0[c1] <-> (1) ADP_c0[c1] + (1) TPP_c0[c1]	7.88684E-05	No
rxn11946	Ubiquinone and other terpenoid-quinone biosynthesis	R05614	(1) S-Adenosyl-L-methionine_c0[c1] + (1) 2-Octaprenyl-3-methyl-5-hydroxy-6-methoxy-1,4-benzoquinone_c0[c1] <-> (1) S-Adenosyl-homocysteine_c0[c1] + (1) H+_c0[c1] + (1) Ubiquinone-8_c0[c1]	7.88684E-05	No
rxn04139	Ubiquinone and other terpenoid-quinone biosynthesis	2-Octaprenyl-3-methyl-6-methoxy-1,4-benzoquinone ,NADPH2:oxygen oxidoreductase	(1) NADPH_c0[c1] + (1) O2_c0[c1] + (1) H+_c0[c1] + (1) 2-Octaprenyl-3-methyl-6-methoxy-1,4-benzoquinone_c0[c1] -> (1) H2O_c0[c1] + (1) NADP_c0[c1] + (1) 2-Octaprenyl-3-methyl-5-hydroxy-6-methoxy-1,4-benzoquinone_c0[c1]	7.88684E-05	No
rxn03893	Ubiquinone and other terpenoid-quinone biosynthesis	all-trans-octaprenyl-diphosphate:4-hydroxybenzoate 3-octaprenyltransferase	(1) 4-Hydroxybenzoate_c0[c1] + (1) Farnesylfarnesylgeraniol_c0[c1] -> (1) PPi_c0[c1] + (1) H+_c0[c1] + (1) 3-Octaprenyl-4-hydroxybenzoate_c0[c1]	7.88684E-05	No
rxn03394	Ubiquinone and other terpenoid-quinone biosynthesis	R04987	(1) NADPH_c0[c1] + (1) O2_c0[c1] + (1) H+_c0[c1] + (1) 2-Octaprenylphenol_c0[c1] -> (1) H2O_c0[c1] + (1) NADP_c0[c1] + (1) 2-Octaprenyl-6-hydroxyphenol_c0[c1]	7.88684E-05	No
rxn03395	Ubiquinone and other terpenoid-quinone biosynthesis	S-adenosyl-L-methionine:3-(all-trans-octaprenyl)benzene-1,2-diol 2-O-methyltransferase	(1) S-Adenosyl-L-methionine_c0[c1] + (1) 2-Octaprenyl-6-hydroxyphenol_c0[c1] <-> (1) S-Adenosyl-homocysteine_c0[c1] + (1) H+_c0[c1] + (1) 2-Octaprenyl-6-methoxyphenol_c0[c1]	7.88684E-05	No
rxn03436	Valine, leucine and isoleucine biosynthesis	(S)-2-Aceto-2-hydroxybutanoate:NADP+ oxidoreductase (isomerizing)	(1) 2-Aceto-2-hydroxybutanoate_c0[c1] <-> (1) (R)-3-Hydroxy-3-methyl-2-oxopentanoate_c0[c1]	0.00615872	No
rxn03435	Valine, leucine and isoleucine biosynthesis	(R)-2,3-Dihydroxy-3-methylpentanoate:NADP+ oxidoreductase (isomerizing)	(1) NADP_c0[c1] + (1) 2,3-Dihydroxy-3-methylvalerate_c0[c1] <-> (1) NADPH_c0[c1] + (1) H+_c0[c1] + (1) (R)-3-Hydroxy-3-methyl-2-oxopentanoate_c0[c1]	-0.00615872	No
rxn00904	Valine, leucine and isoleucine biosynthesis	L-Valine:pyruvate aminotransferase	(1) Pyruvate_c0[c1] + (1) L-Valine_c0[c1] <-> (1) L-Alanine_c0[c1] + (1) 3-Methyl-2-oxobutanoate_c0[c1]	0.0114579	No
rxn03437	Valine, leucine and isoleucine biosynthesis	(R)-2,3-Dihydroxy-3-methylpentanoate hydro-lyase	(1) 2,3-Dihydroxy-3-methylvalerate_c0[c1] -> (1) H2O_c0[c1] + (1) 3MOP_c0[c1]	0.00615872	No
rxn03194	Valine, leucine and isoleucine biosynthesis	(S)-2-Aceto-2-hydroxybutanoate pyruvate-lyase (carboxylating)	(1) 2-Oxobutyrate_c0[c1] + (1) 2-Hydroxyethyl-ThPP_c0[c1] <-> (1) TPP_c0[c1] + (1) 2-Aceto-2-hydroxybutanoate_c0[c1]	0.00615872	No
rxn02933	Valine, leucine and isoleucine degradation	(2S,3S)-3-hydroxy-2-methylbutanoyl-CoA:NAD+ oxidoreductase	(1) NAD_c0[c1] + (1) 2-methyl-3-hydroxy-butyryl-CoA_c0[c1] <-> (1) NADH_c0[c1] + (1) H+_c0[c1] + (1) 2-Methylacetoacetyl-CoA_c0[c1]	-0.00216968	No
rxn01504	Valine, leucine and isoleucine degradation	(S)-3-Hydroxy-3-methylglutaryl-CoA hydro-lyase (trans-3-	(1) HMG-CoA_c0[c1] <-> (1) H2O_c0[c1] + (1) 3-Methylglutaconyl-CoA_c0[c1]	0.00216968	No

		methylglutaconyl-CoA-forming)			
rxn02934	Valine, leucine and isoleucine degradation	(2S,3S)-3-Hydroxy-2-methylbutanoyl-CoA hydro-liase	(1) 2-methyl-3-hydroxy-butyryl-CoA_c0[c1] <-> (1) H2O_c0[c1] + (1) Tiglyl-CoA_c0[c1]	0.00216968	No
rxn02866	Valine, leucine and isoleucine degradation	3-methylbutanoyl-CoA:(acceptor) 2,3-oxidoreductase	(1) FAD_c0[c1] + (1) H+_c0[c1] + (1) Isovaleryl-CoA_c0[c1] <- (1) FADH2_c0[c1] + (1) Dimethylacryloyl-CoA_c0[c1]	-0.00216968	No
rxn02889	Valine, leucine and isoleucine degradation	3-Methylcrotonoyl-CoA:carbon-dioxide ligase (ADP-forming)	(1) ATP_c0[c1] + (1) H2CO3_c0[c1] + (1) Dimethylacryloyl-CoA_c0[c1] <-> (1) ADP_c0[c1] + (1) Phosphate_c0[c1] + (1) H+_c0[c1] + (1) 3-Methylglutaconyl-CoA_c0[c1]	-0.00216968	No
rxn00676	Valine, leucine and isoleucine degradation	acetyl-CoA:propanoyl-CoA 2-C-acetyltransferase	(1) Acetyl-CoA_c0[c1] + (1) Propionyl-CoA_c0[c1] <-> (1) CoA_c0[c1] + (1) 2-Methylacetoacetyl-CoA_c0[c1]	0.00216968	Yes
rxn01355	Valine, leucine and isoleucine degradation|Glyoxylate and dicarboxylate metabolism|Propanoate metabolism|Carbon fixation pathways in prokaryotes	Propanoyl-CoA:carbon-dioxide ligase (ADP-forming)	(1) ATP_c0[c1] + (1) Propionyl-CoA_c0[c1] + (1) H2CO3_c0[c1] <-> (1) ADP_c0[c1] + (1) Phosphate_c0[c1] + (1) H+_c0[c1] + (1) D-methylmalonyl-CoA_c0[c1]	-0.00216968	No
rxn01996	Valine, leucine and isoleucine degradation|Glyoxylate and dicarboxylate metabolism|Propanoate metabolism|Carbon fixation pathways in prokaryotes	Methylmalonyl-CoA epimerase	(1) L-methylmalonyl-CoA_c0[c1] <-> (1) D-methylmalonyl-CoA_c0[c1]	0.00216968	No
rxn00602	Valine, leucine and isoleucine degradation|Glyoxylate and dicarboxylate metabolism|Propanoate metabolism|Carbon fixation pathways in prokaryotes	(R)-Methylmalonyl-CoA CoA-carbonylmutase	(1) L-methylmalonyl-CoA_c0[c1] <-> (1) Succinyl-CoA_c0[c1]	-0.00216968	No
rxn01575	Valine, leucine and isoleucine degradation|Valine, leucine and isoleucine biosynthesis|Glucosinolate biosynthesis	L-Isoleucine:2-oxoglutarate aminotransferase	(1) 2-Oxoglutarate_c0[c1] + (1) L-Isoleucine_c0[c1] <-> (1) L-Glutamate_c0[c1] + (1) 3MOP_c0[c1]	-0.00615872	No
rxn00903	Valine, leucine and isoleucine degradation|Valine, leucine and isoleucine biosynthesis|Pantothenate and CoA biosynthesis|Glucosinolate biosynthesis	L-Valine:2-oxoglutarate aminotransferase	(1) 2-Oxoglutarate_c0[c1] + (1) L-Valine_c0[c1] <-> (1) L-Glutamate_c0[c1] + (1) 3-Methyl-2-oxobutanoate_c0[c1]	-0.0204294	No
***Rhizobium* sp. CF142, community in MOPS medium**					
rxn00555	Alanine, aspartate and glutamate metabolism|Amino sugar and nucleotide sugar metabolism	L-glutamine:D-fructose-6-phosphate isomerase (deaminating)	(1) L-Glutamine_c0[c3] + (1) D-fructose-6-phosphate_c0[c3] <-> (1) L-Glutamate_c0[c3] + (1) D-Glucosamine phosphate_c0[c3]	0.0033975	No
rxn00802	Alanine, aspartate and glutamate metabolism|Arginine and proline metabolism	2-(Nomega-L-arginino)succinate arginine-lyase (fumarate-forming)	(1) L-Argininosuccinate_c0[c3] <-> (1) L-Arginine_c0[c3] + (1) Fumarate_c0[c3]	0.00261074	No
rxn00503	Alanine, aspartate and glutamate metabolism|Arginine and proline metabolism	(S)-1-pyrroline-5-carboxylate:NAD+ oxidoreductase	(2) H2O_c0[c3] + (1) NAD_c0[c3] + (1) 1-Pyrroline-5-carboxylate_c0[c3] <-> (1) NADH_c0[c3] + (1) L-Glutamate_c0[c3] + (1) H+_c0[c3]	-0.0062608	No

rxn01434	Alanine, aspartate and glutamate metabolism|Arginine and proline metabolism	L-Citrulline:L-aspartate ligase (AMP-forming)	(1) ATP_c0[c3] + (1) L-Aspartate_c0[c3] + (1) Citrulline_c0[c3] <-> (1) PPi_c0[c3] + (1) AMP_c0[c3] + (2) H+_c0[c3] + (1) L-Argininosuccinate_c0[c3]	0.00261074	No
rxn00182	Alanine, aspartate and glutamate metabolism|Arginine and proline metabolism|Taurine and hypotaurine metabolism|D-Glutamine and D-glutamate metabolism|Nitrogen metabolism	L-glutamate:NAD+ oxidoreductase (deaminating)	(1) H2O_c0[c3] + (1) NAD_c0[c3] + (1) L-Glutamate_c0[c3] <-> (1) NADH_c0[c3] + (1) NH3_c0[c3] + (1) 2-Oxoglutarate_c0[c3] + (1) H+_c0[c3]	-0.148718	No
rxn00260	Alanine, aspartate and glutamate metabolism|Carbon fixation in photosynthetic organisms	L-Aspartate:2-oxoglutarate aminotransferase	(1) 2-Oxoglutarate_c0[c3] + (1) L-Aspartate_c0[c3] <-> (1) L-Glutamate_c0[c3] + (1) Oxaloacetate_c0[c3]	-0.0049907	Yes
rxn00085	Alanine, aspartate and glutamate metabolism|Nitrogen metabolism	L-Glutamate:NADP+ oxidoreductase (transaminating)	(1) NADP_c0[c3] + (2) L-Glutamate_c0[c3] <-> (1) NADPH_c0[c3] + (1) 2-Oxoglutarate_c0[c3] + (1) L-Glutamine_c0[c3] + (1) H+_c0[c3]	0.0408985	No
rxn00278	Alanine, aspartate and glutamate metabolism|Taurine and hypotaurine metabolism	L-Alanine:NAD+ oxidoreductase (deaminating)	(1) H2O_c0[c3] + (1) NAD_c0[c3] + (1) L-Alanine_c0[c3] <-> (1) NADH_c0[c3] + (1) NH3_c0[c3] + (1) Pyruvate_c0[c3] + (1) H+_c0[c3]	-0.0172914	No
rxn03638	Amino sugar and nucleotide sugar metabolism	Acetyl-CoA:D-glucosamine-1-phosphate N-acetyltransferase	(1) Acetyl-CoA_c0[c3] + (1) D-Glucosamine1-phosphate_c0[c3] -> (1) CoA_c0[c3] + (1) H+_c0[c3] + (1) N-Acetyl-D-glucosamine1-phosphate_c0[c3]	0.0033975	No
rxn01485	Amino sugar and nucleotide sugar metabolism	D-Glucosamine 1-phosphate 1,6-phosphomutase	(1) D-Glucosamine1-phosphate_c0[c3] <-> (1) D-Glucosamine phosphate_c0[c3]	-0.0033975	No
rxn00293	Amino sugar and nucleotide sugar metabolism	UTP:N-acetyl-alpha-D-glucosamine-1-phosphate uridylyltransferase	(1) UTP_c0[c3] + (1) N-Acetyl-D-glucosamine1-phosphate_c0[c3] <-> (1) PPi_c0[c3] + (1) UDP-N-acetylglucosamine_c0[c3]	0.0033975	No
rxn02285	Amino sugar and nucleotide sugar metabolism|Peptidoglycan biosynthesis	UDP-N-acetylmuramate:NADP+ oxidoreductase	(1) NADP_c0[c3] + (1) UDP-MurNAc_c0[c3] <-> (1) NADPH_c0[c3] + (1) H+_c0[c3] + (1) UDP-N-acetylglucosamine enolpyruvate_c0[c3]	-0.000849375	No
rxn00461	Amino sugar and nucleotide sugar metabolism|Peptidoglycan biosynthesis	Phosphoenolpyruvate:UDP-N-acetyl-D-glucosamine 1-carboxyvinyl-transferase	(1) UDP-N-acetylglucosamine_c0[c3] + (1) Phosphoenolpyruvate_c0[c3] <-> (1) Phosphate_c0[c3] + (1) UDP-N-acetylglucosamine enolpyruvate_c0[c3]	0.000849375	No
rxn00179	Arginine and proline metabolism	ATP:L-glutamate 5-phosphotransferase	(1) ATP_c0[c3] + (1) L-Glutamate_c0[c3] <-> (1) ADP_c0[c3] + (1) L-Glutamyl 5-phosphate_c0[c3]	0.00848313	No
rxn02373	Arginine and proline metabolism	L-glutamate-5-semialdehyde:NADP+ 5-oxidoreductase (phosphorylating)	(1) NADP_c0[c3] + (1) Phosphate_c0[c3] + (1) L-Glutamate5-semialdehyde_c0[c3] <- (1) NADPH_c0[c3] + (1) H+_c0[c3] + (1) L-Glutamyl 5-phosphate_c0[c3]	-0.00848313	No
rxn00929	Arginine and proline metabolism	L-Proline:NAD+ 5-oxidoreductase	(1) NAD_c0[c3] + (1) L-Proline_c0[c3] <-> (1) NADH_c0[c3] + (2) H+_c0[c3] + (1) 1-Pyrroline-5-carboxylate_c0[c3]	-0.0062608	No
rxn00467	Arginine and proline metabolism	L-Ornithine:2-oxo-acid aminotransferase	(1) 2-Oxoglutarate_c0[c3] + (1) Ornithine_c0[c3] <-> (1) L-	-0.00848313	No

			Glutamate_c0[c3] + (1) L-Glutamate5-semialdehyde_c0[c3]		
rxn01019	Arginine and proline metabolism	Carbamoyl-phosphate:L-ornithine carbamoyltransferase	(1) Ornithine_c0[c3] + (1) Carbamoylphosphate_c0[c3] -> (1) Phosphate_c0[c3] + (1) H+_c0[c3] + (1) Citrulline_c0[c3]	0.00261074	No
rxn00470	Arginine and proline metabolism|Glutathione metabolism	L-ornithine carboxy-lyase (putrescine-forming)	(1) Ornithine_c0[c3] + (1) H+_c0[c3] -> (1) CO2_c0[c3] + (1) Putrescine_c0[c3]	0.000105158	No
rxn01634	Ascorbate and aldarate metabolism	5-Dehydro-4-deoxy-D-glucarate hydro-lyase (decarboxylating)	(1) H+_c0[c3] + (1) 5-Dehydro-4-deoxy-D-glucarate_c0[c3] -> (1) H2O_c0[c3] + (1) CO2_c0[c3] + (1) 2,5-Dioxopentanoate_c0[c3]	0.0120935	No
rxn01990	Ascorbate and aldarate metabolism	5-Dehydro-4-deoxy-D-glucarate tartronate-semialdehyde-lyase	(1) 5-Dehydro-4-deoxy-D-glucarate_c0[c3] <-> (1) Pyruvate_c0[c3] + (1) Tartronate semialdehyde_c0[c3]	-0.0120935	No
rxn00501	beta-Alanine metabolism|Inositol phosphate metabolism|Propanoate metabolism	3-Oxopropanoate:NAD+ oxidoreductase (decarboxylating, CoA-acetylating)	(1) NAD_c0[c3] + (1) CoA_c0[c3] + (1) 3-Oxopropanoate_c0[c3] <-> (1) NADH_c0[c3] + (1) CO2_c0[c3] + (1) Acetyl-CoA_c0[c3]	-0.000210316	No
rxn01791	beta-Alanine metabolism|Pantothenate and CoA biosynthesis	(R)-Pantoate:beta-alanine ligase (AMP-forming)	(1) ATP_c0[c3] + (1) beta-Alanine_c0[c3] + (1) Pantoate_c0[c3] -> (1) PPi_c0[c3] + (1) AMP_c0[c3] + (2) H+_c0[c3] + (1) PAN_c0[c3]	0.000210316	No
rxn00656	beta-Alanine metabolism|Propanoate metabolism	L-Alanine:3-oxopropanoate aminotransferase	(1) L-Alanine_c0[c3] + (1) 3-Oxopropanoate_c0[c3] <-> (1) Pyruvate_c0[c3] + (1) beta-Alanine_c0[c3]	0.000210316	Yes
rxn02185	Butanoate metabolism	2-Acetolactate pyruvate-lyase (carboxylating)	(1) TPP_c0[c3] + (1) ALCTT_c0[c3] <-> (1) Pyruvate_c0[c3] + (1) 2-Hydroxyethyl-ThPP_c0[c3]	-0.0121724	No
rxn00548	Carbon fixation in photosynthetic organisms	D-fructose-6-phosphate D-erythrose-4-phosphate-lyase (adding phosphate; acetyl-phosphate-forming)	(1) Phosphate_c0[c3] + (1) D-fructose-6-phosphate_c0[c3] -> (1) H2O_c0[c3] + (1) Acetylphosphate_c0[c3] + (1) D-Erythrose4-phosphate_c0[c3]	0.0605367	No
rxn00285	Citrate cycle (TCA cycle)|Propanoate metabolism|Carbon fixation pathways in prokaryotes	Succinate:CoA ligase (ADP-forming)	(1) ATP_c0[c3] + (1) CoA_c0[c3] + (1) Succinate_c0[c3] <-> (1) ADP_c0[c3] + (1) Phosphate_c0[c3] + (1) Succinyl-CoA_c0[c3]	0.0251394	Yes
rxn00248	Citrate cycle (TCA cycle)|Pyruvate metabolism|Glyoxylate and dicarboxylate metabolism|Methane metabolism|Carbon fixation in photosynthetic organisms|Carbon fixation pathways in prokaryotes	(S)-malate:NAD+ oxidoreductase	(1) NAD_c0[c3] + (1) L-Malate_c0[c3] <-> (1) NADH_c0[c3] + (1) Oxaloacetate_c0[c3] + (1) H+_c0[c3]	0.0049907	Yes
rxn05957	Cysteine and methionine metabolism	O4-succinyl-L-homoserine:hydrogen sulfide S-(3-amino-3-carboxypropyl)transferase	(1) H2S_c0[c3] + (1) O-Succinyl-L-homoserine_c0[c3] -> (1) Succinate_c0[c3] + (1) Homocysteine_c0[c3]	0.00444537	No
rxn00126	Cysteine and methionine metabolism	ATP:L-methionine S-adenosyltransferase	(1) H2O_c0[c3] + (1) ATP_c0[c3] + (1) L-Methionine_c0[c3] -> (1) Phosphate_c0[c3] + (1) PPi_c0[c3] + (1) S-Adenosyl-L-methionine_c0[c3] + (1) H+_c0[c3]	0.000736105	No
rxn00141	Cysteine and methionine metabolism	S-Adenosyl-L-homocysteine hydrolase	(1) H2O_c0[c3] + (1) S-Adenosyl-homocysteine_c0[c3] <-> (1) Homocysteine_c0[c3] + (1) Adenosine_c0[c3]	0.000630948	No

rxn00693	Cysteine and methionine metabolism|One carbon pool by folate	5-Methyltetrahydrofolate:L-homocysteine S-methyltransferase	(1) Homocysteine_c0[c3] + (1) 5-Methyltetrahydrofolate_c0[c3] <-> (1) L-Methionine_c0[c3] + (1) Tetrahydrofolate_c0[c3]	0.00507632	No
rxn00423	Cysteine and methionine metabolism|Sulfur metabolism	acetyl-CoA:L-serine O-acetyltransferase	(1) Acetyl-CoA_c0[c3] + (1) L-Serine_c0[c3] -> (1) CoA_c0[c3] + (1) O-Acetyl-L-serine_c0[c3]	0.00290146	No
rxn00649	Cysteine and methionine metabolism|Sulfur metabolism	O3-acetyl-L-serine:hydrogen-sulfide 2-amino-2-carboxyethyltransferase	(1) H2S_c0[c3] + (1) O-Acetyl-L-serine_c0[c3] -> (1) Acetate_c0[c3] + (1) L-Cysteine_c0[c3]	0.00290146	No
rxn01304	Cysteine and methionine metabolism|Sulfur metabolism	Succinyl-CoA:L-homoserine O-succinyltransferase	(1) Succinyl-CoA_c0[c3] + (1) L-Homoserine_c0[c3] -> (1) CoA_c0[c3] + (1) O-Succinyl-L-homoserine_c0[c3]	0.00444537	No
rxn00283	D-Alanine metabolism	alanine racemase	(1) L-Alanine_c0[c3] <-> (1) D-Alanine_c0[c3]	0.00169875	No
rxn00193	D-Glutamine and D-glutamate metabolism	glutamate racemase	(1) L-Glutamate_c0[c3] <-> (1) D-Glutamate_c0[c3]	0.000849375	No
rxn02286	D-Glutamine and D-glutamate metabolism|Peptidoglycan biosynthesis	UDP-N-acetylmuramate:L-alanine ligase (ADP-forming)	(1) ATP_c0[c3] + (1) L-Alanine_c0[c3] + (1) UDP-MurNAc_c0[c3] -> (1) ADP_c0[c3] + (1) Phosphate_c0[c3] + (1) H+_c0[c3] + (1) UDP-N-acetylmuramoyl-L-alanine_c0[c3]	0.000849375	No
rxn02008	D-Glutamine and D-glutamate metabolism|Peptidoglycan biosynthesis	UDP-N-acetylmuramoyl-L-alanine:D-glutamate ligase(ADP-forming)	(1) ATP_c0[c3] + (1) D-Glutamate_c0[c3] + (1) UDP-N-acetylmuramoyl-L-alanine_c0[c3] -> (1) ADP_c0[c3] + (1) Phosphate_c0[c3] + (1) H+_c0[c3] + (1) UDP-N-acetylmuramoyl-L-alanyl-D-glutamate_c0[c3]	0.000849375	No
rxn05345	Fatty acid biosynthesis	dodecanoyl-[acyl-carrier-protein]:malonyl-[acyl-carrier-protein] C-acyltransferase (decarboxylating)	(1) Dodecanoyl-ACP_c0[c3] + (1) Malonyl-acyl-carrierprotein-_c0[c3] -> (1) CO2_c0[c3] + (1) 3-oxotetradecanoyl-acp_c0[c3] + (1) ACP_c0[c3]	0.0033975	Yes
rxn05465	Fatty acid biosynthesis	Malonyl-CoA:[acyl-carrier-protein] S-malonyltransferase	(1) H+_c0[c3] + (1) Malonyl-CoA_c0[c3] + (1) ACP_c0[c3] <-> (1) CoA_c0[c3] + (1) Malonyl-acyl-carrierprotein-_c0[c3]	0.0338656	No
rxn05342	Fatty acid biosynthesis	(3R)-3-Hydroxytetradecanoyl-[acyl-carrier-protein]:NADP+ oxidoreductase	(1) NADP_c0[c3] + (1) HMA_c0[c3] <-> (1) NADPH_c0[c3] + (1) 3-oxotetradecanoyl-acp_c0[c3]	-0.0033975	Yes
rxn02504	Folate biosynthesis	2-amino-4-hydroxy-6-(D-erythro-1,2,3-trihydroxypropyl)-7,8-dihydropteridine glycolaldehyde-lyase	(1) Dihydroneopterin_c0[c3] <-> (1) Glycolaldehyde_c0[c3] + (1) 6-hydroxymethyl dihydropterin_c0[c3]	0.000315474	No
rxn03167	Folate biosynthesis	2-Amino-4-hydroxy-6-(erythro-1,2,3-trihydroxypropyl) dihydropteridine triphosphate phosphohydrolase (alkaline optimum)	(3) H2O_c0[c3] + (1) 7,8-Dihydroneopterin 3'-triphosphate_c0[c3] -> (3) Phosphate_c0[c3] + (3) H+_c0[c3] + (1) Dihydroneopterin_c0[c3]	0.000315474	No
rxn02200	Folate biosynthesis	2-amino-4-hydroxy-6-hydroxymethyl-7,8-dihydropteridine:4-aminobenzoate 2-amino-4-hydroxydihydropteridine-6-methenyltransferase	(1) ABEE_c0[c3] + (1) 6-hydroxymethyl dihydropterin_c0[c3] <-> (1) H2O_c0[c3] + (1) Dihydropteroate_c0[c3]	0.000315474	No

rxn03841	Folate biosynthesis	4-amino-4-deoxychorismate pyruvate-lyase	(1) ADC_c0[c3] -> (1) Pyruvate_c0[c3] + (1) H+_c0[c3] + (1) ABEE_c0[c3]	0.000315474	No
rxn01257	Folate biosynthesis	chorismate:L-glutamine aminotransferase	(1) L-Glutamine_c0[c3] + (1) Chorismate_c0[c3] <-> (1) L-Glutamate_c0[c3] + (1) ADC_c0[c3]	0.000315474	No
rxn01603	Folate biosynthesis	7,8-dihydropteroate:L-glutamate ligase (ADP-forming)	(1) ATP_c0[c3] + (1) L-Glutamate_c0[c3] + (1) Dihydropteroate_c0[c3] -> (1) ADP_c0[c3] + (1) Phosphate_c0[c3] + (1) H+_c0[c3] + (1) Dihydrofolate_c0[c3]	0.000315474	No
rxn00650	Glutathione metabolism	L-cysteinylglycine dipeptidase	(1) H2O_c0[c3] + (1) Cys-Gly_c0[c3] <-> (1) Glycine_c0[c3] + (1) L-Cysteine_c0[c3]	-0.000105158	No
rxn00350	Glutathione metabolism	glutathione gamma-glutamylaminopeptidase	(1) H2O_c0[c3] + (1) GSH_c0[c3] <-> (1) L-Glutamate_c0[c3] + (1) Cys-Gly_c0[c3]	-0.000105158	No
rxn00615	Glycerolipid metabolism	ATP:glycerol 3-phosphotransferase	(1) ATP_c0[c3] + (1) Glycerol_c0[c3] <-> (1) ADP_c0[c3] + (1) H+_c0[c3] + (1) Glycerol-3-phosphate_c0[c3]	0.00108484	No
rxn00539	Glycerophospholipid metabolism	ethanolamine ammonia-lyase (acetaldehyde-forming)	(1) Aminoethanol_c0[c3] -> (1) NH3_c0[c3] + (1) Acetaldehyde_c0[c3]	0.000315474	No
rxn00611	Glycerophospholipid metabolism	sn-Glycerol-3-phosphate:NAD+ 2-oxidoreductase	(1) NAD_c0[c3] + (1) Glycerol-3-phosphate_c0[c3] <-> (1) NADH_c0[c3] + (1) H+_c0[c3] + (1) Glycerone-phosphate_c0[c3]	-0.00650905	No
rxn01068	Glycine, serine and threonine metabolism	L-threonine:NAD+ oxidoreductase	(1) NAD_c0[c3] + (1) L-Threonine_c0[c3] <-> (1) NADH_c0[c3] + (1) H+_c0[c3] + (1) L-2-Amino-acetoacetate_c0[c3]	-0.0179572	No
rxn00541	Glycine, serine and threonine metabolism	L-threonine acetaldehyde-lyase (glycine-forming)	(1) L-Threonine_c0[c3] <-> (1) Glycine_c0[c3] + (1) Acetaldehyde_c0[c3]	-0.000315474	No
rxn00274	Glycine, serine and threonine metabolism	Acetyl-CoA:glycine C-acetyltransferase	(1) Acetyl-CoA_c0[c3] + (1) Glycine_c0[c3] <-> (1) CoA_c0[c3] + (1) L-2-Amino-acetoacetate_c0[c3]	0.0179572	No
rxn00692	Glycine, serine and threonine metabolism|Cyanoamino acid metabolism|Glyoxylate and dicarboxylate metabolism|One carbon pool by folate|Methane metabolism	5,10-Methylenetetrahydrofolate:glycine hydroxymethyltransferase	(1) H2O_c0[c3] + (1) Glycine_c0[c3] + (1) 5-10-Methylenetetrahydrofolate_c0[c3] <-> (1) L-Serine_c0[c3] + (1) Tetrahydrofolate_c0[c3]	-0.0317946	No
rxn00337	Glycine, serine and threonine metabolism|Cysteine and methionine metabolism|Lysine biosynthesis	ATP:L-aspartate 4-phosphotransferase	(1) ATP_c0[c3] + (1) L-Aspartate_c0[c3] <-> (1) ADP_c0[c3] + (1) 4-Phospho-L-aspartate_c0[c3]	0.00529475	No
rxn01301	Glycine, serine and threonine metabolism|Cysteine and methionine metabolism|Lysine biosynthesis	L-Homoserine:NAD+ oxidoreductase	(1) NAD_c0[c3] + (1) L-Homoserine_c0[c3] <-> (1) NADH_c0[c3] + (1) H+_c0[c3] + (1) L-Aspartate4-semialdehyde_c0[c3]	-0.00444537	No
rxn01643	Glycine, serine and threonine metabolism|Cysteine and methionine metabolism|Lysine biosynthesis	L-Aspartate-4-semialdehyde:NADP+ oxidoreductase (phosphorylating)	(1) NADP_c0[c3] + (1) Phosphate_c0[c3] + (1) L-Aspartate4-semialdehyde_c0[c3] <- (1) NADPH_c0[c3] + (1) H+_c0[c3] + (1) 4-Phospho-L-aspartate_c0[c3]	-0.00529475	No
rxn01102	Glycine, serine and threonine metabolism|Glycerolipid	ATP:(R)-glycerate 3-phosphotransferase	(1) ATP_c0[c3] + (1) Glycerate_c0[c3] <-> (1) ADP_c0[c3] + (1) H+_c0[c3] + (1) 3-Phosphoglycerate_c0[c3]	-0.0120935	No

	metabolism|Glyoxylate and dicarboxylate metabolism				
rxn00420	Glycine, serine and threonine metabolism|Methane metabolism	O-phospho-L-serine phosphohydrolase	(1) H2O_c0[c3] + (1) phosphoserine_c0[c3] -> (1) Phosphate_c0[c3] + (1) L-Serine_c0[c3]	0.0434784	No
rxn01101	Glycine, serine and threonine metabolism|Methane metabolism	3-Phospho-D-glycerate:NAD+ 2-oxidoreductase	(1) NAD_c0[c3] + (1) 3-Phosphoglycerate_c0[c3] <-> (1) NADH_c0[c3] + (1) H+_c0[c3] + (1) 3-Phosphonooxypyruvate_c0[c3]	0.0434784	No
rxn02914	Glycine, serine and threonine metabolism|Methane metabolism	3-Phosphoserine:2-oxoglutarate aminotransferase	(1) 2-Oxoglutarate_c0[c3] + (1) phosphoserine_c0[c3] <-> (1) L-Glutamate_c0[c3] + (1) 3-Phosphonooxypyruvate_c0[c3]	-0.0434784	No
rxn01964	Glycine, serine and threonine metabolism|Phenylalanine, tyrosine and tryptophan biosynthesis	L-serine hydro-lyase [adding 1-C-(indol-3-yl)glycerol 3-phosphate; L-tryptophan and glyceraldehyde-3-phosphate-forming]	(1) L-Serine_c0[c3] + (1) Indoleglycerol phosphate_c0[c3] -> (1) H2O_c0[c3] + (1) L-Tryptophan_c0[c3] + (1) Glyceraldehyde3-phosphate_c0[c3]	0.00160301	No
rxn00599	Glycine, serine and threonine metabolism|Porphyrin and chlorophyll metabolism	succinyl-CoA:glycine C-succinyltransferase (decarboxylating)	(1) Glycine_c0[c3] + (1) H+_c0[c3] + (1) Succinyl-CoA_c0[c3] -> (1) CoA_c0[c3] + (1) CO2_c0[c3] + (1) 5-Aminolevulinate_c0[c3]	0.00168253	No
rxn00737	Glycine, serine and threonine metabolism|Valine, leucine and isoleucine biosynthesis	L-threonine ammonia-lyase (2-oxobutanoate-forming)	(1) L-Threonine_c0[c3] -> (1) NH3_c0[c3] + (1) 2-Oxobutyrate_c0[c3]	0.0111045	No
rxn00781	Glycolysis / Gluconeogenesis|Carbon fixation in photosynthetic organisms	D-glyceraldehyde-3-phosphate:NAD+ oxidoreductase (phosphorylating)	(1) NAD_c0[c3] + (1) Phosphate_c0[c3] + (1) Glyceraldehyde3-phosphate_c0[c3] <-> (1) NADH_c0[c3] + (1) H+_c0[c3] + (1) 1,3-Bisphospho-D-glycerate_c0[c3]	0.0898789	Yes
rxn01100	Glycolysis / Gluconeogenesis|Carbon fixation in photosynthetic organisms	ATP:3-phospho-D-glycerate 1-phosphotransferase	(1) ATP_c0[c3] + (1) 3-Phosphoglycerate_c0[c3] <-> (1) ADP_c0[c3] + (1) 1,3-Bisphospho-D-glycerate_c0[c3]	-0.0898789	No
rxn00011	Glycolysis / Gluconeogenesis|Citrate cycle (TCA cycle)|Valine, leucine and isoleucine biosynthesis|Pyruvate metabolism|Butanoate metabolism	pyruvate:thiamin diphosphate acetaldehydetransferase (decarboxylating)	(1) CO2_c0[c3] + (1) 2-Hydroxyethyl-ThPP_c0[c3] <- (1) Pyruvate_c0[c3] + (1) TPP_c0[c3] + (1) H+_c0[c3]	-0.0232769	Yes
rxn00747	Glycolysis / Gluconeogenesis|Fructose and mannose metabolism|Inositol phosphate metabolism|Carbon fixation in photosynthetic organisms	D-glyceraldehyde-3-phosphate aldose-ketose-isomerase	(1) Glyceraldehyde3-phosphate_c0[c3] <-> (1) Glycerone-phosphate_c0[c3]	0.00671937	No
rxn01106	Glycolysis / Gluconeogenesis|Glycine, serine and threonine metabolism|Methane metabolism	2-Phospho-D-glycerate 2,3-phosphomutase	(1) 2-Phospho-D-glycerate_c0[c3] <-> (1) 3-Phosphoglycerate_c0[c3]	-0.034307	Yes
rxn00459	Glycolysis / Gluconeogenesis|Methane metabolism	2-phospho-D-glycerate hydro-lyase (phosphoenolpyruvate-forming)	(1) 2-Phospho-D-glycerate_c0[c3] <-> (1) H2O_c0[c3] + (1) Phosphoenolpyruvate_c0[c3]	0.034307	Yes
rxn00175	Glycolysis / Gluconeogenesis|Methane metabolism|Carbon fixation pathways in prokaryotes	Acetate:CoA ligase (AMP-forming)	(1) ATP_c0[c3] + (1) CoA_c0[c3] + (1) Acetate_c0[c3] <-> (1) PPi_c0[c3] + (1) AMP_c0[c3] + (1) Acetyl-CoA_c0[c3] + (1) H+_c0[c3]	0.120042	No

rxn00148	Glycolysis / Gluconeogenesis|Purine metabolism|Pyruvate metabolism|Carbon fixation in photosynthetic organisms	ATP:pyruvate 2-O-phosphotransferase	(1) ATP_c0[c3] + (1) Pyruvate_c0[c3] <-> (1) ADP_c0[c3] + (1) Phosphoenolpyruvate_c0[c3] + (1) H+_c0[c3]	0.337417	No
rxn00499	Glycolysis / Gluconeogenesis|Pyruvate metabolism	(S)-Lactate:NAD+ oxidoreductase	(1) NAD_c0[c3] + (1) L-Lactate_c0[c3] <-> (1) NADH_c0[c3] + (1) Pyruvate_c0[c3] + (1) H+_c0[c3]	0.286665	No
rxn01281	Glyoxylate and dicarboxylate metabolism	(R)-Glycerate:NADP+ oxidoreductase	(1) NADP_c0[c3] + (1) Glycerate_c0[c3] <-> (1) NADPH_c0[c3] + (1) H+_c0[c3] + (1) Tartronate semialdehyde_c0[c3]	0.0120935	No
rxn00333	Glyoxylate and dicarboxylate metabolism	Glycolate:oxygen 2-oxidoreductase	(1) O2_c0[c3] + (1) Glycolate_c0[c3] -> (1) H2O2_c0[c3] + (1) Glyoxalate_c0[c3]	0.000105158	No
rxn00371	Glyoxylate and dicarboxylate metabolism|Methane metabolism	Formate:NAD+ oxidoreductase	(1) NAD_c0[c3] + (1) Formate_c0[c3] -> (1) NADH_c0[c3] + (1) CO2_c0[c3]	0.00294849	No
rxn02320	Histidine metabolism	5-Amino-2-oxopentanoate:2-oxoglutarate aminotransferase	(1) 2-Oxoglutarate_c0[c3] + (1) L-histidinol-phosphate_c0[c3] <-> (1) L-Glutamate_c0[c3] + (1) imidazole acetol-phosphate_c0[c3]	-0.00269184	No
rxn00789	Histidine metabolism	1-(5-phospho-D-ribosyl)-ATP:diphosphate phospho-alpha-D-ribosyl-transferase	(1) PPi_c0[c3] + (1) H+_c0[c3] + (1) Phosphoribosyl-ATP_c0[c3] <- (1) ATP_c0[c3] + (1) PRPP_c0[c3]	-0.00269184	No
rxn02160	Histidine metabolism	L-Histidinol-phosphate phosphohydrolase	(1) H2O_c0[c3] + (1) L-histidinol-phosphate_c0[c3] -> (1) Phosphate_c0[c3] + (1) L-Histidinol_c0[c3]	0.00269184	No
rxn02159	Histidine metabolism	L-Histidinol:NAD+ oxidoreductase	(1) NAD_c0[c3] + (1) L-Histidinol_c0[c3] <-> (1) NADH_c0[c3] + (1) H+_c0[c3] + (1) L-Histidinal_c0[c3]	0.00269184	No
rxn02835	Histidine metabolism	1-(5-phospho-D-ribosyl)-AMP 1,6-hydrolase	(1) H2O_c0[c3] + (1) Phosphoribosyl-AMP_c0[c3] <-> (1) phosphoribosylformiminoaicar-phosphate_c0[c3]	0.00269184	No
rxn03175	Histidine metabolism	N-(5'-Phospho-D-ribosylformimino)-5-amino-1- (5''-phospho-D-ribosyl)-4-imidazolecarboxamide ketol-isomerase	(1) H+_c0[c3] + (1) phosphoribosylformiminoaicar-phosphate_c0[c3] <-> (1) phosphoribulosylformimino-AICAR-phosphate_c0[c3]	0.00269184	No
rxn03135	Histidine metabolism	R04558	(1) L-Glutamate_c0[c3] + (2) H+_c0[c3] + (1) D-erythro-imidazol-glycerol-phosphate_c0[c3] + (1) AICAR_c0[c3] <- (1) L-Glutamine_c0[c3] + (1) phosphoribulosylformimino-AICAR-phosphate_c0[c3]	-0.00269184	No
rxn00863	Histidine metabolism	L-histidinal:NAD+ oxidoreductase	(1) H2O_c0[c3] + (1) NAD_c0[c3] + (1) L-Histidinal_c0[c3] -> (1) NADH_c0[c3] + (2) H+_c0[c3] + (1) L-Histidine_c0[c3]	0.00269184	No
rxn02834	Histidine metabolism	Phosphoribosyl-ATP pyrophosphohydrolase	(1) H2O_c0[c3] + (1) Phosphoribosyl-ATP_c0[c3] -> (1) PPi_c0[c3] + (2) H+_c0[c3] + (1) Phosphoribosyl-AMP_c0[c3]	0.00269184	No
rxn02473	Histidine metabolism	D-erythro-1-(Imidazol-4-yl)glycerol 3-phosphate hydro-lyase	(1) D-erythro-imidazol-glycerol-phosphate_c0[c3] -> (1) H2O_c0[c3] + (1) imidazole acetol-phosphate_c0[c3]	0.00269184	No

rxn03181	Lipopolysaccharide biosynthesis	ATP:2,3,2',3'-tetrakis(3-hydroxytetradecanoyl)-D-glucosaminyl-beta-D-1,6-glucosaminyl-alpha-phosphate 4-O'-phosphotransferase	(1) ATP_c0[c3] + (1) Lipid A disaccharide_c0[c3] <-> (1) ADP_c0[c3] + (1) H+_c0[c3] + (1) Lipid IV(A)_c0[c3]	0.000849375	No
rxn02331	Lipopolysaccharide biosynthesis	phosphoenolpyruvate:D-arabinose-5-phosphate C-(1-carboxyvinyl)transferase (phosphate-hydrolysing, 2-carboxy-2-oxoethyl-forming)	(1) Phosphate_c0[c3] + (1) 3-Deoxy-D-manno-octulosonate8-phosphate_c0[c3] <- (1) H2O_c0[c3] + (1) Phosphoenolpyruvate_c0[c3] + (1) D-Arabinose5-phosphate_c0[c3]	-0.00254813	No
rxn03159	Lipopolysaccharide biosynthesis	UDP-2,3-bis(3-hydroxytetradecanoyl)glucosamine:2,3-bis-(3-hydroxytetradecanoyl)-alpha-D-glucosaminyl-1-phosphate 2,3-bis(3-hydroxytetradecanoyl)-glucosaminyltransferase	(1) UDP-2,3-bis(3-hydroxytetradecanoyl)glucosamine_c0[c3] + (1) Lipid X_c0[c3] <-> (1) UDP_c0[c3] + (1) Lipid A disaccharide_c0[c3]	0.000849375	No
rxn06723	Lipopolysaccharide biosynthesis	(3R)-3-hydroxymyristoyl-[acyl-carrier protein]:UDP-3-O-[(3R)-3-hydroxymyristoyl]-alpha-D-glucosamine N-acetyltransferase	(1) UDP-2,3-bis(3-hydroxytetradecanoyl)glucosamine_c0[c3] + (1) ACP_c0[c3] <- (1) UDP-3-O-(beta-hydroxymyristoyl)-D-glucosamine_c0[c3] + (1) HMA_c0[c3]	-0.00169875	No
rxn03146	Lipopolysaccharide biosynthesis	UDP-3-O-[(3R)-3-hydroxymyristoyl]-N-acetylglucosamine amidohydrolase	(1) H2O_c0[c3] + (1) UDP-3-O-(beta-hydroxymyristoyl)-N-acetylglucosamine_c0[c3] <-> (1) Acetate_c0[c3] + (1) UDP-3-O-(beta-hydroxymyristoyl)-D-glucosamine_c0[c3]	0.00169875	No
rxn02405	Lipopolysaccharide biosynthesis	CTP:3-deoxy-D-manno-octulosonate cytidylyltransferase	(1) CTP_c0[c3] + (1) KDO_c0[c3] -> (1) PPi_c0[c3] + (1) H+_c0[c3] + (1) CMP-KDO_c0[c3]	0.00254813	No
rxn06865	Lipopolysaccharide biosynthesis	R05146	(1) Lauroyl-KDO2-lipid IV(A)_c0[c3] + (1) ACP_c0[c3] <- (1) kdo2-lipid iva_c0[c3] + (1) Dodecanoyl-ACP_c0[c3]	-0.000849375	No
rxn03439	Lipopolysaccharide biosynthesis	CMP-3-deoxy-D-manno-oct-2-ulosonate:(KDO)-lipid IVA 3-deoxy-D-manno-oct-2-ulosonate transferase	(1) CMP-KDO_c0[c3] + (1) KDO-lipid IV(A)_c0[c3] <-> (1) CMP_c0[c3] + (1) H+_c0[c3] + (1) kdo2-lipid iva_c0[c3]	0.000849375	No
rxn06729	Lipopolysaccharide biosynthesis	(R)-3-Hydroxytetradecanoly-[acyl-carrier-protein]:UDP-N-acetyl-glucosamine 3-O-(3-hydroxytetradecanoyl)transferase	(1) UDP-N-acetylglucosamine_c0[c3] + (1) H+_c0[c3] + (1) HMA_c0[c3] -> (1) UDP-3-O-(beta-hydroxymyristoyl)-N-acetylglucosamine_c0[c3] + (1) ACP_c0[c3]	0.00169875	No
rxn03182	Lipopolysaccharide biosynthesis	CMP-3-deoxy-D-manno-oct-2-ulosonate:lipid IVA 3-deoxy-D-manno-oct-2-ulosonate transferase	(1) CMP-KDO_c0[c3] + (1) Lipid IV(A)_c0[c3] <-> (1) CMP_c0[c3] + (1) H+_c0[c3] + (1) KDO-lipid IV(A)_c0[c3]	0.000849375	No
rxn02011	Lysine biosynthesis|Peptidoglycan biosynthesis	UDP-N-acetylmuramoyl-L-alanyl-D-glutamate:(L)-meso-2,6-diaminoheptanedioate gamma-ligase (ADP-forming)	(1) ATP_c0[c3] + (1) meso-2,6-Diaminopimelate_c0[c3] + (1) UDP-N-acetylmuramoyl-L-alanyl-D-glutamate_c0[c3] -> (1) ADP_c0[c3] + (1) Phosphate_c0[c3] + (1) H+_c0[c3] + (1) UDP-N-acetylmuramoyl-L-alanyl-D-gamma-glutamyl-meso-2-6-diaminopimelate_c0[c3]	0.000849375	No

rxn02988	Nicotinate and nicotinamide metabolism	glycerone phosphate:iminosuccinate alkyltransferase (cyclizing)	(2) H2O_c0[c3] + (1) Phosphate_c0[c3] + (1) Quinolinate_c0[c3] <- (1) Glycerone-phosphate_c0[c3] + (1) Iminoaspartate_c0[c3]	-0.000210316	No
rxn02402	Nicotinate and nicotinamide metabolism	Nicotinate-nucleotide:pyrophosphate phosphoribosyltransferase (carboxylating)	(1) CO2_c0[c3] + (1) PPi_c0[c3] + (1) Nicotinate ribonucleotide_c0[c3] <- (1) H+_c0[c3] + (1) PRPP_c0[c3] + (1) Quinolinate_c0[c3]	-0.000210316	No
rxn02155	Nicotinate and nicotinamide metabolism	ATP:nicotinamide-nucleotide adenylyltransferase	(1) ATP_c0[c3] + (1) Nicotinate ribonucleotide_c0[c3] <-> (1) PPi_c0[c3] + (1) Deamido-NAD_c0[c3]	0.000210316	No
rxn00083	Nicotinate and nicotinamide metabolism	NADPH:NAD+ oxidoreductase	(1) NAD_c0[c3] + (1) NADPH_c0[c3] <-> (1) NADH_c0[c3] + (1) NADP_c0[c3]	-0.110598	Yes
rxn01265	Nicotinate and nicotinamide metabolism	Nicotinate D-ribonucleotide:diphosphate phosphoribosyltransferase	(1) PPi_c0[c3] + (1) Nicotinate ribonucleotide_c0[c3] <-> (1) PRPP_c0[c3] + (1) Niacin_c0[c3]	-0.000105158	No
rxn00077	Nicotinate and nicotinamide metabolism	ATP:NAD+ 2'-phosphotransferase	(1) ATP_c0[c3] + (1) NAD_c0[c3] <-> (1) NADP_c0[c3] + (1) ADP_c0[c3] + (1) H+_c0[c3]	0.000105158	No
rxn00338	Nicotinate and nicotinamide metabolism	L-aspartate:oxygen oxidoreductase	(1) O2_c0[c3] + (1) L-Aspartate_c0[c3] -> (1) H2O2_c0[c3] + (1) H+_c0[c3] + (1) Iminoaspartate_c0[c3]	0.000210316	No
rxn00138	Nicotinate and nicotinamide metabolism	deamido-NAD+:ammonia ligase (AMP-forming)	(1) ATP_c0[c3] + (1) NH3_c0[c3] + (1) Deamido-NAD_c0[c3] -> (1) NAD_c0[c3] + (1) PPi_c0[c3] + (1) AMP_c0[c3] + (2) H+_c0[c3]	0.000210316	No
rxn00102	Nitrogen metabolism	carbonate hydro-lyase (carbon-dioxide-forming)	(1) H+_c0[c3] + (1) H2CO3_c0[c3] <-> (1) H2O_c0[c3] + (1) CO2_c0[c3]	-0.0981713	No
rxn00907	One carbon pool by folate|Carbon fixation pathways in prokaryotes	5,10-methylenetetrahydrofolate:NADP+ oxidoreductase	(1) NADP_c0[c3] + (1) 5-10-Methylenetetrahydrofolate_c0[c3] <-> (1) NADPH_c0[c3] + (1) 5-10-Methenyltetrahydrofolate_c0[c3]	0.0259689	No
rxn01211	One carbon pool by folate|Carbon fixation pathways in prokaryotes	5,10-Methenyltetrahydrofolate 5-hydrolase (decyclizing)	(1) H2O_c0[c3] + (1) 5-10-Methenyltetrahydrofolate_c0[c3] <-> (1) H+_c0[c3] + (1) 10-Formyltetrahydrofolate_c0[c3]	0.0259689	No
rxn00690	One carbon pool by folate|Carbon fixation pathways in prokaryotes	Formate:tetrahydrofolate ligase (ADP-forming)	(1) ATP_c0[c3] + (1) Formate_c0[c3] + (1) Tetrahydrofolate_c0[c3] <-> (1) ADP_c0[c3] + (1) Phosphate_c0[c3] + (1) 10-Formyltetrahydrofolate_c0[c3]	-0.00200206	No
rxn00686	One carbon pool by folate|Folate biosynthesis	5,6,7,8-tetrahydrofolate:NADP+ oxidoreductase	(1) NADP_c0[c3] + (1) Tetrahydrofolate_c0[c3] <-> (1) NADPH_c0[c3] + (1) H+_c0[c3] + (1) Dihydrofolate_c0[c3]	-0.000749424	No
rxn04954	One carbon pool by folate|Methane metabolism|Carbon fixation pathways in prokaryotes	5-methyltetrahydrofolate:NAD+ oxidoreductase	(1) NAD_c0[c3] + (1) 5-Methyltetrahydrofolate_c0[c3] <-> (1) NADH_c0[c3] + (1) H+_c0[c3] + (1) 5-10-Methylenetetrahydrofolate_c0[c3]	-0.00518148	No
rxn00898	Pantothenate and CoA biosynthesis	2,3-Dihydroxy-3-methylbutanoate hydro-lyase	(1) 2,3-Dihydroxy-isovalerate_c0[c3] -> (1) H2O_c0[c3] + (1) 3-Methyl-2-oxobutanoate_c0[c3]	0.0121724	No
rxn12512	Pantothenate and CoA biosynthesis	(R)-4'-Phosphopantothenate:L-cysteine ligase	(1) ATP_c0[c3] + (1) L-Cysteine_c0[c3] + (1) 4-phosphopantothenate_c0[c3] -> (1) PPi_c0[c3] + (1) AMP_c0[c3] + (2) H+_c0[c3] + (1) (R)-4'-Phosphopantothenoyl-L-cysteine_c0[c3]	0.000210316	No
rxn00100	Pantothenate and CoA biosynthesis	ATP:dephospho-CoA 3'-phosphotransferase	(1) ATP_c0[c3] + (1) Dephospho-CoA_c0[c3] -> (1) ADP_c0[c3] + (1) CoA_c0[c3] + (1) H+_c0[c3]	0.000210316	No

rxn02175	Pantothenate and CoA biosynthesis	ATP:pantetheine-4'-phosphate adenylyltransferase	(1) ATP_c0[c3] + (1) Phosphopantetheine_c0[c3] <-> (1) PPi_c0[c3] + (1) Dephospho-CoA_c0[c3]	0.000210316	No
rxn06023	Pantothenate and CoA biosynthesis	CoA:apo-[acyl-carrier-protein] pantetheinephosphotransferase	(1) CoA_c0[c3] + (1) apo-ACP_c0[c3] <-> (1) Adenosine 3-5-bisphosphate_c0[c3] + (1) ACP_c0[c3]	0.000105158	No
rxn12510	Pantothenate and CoA biosynthesis	ATP:pantothenate 4'-phosphotransferase	(1) ATP_c0[c3] + (1) PAN_c0[c3] <-> (1) ADP_c0[c3] + (1) H+_c0[c3] + (1) 4-phosphopantothenate_c0[c3]	0.000210316	No
rxn01790	Pantothenate and CoA biosynthesis	(R)-Pantoate:NADP+ 2-oxidoreductase	(1) NADP_c0[c3] + (1) Pantoate_c0[c3] <-> (1) NADPH_c0[c3] + (1) H+_c0[c3] + (1) 2-Dehydropantoate_c0[c3]	-0.000210316	Yes
rxn00912	Pantothenate and CoA biosynthesis	5,10-Methylenetetrahydrofolate: 3-methyl-2-oxobutanoate hydroxymethyltransferase	(1) H2O_c0[c3] + (1) 3-Methyl-2-oxobutanoate_c0[c3] + (1) 5-10-Methylenetetrahydrofolate_c0[c3] <-> (1) Tetrahydrofolate_c0[c3] + (1) 2-Dehydropantoate_c0[c3]	0.000210316	No
rxn02341	Pantothenate and CoA biosynthesis	N-[(R)-4'-Phosphopantothenoyl]-L-cysteine carboxy-lyase	(1) H+_c0[c3] + (1) (R)-4'-Phosphopantothenoyl-L-cysteine_c0[c3] -> (1) CO2_c0[c3] + (1) Phosphopantetheine_c0[c3]	0.000210316	No
rxn02186	Pantothenate and CoA biosynthesis	2,3-Dihydroxy-3-methylbutanoate:NADP+ oxidoreductase (isomerizing)	(1) NADPH_c0[c3] + (1) H+_c0[c3] + (1) ALCTT_c0[c3] <-> (1) NADP_c0[c3] + (1) 2,3-Dihydroxy-isovalerate_c0[c3]	0.0121724	Yes
rxn00196	Pentose and glucuronate interconversions|Ascorbate and aldarate metabolism	2,5-dioxopentanoate:NADP+ 5-oxidoreductase	(1) H2O_c0[c3] + (1) NADP_c0[c3] + (1) 2,5-Dioxopentanoate_c0[c3] -> (1) NADPH_c0[c3] + (1) 2-Oxoglutarate_c0[c3] + (2) H+_c0[c3]	0.0120935	No
rxn00213	Pentose and glucuronate interconversions|Galactose metabolism|Starch and sucrose metabolism|Amino sugar and nucleotide sugar metabolism	UTP:alpha-D-glucose-1-phosphate uridylyltransferase	(1) UTP_c0[c3] + (1) Glucose-1-phosphate_c0[c3] <-> (1) PPi_c0[c3] + (1) UDP-glucose_c0[c3]	-0.000849375	No
rxn01477	Pentose phosphate pathway	6-Phospho-D-gluconate hydro-lyase(2-dehydro-3-deoxy-6-phospho-D-gluconate-forming)	(1) 6-Phospho-D-gluconate_c0[c3] -> (1) H2O_c0[c3] + (1) 2-Keto-3-deoxy-6-phosphogluconate_c0[c3]	0.0486646	No
rxn01476	Pentose phosphate pathway	6-Phospho-D-glucono-1,5-lactone lactonohydrolase	(1) H2O_c0[c3] + (1) 6-phospho-D-glucono-1-5-lactone_c0[c3] -> (1) H+_c0[c3] + (1) 6-Phospho-D-gluconate_c0[c3]	0.0190575	No
rxn01333	Pentose phosphate pathway	sedoheptulose-7-phosphate:D-glyceraldehyde-3-phosphate glyceronetransferase	(1) Glyceraldehyde3-phosphate_c0[c3] + (1) Sedoheptulose7-phosphate_c0[c3] <-> (1) D-fructose-6-phosphate_c0[c3] + (1) D-Erythrose4-phosphate_c0[c3]	-0.0583027	No
rxn00777	Pentose phosphate pathway|Carbon fixation in photosynthetic organisms	D-ribose-5-phosphate aldose-ketose-isomerase	(1) ribose-5-phosphate_c0[c3] <-> (1) D-Ribulose5-phosphate_c0[c3]	0.032681	No
rxn01200	Pentose phosphate pathway|Carbon fixation in photosynthetic organisms	Sedoheptulose-7-phosphate:D-glyceraldehyde-3-phosphate glycolaldehyde transferase	(1) Glyceraldehyde3-phosphate_c0[c3] + (1) Sedoheptulose7-phosphate_c0[c3] <-> (1) ribose-5-phosphate_c0[c3] + (1) D-Xylulose5-phosphate_c0[c3]	0.0549052	Yes
rxn01187	Pentose phosphate pathway|Methane	D-Xylulose 5-phosphate D-glyceraldehyde-3-phosphate-lyase (adding	(1) Phosphate_c0[c3] + (1) D-Xylulose5-phosphate_c0[c3] -> (1) H2O_c0[c3] + (1) Glyceraldehyde3-	0.0549052	No

	metabolism|Carbon fixation in photosynthetic organisms	phosphate; acetyl-phosphate-forming)	phosphate_c0[c3] + (1) Acetylphosphate_c0[c3]		
rxn03884	Pentose phosphate pathway|Pentose and glucuronate interconversions	2-dehydro-3-deoxy-D-gluconate-6-phosphate D-glyceraldehyde-3-phosphate-lyase	(1) 2-Keto-3-deoxy-6-phosphogluconate_c0[c3] <-> (1) Pyruvate_c0[c3] + (1) Glyceraldehyde3-phosphate_c0[c3]	0.0486646	No
rxn00770	Pentose phosphate pathway|Purine metabolism	ATP:D-ribose-5-phosphate diphosphotransferase	(1) ATP_c0[c3] + (1) ribose-5-phosphate_c0[c3] <-> (1) AMP_c0[c3] + (1) H+_c0[c3] + (1) PRPP_c0[c3]	0.0222242	No
rxn00778	Pentose phosphate pathway|Purine metabolism	D-Ribose 1,5-phosphomutase	(1) Ribose 1-phosphate_c0[c3] <-> (1) ribose-5-phosphate_c0[c3]	0.000630948	No
rxn03408	Peptidoglycan biosynthesis	UDP-N-acetyl-D-glucosamine:undecaprenyl-diphospho-N-acetylmuramoyl-L-alanyl-gamma-D-glutamyl-meso-2,6-diaminopimeloyl-D-alanyl-D-alanine 4-beta-N-acetylglucosaminlytransferase	(1) UDP-N-acetylglucosamine_c0[c3] + (1) Undecaprenyl-diphospho-N-acetylmuramoyl-L-alanyl-D-glutamyl-meso-2-6-diaminopimeloyl-D-alanyl-D-alanine_c0[c3] <-> (1) UDP_c0[c3] + (1) Undecaprenyl-diphospho-N-acetylmuramoyl--N-acetylglucosamine-L-ala-D-glu-meso-2-6-diaminopimeloyl-D-ala-D-ala_c0[c3]	0.000849375	No
rxn03904	Peptidoglycan biosynthesis	UDP-N-acetylmuramoyl-L-alanyl-gamma-D-glutamyl-meso-2,6-diaminopimeloyl-D-alanyl-D-alanine:undecaprenyl-phosphate phospho-N-acetylmuramoyl-pentapeptide-transferase	(1) Undecaprenylphosphate_c0[c3] + (1) UDP-N-acetylmuramoyl-L-alanyl-D-glutamyl-6-carboxy-L-lysyl-D-alanyl- D-alanine_c0[c3] <-> (1) UMP_c0[c3] + (1) Undecaprenyl-diphospho-N-acetylmuramoyl-L-alanyl-D-glutamyl-meso-2-6-diaminopimeloyl-D-alanyl-D-alanine_c0[c3]	0.000849375	No
rxn03901	Peptidoglycan biosynthesis	undecaprenyl-diphosphate phosphohydrolase	(1) H2O_c0[c3] + (1) Bactoprenyl diphosphate_c0[c3] -> (1) Phosphate_c0[c3] + (2) H+_c0[c3] + (1) Undecaprenylphosphate_c0[c3]	0.000849375	No
rxn01739	Phenylalanine, tyrosine and tryptophan biosynthesis	ATP:shikimate 3-phosphotransferase	(1) ATP_c0[c3] + (1) Shikimate_c0[c3] <-> (1) ADP_c0[c3] + (1) H+_c0[c3] + (1) 3-phosphoshikimate_c0[c3]	0.00223395	No
rxn02212	Phenylalanine, tyrosine and tryptophan biosynthesis	2-Dehydro-3-deoxy-D-arabino-heptonate 7-phosphate phosphate-lyase (cyclyzing)	(1) DAHP_c0[c3] -> (1) Phosphate_c0[c3] + (1) 5-Dehydroquinate_c0[c3]	0.00223395	No
rxn01740	Phenylalanine, tyrosine and tryptophan biosynthesis	Shikimate:NADP+ 3-oxidoreductase	(1) NADP_c0[c3] + (1) Shikimate_c0[c3] <-> (1) NADPH_c0[c3] + (1) H+_c0[c3] + (1) 3-Dehydroshikimate_c0[c3]	-0.00223395	No
rxn01255	Phenylalanine, tyrosine and tryptophan biosynthesis	5-O-(1-Carboxyvinyl)-3-phosphoshikimate phosphate-lyase (chorismate-forming)	(1) 5-O--1-Carboxyvinyl-3-phosphoshikimate_c0[c3] -> (1) Phosphate_c0[c3] + (1) Chorismate_c0[c3]	0.00223395	No
rxn02508	Phenylalanine, tyrosine and tryptophan biosynthesis	N-(5-Phospho-beta-D-ribosyl)anthranilate ketol-isomerase	(1) N-5-phosphoribosyl-anthranilate_c0[c3] <-> (1) 1-(2-carboxyphenylamino)-1-deoxyribulose 5-phosphate_c0[c3]	0.00160301	No
rxn00726	Phenylalanine, tyrosine and tryptophan biosynthesis	chorismate pyruvate-lyase (amino-accepting; anthranilate-forming)	(1) NH3_c0[c3] + (1) Chorismate_c0[c3] -> (1) H2O_c0[c3] + (1) Pyruvate_c0[c3] + (1) H+_c0[c3] + (1) Anthranilate_c0[c3]	0.00160301	No
rxn00791	Phenylalanine, tyrosine and tryptophan biosynthesis	N-(5-Phospho-D-ribosyl)anthranilate:pyrophosphate phosphoribosyl-transferase	(1) PPi_c0[c3] + (1) H+_c0[c3] + (1) N-5-phosphoribosyl-anthranilate_c0[c3] <- (1) Anthranilate_c0[c3] + (1) PRPP_c0[c3]	-0.00160301	No
rxn02476	Phenylalanine, tyrosine and tryptophan biosynthesis	Phosphoenolpyruvate:3-phosphoshikimate 5-O-(1-carboxyvinyl)-transferase	(1) Phosphoenolpyruvate_c0[c3] + (1) 3-phosphoshikimate_c0[c3] <-> (1) Phosphate_c0[c3] + (1) 5-O--1-	0.00223395	No

			Carboxyvinyl-3-phosphoshikimate_c0[c3]		
rxn02213	Phenylalanine, tyrosine and tryptophan biosynthesis	3-Dehydroquinate hydro-lyase	(1) 5-Dehydroquinate_c0[c3] -> (1) H2O_c0[c3] + (1) 3-Dehydroshikimate_c0[c3]	0.00223395	No
rxn01332	Phenylalanine, tyrosine and tryptophan biosynthesis	Phosphoenolpyruvate:D-erythrose-4-phosphate C-(1-carboxyvinyl)transferase (phosphate hydrolysing, 2-carboxy-2-oxoethyl-forming)	(1) H2O_c0[c3] + (1) Phosphoenolpyruvate_c0[c3] + (1) D-Erythrose4-phosphate_c0[c3] -> (1) Phosphate_c0[c3] + (1) DAHP_c0[c3]	0.00223395	No
rxn02507	Phenylalanine, tyrosine and tryptophan biosynthesis	1-(2-Carboxyphenylamino)-1-deoxy-D-ribulose-5-phosphate carboxy-lyase(cyclizing)	(1) H+_c0[c3] + (1) 1-(2-carboxyphenylamino)-1-deoxyribulose 5-phosphate_c0[c3] -> (1) H2O_c0[c3] + (1) CO2_c0[c3] + (1) Indoleglycerol phosphate_c0[c3]	0.00160301	No
rxn00060	Porphyrin and chlorophyll metabolism	porphobilinogen:(4-[2-carboxyethyl]-3-[carboxymethyl]pyrrol-2-yl)methyltransferase (hydrolysing)	(1) H2O_c0[c3] + (4) Porphobilinogen_c0[c3] -> (4) NH3_c0[c3] + (1) Hydroxymethylbilane_c0[c3]	0.000210316	No
rxn02264	Porphyrin and chlorophyll metabolism	Hydroxymethylbilane hydro-lyase(cyclizing)	(1) Hydroxymethylbilane_c0[c3] <-> (1) H2O_c0[c3] + (1) UroporphyrinogenIII_c0[c3]	0.000210316	No
rxn00029	Porphyrin and chlorophyll metabolism	5-aminolevulinate hydro-lyase (adding 5-aminolevulinate and cyclizing; porphobilinogen-forming)	(2) 5-Aminolevulinate_c0[c3] -> (2) H2O_c0[c3] + (1) H+_c0[c3] + (1) Porphobilinogen_c0[c3]	0.000841263	No
rxn03537	Porphyrin and chlorophyll metabolism	R05222	(1) GTP_c0[c3] + (1) Adenosyl cobinamide phosphate_c0[c3] <-> (1) PPi_c0[c3] + (1) H+_c0[c3] + (1) Adenosylcobinamide-GDP_c0[c3]	0.000105158	No
rxn04413	Porphyrin and chlorophyll metabolism	R06558	(1) GTP_c0[c3] + (1) Adenosyl cobinamide_c0[c3] <-> (1) GDP_c0[c3] + (1) H+_c0[c3] + (1) Adenosyl cobinamide phosphate_c0[c3]	0.000105158	No
rxn05029	Porphyrin and chlorophyll metabolism	ATP:cobinamide Cobeta-adenosyltransferase	(1) ATP_c0[c3] + (1) H+_c0[c3] + (1) Cobinamide_c0[c3] <-> (1) Triphosphate_c0[c3] + (1) Adenosyl cobinamide_c0[c3]	0.000105158	No
rxn02303	Porphyrin and chlorophyll metabolism	Coproporphyrinogen:oxygen oxidoreductase(decarboxylating)	(1) O2_c0[c3] + (2) H+_c0[c3] + (1) CoproporphyrinogenIII_c0[c3] <-> (2) H2O_c0[c3] + (2) CO2_c0[c3] + (1) ProtoporphyrinogenIX_c0[c3]	0.000105158	No
rxn03538	Porphyrin and chlorophyll metabolism	R05223	(1) alpha-Ribazole_c0[c3] + (1) Adenosylcobinamide-GDP_c0[c3] <-> (1) H+_c0[c3] + (1) GMP_c0[c3] + (1) Calomide_c0[c3]	0.000105158	No
rxn00056	Porphyrin and chlorophyll metabolism	Fe(II):oxygen oxidoreductase	(1) O2_c0[c3] + (4) H+_c0[c3] + (4) Fe2+_c0[c3] <-> (2) H2O_c0[c3] + (4) fe3_c0[c3]	-7.88684E-05	No
rxn02897	Porphyrin and chlorophyll metabolism	Nicotinate-nucleotide:dimethylbenzimidazole phospho-D-ribosyltransferase	(1) Nicotinate ribonucleotide_c0[c3] + (1) Dimethylbenzimidazole_c0[c3] <-> (1) H+_c0[c3] + (1) Niacin_c0[c3] + (1) alpha-Ribazole 5'-phosphate_c0[c3]	0.000105158	No
rxn02056	Porphyrin and chlorophyll metabolism	S-Adenosyl-L-methionine:uroporphyrin-III C-methyltransferase	(2) H+_c0[c3] + (1) Siroheme_c0[c3] <-> (1) Sirohydrochlorin_c0[c3] + (1) Fe2+_c0[c3]	-0.000105158	No
rxn02288	Porphyrin and chlorophyll metabolism	Uroporphyrinogen-III carboxy-lyase	(4) H+_c0[c3] + (1) UroporphyrinogenIII_c0[c3] <-> (4)	0.000105158	No

			CO2_c0[c3] + (1) CoproporphyrinogenIII_c0[c3]		
rxn00224	Porphyrin and chlorophyll metabolism	protoheme ferro-lyase (protoporphyrin-forming)	(1) Protoporphyrin_c0[c3] + (1) Fe2+_c0[c3] <-> (1) Heme_c0[c3] + (2) H+_c0[c3]	0.000105158	No
rxn00097	Purine metabolism	ATP:AMP phosphotransferase	(1) ATP_c0[c3] + (1) AMP_c0[c3] + (1) H+_c0[c3] <-> (2) ADP_c0[c3]	-0.0482898	No
rxn00131	Purine metabolism	AMP phosphoribohydrolase	(1) H2O_c0[c3] + (1) AMP_c0[c3] <-> (1) ribose-5-phosphate_c0[c3] + (1) Adenine_c0[c3]	-0.000630948	No
rxn01445	Purine metabolism	2'-Deoxyguanosine 5'-monophosphate phosphohydrolase	(1) H2O_c0[c3] + (1) dGMP_c0[c3] -> (1) Phosphate_c0[c3] + (1) Deoxyguanosine_c0[c3]	0.00065408	No
rxn00917	Purine metabolism	Xanthosine-5'-phosphate:L-glutamine amido-ligase (AMP-forming)	(1) H2O_c0[c3] + (1) ATP_c0[c3] + (1) L-Glutamine_c0[c3] + (1) XMP_c0[c3] -> (1) PPi_c0[c3] + (1) AMP_c0[c3] + (1) L-Glutamate_c0[c3] + (3) H+_c0[c3] + (1) GMP_c0[c3]	0.00577837	No
rxn01352	Purine metabolism	dGTP triphosphohydrolase	(1) H2O_c0[c3] + (1) dGTP_c0[c3] <-> (1) Deoxyguanosine_c0[c3] + (1) Triphosphate_c0[c3]	-0.00065408	No
rxn01509	Purine metabolism	ATP:dGMP phosphotransferase	(1) ATP_c0[c3] + (1) H+_c0[c3] + (1) dGMP_c0[c3] <-> (1) ADP_c0[c3] + (1) dGDP_c0[c3]	-0.00065408	No
rxn03147	Purine metabolism	1-(5-Phosphoribosyl)-5-amino-4-carboxyimidazole:L-aspartate ligase (ADP-forming)	(1) ATP_c0[c3] + (1) L-Aspartate_c0[c3] + (1) 5'-Phosphoribosyl-4-carboxy-5-aminoimidazole_c0[c3] -> (1) ADP_c0[c3] + (1) Phosphate_c0[c3] + (1) H+_c0[c3] + (1) SAICAR_c0[c3]	0.0105849	No
rxn01138	Purine metabolism	Adenosine:phosphate alpha-D-ribosyltransferase	(1) Phosphate_c0[c3] + (1) Adenosine_c0[c3] <-> (1) Adenine_c0[c3] + (1) Ribose 1-phosphate_c0[c3]	0.000630948	No
rxn00832	Purine metabolism	IMP 1,2-hydrolase (decyclizing)	(1) H2O_c0[c3] + (1) IMP_c0[c3] <-> (1) FAICAR_c0[c3]	-0.0132767	No
rxn00515	Purine metabolism	ATP:IDP phosphotransferase	(1) ATP_c0[c3] + (1) IDP_c0[c3] <-> (1) ADP_c0[c3] + (1) ITP_c0[c3]	0.0087277	Yes
rxn02895	Purine metabolism	5-Phospho-D-ribosylamine:glycine ligase (ADP-forming)	(1) ATP_c0[c3] + (1) Glycine_c0[c3] + (1) 5-Phosphoribosylamine_c0[c3] -> (1) ADP_c0[c3] + (1) Phosphate_c0[c3] + (1) H+_c0[c3] + (1) GAR_c0[c3]	0.0105849	No
rxn05231	Purine metabolism	2'-Deoxyadenosine 5'-diphosphate:oxidized-thioredoxin 2'-oxidoreductase	(1) ADP_c0[c3] + (1) trdrd_c0[c3] -> (1) H2O_c0[c3] + (1) dADP_c0[c3] + (1) trdox_c0[c3]	0.00043395	No
rxn03136	Purine metabolism	1-(5'-Phosphoribosyl)-5-amino-4-(N-succinocarboxamide)-imidazole AMP-lyase	(1) SAICAR_c0[c3] <-> (1) H+_c0[c3] + (1) Fumarate_c0[c3] + (1) AICAR_c0[c3]	0.0105849	No
rxn02937	Purine metabolism	2-(Formamido)-N1-(5-phosphoribosyl)acetamidine cyclo-ligase (ADP-forming)	(1) ATP_c0[c3] + (1) 5'-Phosphoribosylformylglycinamidine_c0[c3] <-> (1) ADP_c0[c3] + (1) Phosphate_c0[c3] + (1) H+_c0[c3] + (1) AIR_c0[c3]	0.0105849	No
rxn00834	Purine metabolism	IMP:NAD+ oxidoreductase	(1) H2O_c0[c3] + (1) NAD_c0[c3] + (1) IMP_c0[c3] <-> (1) NADH_c0[c3] + (1) H+_c0[c3] + (1) XMP_c0[c3]	0.00577837	No
rxn03084	Purine metabolism	5'-Phosphoribosylformylglyci	(1) H2O_c0[c3] + (1) ATP_c0[c3] + (1) L-Glutamine_c0[c3] + (1) N-Formyl-GAR_c0[c3] -> (1) ADP_c0[c3] + (1) Phosphate_c0[c3] + (1) L-	0.0105849	No

		namide:L-glutamine amido-ligase (ADP-forming)	Glutamate_c0[c3] + (1) H+_c0[c3] + (1) 5'-Phosphoribosylformylglycinamidine_c0[c3]		
rxn05233	Purine metabolism	2'-Deoxyguanosine 5'-diphosphate:oxidized-thioredoxin 2'-oxidoreductase	(1) GDP_c0[c3] + (1) trdrd_c0[c3] -> (1) H2O_c0[c3] + (1) dGDP_c0[c3] + (1) trdox_c0[c3]	0.00065408	No
rxn00239	Purine metabolism	ATP:GMP phosphotransferase	(1) ATP_c0[c3] + (1) H+_c0[c3] + (1) GMP_c0[c3] <-> (1) ADP_c0[c3] + (1) GDP_c0[c3]	0.00588353	No
rxn00839	Purine metabolism	ATP:dADP phosphotransferase	(1) ATP_c0[c3] + (1) dADP_c0[c3] <-> (1) ADP_c0[c3] + (1) dATP_c0[c3]	0.00043395	Yes
rxn00800	Purine metabolism|Alanine, aspartate and glutamate metabolism	N6-(1,2-dicarboxyethyl)AMP AMP-lyase (fumarate-forming)	(1) Adenylosuccinate_c0[c3] <-> (1) AMP_c0[c3] + (1) Fumarate_c0[c3]	0.00749837	No
rxn00790	Purine metabolism|Alanine, aspartate and glutamate metabolism	5-phosphoribosylamine:diphosphate phospho-alpha-D-ribosyltransferase (glutamate-amidating)	(1) PPi_c0[c3] + (1) L-Glutamate_c0[c3] + (1) H+_c0[c3] + (1) 5-Phosphoribosylamine_c0[c3] <- (1) H2O_c0[c3] + (1) L-Glutamine_c0[c3] + (1) PRPP_c0[c3]	-0.0105849	No
rxn00838	Purine metabolism|Alanine, aspartate and glutamate metabolism	IMP:L-aspartate ligase (GDP-forming)	(1) GTP_c0[c3] + (1) L-Aspartate_c0[c3] + (1) IMP_c0[c3] -> (1) Phosphate_c0[c3] + (1) GDP_c0[c3] + (2) H+_c0[c3] + (1) Adenylosuccinate_c0[c3]	0.00749837	No
rxn00114	Purine metabolism|Arginine and proline metabolism|Nitrogen metabolism	ATP:carbamate phosphotransferase	(1) ATP_c0[c3] + (1) CO2_c0[c3] + (1) NH3_c0[c3] <-> (1) ADP_c0[c3] + (2) H+_c0[c3] + (1) Carbamoylphosphate_c0[c3]	0.00963969	No
rxn03137	Purine metabolism|One carbon pool by folate	10-Formyltetrahydrofolate:5'-phosphoribosyl-5-amino-4-imidazolecarboxamide formyltransferase	(1) 10-Formyltetrahydrofolate_c0[c3] + (1) AICAR_c0[c3] <-> (1) Tetrahydrofolate_c0[c3] + (1) FAICAR_c0[c3]	0.0132767	No
rxn03004	Purine metabolism|One carbon pool by folate	10-Formyltetrahydrofolate:5'-phosphoribosylglycinamide formyltransferase	(1) 10-Formyltetrahydrofolate_c0[c3] + (1) GAR_c0[c3] <-> (1) H+_c0[c3] + (1) Tetrahydrofolate_c0[c3] + (1) N-Formyl-GAR_c0[c3]	0.0105849	No
rxn00379	Purine metabolism|Sulfur metabolism	ATP:sulfate adenylyltransferase	(1) ATP_c0[c3] + (1) Sulfate_c0[c3] <-> (1) PPi_c0[c3] + (1) APS_c0[c3]	0.0201798	No
rxn00364	Pyrimidine metabolism	ATP:CMP phosphotransferase	(1) ATP_c0[c3] + (1) CMP_c0[c3] + (1) H+_c0[c3] <-> (1) ADP_c0[c3] + (1) CDP_c0[c3]	0.0274821	No
rxn01673	Pyrimidine metabolism	ATP:dCDP phosphotransferase	(1) ATP_c0[c3] + (1) dCDP_c0[c3] <-> (1) ADP_c0[c3] + (1) dCTP_c0[c3]	0.00065408	Yes
rxn00710	Pyrimidine metabolism	orotidine-5'-phosphate carboxy-lyase (UMP-forming)	(1) H+_c0[c3] + (1) Orotidylic acid_c0[c3] -> (1) CO2_c0[c3] + (1) UMP_c0[c3]	0.00702895	No
rxn01362	Pyrimidine metabolism	Orotidine-5'-phosphate:diphosphate phospho-alpha-D-ribosyl-transferase	(1) PPi_c0[c3] + (1) H+_c0[c3] + (1) Orotidylic acid_c0[c3] <- (1) PRPP_c0[c3] + (1) Orotate_c0[c3]	-0.00702895	No
rxn01465	Pyrimidine metabolism	(S)-dihydroorotate amidohydrolase	(1) H2O_c0[c3] + (1) S-Dihydroorotate_c0[c3] <-> (1) H+_c0[c3] + (1) N-Carbamoyl-L-aspartate_c0[c3]	-0.00702895	No
rxn00707	Pyrimidine metabolism	ITP:cytidine 5'-phosphotransferase	(1) ITP_c0[c3] + (1) Cytidine_c0[c3] <-> (1) CMP_c0[c3] + (1) H+_c0[c3] + (1) IDP_c0[c3]	0.0087277	No

rxn00708	Pyrimidine metabolism	Uridine 5'-monophosphate phosphohydrolase	(1) H2O_c0[c3] + (1) UMP_c0[c3] -> (1) Phosphate_c0[c3] + (1) Uridine_c0[c3]	0.0087277	No
rxn01678	Pyrimidine metabolism	ATP:dUDP phosphotransferase	(1) ATP_c0[c3] + (1) dUDP_c0[c3] <-> (1) ADP_c0[c3] + (1) dUTP_c0[c3]	0.00043395	Yes
rxn00410	Pyrimidine metabolism	UTP:ammonia ligase (ADP-forming)	(1) ATP_c0[c3] + (1) NH3_c0[c3] + (1) UTP_c0[c3] <-> (1) ADP_c0[c3] + (1) Phosphate_c0[c3] + (1) CTP_c0[c3] + (2) H+_c0[c3]	-0.00521741	No
rxn01512	Pyrimidine metabolism	ATP:dTDP phosphotransferase	(1) ATP_c0[c3] + (1) dTDP_c0[c3] <-> (1) ADP_c0[c3] + (1) TTP_c0[c3]	0.00128333	Yes
rxn01368	Pyrimidine metabolism	Cytidine aminohydrolase	(1) H2O_c0[c3] + (1) H+_c0[c3] + (1) Cytidine_c0[c3] <-> (1) NH3_c0[c3] + (1) Uridine_c0[c3]	-0.0087277	No
rxn06076	Pyrimidine metabolism	2'-Deoxycytidine diphosphate:oxidized-thioredoxin 2'-oxidoreductase	(1) H2O_c0[c3] + (1) dCDP_c0[c3] + (1) trdox_c0[c3] <- (1) CDP_c0[c3] + (1) trdrd_c0[c3]	-0.00065408	No
rxn00409	Pyrimidine metabolism	ATP:CDP phosphotransferase	(1) ATP_c0[c3] + (1) CDP_c0[c3] <-> (1) ADP_c0[c3] + (1) CTP_c0[c3]	0.026828	Yes
rxn01519	Pyrimidine metabolism	dUTP nucleotidohydrolase	(1) H2O_c0[c3] + (1) dUTP_c0[c3] -> (1) PPi_c0[c3] + (2) H+_c0[c3] + (1) dUMP_c0[c3]	0.00043395	No
rxn01513	Pyrimidine metabolism	ATP:dTMP phosphotransferase	(1) ATP_c0[c3] + (1) H+_c0[c3] + (1) dTMP_c0[c3] <-> (1) ADP_c0[c3] + (1) dTDP_c0[c3]	0.00043395	No
rxn06075	Pyrimidine metabolism	2'-Deoxyuridine 5'-diphosphate:oxidized-thioredoxin 2'-oxidoreductase	(1) H2O_c0[c3] + (1) dUDP_c0[c3] + (1) trdox_c0[c3] <- (1) UDP_c0[c3] + (1) trdrd_c0[c3]	-0.00043395	No
rxn05289	Pyrimidine metabolism	NADPH:oxidized-thioredoxin oxidoreductase	(1) NADPH_c0[c3] + (1) H+_c0[c3] + (1) trdox_c0[c3] <-> (1) NADP_c0[c3] + (1) trdrd_c0[c3]	0.00952289	No
rxn00117	Pyrimidine metabolism	ATP:UDP phosphotransferase	(1) ATP_c0[c3] + (1) UDP_c0[c3] <-> (1) ADP_c0[c3] + (1) UTP_c0[c3]	0.000415425	Yes
rxn01018	Pyrimidine metabolism|Alanine, aspartate and glutamate metabolism	carbamoyl-phosphate:L-aspartate carbamoyltransferase	(1) L-Aspartate_c0[c3] + (1) Carbamoylphosphate_c0[c3] -> (1) Phosphate_c0[c3] + (1) H+_c0[c3] + (1) N-Carbamoyl-L-aspartate_c0[c3]	0.00702895	No
rxn01520	Pyrimidine metabolism|One carbon pool by folate	5,10-Methylenetetrahydrofolate: dUMP C-methyltransferase	(1) 5-10-Methylenetetrahydrofolate_c0[c3] + (1) dUMP_c0[c3] -> (1) dTMP_c0[c3] + (1) Dihydrofolate_c0[c3]	0.00043395	No
rxn00145	Pyruvate metabolism	(S)-Lactate:ferricytochrome-c 2-oxidoreductase	(2) Cytochrome c3+_c0[c3] + (1) L-Lactate_c0[c3] <-> (1) Pyruvate_c0[c3] + (2) H+_c0[c3] + (2) Cytochrome c2+_c0[c3]	-0.286665	No
rxn00151	Pyruvate metabolism|Carbon fixation in photosynthetic organisms	ATP:pyruvate,phosphate phosphotransferase	(1) ATP_c0[c3] + (1) Phosphate_c0[c3] + (1) Pyruvate_c0[c3] <-> (1) PPi_c0[c3] + (1) AMP_c0[c3] + (1) Phosphoenolpyruvate_c0[c3] + (2) H+_c0[c3]	-0.222564	No
rxn05040	Riboflavin metabolism	D-ribulose 5-phosphate formate-lyase (L-3,4-dihydroxybutan-2-one 4-phosphate-forming)	(1) D-Ribulose5-phosphate_c0[c3] -> (1) Formate_c0[c3] + (1) H+_c0[c3] + (1) 3-4-dihydroxy-2-butanone4-phosphate_c0[c3]	0.000420632	No
rxn00300	Riboflavin metabolism	GTP 7,8-8,9-dihydrolase (diphosphate-forming)	(3) H2O_c0[c3] + (1) GTP_c0[c3] -> (1) PPi_c0[c3] + (1) Formate_c0[c3] + (3) H+_c0[c3] + (1) 2,5-Diamino-6-(5'-phosphoribosylamino)-4-pyrimidineone_c0[c3]	0.000210316	No

rxn00392	Riboflavin metabolism	ATP:riboflavin 5'-phosphotransferase	(1) ATP_c0[c3] + (1) Riboflavin_c0[c3] <-> (1) ADP_c0[c3] + (1) FMN_c0[c3] + (1) H+_c0[c3]	0.000105158	No
rxn03080	Riboflavin metabolism	5-amino-6-(D-ribitylamino)uracil butanedionetransferase	(1) 4--1-D-Ribitylamino-5-aminouracil_c0[c3] + (1) 3-4-dihydroxy-2-butanone4-phosphate_c0[c3] <-> (2) H2O_c0[c3] + (1) Phosphate_c0[c3] + (1) H+_c0[c3] + (1) 6-7-Dimethyl-8--1-D-ribityllumazine_c0[c3]	0.000420632	No
rxn00048	Riboflavin metabolism	6,7-Dimethyl-8-(1-D-ribityl)lumazine:6,7-dimethyl-8-(1-D-ribityl)lumazine 2,3-butanediyltransferase	(1) H+_c0[c3] + (2) 6-7-Dimethyl-8--1-D-ribityllumazine_c0[c3] -> (1) Riboflavin_c0[c3] + (1) 4--1-D-Ribitylamino-5-aminouracil_c0[c3]	0.000210316	No
rxn02475	Riboflavin metabolism	2,5-Diamino-6-hydroxy-4-(5-phosphoribosylamino)-pyrimidine 2-aminohydrolase	(1) H2O_c0[c3] + (1) H+_c0[c3] + (1) 2,5-Diamino-6-(5'-phosphoribosylamino)-4-pyrimidineone_c0[c3] -> (1) NH3_c0[c3] + (1) 5-Amino-6--5-phosphoribosylaminouracil_c0[c3]	0.000210316	No
rxn00122	Riboflavin metabolism	ATP:FMN adenylyltransferase	(1) ATP_c0[c3] + (1) FMN_c0[c3] -> (1) PPi_c0[c3] + (1) FAD_c0[c3]	0.000105158	No
rxn02474	Riboflavin metabolism	5-amino-6-(5-phosphoribitylamino)uracil: NADP+ 1'-oxidoreductase	(1) NADP_c0[c3] + (1) 5-Amino-6--5-phosphoribitylaminouracil_c0[c3] <-> (1) NADPH_c0[c3] + (1) H+_c0[c3] + (1) 5-Amino-6--5-phosphoribosylaminouracil_c0[c3]	-0.000210316	No
rxn01997	Streptomycin biosynthesis|Polyketide sugar unit biosynthesis|Biosynthesis of vancomycin group antibiotics	dTDPglucose 4,6-hydro-lyase	(1) dTDPglucose_c0[c3] -> (1) H2O_c0[c3] + (1) dTDP-4-oxo-6-deoxy-D-glucose_c0[c3]	0.000849375	No
rxn00623	Sulfur metabolism	hydrogen-sulfide:NADP+ oxidoreductase	(3) H2O_c0[c3] + (3) NADP_c0[c3] + (1) H2S_c0[c3] <-> (3) NADPH_c0[c3] + (3) H+_c0[c3] + (1) Sulfite_c0[c3]	-0.00734683	No
rxn00225	Taurine and hypotaurine metabolism|Pyruvate metabolism|Methane metabolism|Carbon fixation pathways in prokaryotes	ATP:acetate phosphotransferase	(1) ATP_c0[c3] + (1) Acetate_c0[c3] <-> (1) ADP_c0[c3] + (1) Acetylphosphate_c0[c3]	-0.115442	No
rxn03958	Terpenoid backbone biosynthesis	1-Deoxy-D-xylulose-5-phosphate isomeroreductase	(1) NADP_c0[c3] + (1) 2-C-methyl-D-erythritol4-phosphate_c0[c3] <-> (1) NADPH_c0[c3] + (1) H+_c0[c3] + (1) 1-deoxy-D-xylulose5-phosphate_c0[c3]	-0.0118669	No
rxn03910	Terpenoid backbone biosynthesis	2-Phospho-4-(cytidine 5'-diphospho)-2-C-methyl-D-erythritol CMP-lyase (cyclizing)	(1) 2-phospho-4--cytidine5-diphospho-2-C-methyl-D-erythritol_c0[c3] <-> (1) CMP_c0[c3] + (1) 2-C-methyl-D-erythritol2-4-cyclodiphosphate_c0[c3]	0.0118669	No
rxn03908	Terpenoid backbone biosynthesis	ATP:4-(Cytidine 5'-diphospho)-2-C-methyl-D-erythritol 2-phosphotransferase	(1) ATP_c0[c3] + (1) 4--cytidine5-diphospho-2-C-methyl-D-erythritol_c0[c3] <-> (1) ADP_c0[c3] + (1) H+_c0[c3] + (1) 2-phospho-4--cytidine5-diphospho-2-C-methyl-D-erythritol_c0[c3]	0.0118669	No
rxn08352	Terpenoid backbone biosynthesis	R08210	(1) NADH_c0[c3] + (1) H+_c0[c3] + (1) 1-Hydroxy-2-methyl-2-butenyl 4-diphosphate_c0[c3] -> (1) H2O_c0[c3] + (1) NAD_c0[c3] + (1) DMAPP_c0[c3]	0.00116485	No

rxn08756	Terpenoid backbone biosynthesis	isopentenyl-diphosphate:NAD+ oxidoreductase	(1) NADH_c0[c3] + (1) H+_c0[c3] + (1) 1-Hydroxy-2-methyl-2-butenyl 4-diphosphate_c0[c3] -> (1) H2O_c0[c3] + (1) NAD_c0[c3] + (1) Isopentenyldiphosphate_c0[c3]	0.0107021	No
rxn03907	Terpenoid backbone biosynthesis	CTP: 2-C-Methyl-D-erythritol 4-phosphate cytidylyltransferase	(1) CTP_c0[c3] + (1) 2-C-methyl-D-erythritol4-phosphate_c0[c3] <-> (1) PPi_c0[c3] + (1) 4--cytidine5-diphospho-2-C-methyl-D-erythritol_c0[c3]	0.0118669	No
rxn01213	Terpenoid backbone biosynthesis	GPPSYN-RXN	(1) Isopentenyldiphosphate_c0[c3] + (1) DMAPP_c0[c3] -> (1) PPi_c0[c3] + (1) H+_c0[c3] + (1) Geranyldiphosphate_c0[c3]	0.00116485	No
rxn03909	Terpenoid backbone biosynthesis	1-Deoxy-D-xylulose-5-phosphate pyruvate-lyase (carboxylating)	(1) Pyruvate_c0[c3] + (1) H+_c0[c3] + (1) Glyceraldehyde3-phosphate_c0[c3] -> (1) CO2_c0[c3] + (1) 1-deoxy-D-xylulose5-phosphate_c0[c3]	0.0118669	No
rxn01466	Terpenoid backbone biosynthesis	Geranyl-diphosphate:isopentenyl-diphosphate geranyltrans-transferase	(1) Isopentenyldiphosphate_c0[c3] + (1) Geranyldiphosphate_c0[c3] -> (1) PPi_c0[c3] + (1) H+_c0[c3] + (1) Farnesyldiphosphate_c0[c3]	0.00116485	No
rxn00533	Tetracycline biosynthesis|Pyruvate metabolism|Propanoate metabolism|Carbon fixation pathways in prokaryotes	Acetyl-CoA:carbon-dioxide ligase (ADP-forming)	(1) ATP_c0[c3] + (1) Acetyl-CoA_c0[c3] + (1) H2CO3_c0[c3] <-> (1) ADP_c0[c3] + (1) Phosphate_c0[c3] + (1) H+_c0[c3] + (1) Malonyl-CoA_c0[c3]	0.0875864	No
rxn00440	Thiamine metabolism	ATP:thiamine diphosphotransferase	(1) ATP_c0[c3] + (1) Thiamin_c0[c3] <-> (1) AMP_c0[c3] + (1) TPP_c0[c3] + (1) H+_c0[c3]	0.000105158	No
rxn11946	Ubiquinone and other terpenoid-quinone biosynthesis	R05614	(1) S-Adenosyl-L-methionine_c0[c3] + (1) 2-Octaprenyl-3-methyl-5-hydroxy-6-methoxy-1,4-benzoquinone_c0[c3] <-> (1) S-Adenosyl-homocysteine_c0[c3] + (1) H+_c0[c3] + (1) Ubiquinone-8_c0[c3]	0.000105158	No
rxn03397	Ubiquinone and other terpenoid-quinone biosynthesis	UDP-L-rhamnose:flavonol-3-O-D-glucoside L-rhamnosyltransferase	(1) S-Adenosyl-L-methionine_c0[c3] + (1) 2-Octaprenyl-6-methoxy-1,4-benzoquinone_c0[c3] -> (1) S-Adenosyl-homocysteine_c0[c3] + (1) H+_c0[c3] + (1) 2-Octaprenyl-3-methyl-6-methoxy-1,4-benzoquinone_c0[c3]	0.000105158	No
rxn03893	Ubiquinone and other terpenoid-quinone biosynthesis	all-trans-octaprenyl-diphosphate:4-hydroxybenzoate 3-octaprenyltransferase	(1) 4-Hydroxybenzoate_c0[c3] + (1) Farnesylfarnesylgeraniol_c0[c3] -> (1) PPi_c0[c3] + (1) H+_c0[c3] + (1) 3-Octaprenyl-4-hydroxybenzoate_c0[c3]	0.000105158	No
rxn03393	Ubiquinone and other terpenoid-quinone biosynthesis	3-octaprenyl-4-hydroxybenzoate carboxy-lyase	(1) H+_c0[c3] + (1) 3-Octaprenyl-4-hydroxybenzoate_c0[c3] -> (1) CO2_c0[c3] + (1) 2-Octaprenylphenol_c0[c3]	0.000105158	No
rxn03394	Ubiquinone and other terpenoid-quinone biosynthesis	R04987	(1) NADPH_c0[c3] + (1) O2_c0[c3] + (1) H+_c0[c3] + (1) 2-Octaprenylphenol_c0[c3] -> (1) H2O_c0[c3] + (1) NADP_c0[c3] + (1) 2-Octaprenyl-6-hydroxyphenol_c0[c3]	0.000105158	No
rxn03395	Ubiquinone and other terpenoid-quinone biosynthesis	S-adenosyl-L-methionine:3-(all-trans-octaprenyl)benzene-1,2-diol 2-O-methyltransferase	(1) S-Adenosyl-L-methionine_c0[c3] + (1) 2-Octaprenyl-6-hydroxyphenol_c0[c3] <-> (1) S-Adenosyl-homocysteine_c0[c3] + (1) H+_c0[c3] + (1) 2-Octaprenyl-6-methoxyphenol_c0[c3]	0.000105158	No

rxn03436	Valine, leucine and isoleucine biosynthesis	(S)-2-Aceto-2-hydroxybutanoate:NADP+ oxidoreductase (isomerizing)	(1) 2-Aceto-2-hydroxybutanoate_c0[c3] <-> (1) (R)-3-Hydroxy-3-methyl-2-oxopentanoate_c0[c3]	0.0111045	Yes
rxn03435	Valine, leucine and isoleucine biosynthesis	(R)-2,3-Dihydroxy-3-methylpentanoate:NADP+ oxidoreductase (isomerizing)	(1) NADP_c0[c3] + (1) 2,3-Dihydroxy-3-methylvalerate_c0[c3] <-> (1) NADPH_c0[c3] + (1) H+_c0[c3] + (1) (R)-3-Hydroxy-3-methyl-2-oxopentanoate_c0[c3]	-0.0111045	Yes
rxn03437	Valine, leucine and isoleucine biosynthesis	(R)-2,3-Dihydroxy-3-methylpentanoate hydro-lyase	(1) 2,3-Dihydroxy-3-methylvalerate_c0[c3] -> (1) H2O_c0[c3] + (1) 3MOP_c0[c3]	0.0111045	No
rxn03194	Valine, leucine and isoleucine biosynthesis	(S)-2-Aceto-2-hydroxybutanoate pyruvate-lyase (carboxylating)	(1) 2-Oxobutyrate_c0[c3] + (1) 2-Hydroxyethyl-ThPP_c0[c3] <-> (1) TPP_c0[c3] + (1) 2-Aceto-2-hydroxybutanoate_c0[c3]	0.0111045	No
rxn07434	Valine, leucine and isoleucine degradation	R07603	(1) TPP_c0[c3] + (1) H+_c0[c3] + (1) 3MOP_c0[c3] -> (1) CO2_c0[c3] + (1) 2-Methyl-1-hydroxybutyl-TPP_c0[c3]	0.00289291	No
rxn07433	Valine, leucine and isoleucine degradation	R07602	(1) Lipoamide_c0[c3] + (1) 3-Methyl-1-hydroxybutyl-TPP_c0[c3] <-> (1) TPP_c0[c3] + (1) S-(3-Methylbutanoyl)-dihydrolipoamide-E_c0[c3]	0.00289291	No
rxn07435	Valine, leucine and isoleucine degradation	R07604	(1) Lipoamide_c0[c3] + (1) 2-Methyl-1-hydroxybutyl-TPP_c0[c3] <-> (1) TPP_c0[c3] + (1) S-(2-Methylbutanoyl)-dihydrolipoamide-E_c0[c3]	0.00289291	No
rxn06586	Valine, leucine and isoleucine degradation	3-methylbutanoyl-CoA:enzyme N6-(dihydrolipoyl)lysine S-(3-methylbutanoyl)transferase	(1) Dihydrolipoamide_c0[c3] + (1) Isovaleryl-CoA_c0[c3] <-> (1) CoA_c0[c3] + (1) S-(3-Methylbutanoyl)-dihydrolipoamide-E_c0[c3]	-0.00289291	No
rxn07432	Valine, leucine and isoleucine degradation	R07601	(1) TPP_c0[c3] + (1) H+_c0[c3] + (1) 4MOP_c0[c3] -> (1) CO2_c0[c3] + (1) 3-Methyl-1-hydroxybutyl-TPP_c0[c3]	0.00289291	No
rxn06335	Valine, leucine and isoleucine degradation	(S)-2-methylbutanoyl-CoA:enzyme N6-(dihydrolipoyl)lysine S-(2-methylbutanoyl)transferase	(1) Dihydrolipoamide_c0[c3] + (1) 2-Methylbutyryl-CoA_c0[c3] <-> (1) CoA_c0[c3] + (1) S-(2-Methylbutanoyl)-dihydrolipoamide-E_c0[c3]	-0.00289291	No
rxn01575	Valine, leucine and isoleucine degradation|Valine, leucine and isoleucine biosynthesis|Glucosinolate biosynthesis	L-Isoleucine:2-oxoglutarate aminotransferase	(1) 2-Oxoglutarate_c0[c3] + (1) L-Isoleucine_c0[c3] <-> (1) L-Glutamate_c0[c3] + (1) 3MOP_c0[c3]	-0.00821163	No
rxn00806	Valine, leucine and isoleucine degradation|Valine, leucine and isoleucine biosynthesis|Glucosinolate biosynthesis	L-Leucine:2-oxoglutarate aminotransferase	(1) 2-Oxoglutarate_c0[c3] + (1) L-Leucine_c0[c3] <-> (1) L-Glutamate_c0[c3] + (1) 4MOP_c0[c3]	0.00289291	No
rxn00903	Valine, leucine and isoleucine degradation|Valine, leucine and isoleucine biosynthesis|Pantothenate and CoA biosynthesis|Glucosinolate biosynthesis	L-Valine:2-oxoglutarate aminotransferase	(1) 2-Oxoglutarate_c0[c3] + (1) L-Valine_c0[c3] <-> (1) L-Glutamate_c0[c3] + (1) 3-Methyl-2-oxobutanoate_c0[c3]	-0.0119621	No

***Pantoea* sp. YR343, community in R2A medium**

rxn00262	Alanine, aspartate and glutamate metabolism	L-Aspartic acid:oxygen oxidoreductase (deaminating)	(1) H2O_c0[c3] + (1) O2_c0[c3] + (1) L-Aspartate_c0[c3] -> (1) NH3_c0[c3] + (1) H2O2_c0[c3] + (1) Oxaloacetate_c0[c3]	0.00179542	Yes
rxn00555	Alanine, aspartate and glutamate metabolism|Amino sugar and nucleotide sugar metabolism	L-glutamine:D-fructose-6-phosphate isomerase (deaminating)	(1) L-Glutamine_c0[c3] + (1) D-fructose-6-phosphate_c0[c3] <-> (1) L-Glutamate_c0[c3] + (1) D-Glucosamine phosphate_c0[c3]	0.0580075	Yes
rxn00503	Alanine, aspartate and glutamate metabolism|Arginine and proline metabolism	(S)-1-pyrroline-5-carboxylate:NAD+ oxidoreductase	(2) H2O_c0[c3] + (1) NAD_c0[c3] + (1) 1-Pyrroline-5-carboxylate_c0[c3] <-> (1) NADH_c0[c3] + (1) L-Glutamate_c0[c3] + (1) H+_c0[c3]	0.526434	Yes
rxn00182	Alanine, aspartate and glutamate metabolism|Arginine and proline metabolism|Taurine and hypotaurine metabolism|D-Glutamine and D-glutamate metabolism|Nitrogen metabolism	L-glutamate:NAD+ oxidoreductase (deaminating)	(1) H2O_c0[c3] + (1) NAD_c0[c3] + (1) L-Glutamate_c0[c3] <-> (1) NADH_c0[c3] + (1) NH3_c0[c3] + (1) 2-Oxoglutarate_c0[c3] + (1) H+_c0[c3]	-0.298033	Yes
rxn00260	Alanine, aspartate and glutamate metabolism|Carbon fixation in photosynthetic organisms	L-Aspartate:2-oxoglutarate aminotransferase	(1) 2-Oxoglutarate_c0[c3] + (1) L-Aspartate_c0[c3] <-> (1) L-Glutamate_c0[c3] + (1) Oxaloacetate_c0[c3]	-0.162703	Yes
rxn00416	Alanine, aspartate and glutamate metabolism|Nitrogen metabolism	L-aspartate:L-glutamine amido-ligase (AMP-forming)	(1) H2O_c0[c3] + (1) ATP_c0[c3] + (1) L-Aspartate_c0[c3] + (1) L-Glutamine_c0[c3] -> (1) PPi_c0[c3] + (1) AMP_c0[c3] + (1) L-Glutamate_c0[c3] + (2) H+_c0[c3] + (1) L-Asparagine_c0[c3]	0.116448	Yes
rxn00085	Alanine, aspartate and glutamate metabolism|Nitrogen metabolism	L-Glutamate:NADP+ oxidoreductase (transaminating)	(1) NADP_c0[c3] + (2) L-Glutamate_c0[c3] <-> (1) NADPH_c0[c3] + (1) 2-Oxoglutarate_c0[c3] + (1) L-Glutamine_c0[c3] + (1) H+_c0[c3]	0.406678	Yes
rxn03638	Amino sugar and nucleotide sugar metabolism	Acetyl-CoA:D-glucosamine-1-phosphate N-acetyltransferase	(1) Acetyl-CoA_c0[c3] + (1) D-Glucosamine1-phosphate_c0[c3] -> (1) CoA_c0[c3] + (1) H+_c0[c3] + (1) N-Acetyl-D-glucosamine1-phosphate_c0[c3]	0.0580075	Yes
rxn01485	Amino sugar and nucleotide sugar metabolism	D-Glucosamine 1-phosphate 1,6-phosphomutase	(1) D-Glucosamine1-phosphate_c0[c3] <-> (1) D-Glucosamine phosphate_c0[c3]	-0.0580075	Yes
rxn00293	Amino sugar and nucleotide sugar metabolism	UTP:N-acetyl-alpha-D-glucosamine-1-phosphate uridylyltransferase	(1) UTP_c0[c3] + (1) N-Acetyl-D-glucosamine1-phosphate_c0[c3] <-> (1) PPi_c0[c3] + (1) UDP-N-acetylglucosamine_c0[c3]	0.0580075	Yes
rxn02285	Amino sugar and nucleotide sugar metabolism|Peptidoglycan biosynthesis	UDP-N-acetylmuramate:NADP+ oxidoreductase	(1) NADP_c0[c3] + (1) UDP-MurNAc_c0[c3] <-> (1) NADPH_c0[c3] + (1) H+_c0[c3] + (1) UDP-N-acetylglucosamine enolpyruvate_c0[c3]	-0.0145019	Yes
rxn00461	Amino sugar and nucleotide sugar metabolism|Peptidoglycan biosynthesis	Phosphoenolpyruvate:UDP-N-acetyl-D-glucosamine 1-carboxyvinyl-transferase	(1) UDP-N-acetylglucosamine_c0[c3] + (1) Phosphoenolpyruvate_c0[c3] <-> (1) Phosphate_c0[c3] + (1) UDP-N-acetylglucosamine enolpyruvate_c0[c3]	0.0145019	Yes
rxn00931	Arginine and proline metabolism	L-Proline:NADP+ 5-oxidoreductase	(1) NADP_c0[c3] + (1) L-Proline_c0[c3] <-> (1) NADPH_c0[c3] + (2) H+_c0[c3] + (1) 1-Pyrroline-5-carboxylate_c0[c3]	0.234799	Yes

rxn00470	Arginine and proline metabolism|Glutathione metabolism	L-ornithine carboxy-lyase (putrescine-forming)	(1) Ornithine_c0[c3] + (1) H+_c0[c3] -> (1) CO2_c0[c3] + (1) Putrescine_c0[c3]	0.00387013	Yes
rxn00171	Benzoate degradation|Pyruvate metabolism|Dioxin degradation|Xylene degradation	acetaldehyde:NAD+ oxidoreductase (CoA-acetylating)	(1) NAD_c0[c3] + (1) CoA_c0[c3] + (1) Acetaldehyde_c0[c3] <-> (1) NADH_c0[c3] + (1) Acetyl-CoA_c0[c3] + (1) H+_c0[c3]	0.535187	Yes
rxn00785	Carbon fixation in photosynthetic organisms	D-Fructose 6-phosphate:D-glyceraldehyde-3-phosphate glycolaldehyde transferase	(1) D-fructose-6-phosphate_c0[c3] + (1) Glyceraldehyde3-phosphate_c0[c3] <-> (1) D-Xylulose5-phosphate_c0[c3] + (1) D-Erythrose4-phosphate_c0[c3]	0.115729	Yes
rxn00452	Cysteine and methionine metabolism	S-Adenosyl-L-methionine:L-homocysteine S-methyltransferase	(1) S-Adenosyl-L-methionine_c0[c3] + (1) Homocysteine_c0[c3] <-> (1) S-Adenosyl-homocysteine_c0[c3] + (1) L-Methionine_c0[c3] + (1) H+_c0[c3]	-0.0107725	No
rxn00126	Cysteine and methionine metabolism	ATP:L-methionine S-adenosyltransferase	(1) H2O_c0[c3] + (1) ATP_c0[c3] + (1) L-Methionine_c0[c3] -> (1) Phosphate_c0[c3] + (1) PPi_c0[c3] + (1) S-Adenosyl-L-methionine_c0[c3] + (1) H+_c0[c3]	0.00179542	Yes
rxn00740	Cysteine and methionine metabolism	O-Succinyl-L-homoserine succinate-lyase (deaminating; 2-oxobutanoate-forming)	(1) H2O_c0[c3] + (1) O-Succinyl-L-homoserine_c0[c3] -> (1) NH3_c0[c3] + (1) Succinate_c0[c3] + (1) H+_c0[c3] + (1) 2-Oxobutyrate_c0[c3]	0.0493924	Yes
rxn00693	Cysteine and methionine metabolism|One carbon pool by folate	5-Methyltetrahydrofolate:L-homocysteine S-methyltransferase	(1) Homocysteine_c0[c3] + (1) 5-Methyltetrahydrofolate_c0[c3] <-> (1) L-Methionine_c0[c3] + (1) Tetrahydrofolate_c0[c3]	0.0107725	Yes
rxn00423	Cysteine and methionine metabolism|Sulfur metabolism	acetyl-CoA:L-serine O-acetyltransferase	(1) Acetyl-CoA_c0[c3] + (1) L-Serine_c0[c3] -> (1) CoA_c0[c3] + (1) O-Acetyl-L-serine_c0[c3]	0.0495382	Yes
rxn00649	Cysteine and methionine metabolism|Sulfur metabolism	O3-acetyl-L-serine:hydrogen-sulfide 2-amino-2-carboxyethyltransferase	(1) H2S_c0[c3] + (1) O-Acetyl-L-serine_c0[c3] -> (1) Acetate_c0[c3] + (1) L-Cysteine_c0[c3]	0.0495382	Yes
rxn01304	Cysteine and methionine metabolism|Sulfur metabolism	Succinyl-CoA:L-homoserine O-succinyltransferase	(1) Succinyl-CoA_c0[c3] + (1) L-Homoserine_c0[c3] -> (1) CoA_c0[c3] + (1) O-Succinyl-L-homoserine_c0[c3]	0.0493924	No
rxn00283	D-Alanine metabolism	alanine racemase	(1) L-Alanine_c0[c3] <-> (1) D-Alanine_c0[c3]	-0.262631	Yes
rxn00193	D-Glutamine and D-glutamate metabolism	glutamate racemase	(1) L-Glutamate_c0[c3] <-> (1) D-Glutamate_c0[c3]	0.0145019	Yes
rxn02286	D-Glutamine and D-glutamate metabolism|Peptidoglycan biosynthesis	UDP-N-acetylmuramate:L-alanine ligase (ADP-forming)	(1) ATP_c0[c3] + (1) L-Alanine_c0[c3] + (1) UDP-MurNAc_c0[c3] -> (1) ADP_c0[c3] + (1) Phosphate_c0[c3] + (1) H+_c0[c3] + (1) UDP-N-acetylmuramoyl-L-alanine_c0[c3]	0.0145019	Yes
rxn02008	D-Glutamine and D-glutamate metabolism|Peptidoglycan biosynthesis	UDP-N-acetylmuramoyl-L-alanine:D-glutamate ligase(ADP-forming)	(1) ATP_c0[c3] + (1) D-Glutamate_c0[c3] + (1) UDP-N-acetylmuramoyl-L-alanine_c0[c3] -> (1) ADP_c0[c3] + (1) Phosphate_c0[c3] + (1) H+_c0[c3] + (1) UDP-N-acetylmuramoyl-L-alanyl-D-glutamate_c0[c3]	0.0145019	Yes
rxn06672	Fatty acid biosynthesis	biotin-carboxyl-carrier-protein:carbon-dioxide ligase (ADP-forming)	(1) ATP_c0[c3] + (1) H2CO3_c0[c3] + (1) Holo-[carboxylase]_c0[c3] <-> (1) ADP_c0[c3] + (1) Phosphate_c0[c3] + (1) H+_c0[c3] + (1) Carboxybiotin-carboxyl-carrier protein_c0[c3]	0.578207	Yes
rxn05345	Fatty acid biosynthesis	dodecanoyl-[acyl-carrier-protein]:malonyl-[acyl-	(1) Dodecanoyl-ACP_c0[c3] + (1) Malonyl-acyl-carrierprotein-_c0[c3] ->	0.0580075	Yes

		carrier-protein] C-acyltransferase (decarboxylating)	(1) CO2_c0[c3] + (1) 3-oxotetradecanoyl-acp_c0[c3] + (1) ACP_c0[c3]		
rxn06673	Fatty acid biosynthesis	Acetyl-CoA:carbon-dioxide ligase (ADP-forming)	(1) Acetyl-CoA_c0[c3] + (1) Carboxybiotin-carboxyl-carrier protein_c0[c3] -> (1) Malonyl-CoA_c0[c3] + (1) Holo-[carboxylase]_c0[c3]	0.578207	Yes
rxn05465	Fatty acid biosynthesis	Malonyl-CoA:[acyl-carrier-protein] S-malonyltransferase	(1) H+_c0[c3] + (1) Malonyl-CoA_c0[c3] + (1) ACP_c0[c3] <-> (1) CoA_c0[c3] + (1) Malonyl-acyl-carrierprotein-_c0[c3]	0.578207	Yes
rxn05342	Fatty acid biosynthesis	(3R)-3-Hydroxytetradecanoyl-[acyl-carrier-protein]:NADP+ oxidoreductase	(1) NADP_c0[c3] + (1) HMA_c0[c3] <-> (1) NADPH_c0[c3] + (1) 3-oxotetradecanoyl-acp_c0[c3]	-0.0580075	Yes
rxn02504	Folate biosynthesis	2-amino-4-hydroxy-6-(D-erythro-1,2,3-trihydroxypropyl)-7,8-dihydropteridine glycolaldehyde-lyase	(1) Dihydroneopterin_c0[c3] <-> (1) Glycolaldehyde_c0[c3] + (1) 6-hydroxymethyl dihydropterin_c0[c3]	0.00538627	Yes
rxn03167	Folate biosynthesis	2-Amino-4-hydroxy-6-(erythro-1,2,3-trihydroxypropyl) dihydropteridine triphosphate phosphohydrolase (alkaline optimum)	(3) H2O_c0[c3] + (1) 7,8-Dihydroneopterin 3'-triphosphate_c0[c3] -> (3) Phosphate_c0[c3] + (3) H+_c0[c3] + (1) Dihydroneopterin_c0[c3]	0.00538627	Yes
rxn02200	Folate biosynthesis	2-amino-4-hydroxy-6-hydroxymethyl-7,8-dihydropteridine:4-aminobenzoate 2-amino-4-hydroxydihydropteridine-6-methenyltransferase	(1) ABEE_c0[c3] + (1) 6-hydroxymethyl dihydropterin_c0[c3] <-> (1) H2O_c0[c3] + (1) Dihydropteroate_c0[c3]	0.00538627	Yes
rxn03841	Folate biosynthesis	4-amino-4-deoxychorismate pyruvate-lyase	(1) ADC_c0[c3] -> (1) Pyruvate_c0[c3] + (1) H+_c0[c3] + (1) ABEE_c0[c3]	0.00538627	No
rxn01257	Folate biosynthesis	chorismate:L-glutamine aminotransferase	(1) L-Glutamine_c0[c3] + (1) Chorismate_c0[c3] <-> (1) L-Glutamate_c0[c3] + (1) ADC_c0[c3]	0.00538627	No
rxn01603	Folate biosynthesis	7,8-dihydropteroate:L-glutamate ligase (ADP-forming)	(1) ATP_c0[c3] + (1) L-Glutamate_c0[c3] + (1) Dihydropteroate_c0[c3] -> (1) ADP_c0[c3] + (1) Phosphate_c0[c3] + (1) H+_c0[c3] + (1) Dihydrofolate_c0[c3]	0.00538627	Yes
rxn01492	Fructose and mannose metabolism	ATP:D-fructose-1-phosphate 6-phosphotransferase	(1) ATP_c0[c3] + (1) D-fructose-1-phosphate_c0[c3] <-> (1) ADP_c0[c3] + (1) H+_c0[c3] + (1) D-fructose-1,6-bisphosphate_c0[c3]	0.265088	Yes
rxn00650	Glutathione metabolism	L-cysteinylglycine dipeptidase	(1) H2O_c0[c3] + (1) Cys-Gly_c0[c3] <-> (1) Glycine_c0[c3] + (1) L-Cysteine_c0[c3]	-0.00179542	Yes
rxn00350	Glutathione metabolism	glutathione gamma-glutamylaminopeptidase	(1) H2O_c0[c3] + (1) GSH_c0[c3] <-> (1) L-Glutamate_c0[c3] + (1) Cys-Gly_c0[c3]	-0.00179542	Yes
rxn00615	Glycerolipid metabolism	ATP:glycerol 3-phosphotransferase	(1) ATP_c0[c3] + (1) Glycerol_c0[c3] <-> (1) ADP_c0[c3] + (1) H+_c0[c3] + (1) Glycerol-3-phosphate_c0[c3]	0.0199628	Yes

rxn00611	Glycerophospholipid metabolism	sn-Glycerol-3-phosphate:NAD+ 2-oxidoreductase	(1) NAD_c0[c3] + (1) Glycerol-3-phosphate_c0[c3] <-> (1) NADH_c0[c3] + (1) H+_c0[c3] + (1) Glycerone-phosphate_c0[c3]	-0.0154038	No
rxn01300	Glycine, serine and threonine metabolism	ATP:L-homoserine O-phosphotransferase	(1) ATP_c0[c3] + (1) L-Homoserine_c0[c3] <-> (1) ADP_c0[c3] + (1) H+_c0[c3] + (1) O-Phospho-L-homoserine_c0[c3]	0.183495	Yes
rxn01069	Glycine, serine and threonine metabolism	O-phospho-L-homoserine phosphate-lyase (adding water;L-threonine-forming)	(1) H2O_c0[c3] + (1) O-Phospho-L-homoserine_c0[c3] -> (1) Phosphate_c0[c3] + (1) L-Threonine_c0[c3]	0.183495	Yes
rxn01068	Glycine, serine and threonine metabolism	L-threonine:NAD+ oxidoreductase	(1) NAD_c0[c3] + (1) L-Threonine_c0[c3] <-> (1) NADH_c0[c3] + (1) H+_c0[c3] + (1) L-2-Amino-acetoacetate_c0[c3]	0.0611089	Yes
rxn00274	Glycine, serine and threonine metabolism	Acetyl-CoA:glycine C-acetyltransferase	(1) Acetyl-CoA_c0[c3] + (1) Glycine_c0[c3] <-> (1) CoA_c0[c3] + (1) L-2-Amino-acetoacetate_c0[c3]	-0.0611089	Yes
rxn00337	Glycine, serine and threonine metabolism|Cysteine and methionine metabolism|Lysine biosynthesis	ATP:L-aspartate 4-phosphotransferase	(1) ATP_c0[c3] + (1) L-Aspartate_c0[c3] <-> (1) ADP_c0[c3] + (1) 4-Phospho-L-aspartate_c0[c3]	0.234683	Yes
rxn01301	Glycine, serine and threonine metabolism|Cysteine and methionine metabolism|Lysine biosynthesis	L-Homoserine:NAD+ oxidoreductase	(1) NAD_c0[c3] + (1) L-Homoserine_c0[c3] <-> (1) NADH_c0[c3] + (1) H+_c0[c3] + (1) L-Aspartate4-semialdehyde_c0[c3]	-0.234683	Yes
rxn01643	Glycine, serine and threonine metabolism|Cysteine and methionine metabolism|Lysine biosynthesis	L-Aspartate-4-semialdehyde:NADP+ oxidoreductase (phosphorylating)	(1) NADP_c0[c3] + (1) Phosphate_c0[c3] + (1) L-Aspartate4-semialdehyde_c0[c3] <- (1) NADPH_c0[c3] + (1) H+_c0[c3] + (1) 4-Phospho-L-aspartate_c0[c3]	-0.234683	Yes
rxn00781	Glycolysis / Gluconeogenesis|Carbon fixation in photosynthetic organisms	D-glyceraldehyde-3-phosphate:NAD+ oxidoreductase (phosphorylating)	(1) NAD_c0[c3] + (1) Phosphate_c0[c3] + (1) Glyceraldehyde3-phosphate_c0[c3] <-> (1) NADH_c0[c3] + (1) H+_c0[c3] + (1) 1,3-Bisphospho-D-glycerate_c0[c3]	0.78424	Yes
rxn01100	Glycolysis / Gluconeogenesis|Carbon fixation in photosynthetic organisms	ATP:3-phospho-D-glycerate 1-phosphotransferase	(1) ATP_c0[c3] + (1) 3-Phosphoglycerate_c0[c3] <-> (1) ADP_c0[c3] + (1) 1,3-Bisphospho-D-glycerate_c0[c3]	-0.78424	Yes
rxn00747	Glycolysis / Gluconeogenesis|Fructose and mannose metabolism|Inositol phosphate metabolism|Carbon fixation in photosynthetic organisms	D-glyceraldehyde-3-phosphate aldose-ketose-isomerase	(1) Glyceraldehyde3-phosphate_c0[c3] <-> (1) Glycerone-phosphate_c0[c3]	-0.246093	Yes
rxn01106	Glycolysis / Gluconeogenesis|Glycine, serine and threonine metabolism|Methane metabolism	2-Phospho-D-glycerate 2,3-phosphomutase	(1) 2-Phospho-D-glycerate_c0[c3] <-> (1) 3-Phosphoglycerate_c0[c3]	-0.78424	Yes
rxn00459	Glycolysis / Gluconeogenesis|Methane metabolism	2-phospho-D-glycerate hydro-lyase (phosphoenolpyruvate-forming)	(1) 2-Phospho-D-glycerate_c0[c3] <-> (1) H2O_c0[c3] + (1) Phosphoenolpyruvate_c0[c3]	0.78424	Yes
rxn00175	Glycolysis / Gluconeogenesis|Methane metabolism|Carbon fixation pathways in prokaryotes	Acetate:CoA ligase (AMP-forming)	(1) ATP_c0[c3] + (1) CoA_c0[c3] + (1) Acetate_c0[c3] <-> (1) PPi_c0[c3] + (1) AMP_c0[c3] + (1) Acetyl-CoA_c0[c3] + (1) H+_c0[c3]	-0.503699	Yes

rxn00371	Glyoxylate and dicarboxylate metabolism|Methane metabolism	Formate:NAD+ oxidoreductase	(1) NAD_c0[c3] + (1) Formate_c0[c3] -> (1) NADH_c0[c3] + (1) CO2_c0[c3]	0.0511878	Yes
rxn03181	Lipopolysaccharide biosynthesis	ATP:2,3,2',3'-tetrakis(3-hydroxytetradecanoyl)-D-glucosaminyl-beta-D-1,6-glucosaminyl-alpha-phosphate 4-O'-phosphotransferase	(1) ATP_c0[c3] + (1) Lipid A disaccharide_c0[c3] <-> (1) ADP_c0[c3] + (1) H+_c0[c3] + (1) Lipid IV(A)_c0[c3]	0.0145019	No
rxn02331	Lipopolysaccharide biosynthesis	phosphoenolpyruvate:D-arabinose-5-phosphate C-(1-carboxyvinyl)transferase (phosphate-hydrolysing, 2-carboxy-2-oxoethyl-forming)	(1) Phosphate_c0[c3] + (1) 3-Deoxy-D-manno-octulosonate8-phosphate_c0[c3] <- (1) H2O_c0[c3] + (1) Phosphoenolpyruvate_c0[c3] + (1) D-Arabinose5-phosphate_c0[c3]	-0.0435056	Yes
rxn02404	Lipopolysaccharide biosynthesis	3-Deoxy-D-manno-octulosonate-8-phosphate 8-phosphohydrolase	(1) H2O_c0[c3] + (1) 3-Deoxy-D-manno-octulosonate8-phosphate_c0[c3] -> (1) Phosphate_c0[c3] + (1) KDO_c0[c3]	0.0435056	Yes
rxn03919	Lipopolysaccharide biosynthesis	D-glycero-beta-D-manno-heptose 1,7-bisphosphate 7-phosphohydrolase	(1) H2O_c0[c3] + (1) D-Glycero-D-manno-heptose1-7-bisphosphate_c0[c3] -> (1) Phosphate_c0[c3] + (1) D-Glycero-D-manno-heptose1-phosphate_c0[c3]	0.0580075	No
rxn03159	Lipopolysaccharide biosynthesis	UDP-2,3-bis(3-hydroxytetradecanoyl)glucosamine:2,3-bis-(3-hydroxytetradecanoyl)-alpha-D-glucosaminyl-1-phosphate 2,3-bis(3-hydroxytetradecanoyl)-glucosaminyltransferase	(1) UDP-2,3-bis(3-hydroxytetradecanoyl)glucosamine_c0[c3] + (1) Lipid X_c0[c3] <-> (1) UDP_c0[c3] + (1) Lipid A disaccharide_c0[c3]	0.0145019	Yes
rxn06723	Lipopolysaccharide biosynthesis	(3R)-3-hydroxymyristoyl-[acyl-carrier protein]:UDP-3-O-[(3R)-3-hydroxymyristoyl]-alpha-D-glucosamine N-acetyltransferase	(1) UDP-2,3-bis(3-hydroxytetradecanoyl)glucosamine_c0[c3] + (1) ACP_c0[c3] <- (1) UDP-3-O-(beta-hydroxymyristoyl)-D-glucosamine_c0[c3] + (1) HMA_c0[c3]	-0.0290038	Yes
rxn03146	Lipopolysaccharide biosynthesis	UDP-3-O-[(3R)-3-hydroxymyristoyl]-N-acetylglucosamine amidohydrolase	(1) H2O_c0[c3] + (1) UDP-3-O-(beta-hydroxymyristoyl)-N-acetylglucosamine_c0[c3] <-> (1) Acetate_c0[c3] + (1) UDP-3-O-(beta-hydroxymyristoyl)-D-glucosamine_c0[c3]	0.0290038	Yes
rxn02405	Lipopolysaccharide biosynthesis	CTP:3-deoxy-D-manno-octulosonate cytidylyltransferase	(1) CTP_c0[c3] + (1) KDO_c0[c3] -> (1) PPi_c0[c3] + (1) H+_c0[c3] + (1) CMP-KDO_c0[c3]	0.0435056	Yes
rxn06865	Lipopolysaccharide biosynthesis	R05146	(1) Lauroyl-KDO2-lipid IV(A)_c0[c3] + (1) ACP_c0[c3] <- (1) kdo2-lipid iva_c0[c3] + (1) Dodecanoyl-ACP_c0[c3]	-0.0145019	Yes
rxn03439	Lipopolysaccharide biosynthesis	CMP-3-deoxy-D-manno-oct-2-ulosonate:(KDO)-lipid IVA 3-deoxy-D-manno-oct-2-ulosonate transferase	(1) CMP-KDO_c0[c3] + (1) KDO-lipid IV(A)_c0[c3] <-> (1) CMP_c0[c3] + (1) H+_c0[c3] + (1) kdo2-lipid iva_c0[c3]	0.0145019	Yes
rxn06729	Lipopolysaccharide biosynthesis	(R)-3-Hydroxytetradecanoly-[acyl-carrier-protein]:UDP-N-acetyl-glucosamine 3-O-(3-hydroxytetradecanoyl)transferase	(1) UDP-N-acetylglucosamine_c0[c3] + (1) H+_c0[c3] + (1) HMA_c0[c3] -> (1) UDP-3-O-(beta-hydroxymyristoyl)-N-acetylglucosamine_c0[c3] + (1) ACP_c0[c3]	0.0290038	Yes

rxn03130	Lipopolysaccharide biosynthesis	UDP-2,3-bis[(3R)-3-hydroxymyristoyl]-alpha-D-glucosamine 2,3-bis[(3R)-3-hydroxymyristoyl]-beta-D-glucosaminyl 1-phosphate phosphohydrolase	(1) H2O_c0[c3] + (1) UDP-2,3-bis(3-hydroxytetradecanoyl)glucosamine_c0[c3] -> (2) H+_c0[c3] + (1) UMP_c0[c3] + (1) Lipid X_c0[c3]	0.0145019	Yes
rxn03511	Lipopolysaccharide biosynthesis	ADP-D-glycero-D-manno-heptose 6-epimerase	(1) ADP-D-glycero-D-manno-heptose_c0[c3] <-> (1) ADP-L-glycero-D-manno-heptose_c0[c3]	0.0580075	Yes
rxn03182	Lipopolysaccharide biosynthesis	CMP-3-deoxy-D-manno-oct-2-ulosonate:lipid IVA 3-deoxy-D-manno-oct-2-ulosonate transferase	(1) CMP-KDO_c0[c3] + (1) Lipid IV(A)_c0[c3] <-> (1) CMP_c0[c3] + (1) H+_c0[c3] + (1) KDO-lipid IV(A)_c0[c3]	0.0145019	Yes
rxn03918	Lipopolysaccharide biosynthesis	ATP:D-glycero-beta-D-manno-heptose 7-phosphate 1-phosphotransferase	(1) ATP_c0[c3] + (1) D-Glycero-D-manno-heptose7-phosphate[c3] -> (1) ADP_c0[c3] + (1) H+_c0[c3] + (1) D-Glycero-D-manno-heptose1-7-bisphosphate_c0[c3]	0.0580075	No
rxn06848	Lipopolysaccharide biosynthesis	R05075	(1) kdo2-lipid a_c0[c3] + (1) ACP_c0[c3] <- (1) Lauroyl-KDO2-lipid IV(A)_c0[c3] + (1) Myristoyl-ACP_c0[c3]	-0.0145019	No
rxn03916	Lipopolysaccharide biosynthesis	ATP:D-glycero-beta-D-manno-heptose 1-phosphate adenylyltransferase	(1) ATP_c0[c3] + (1) D-Glycero-D-manno-heptose1-phosphate_c0[c3] -> (1) PPi_c0[c3] + (1) ADP-D-glycero-D-manno-heptose_c0[c3]	0.0580075	No
rxn03917	Lipopolysaccharide biosynthesis	D-glycero-beta-D-manno-heptose-7-phosphate aldose-ketose-isomerase	(1) Sedoheptulose7-phosphate_c0[c3] -> (1) D-Glycero-D-manno-heptose7-phosphate[c3]	0.0580075	No
rxn00313	Lysine biosynthesis	meso-2,6-diaminoheptanedioate carboxy-lyase (L-lysine-forming)	(1) H+_c0[c3] + (1) meso-2,6-Diaminopimelate_c0[c3] <-> (1) CO2_c0[c3] + (1) L-Lysine_c0[c3]	-0.0145019	Yes
rxn02011	Lysine biosynthesis|Peptidoglycan biosynthesis	UDP-N-acetylmuramoyl-L-alanyl-D-glutamate:(L)-meso-2,6-diaminoheptanedioate gamma-ligase (ADP-forming)	(1) ATP_c0[c3] + (1) meso-2,6-Diaminopimelate_c0[c3] + (1) UDP-N-acetylmuramoyl-L-alanyl-D-glutamate_c0[c3] -> (1) ADP_c0[c3] + (1) Phosphate_c0[c3] + (1) H+_c0[c3] + (1) UDP-N-acetylmuramoyl-L-alanyl-D-gamma-glutamyl-meso-2-6-diaminopimelate_c0[c3]	0.0145019	Yes
rxn03164	Lysine biosynthesis|Peptidoglycan biosynthesis	UDP-N-acetylmuramoyl-L-alanyl-D-glutamyl-meso-2,6-diaminoheptanedioate:D-alanyl-D-alanine ligase(ADP-forming)	(1) ATP_c0[c3] + (1) Ala-Ala[c3] + (1) UDP-N-acetylmuramoyl-L-alanyl-D-gamma-glutamyl-meso-2-6-diaminopimelate_c0[c3] -> (1) ADP_c0[c3] + (1) Phosphate_c0[c3] + (1) H+_c0[c3] + (1) UDP-N-acetylmuramoyl-L-alanyl-D-glutamyl-6-carboxy-L-lysyl-D-alanyl- D-alanine_c0[c3]	0.0145019	No
rxn00786	Methane metabolism|Carbon fixation in photosynthetic organisms	D-fructose-1,6-bisphosphate D-glyceraldehyde-3-phosphate-lyase (glycerone-phosphate-forming)	(1) D-fructose-1,6-bisphosphate_c0[c3] <-> (1) Glycerone-phosphate_c0[c3] + (1) Glyceraldehyde3-phosphate_c0[c3]	0.265088	Yes
rxn02988	Nicotinate and nicotinamide metabolism	glycerone phosphate:iminosuccinate alkyltransferase (cyclizing)	(2) H2O_c0[c3] + (1) Phosphate_c0[c3] + (1) Quinolinate_c0[c3] <- (1) Glycerone-phosphate_c0[c3] + (1) Iminoaspartate_c0[c3]	-0.00359084	No
rxn02402	Nicotinate and nicotinamide metabolism	Nicotinate-nucleotide:pyrophosphate	(1) CO2_c0[c3] + (1) PPi_c0[c3] + (1) Nicotinate ribonucleotide_c0[c3] <- (1)	-0.00359084	Yes

		phosphoribosyltransferase (carboxylating)	H+_c0[c3] + (1) PRPP_c0[c3] + (1) Quinolinate_c0[c3]		
rxn02155	Nicotinate and nicotinamide metabolism	ATP:nicotinamide-nucleotide adenylyltransferase	(1) ATP_c0[c3] + (1) Nicotinate ribonucleotide_c0[c3] <-> (1) PPi_c0[c3] + (1) Deamido-NAD_c0[c3]	0.00359084	No
rxn00083	Nicotinate and nicotinamide metabolism	NADPH:NAD+ oxidoreductase	(1) NAD_c0[c3] + (1) NADPH_c0[c3] <-> (1) NADH_c0[c3] + (1) NADP_c0[c3]	-0.560298	Yes
rxn01265	Nicotinate and nicotinamide metabolism	Nicotinate D-ribonucleotide:diphosphate phosphoribosyltransferase	(1) PPi_c0[c3] + (1) Nicotinate ribonucleotide_c0[c3] <-> (1) PRPP_c0[c3] + (1) Niacin_c0[c3]	-0.00179542	No
rxn00077	Nicotinate and nicotinamide metabolism	ATP:NAD+ 2'-phosphotransferase	(1) ATP_c0[c3] + (1) NAD_c0[c3] <-> (1) NADP_c0[c3] + (1) ADP_c0[c3] + (1) H+_c0[c3]	0.00179542	Yes
rxn00338	Nicotinate and nicotinamide metabolism	L-aspartate:oxygen oxidoreductase	(1) O2_c0[c3] + (1) L-Aspartate_c0[c3] -> (1) H2O2_c0[c3] + (1) H+_c0[c3] + (1) Iminoaspartate_c0[c3]	0.00359084	Yes
rxn00138	Nicotinate and nicotinamide metabolism	deamido-NAD+:ammonia ligase (AMP-forming)	(1) ATP_c0[c3] + (1) NH3_c0[c3] + (1) Deamido-NAD_c0[c3] -> (1) NAD_c0[c3] + (1) PPi_c0[c3] + (1) AMP_c0[c3] + (2) H+_c0[c3]	0.00359084	Yes
rxn00102	Nitrogen metabolism	carbonate hydro-lyase (carbon-dioxide-forming)	(1) H+_c0[c3] + (1) H2CO3_c0[c3] <-> (1) H2O_c0[c3] + (1) CO2_c0[c3]	-0.578207	Yes
rxn00907	One carbon pool by folate|Carbon fixation pathways in prokaryotes	5,10-methylenetetrahydrofolate: NADP+ oxidoreductase	(1) NADP_c0[c3] + (1) 5-10-Methylenetetrahydrofolate_c0[c3] <-> (1) NADPH_c0[c3] + (1) 5-10-Methenyltetrahydrofolate_c0[c3]	-0.012568	Yes
rxn01211	One carbon pool by folate|Carbon fixation pathways in prokaryotes	5,10-Methenyltetrahydrofolate 5-hydrolase (decyclizing)	(1) H2O_c0[c3] + (1) 5-10-Methenyltetrahydrofolate_c0[c3] <-> (1) H+_c0[c3] + (1) 10-Formyltetrahydrofolate_c0[c3]	-0.012568	Yes
rxn00686	One carbon pool by folate|Folate biosynthesis	5,6,7,8-tetrahydrofolate:NADP+ oxidoreductase	(1) NADP_c0[c3] + (1) Tetrahydrofolate_c0[c3] <-> (1) NADPH_c0[c3] + (1) H+_c0[c3] + (1) Dihydrofolate_c0[c3]	-0.00538627	Yes
rxn04954	One carbon pool by folate|Methane metabolism|Carbon fixation pathways in prokaryotes	5-methyltetrahydrofolate:NAD+ oxidoreductase	(1) NAD_c0[c3] + (1) 5-Methyltetrahydrofolate_c0[c3] <-> (1) NADH_c0[c3] + (1) H+_c0[c3] + (1) 5-10-Methylenetetrahydrofolate_c0[c3]	-0.012568	No
rxn12512	Pantothenate and CoA biosynthesis	(R)-4'-Phosphopantothenate:L-cysteine ligase	(1) ATP_c0[c3] + (1) L-Cysteine_c0[c3] + (1) 4-phosphopantothenate_c0[c3] -> (1) PPi_c0[c3] + (1) AMP_c0[c3] + (2) H+_c0[c3] + (1) (R)-4'-Phosphopantothenoyl-L-cysteine_c0[c3]	0.00359084	Yes
rxn00100	Pantothenate and CoA biosynthesis	ATP:dephospho-CoA 3'-phosphotransferase	(1) ATP_c0[c3] + (1) Dephospho-CoA_c0[c3] -> (1) ADP_c0[c3] + (1) CoA_c0[c3] + (1) H+_c0[c3]	0.00359084	No
rxn02175	Pantothenate and CoA biosynthesis	ATP:pantetheine-4'-phosphate adenylyltransferase	(1) ATP_c0[c3] + (1) Phosphopantetheine_c0[c3] <-> (1) PPi_c0[c3] + (1) Dephospho-CoA_c0[c3]	0.00359084	Yes
rxn06023	Pantothenate and CoA biosynthesis	CoA:apo-[acyl-carrier-protein] pantetheinephosphotransferase	(1) CoA_c0[c3] + (1) apo-ACP_c0[c3] <-> (1) Adenosine 3-5-bisphosphate_c0[c3] + (1) ACP_c0[c3]	0.00179542	Yes
rxn12510	Pantothenate and CoA biosynthesis	ATP:pantothenate 4'-phosphotransferase	(1) ATP_c0[c3] + (1) PAN_c0[c3] <-> (1) ADP_c0[c3] + (1) H+_c0[c3] + (1) 4-phosphopantothenate_c0[c3]	0.00359084	Yes

rxn02341	Pantothenate and CoA biosynthesis	N-[(R)-4'-Phosphopantothenoyl]-L-cysteine carboxy-lyase	(1) H+_c0[c3] + (1) (R)-4'-Phosphopantothenoyl-L-cysteine_c0[c3] -> (1) CO2_c0[c3] + (1) Phosphopantetheine_c0[c3]	0.00359084	Yes
rxn00213	Pentose and glucuronate interconversions|Galactose metabolism|Starch and sucrose metabolism|Amino sugar and nucleotide sugar metabolism	UTP:alpha-D-glucose-1-phosphate uridylyltransferase	(1) UTP_c0[c3] + (1) Glucose-1-phosphate_c0[c3] <-> (1) PPi_c0[c3] + (1) UDP-glucose_c0[c3]	-0.0145019	Yes
rxn00772	Pentose phosphate pathway	ATP:D-ribose 5-phosphotransferase	(1) ATP_c0[c3] + (1) D-Ribose_c0[c3] <-> (1) ADP_c0[c3] + (1) H+_c0[c3] + (1) ribose-5-phosphate_c0[c3]	-0.0976488	Yes
rxn01333	Pentose phosphate pathway	sedoheptulose-7-phosphate:D-glyceraldehyde-3-phosphate glyceronetransferase	(1) Glyceraldehyde3-phosphate_c0[c3] + (1) Sedoheptulose7-phosphate_c0[c3] <-> (1) D-fructose-6-phosphate_c0[c3] + (1) D-Erythrose4-phosphate_c0[c3]	-0.104956	Yes
rxn00784	Pentose phosphate pathway	2-deoxy-D-ribose-5-phosphate acetaldehyde-lyase (D-glyceraldehyde-3-phosphate-forming)	(1) deoxyribose-5-phosphate_c0[c3] <-> (1) Acetaldehyde_c0[c3] + (1) Glyceraldehyde3-phosphate_c0[c3]	0.535187	Yes
rxn00777	Pentose phosphate pathway|Carbon fixation in photosynthetic organisms	D-ribose-5-phosphate aldose-ketose-isomerase	(1) ribose-5-phosphate_c0[c3] <-> (1) D-Ribulose5-phosphate_c0[c3]	-0.110195	Yes
rxn01200	Pentose phosphate pathway|Carbon fixation in photosynthetic organisms	Sedoheptulose-7-phosphate:D-glyceraldehyde-3-phosphate glycolaldehyde transferase	(1) Glyceraldehyde3-phosphate_c0[c3] + (1) Sedoheptulose7-phosphate_c0[c3] <-> (1) ribose-5-phosphate_c0[c3] + (1) D-Xylulose5-phosphate_c0[c3]	0.0469487	Yes
rxn01116	Pentose phosphate pathway|Pentose and glucuronate interconversions|Carbon fixation in photosynthetic organisms	D-Ribulose-5-phosphate 3-epimerase	(1) D-Ribulose5-phosphate_c0[c3] <-> (1) D-Xylulose5-phosphate_c0[c3]	-0.162677	Yes
rxn00770	Pentose phosphate pathway|Purine metabolism	ATP:D-ribose-5-phosphate diphosphotransferase	(1) ATP_c0[c3] + (1) ribose-5-phosphate_c0[c3] <-> (1) AMP_c0[c3] + (1) H+_c0[c3] + (1) PRPP_c0[c3]	0.00538627	Yes
rxn03408	Peptidoglycan biosynthesis	UDP-N-acetyl-D-glucosamine:undecaprenyl-diphospho-N-acetylmuramoyl-L-alanyl-gamma-D-glutamyl-meso-2,6-diaminopimeloyl-D-alanyl-D-alanine 4-beta-N-acetylglucosaminlytransferase	(1) UDP-N-acetylglucosamine_c0[c3] + (1) Undecaprenyl-diphospho-N-acetylmuramoyl-L-alanyl-D-glutamyl-meso-2-6-diaminopimeloyl-D-alanyl-D-alanine_c0[c3] <-> (1) UDP_c0[c3] + (1) Undecaprenyl-diphospho-N-acetylmuramoyl--N-acetylglucosamine-L-ala-D-glu-meso-2-6-diaminopimeloyl-D-ala-D-ala_c0[c3]	0.0145019	Yes
rxn03904	Peptidoglycan biosynthesis	UDP-N-acetylmuramoyl-L-alanyl-gamma-D-glutamyl-meso-2,6-diaminopimeloyl-D-alanyl-D-alanine:undecaprenyl-phosphate phospho-N-acetylmuramoyl-pentapeptide-transferase	(1) Undecaprenylphosphate_c0[c3] + (1) UDP-N-acetylmuramoyl-L-alanyl-D-glutamyl-6-carboxy-L-lysyl-D-alanyl- D-alanine_c0[c3] <-> (1) UMP_c0[c3] + (1) Undecaprenyl-diphospho-N-acetylmuramoyl-L-alanyl-D-glutamyl-meso-2-6-diaminopimeloyl-D-alanyl-D-alanine_c0[c3]	0.0145019	Yes
rxn03901	Peptidoglycan biosynthesis	undecaprenyl-diphosphate phosphohydrolase	(1) H2O_c0[c3] + (1) Bactoprenyl diphosphate_c0[c3] -> (1) Phosphate_c0[c3] + (2) H+_c0[c3] + (1) Undecaprenylphosphate_c0[c3]	0.0145019	Yes
rxn01739	Phenylalanine, tyrosine and tryptophan biosynthesis	ATP:shikimate 3-phosphotransferase	(1) ATP_c0[c3] + (1) Shikimate_c0[c3] <-> (1) ADP_c0[c3] + (1) H+_c0[c3] + (1) 3-phosphoshikimate_c0[c3]	0.0107725	Yes

rxn02212	Phenylalanine, tyrosine and tryptophan biosynthesis	2-Dehydro-3-deoxy-D-arabino-heptonate 7-phosphate phosphate-lyase (cyclyzing)	(1) DAHP_c0[c3] -> (1) Phosphate_c0[c3] + (1) 5-Dehydroquinate_c0[c3]	0.0107725	Yes
rxn01740	Phenylalanine, tyrosine and tryptophan biosynthesis	Shikimate:NADP+ 3-oxidoreductase	(1) NADP_c0[c3] + (1) Shikimate_c0[c3] <-> (1) NADPH_c0[c3] + (1) H+_c0[c3] + (1) 3-Dehydroshikimate_c0[c3]	-0.0107725	Yes
rxn01255	Phenylalanine, tyrosine and tryptophan biosynthesis	5-O-(1-Carboxyvinyl)-3-phosphoshikimate phosphate-lyase (chorismate-forming)	(1) 5-O--1-Carboxyvinyl-3-phosphoshikimate_c0[c3] -> (1) Phosphate_c0[c3] + (1) Chorismate_c0[c3]	0.0107725	Yes
rxn02476	Phenylalanine, tyrosine and tryptophan biosynthesis	Phosphoenolpyruvate:3-phosphoshikimate 5-O-(1-carboxyvinyl)-transferase	(1) Phosphoenolpyruvate_c0[c3] + (1) 3-phosphoshikimate_c0[c3] <-> (1) Phosphate_c0[c3] + (1) 5-O--1-Carboxyvinyl-3-phosphoshikimate_c0[c3]	0.0107725	Yes
rxn02213	Phenylalanine, tyrosine and tryptophan biosynthesis	3-Dehydroquinate hydro-lyase	(1) 5-Dehydroquinate_c0[c3] -> (1) H2O_c0[c3] + (1) 3-Dehydroshikimate_c0[c3]	0.0107725	Yes
rxn01332	Phenylalanine, tyrosine and tryptophan biosynthesis	Phosphoenolpyruvate:D-erythrose-4-phosphate C-(1-carboxyvinyl)transferase (phosphate hydrolysing, 2-carboxy-2-oxoethyl-forming)	(1) H2O_c0[c3] + (1) Phosphoenolpyruvate_c0[c3] + (1) D-Erythrose4-phosphate_c0[c3] -> (1) Phosphate_c0[c3] + (1) DAHP_c0[c3]	0.0107725	Yes
rxn00060	Porphyrin and chlorophyll metabolism	porphobilinogen:(4-[2-carboxyethyl]-3-[carboxymethyl]pyrrol-2-yl)methyltransferase (hydrolysing)	(1) H2O_c0[c3] + (4) Porphobilinogen_c0[c3] -> (4) NH3_c0[c3] + (1) Hydroxymethylbilane_c0[c3]	0.00359084	Yes
rxn02264	Porphyrin and chlorophyll metabolism	Hydroxymethylbilane hydro-lyase(cyclizing)	(1) Hydroxymethylbilane_c0[c3] <-> (1) H2O_c0[c3] + (1) UroporphyrinogenIII_c0[c3]	0.00359084	Yes
rxn00029	Porphyrin and chlorophyll metabolism	5-aminolevulinate hydro-lyase (adding 5-aminolevulinate and cyclizing; porphobilinogen-forming)	(2) 5-Aminolevulinate_c0[c3] -> (2) H2O_c0[c3] + (1) H+_c0[c3] + (1) Porphobilinogen_c0[c3]	0.0143634	Yes
rxn01629	Porphyrin and chlorophyll metabolism	(S)-4-Amino-5-oxopentanoate 4,5-aminomutase	(1) 5-Aminolevulinate_c0[c3] <-> (1) L-Glutamate1-semialdehyde_c0[c3]	-0.0287268	Yes
rxn05029	Porphyrin and chlorophyll metabolism	ATP:cobinamide Cobeta-adenosyltransferase	(1) ATP_c0[c3] + (1) H+_c0[c3] + (1) Cobinamide_c0[c3] <-> (1) Triphosphate_c0[c3] + (1) Adenosyl cobinamide_c0[c3]	0.00179542	No
rxn02303	Porphyrin and chlorophyll metabolism	Coproporphyrinogen:oxyge n oxidoreductase(decarboxyla ting)	(1) O2_c0[c3] + (2) H+_c0[c3] + (1) CoproporphyrinogenIII_c0[c3] <-> (2) H2O_c0[c3] + (2) CO2_c0[c3] + (1) ProtoporphyrinogenIX_c0[c3]	0.00179542	No
rxn00056	Porphyrin and chlorophyll metabolism	Fe(II):oxygen oxidoreductase	(1) O2_c0[c3] + (4) H+_c0[c3] + (4) Fe2+_c0[c3] <-> (2) H2O_c0[c3] + (4) fe3_c0[c3]	-0.00134657	Yes
rxn02056	Porphyrin and chlorophyll metabolism	S-Adenosyl-L-methionine:uroporphyrin-III C-methyltransferase	(2) H+_c0[c3] + (1) Siroheme_c0[c3] <-> (1) Sirohydrochlorin_c0[c3] + (1) Fe2+_c0[c3]	-0.00179542	No
rxn02288	Porphyrin and chlorophyll metabolism	Uroporphyrinogen-III carboxy-lyase	(4) H+_c0[c3] + (1) UroporphyrinogenIII_c0[c3] <-> (4) CO2_c0[c3] + (1) CoproporphyrinogenIII_c0[c3]	0.00179542	Yes
rxn06591	Porphyrin and chlorophyll metabolism	L-glutamate-semialdehyde: NADP+ oxidoreductase(L-	(1) NADPH_c0[c3] + (1) H+_c0[c3] + (1) L-Glutamyl-tRNA-Glu_c0[c3] <->	0.0287268	No

		glutamyl-tRNA(Glu)-forming)	(1) NADP_c0[c3] + (1) L-Glutamate1-semialdehyde_c0[c3] + (1) tRNA-Glu_c0[c3]		
rxn00224	Porphyrin and chlorophyll metabolism	protoheme ferro-lyase (protoporphyrin-forming)	(1) Protoporphyrin_c0[c3] + (1) Fe2+_c0[c3] <-> (1) Heme_c0[c3] + (2) H+_c0[c3]	0.00179542	Yes
rxn03537	Porphyrin and chlorophyll metabolism	R05222	(1) GTP_c0[c3] + (1) Adenosyl cobinamide phosphate[c3] -> (1) PPi_c0[c3] + (1) H+_c0[c3] + (1) Adenosylcobinamide-GDP[c3]	0.00179542	No
rxn03538	Porphyrin and chlorophyll metabolism	R05223	(1) alpha-Ribazole[c3] + (1) Adenosylcobinamide-GDP[c3] -> (1) H+_c0[c3] + (1) GMP_c0[c3] + (1) Calomide_c0[c3]	0.00179542	No
rxn03150	Porphyrin and chlorophyll metabolism	R04594	(1) H2O_c0[c3] + (1) alpha-Ribazole 5'-phosphate[c3] -> (1) Phosphate_c0[c3] + (1) alpha-Ribazole[c3]	0.00179542	No
rxn02897	Porphyrin and chlorophyll metabolism	Nicotinate-nucleotide:dimethylbenzimidazole phospho-D-ribosyltransferase	(1) Nicotinate ribonucleotide_c0[c3] + (1) Dimethylbenzimidazole_c0[c3] -> (1) H+_c0[c3] + (1) Niacin_c0[c3] + (1) alpha-Ribazole 5'-phosphate[c3]	0.00179542	No
rxn03536	Porphyrin and chlorophyll metabolism	R05221	(1) ATP_c0[c3] + (1) Adenosyl cobinamide_c0[c3] -> (1) ADP_c0[c3] + (1) H+_c0[c3] + (1) Adenosyl cobinamide phosphate[c3]	0.00179542	No
rxn06937	Porphyrin and chlorophyll metabolism|Aminoacyl-tRNA biosynthesis	L-glutamate:tRNA(Glu) ligase (AMP-forming)	(1) ATP_c0[c3] + (1) L-Glutamate_c0[c3] + (1) tRNA-Glu_c0[c3] -> (1) PPi_c0[c3] + (1) AMP_c0[c3] + (1) H+_c0[c3] + (1) L-Glutamyl-tRNA-Glu_c0[c3]	0.0287268	Yes
rxn04794	Propanoate metabolism	propanoyl-CoA:formate C-propanoyltransferase	(1) CoA_c0[c3] + (1) 2-Oxobutyrate_c0[c3] <-> (1) Formate_c0[c3] + (1) Propionyl-CoA_c0[c3]	0.0493924	Yes
rxn01297	Purine metabolism	hypoxanthine:NAD+ oxidoreductase	(1) H2O_c0[c3] + (1) NAD_c0[c3] + (1) HYXN_c0[c3] <-> (1) NADH_c0[c3] + (1) H+_c0[c3] + (1) XAN_c0[c3]	0.0133579	Yes
rxn00131	Purine metabolism	AMP phosphoribohydrolase	(1) H2O_c0[c3] + (1) AMP_c0[c3] <-> (1) ribose-5-phosphate_c0[c3] + (1) Adenine_c0[c3]	-0.083112	Yes
rxn01445	Purine metabolism	2'-Deoxyguanosine 5'-monophosphate phosphohydrolase	(1) H2O_c0[c3] + (1) dGMP_c0[c3] -> (1) Phosphate_c0[c3] + (1) Deoxyguanosine_c0[c3]	0.000396649	No
rxn00917	Purine metabolism	Xanthosine-5'-phosphate:L-glutamine amido-ligase (AMP-forming)	(1) H2O_c0[c3] + (1) ATP_c0[c3] + (1) L-Glutamine_c0[c3] + (1) XMP_c0[c3] -> (1) PPi_c0[c3] + (1) AMP_c0[c3] + (1) L-Glutamate_c0[c3] + (3) H+_c0[c3] + (1) GMP_c0[c3]	0.0980079	Yes
rxn05234	Purine metabolism	2'-Deoxyguanosine 5'-triphosphate:oxydized-thioredoxin 2'-oxidoreductase	(1) GTP_c0[c3] + (1) trdrd_c0[c3] -> (1) H2O_c0[c3] + (1) dGTP_c0[c3] + (1) trdox_c0[c3]	0.0101213	No
rxn00927	Purine metabolism	Adenosine ribohydrolase	(1) H2O_c0[c3] + (1) Adenosine_c0[c3] <-> (1) D-Ribose_c0[c3] + (1) Adenine_c0[c3]	-0.0976488	No
rxn00237	Purine metabolism	ATP:GDP phosphotransferase	(1) ATP_c0[c3] + (1) GDP_c0[c3] <-> (1) ADP_c0[c3] + (1) GTP_c0[c3]	-25.8483	Yes
rxn01509	Purine metabolism	ATP:dGMP phosphotransferase	(1) ATP_c0[c3] + (1) H+_c0[c3] + (1) dGMP_c0[c3] <-> (1) ADP_c0[c3] + (1) dGDP_c0[c3]	-0.000396649	Yes

rxn00834	Purine metabolism	IMP:NAD+ oxidoreductase	(1) H2O_c0[c3] + (1) NAD_c0[c3] + (1) IMP_c0[c3] <-> (1) NADH_c0[c3] + (1) H+_c0[c3] + (1) XMP_c0[c3]	0.0980079	Yes
rxn01859	Purine metabolism	Deoxyadenosine:orthophosphate ribosyltransferase	(1) Phosphate_c0[c3] + (1) Deoxyadenosine_c0[c3] <-> (1) Adenine_c0[c3] + (1) deoxyribose-1-phosphate_c0[c3]	0.180761	No
rxn05233	Purine metabolism	2'-Deoxyguanosine 5'-diphosphate:oxidized-thioredoxin 2'-oxidoreductase	(1) GDP_c0[c3] + (1) trdrd_c0[c3] -> (1) H2O_c0[c3] + (1) dGDP_c0[c3] + (1) trdox_c0[c3]	0.000396649	Yes
rxn00835	Purine metabolism	ATP:inosine 5'-phosphotransferase	(1) ATP_c0[c3] + (1) Inosine_c0[c3] <-> (1) ADP_c0[c3] + (1) H+_c0[c3] + (1) IMP_c0[c3]	0.0980079	Yes
rxn01985	Purine metabolism	Deoxyinosine:orthophosphate ribosyltransferase	(1) Phosphate_c0[c3] + (1) Deoxyinosine_c0[c3] <-> (1) HYXN_c0[c3] + (1) deoxyribose-1-phosphate_c0[c3]	0.0193444	No
rxn01858	Purine metabolism	Deoxyadenosine aminohydrolase	(1) H2O_c0[c3] + (1) H+_c0[c3] + (1) Deoxyadenosine_c0[c3] -> (1) NH3_c0[c3] + (1) Deoxyinosine_c0[c3]	0.0193444	Yes
rxn00239	Purine metabolism	ATP:GMP phosphotransferase	(1) ATP_c0[c3] + (1) H+_c0[c3] + (1) GMP_c0[c3] <-> (1) ADP_c0[c3] + (1) GDP_c0[c3]	0.0998033	Yes
rxn01137	Purine metabolism	Adenosine aminohydrolase	(1) H2O_c0[c3] + (1) H+_c0[c3] + (1) Adenosine_c0[c3] -> (1) NH3_c0[c3] + (1) Inosine_c0[c3]	0.0934058	Yes
rxn05232	Purine metabolism	2'-Deoxyadenosine 5'-triphosphate:oxydized-thioredoxin 2'-oxidoreductase	(1) ATP_c0[c3] + (1) trdrd_c0[c3] -> (1) H2O_c0[c3] + (1) dATP_c0[c3] + (1) trdox_c0[c3]	0.00845636	No
rxn03004	Purine metabolism|One carbon pool by folate	10-Formyltetrahydrofolate:5'-phosphoribosylglycinamide formyltransferase	(1) 10-Formyltetrahydrofolate_c0[c3] + (1) GAR_c0[c3] <-> (1) H+_c0[c3] + (1) Tetrahydrofolate_c0[c3] + (1) N-Formyl-GAR_c0[c3]	-0.0143634	Yes
rxn00379	Purine metabolism|Sulfur metabolism	ATP:sulfate adenylyltransferase	(1) ATP_c0[c3] + (1) Sulfate_c0[c3] <-> (1) PPi_c0[c3] + (1) APS_c0[c3]	26.1523	Yes
rxn00364	Pyrimidine metabolism	ATP:CMP phosphotransferase	(1) ATP_c0[c3] + (1) CMP_c0[c3] + (1) H+_c0[c3] <-> (1) ADP_c0[c3] + (1) CDP_c0[c3]	0.460763	Yes
rxn05235	Pyrimidine metabolism	2'-Deoxyuridine 5'-triphosphate:oxydized-thioredoxin 2'-oxidoreductase	(1) CTP_c0[c3] + (1) trdrd_c0[c3] -> (1) H2O_c0[c3] + (1) dCTP_c0[c3] + (1) trdox_c0[c3]	0.00407577	No
rxn01516	Pyrimidine metabolism	dTTP:uridine 5'-phosphotransferase	(1) Uridine_c0[c3] + (1) TTP_c0[c3] <-> (1) H+_c0[c3] + (1) UMP_c0[c3] + (1) dTDP_c0[c3]	-0.0229582	Yes
rxn00369	Pyrimidine metabolism	GTP:cytidine 5'-phosphotransferase	(1) GTP_c0[c3] + (1) Cytidine_c0[c3] <-> (1) GDP_c0[c3] + (1) CMP_c0[c3] + (1) H+_c0[c3]	0.15506	Yes
rxn00410	Pyrimidine metabolism	UTP:ammonia ligase (ADP-forming)	(1) ATP_c0[c3] + (1) NH3_c0[c3] + (1) UTP_c0[c3] <-> (1) ADP_c0[c3] + (1) Phosphate_c0[c3] + (1) CTP_c0[c3] + (2) H+_c0[c3]	-0.0816708	Yes
rxn00368	Pyrimidine metabolism	UTP:cytidine 5'-phosphotransferase	(1) UTP_c0[c3] + (1) Cytidine_c0[c3] <-> (1) UDP_c0[c3] + (1) CMP_c0[c3] + (1) H+_c0[c3]	-0.0145019	Yes
rxn01366	Pyrimidine metabolism	Uridine:phosphate alpha-D-ribosyltransferase	(1) Phosphate_c0[c3] + (1) Uridine_c0[c3] <-> (1) Uracil_c0[c3] + (1) Ribose 1-phosphate_c0[c3]	0.0290038	Yes

rxn01143	Pyrimidine metabolism	ATP:thymidine 5'-phosphotransferase	(1) ATP_c0[c3] + (1) Thymidine_c0[c3] <-> (1) ADP_c0[c3] + (1) H+_c0[c3] + (1) dTMP_c0[c3]	0.00845636	Yes
rxn01800	Pyrimidine metabolism	Deoxycytidine aminohydrolase	(1) H2O_c0[c3] + (1) H+_c0[c3] + (1) Deoxycytidine_c0[c3] -> (1) NH3_c0[c3] + (1) Deoxyuridine_c0[c3]	0.335081	Yes
rxn06076	Pyrimidine metabolism	2'-Deoxycytidine diphosphate:oxidized-thioredoxin 2'-oxidoreductase	(1) H2O_c0[c3] + (1) dCDP_c0[c3] + (1) trdox_c0[c3] <- (1) CDP_c0[c3] + (1) trdrd_c0[c3]	-0.00604552	Yes
rxn01674	Pyrimidine metabolism	dCTP:uridine 5'-phosphotransferase	(1) Uridine_c0[c3] + (1) dCTP_c0[c3] <-> (1) H+_c0[c3] + (1) UMP_c0[c3] + (1) dCDP_c0[c3]	-0.00604552	Yes
rxn00409	Pyrimidine metabolism	ATP:CDP phosphotransferase	(1) ATP_c0[c3] + (1) CDP_c0[c3] <-> (1) ADP_c0[c3] + (1) CTP_c0[c3]	0.454717	Yes
rxn01799	Pyrimidine metabolism	deoxyuridine:orthophosphate 2-deoxy-D-ribosyltransferase	(1) Phosphate_c0[c3] + (1) Deoxyuridine_c0[c3] <-> (1) Uracil_c0[c3] + (1) deoxyribose-1-phosphate_c0[c3]	0.335081	No
rxn01513	Pyrimidine metabolism	ATP:dTMP phosphotransferase	(1) ATP_c0[c3] + (1) H+_c0[c3] + (1) dTMP_c0[c3] <-> (1) ADP_c0[c3] + (1) dTDP_c0[c3]	0.00845636	Yes
rxn05289	Pyrimidine metabolism	NADPH:oxidized-thioredoxin oxidoreductase	(1) NADPH_c0[c3] + (1) H+_c0[c3] + (1) trdox_c0[c3] <-> (1) NADP_c0[c3] + (1) trdrd_c0[c3]	0.0786338	Yes
rxn00251	Pyruvate metabolism|Methane metabolism|Carbon fixation in photosynthetic organisms|Carbon fixation pathways in prokaryotes	phosphate:oxaloacetate carboxy-lyase (adding phosphate;phosphoenolpyruvate-forming)	(1) Phosphate_c0[c3] + (1) Oxaloacetate_c0[c3] + (1) H+_c0[c3] <-> (1) H2O_c0[c3] + (1) CO2_c0[c3] + (1) Phosphoenolpyruvate_c0[c3]	-0.160907	Yes
rxn05040	Riboflavin metabolism	D-ribulose 5-phosphate formate-lyase (L-3,4-dihydroxybutan-2-one 4-phosphate-forming)	(1) D-Ribulose5-phosphate_c0[c3] -> (1) Formate_c0[c3] + (1) H+_c0[c3] + (1) 3-4-dihydroxy-2-butanone4-phosphate_c0[c3]	0.00718169	Yes
rxn00300	Riboflavin metabolism	GTP 7,8-8,9-dihydrolase (diphosphate-forming)	(3) H2O_c0[c3] + (1) GTP_c0[c3] -> (1) PPi_c0[c3] + (1) Formate_c0[c3] + (3) H+_c0[c3] + (1) 2,5-Diamino-6-(5'-phosphoribosylamino)-4-pyrimidineone_c0[c3]	0.00359084	Yes
rxn00392	Riboflavin metabolism	ATP:riboflavin 5'-phosphotransferase	(1) ATP_c0[c3] + (1) Riboflavin_c0[c3] <-> (1) ADP_c0[c3] + (1) FMN_c0[c3] + (1) H+_c0[c3]	0.00179542	Yes
rxn03080	Riboflavin metabolism	5-amino-6-(D-ribitylamino)uracil butanedionetransferase	(1) 4--1-D-Ribitylamino-5-aminouracil_c0[c3] + (1) 3-4-dihydroxy-2-butanone4-phosphate_c0[c3] <-> (2) H2O_c0[c3] + (1) Phosphate_c0[c3] + (1) H+_c0[c3] + (1) 6-7-Dimethyl-8--1-D-ribityllumazine_c0[c3]	0.00718169	Yes
rxn00048	Riboflavin metabolism	6,7-Dimethyl-8-(1-D-ribityl)lumazine:6,7-dimethyl-8-(1-D-ribityl)lumazine 2,3-butanediyltransferase	(1) H+_c0[c3] + (2) 6-7-Dimethyl-8--1-D-ribityllumazine_c0[c3] -> (1) Riboflavin_c0[c3] + (1) 4--1-D-Ribitylamino-5-aminouracil_c0[c3]	0.00359084	Yes
rxn02475	Riboflavin metabolism	2,5-Diamino-6-hydroxy-4-(5-phosphoribosylamino)-pyrimidine 2-aminohydrolase	(1) H2O_c0[c3] + (1) H+_c0[c3] + (1) 2,5-Diamino-6-(5'-phosphoribosylamino)-4-pyrimidineone_c0[c3] -> (1) NH3_c0[c3] + (1) 5-Amino-6--5-phosphoribosylaminouracil_c0[c3]	0.00359084	Yes
rxn00122	Riboflavin metabolism	ATP:FMN adenylyltransferase	(1) ATP_c0[c3] + (1) FMN_c0[c3] -> (1) PPi_c0[c3] + (1) FAD_c0[c3]	0.00179542	Yes

rxn02474	Riboflavin metabolism	5-amino-6-(5-phosphoribitylamino)uracil: NADP+ 1'-oxidoreductase	(1) NADP_c0[c3] + (1) 5-Amino-6--5-phosphoribitylaminouracil_c0[c3] <-> (1) NADPH_c0[c3] + (1) H+_c0[c3] + (1) 5-Amino-6--5-phosphoribosylaminouracil_c0[c3]	-0.00359084	Yes
rxn05039	Riboflavin metabolism	R07280	(1) H2O_c0[c3] + (1) 5-Amino-6--5-phosphoribitylaminouracil_c0[c3] -> (1) Phosphate_c0[c3] + (1) 4--1-D-Ribitylamino-5-aminouracil_c0[c3]	0.00359084	No
rxn00695	Starch and sucrose metabolism|Amino sugar and nucleotide sugar metabolism	ATP:alpha-D-glucose-1-phosphate adenylyltransferase	(1) ATP_c0[c3] + (1) Glucose-1-phosphate_c0[c3] <-> (1) PPi_c0[c3] + (1) ADPglucose_c0[c3]	-0.840155	Yes
rxn01675	Streptomycin biosynthesis|Polyketide sugar unit biosynthesis	dTTP:alpha-D-glucose-1-phosphate thymidylyltransferase	(1) Glucose-1-phosphate_c0[c3] + (1) TTP_c0[c3] <-> (1) PPi_c0[c3] + (1) dTDPglucose_c0[c3]	0.0145019	Yes
rxn02000	Streptomycin biosynthesis|Polyketide sugar unit biosynthesis	dTDP-4-dehydro-6-deoxy-D-glucose 3,5-epimerase	(1) dTDP-4-oxo-6-deoxy-D-glucose_c0[c3] -> (1) dTDP-4-oxo-L-rhamnose[c3]	0.0145019	No
rxn02003	Streptomycin biosynthesis|Polyketide sugar unit biosynthesis	dTDP-6-deoxy-L-mannose:NADP+ 4-oxidoreductase	(1) NADP_c0[c3] + (1) dTDP-rhamnose[c3] <- (1) NADPH_c0[c3] + (1) H+_c0[c3] + (1) dTDP-4-oxo-L-rhamnose[c3]	-0.0145019	No
rxn01997	Streptomycin biosynthesis|Polyketide sugar unit biosynthesis|Biosynthesis of vancomycin group antibiotics	dTDPglucose 4,6-hydro-lyase	(1) dTDPglucose_c0[c3] -> (1) H2O_c0[c3] + (1) dTDP-4-oxo-6-deoxy-D-glucose_c0[c3]	0.0145019	Yes
rxn00623	Sulfur metabolism	hydrogen-sulfide:NADP+ oxidoreductase	(3) H2O_c0[c3] + (3) NADP_c0[c3] + (1) H2S_c0[c3] <-> (3) NADPH_c0[c3] + (3) H+_c0[c3] + (1) Sulfite_c0[c3]	-0.0495382	Yes
rxn00225	Taurine and hypotaurine metabolism|Pyruvate metabolism|Methane metabolism|Carbon fixation pathways in prokaryotes	ATP:acetate phosphotransferase	(1) ATP_c0[c3] + (1) Acetate_c0[c3] <-> (1) ADP_c0[c3] + (1) Acetylphosphate_c0[c3]	0.582241	Yes
rxn00173	Taurine and hypotaurine metabolism|Pyruvate metabolism|Methane metabolism|Carbon fixation pathways in prokaryotes	acetyl-CoA:phosphate acetyltransferase	(1) Phosphate_c0[c3] + (1) Acetyl-CoA_c0[c3] <-> (1) CoA_c0[c3] + (1) Acetylphosphate_c0[c3]	-0.582241	Yes
rxn01486	Terpenoid backbone biosynthesis	trans,trans-Farnesyl-diphosphate:isopentenyl-diphosphate farnesyltranstransferase	(1) Isopentenyldiphosphate_c0[c3] + (1) Farnesyldiphosphate_c0[c3] -> (1) PPi_c0[c3] + (1) H+_c0[c3] + (1) Geranylgeranyl diphosphate_c0[c3]	0.00538627	No
rxn03958	Terpenoid backbone biosynthesis	1-Deoxy-D-xylulose-5-phosphate isomeroreductase	(1) NADP_c0[c3] + (1) 2-C-methyl-D-erythritol4-phosphate_c0[c3] <-> (1) NADPH_c0[c3] + (1) H+_c0[c3] + (1) 1-deoxy-D-xylulose5-phosphate_c0[c3]	-0.202611	No
rxn03910	Terpenoid backbone biosynthesis	2-Phospho-4-(cytidine 5'-diphospho)-2-C-methyl-D-erythritol CMP-lyase (cyclizing)	(1) 2-phospho-4--cytidine5-diphospho-2-C-methyl-D-erythritol_c0[c3] <-> (1) CMP_c0[c3] + (1) 2-C-methyl-D-erythritol2-4-cyclodiphosphate_c0[c3]	0.202611	Yes
rxn03908	Terpenoid backbone biosynthesis	ATP:4-(Cytidine 5'-diphospho)-2-C-methyl-D-erythritol 2-phosphotransferase	(1) ATP_c0[c3] + (1) 4--cytidine5-diphospho-2-C-methyl-D-erythritol_c0[c3] <-> (1) ADP_c0[c3] + (1) H+_c0[c3] + (1) 2-phospho-4--cytidine5-diphospho-2-C-methyl-D-erythritol_c0[c3]	0.202611	No
rxn08352	Terpenoid backbone biosynthesis	R08210	(1) NADH_c0[c3] + (1) H+_c0[c3] + (1) 1-Hydroxy-2-methyl-2-butenyl 4-diphosphate_c0[c3] -> (1) H2O_c0[c3]	0.0198881	Yes

			+ (1) NAD_c0[c3] + (1) DMAPP_c0[c3]		
rxn08756	Terpenoid backbone biosynthesis	isopentenyl-diphosphate:NAD+ oxidoreductase	(1) NADH_c0[c3] + (1) H+_c0[c3] + (1) 1-Hydroxy-2-methyl-2-butenyl 4-diphosphate_c0[c3] -> (1) H2O_c0[c3] + (1) NAD_c0[c3] + (1) Isopentenyldiphosphate_c0[c3]	0.182723	Yes
rxn03907	Terpenoid backbone biosynthesis	CTP: 2-C-Methyl-D-erythritol 4-phosphate cytidylyltransferase	(1) CTP_c0[c3] + (1) 2-C-methyl-D-erythritol4-phosphate_c0[c3] <-> (1) PPi_c0[c3] + (1) 4--cytidine5-diphospho-2-C-methyl-D-erythritol_c0[c3]	0.202611	No
rxn01213	Terpenoid backbone biosynthesis	GPPSYN-RXN	(1) Isopentenyldiphosphate_c0[c3] + (1) DMAPP_c0[c3] -> (1) PPi_c0[c3] + (1) H+_c0[c3] + (1) Geranyldiphosphate_c0[c3]	0.0198881	Yes
rxn03909	Terpenoid backbone biosynthesis	1-Deoxy-D-xylulose-5-phosphate pyruvate-lyase (carboxylating)	(1) Pyruvate_c0[c3] + (1) H+_c0[c3] + (1) Glyceraldehyde3-phosphate_c0[c3] -> (1) CO2_c0[c3] + (1) 1-deoxy-D-xylulose5-phosphate_c0[c3]	0.202611	Yes
rxn01466	Terpenoid backbone biosynthesis	Geranyl-diphosphate:isopentenyl-diphosphate geranyltrans-transferase	(1) Isopentenyldiphosphate_c0[c3] + (1) Geranyldiphosphate_c0[c3] -> (1) PPi_c0[c3] + (1) H+_c0[c3] + (1) Farnesyldiphosphate_c0[c3]	0.0198881	Yes
rxn00438	Thiamine metabolism	ATP:thiamin-phosphate phosphotransferase	(1) ATP_c0[c3] + (1) H+_c0[c3] + (1) Thiamine phosphate_c0[c3] <-> (1) ADP_c0[c3] + (1) TPP_c0[c3]	0.00179542	Yes
rxn11946	Ubiquinone and other terpenoid-quinone biosynthesis	R05614	(1) S-Adenosyl-L-methionine_c0[c3] + (1) 2-Octaprenyl-3-methyl-5-hydroxy-6-methoxy-1,4-benzoquinone_c0[c3] <-> (1) S-Adenosyl-homocysteine_c0[c3] + (1) H+_c0[c3] + (1) Ubiquinone-8_c0[c3]	0.00179542	Yes
rxn00966	Ubiquinone and other terpenoid-quinone biosynthesis	chorismate pyruvate-lyase (4-hydroxybenzoate-forming)	(1) Pyruvate_c0[c3] + (1) 4-Hydroxybenzoate_c0[c3] <- (1) Chorismate_c0[c3]	-0.00179542	No
rxn04139	Ubiquinone and other terpenoid-quinone biosynthesis	2-Octaprenyl-3-methyl-6-methoxy-1,4-benzoquinone ,NADPH2:oxygen oxidoreductase	(1) NADPH_c0[c3] + (1) O2_c0[c3] + (1) H+_c0[c3] + (1) 2-Octaprenyl-3-methyl-6-methoxy-1,4-benzoquinone_c0[c3] -> (1) H2O_c0[c3] + (1) NADP_c0[c3] + (1) 2-Octaprenyl-3-methyl-5-hydroxy-6-methoxy-1,4-benzoquinone_c0[c3]	0.00179542	Yes
rxn03893	Ubiquinone and other terpenoid-quinone biosynthesis	all-trans-octaprenyl-diphosphate:4-hydroxybenzoate 3-octaprenyltransferase	(1) 4-Hydroxybenzoate_c0[c3] + (1) Farnesylfarnesylgeraniol_c0[c3] -> (1) PPi_c0[c3] + (1) H+_c0[c3] + (1) 3-Octaprenyl-4-hydroxybenzoate_c0[c3]	0.00179542	Yes
rxn03393	Ubiquinone and other terpenoid-quinone biosynthesis	3-octaprenyl-4-hydroxybenzoate carboxy-lyase	(1) H+_c0[c3] + (1) 3-Octaprenyl-4-hydroxybenzoate_c0[c3] -> (1) CO2_c0[c3] + (1) 2-Octaprenylphenol_c0[c3]	0.00179542	No
rxn03394	Ubiquinone and other terpenoid-quinone biosynthesis	R04987	(1) NADPH_c0[c3] + (1) O2_c0[c3] + (1) H+_c0[c3] + (1) 2-Octaprenylphenol_c0[c3] -> (1) H2O_c0[c3] + (1) NADP_c0[c3] + (1) 2-Octaprenyl-6-hydroxyphenol_c0[c3]	0.00179542	Yes
rxn03395	Ubiquinone and other terpenoid-quinone biosynthesis	S-adenosyl-L-methionine:3-(all-trans-octaprenyl)benzene-1,2-diol 2-O-methyltransferase	(1) S-Adenosyl-L-methionine_c0[c3] + (1) 2-Octaprenyl-6-hydroxyphenol_c0[c3] <-> (1) S-Adenosyl-homocysteine_c0[c3] + (1) H+_c0[c3] + (1) 2-Octaprenyl-6-methoxyphenol_c0[c3]	0.00179542	Yes

rxn02831	Ubiquinone and other terpenoid-quinone biosynthesis	O-Succinylbenzoate:CoA ligase (AMP-forming)	(1) ATP_c0[c3] + (1) CoA_c0[c3] + (1) Succinylbenzoate[c3] -> (1) PPi_c0[c3] + (1) AMP_c0[c3] + (1) H+_c0[c3] + (1) Succinylbenzoyl-CoA[c3]	0.00359084	No
rxn03397	Ubiquinone and other terpenoid-quinone biosynthesis	UDP-L-rhamnose:flavonol-3-O-D-glucoside L-rhamnosyltransferase	(1) S-Adenosyl-L-methionine_c0[c3] + (1) 2-Octaprenyl-6-methoxy-1,4-benzoquinone_c0[c3] -> (1) S-Adenosyl-homocysteine_c0[c3] + (1) H+_c0[c3] + (1) 2-Octaprenyl-3-methyl-6-methoxy-1,4-benzoquinone_c0[c3]	0.00179542	No
rxn02832	Ubiquinone and other terpenoid-quinone biosynthesis	(1R,6R)-6-hydroxy-2-succinylcyclohexa-2,4-diene-1-carboxylate hydrolyase (2-succinylbenzoate-forming)	(1) H2O_c0[c3] + (1) Succinylbenzoate[c3] <- (1) SHCHC[c3]	-0.00359084	No
rxn01258	Ubiquinone and other terpenoid-quinone biosynthesis|Biosynthesis of siderophore group nonribosomal peptides	chorismate hydroxymutase	(1) Chorismate_c0[c3] <-> (1) Isochorismate_c0[c3]	0.00359084	Yes
rxn02933	Valine, leucine and isoleucine degradation	(2S,3S)-3-hydroxy-2-methylbutanoyl-CoA:NAD+ oxidoreductase	(1) NAD_c0[c3] + (1) 2-methyl-3-hydroxy-butyryl-CoA_c0[c3] <-> (1) NADH_c0[c3] + (1) H+_c0[c3] + (1) 2-Methylacetoacetyl-CoA_c0[c3]	-0.0493924	Yes
rxn02934	Valine, leucine and isoleucine degradation	(2S,3S)-3-Hydroxy-2-methylbutanoyl-CoA hydro-liase	(1) 2-methyl-3-hydroxy-butyryl-CoA_c0[c3] <-> (1) H2O_c0[c3] + (1) Tiglyl-CoA_c0[c3]	0.0493924	Yes
rxn00676	Valine, leucine and isoleucine degradation	acetyl-CoA:propanoyl-CoA 2-C-acetyltransferase	(1) Acetyl-CoA_c0[c3] + (1) Propionyl-CoA_c0[c3] <-> (1) CoA_c0[c3] + (1) 2-Methylacetoacetyl-CoA_c0[c3]	0.0493924	Yes
rxn00806	Valine, leucine and isoleucine degradation|Valine, leucine and isoleucine biosynthesis|Glucosinolate biosynthesis	L-Leucine:2-oxoglutarate aminotransferase	(1) 2-Oxoglutarate_c0[c3] + (1) L-Leucine_c0[c3] <-> (1) L-Glutamate_c0[c3] + (1) 4MOP_c0[c3]	0.0493924	Yes
***Pseudomonas* sp. GM17, community in R2A medium**					
rxn00555	Alanine, aspartate and glutamate metabolism|Amino sugar and nucleotide sugar metabolism	L-glutamine:D-fructose-6-phosphate isomerase (deaminating)	(1) L-Glutamine_c0[c2] + (1) D-fructose-6-phosphate_c0[c2] <-> (1) L-Glutamate_c0[c2] + (1) D-Glucosamine phosphate_c0[c2]	0.0598724	Yes
rxn00503	Alanine, aspartate and glutamate metabolism|Arginine and proline metabolism	(S)-1-pyrroline-5-carboxylate:NAD+ oxidoreductase	(2) H2O_c0[c2] + (1) NAD_c0[c2] + (1) 1-Pyrroline-5-carboxylate_c0[c2] <-> (1) NADH_c0[c2] + (1) L-Glutamate_c0[c2] + (1) H+_c0[c2]	-0.183346	Yes
rxn00189	Alanine, aspartate and glutamate metabolism|Arginine and proline metabolism|D-Glutamine and D-glutamate metabolism|Nitrogen metabolism	L-Glutamine amidohydrolase	(1) H2O_c0[c2] + (1) L-Glutamine_c0[c2] -> (1) NH3_c0[c2] + (1) L-Glutamate_c0[c2]	0.383635	Yes
rxn00182	Alanine, aspartate and glutamate metabolism|Arginine and proline metabolism|Taurine and hypotaurine metabolism|D-Glutamine and D-glutamate metabolism|Nitrogen metabolism	L-glutamate:NAD+ oxidoreductase (deaminating)	(1) H2O_c0[c2] + (1) NAD_c0[c2] + (1) L-Glutamate_c0[c2] <-> (1) NADH_c0[c2] + (1) NH3_c0[c2] + (1) 2-Oxoglutarate_c0[c2] + (1) H+_c0[c2]	-0.741439	Yes

rxn00260	Alanine, aspartate and glutamate metabolism|Carbon fixation in photosynthetic organisms	L-Aspartate:2-oxoglutarate aminotransferase	(1) 2-Oxoglutarate_c0[c2] + (1) L-Aspartate_c0[c2] <-> (1) L-Glutamate_c0[c2] + (1) Oxaloacetate_c0[c2]	-0.391694	Yes
rxn00416	Alanine, aspartate and glutamate metabolism|Nitrogen metabolism	L-aspartate:L-glutamine amido-ligase (AMP-forming)	(1) H2O_c0[c2] + (1) ATP_c0[c2] + (1) L-Aspartate_c0[c2] + (1) L-Glutamine_c0[c2] -> (1) PPi_c0[c2] + (1) AMP_c0[c2] + (1) L-Glutamate_c0[c2] + (2) H+_c0[c2] + (1) L-Asparagine_c0[c2]	0.0129386	Yes
rxn01484	Amino sugar and nucleotide sugar metabolism	N-Acetyl-D-glucosamine-6-phosphate amidohydrolase	(1) H2O_c0[c2] + (1) N-Acetyl-D-glucosamine 6-phosphate_c0[c2] <-> (1) Acetate_c0[c2] + (1) D-Glucosamine phosphate_c0[c2]	-0.0553607	Yes
rxn03638	Amino sugar and nucleotide sugar metabolism	Acetyl-CoA:D-glucosamine-1-phosphate N-acetyltransferase	(1) Acetyl-CoA_c0[c2] + (1) D-Glucosamine1-phosphate_c0[c2] -> (1) CoA_c0[c2] + (1) H+_c0[c2] + (1) N-Acetyl-D-glucosamine1-phosphate_c0[c2]	0.0045117	Yes
rxn01485	Amino sugar and nucleotide sugar metabolism	D-Glucosamine 1-phosphate 1,6-phosphomutase	(1) D-Glucosamine1-phosphate_c0[c2] <-> (1) D-Glucosamine phosphate_c0[c2]	-0.0045117	Yes
rxn00293	Amino sugar and nucleotide sugar metabolism	UTP:N-acetyl-alpha-D-glucosamine-1-phosphate uridylyltransferase	(1) UTP_c0[c2] + (1) N-Acetyl-D-glucosamine1-phosphate_c0[c2] <-> (1) PPi_c0[c2] + (1) UDP-N-acetylglucosamine_c0[c2]	0.0045117	Yes
rxn02285	Amino sugar and nucleotide sugar metabolism|Peptidoglycan biosynthesis	UDP-N-acetylmuramate:NADP+ oxidoreductase	(1) NADP_c0[c2] + (1) UDP-MurNAc_c0[c2] <-> (1) NADPH_c0[c2] + (1) H+_c0[c2] + (1) UDP-N-acetylglucosamine enolpyruvate_c0[c2]	-0.00112792	Yes
rxn00461	Amino sugar and nucleotide sugar metabolism|Peptidoglycan biosynthesis	Phosphoenolpyruvate:UDP-N-acetyl-D-glucosamine 1-carboxyvinyl-transferase	(1) UDP-N-acetylglucosamine_c0[c2] + (1) Phosphoenolpyruvate_c0[c2] <-> (1) Phosphate_c0[c2] + (1) UDP-N-acetylglucosamine enolpyruvate_c0[c2]	0.00112792	Yes
rxn01636	Arginine and proline metabolism	N2-Acetyl-L-ornithine:L-glutamate N-acetyltransferase	(1) L-Glutamate_c0[c2] + (1) N-Acetylornithine_c0[c2] <-> (1) Ornithine_c0[c2] + (1) N-Acetyl-L-glutamate_c0[c2]	0.00387013	Yes
rxn01637	Arginine and proline metabolism	N2-Acetyl-L-ornithine:2-oxoglutarate aminotransferase	(1) 2-Oxoglutarate_c0[c2] + (1) N-Acetylornithine_c0[c2] <-> (1) L-Glutamate_c0[c2] + (1) 2-Acetamido-5-oxopentanoate_c0[c2]	-0.00387013	No
rxn01917	Arginine and proline metabolism	ATP:N-acetyl-L-glutamate 5-phosphotransferase	(1) ATP_c0[c2] + (1) N-Acetyl-L-glutamate_c0[c2] <-> (1) ADP_c0[c2] + (1) n-acetylglutamyl-phosphate_c0[c2]	0.00387013	Yes
rxn00929	Arginine and proline metabolism	L-Proline:NAD+ 5-oxidoreductase	(1) NAD_c0[c2] + (1) L-Proline_c0[c2] <-> (1) NADH_c0[c2] + (2) H+_c0[c2] + (1) 1-Pyrroline-5-carboxylate_c0[c2]	-0.183346	Yes
rxn02465	Arginine and proline metabolism	N-acetyl-L-glutamate-5-semialdehyde:NADP+ 5-oxidoreductase (phosphrylating)	(1) NADP_c0[c2] + (1) Phosphate_c0[c2] + (1) 2-Acetamido-5-oxopentanoate_c0[c2] <- (1) NADPH_c0[c2] + (1) H+_c0[c2] + (1) n-acetylglutamyl-phosphate_c0[c2]	-0.00387013	Yes
rxn00501	beta-Alanine metabolism|Inositol phosphate metabolism|Propanoate metabolism	3-Oxopropanoate:NAD+ oxidoreductase (decarboxylating, CoA-acetylating)	(1) NAD_c0[c2] + (1) CoA_c0[c2] + (1) 3-Oxopropanoate_c0[c2] <-> (1) NADH_c0[c2] + (1) CO2_c0[c2] + (1) Acetyl-CoA_c0[c2]	0.354472	Yes
rxn01791	beta-Alanine metabolism|Pantothenate and CoA biosynthesis	(R)-Pantoate:beta-alanine ligase (AMP-forming)	(1) ATP_c0[c2] + (1) beta-Alanine_c0[c2] + (1) Pantoate_c0[c2] -> (1) PPi_c0[c2] + (1) AMP_c0[c2] + (2) H+_c0[c2] + (1) PAN_c0[c2]	0.000279288	Yes

rxn00656	beta-Alanine metabolism|Propanoate metabolism	L-Alanine:3-oxopropanoate aminotransferase	(1) L-Alanine_c0[c2] + (1) 3-Oxopropanoate_c0[c2] <-> (1) Pyruvate_c0[c2] + (1) beta-Alanine_c0[c2]	-0.354472	Yes
rxn00785	Carbon fixation in photosynthetic organisms	D-Fructose 6-phosphate:D-glyceraldehyde-3-phosphate glycolaldehyde transferase	(1) D-fructose-6-phosphate_c0[c2] + (1) Glyceraldehyde3-phosphate_c0[c2] <-> (1) D-Xylulose5-phosphate_c0[c2] + (1) D-Erythrose4-phosphate_c0[c2]	0.436943	Yes
rxn00285	Citrate cycle (TCA cycle)|Propanoate metabolism|Carbon fixation pathways in prokaryotes	Succinate:CoA ligase (ADP-forming)	(1) ATP_c0[c2] + (1) CoA_c0[c2] + (1) Succinate_c0[c2] <-> (1) ADP_c0[c2] + (1) Phosphate_c0[c2] + (1) Succinyl-CoA_c0[c2]	0.0417852	Yes
rxn05104	Cysteine and methionine metabolism	S-methyl-5-thio-D-ribulose-1-phosphate hydro-lyase	(1) methylthioribulose-1-phosphate_c0[c2] -> (1) H2O_c0[c2] + (1) 2,3-diketo5-methylthio-1-phosphopentane_c0[c2]	0.000139644	No
rxn03057	Cysteine and methionine metabolism	5-Methylthio-5-deoxy-D-ribose-1-phosphate ketol-isomerase	(1) methylthioribose-1-phosphate_c0[c2] <-> (1) methylthioribulose-1-phosphate_c0[c2]	0.000139644	Yes
rxn01022	Cysteine and methionine metabolism	S-methyl-5'-thioadenosine:phosphate S-methyl-5-thio-alpha-D-ribosyl-transferase	(1) Phosphate_c0[c2] + (1) 5-Methylthioadenosine_c0[c2] <-> (1) Adenine_c0[c2] + (1) methylthioribose-1-phosphate_c0[c2]	0.000139644	Yes
rxn00126	Cysteine and methionine metabolism	ATP:L-methionine S-adenosyltransferase	(1) H2O_c0[c2] + (1) ATP_c0[c2] + (1) L-Methionine_c0[c2] -> (1) Phosphate_c0[c2] + (1) PPi_c0[c2] + (1) S-Adenosyl-L-methionine_c0[c2] + (1) H+_c0[c2]	0.00111715	Yes
rxn05108	Cysteine and methionine metabolism	R07396	(1) L-Glutamate_c0[c2] + (1) 4-methylthio 2-oxobutyrate_c0[c2] <-> (1) 2-Oxoglutarate_c0[c2] + (1) L-Methionine_c0[c2]	0.000139644	Yes
rxn05092	Cysteine and methionine metabolism	1,2-dihydroxy-5-(methylthio)pent-1-en-3-one:oxygen oxidoreductase (formate-forming)	(1) O2_c0[c2] + (1) 1,2-dihydroxy-3-keto-5-methylthiopentene_c0[c2] -> (1) Formate_c0[c2] + (2) H+_c0[c2] + (1) 4-methylthio 2-oxobutyrate_c0[c2]	0.000139644	Yes
rxn00141	Cysteine and methionine metabolism	S-Adenosyl-L-homocysteine hydrolase	(1) H2O_c0[c2] + (1) S-Adenosyl-homocysteine_c0[c2] <-> (1) Homocysteine_c0[c2] + (1) Adenosine_c0[c2]	0.000837864	Yes
rxn05106	Cysteine and methionine metabolism	2-hydroxy-5-(methylthio)-3-oxopent-1-enyl phosphate phosphohydrolase	(1) H2O_c0[c2] + (1) 2-Hydroxy-3-keto-5-methylthiopentenyl-1-phosphate_c0[c2] -> (1) Phosphate_c0[c2] + (1) 1,2-dihydroxy-3-keto-5-methylthiopentene_c0[c2]	0.000139644	Yes
rxn05105	Cysteine and methionine metabolism	2,3-diketo-5-methylthiopentyl-1-phosphate keto---enol-isomerase	(1) 2,3-diketo5-methylthio-1-phosphopentane_c0[c2] <-> (1) 2-Hydroxy-3-keto-5-methylthiopentenyl-1-phosphate_c0[c2]	0.000139644	Yes
rxn00693	Cysteine and methionine metabolism|One carbon pool by folate	5-Methyltetrahydrofolate:L-homocysteine S-methyltransferase	(1) Homocysteine_c0[c2] + (1) 5-Methyltetrahydrofolate_c0[c2] <-> (1) L-Methionine_c0[c2] + (1) Tetrahydrofolate_c0[c2]	-0.00301511	Yes
rxn00283	D-Alanine metabolism	alanine racemase	(1) L-Alanine_c0[c2] <-> (1) D-Alanine_c0[c2]	0.00225585	Yes
rxn00193	D-Glutamine and D-glutamate metabolism	glutamate racemase	(1) L-Glutamate_c0[c2] <-> (1) D-Glutamate_c0[c2]	0.00112792	No
rxn02286	D-Glutamine and D-glutamate metabolism|Peptidoglycan biosynthesis	UDP-N-acetylmuramate:L-alanine ligase (ADP-forming)	(1) ATP_c0[c2] + (1) L-Alanine_c0[c2] + (1) UDP-MurNAc_c0[c2] -> (1) ADP_c0[c2] + (1) Phosphate_c0[c2] + (1) H+_c0[c2] + (1) UDP-N-acetylmuramoyl-L-alanine_c0[c2]	0.00112792	Yes

rxn02008	D-Glutamine and D-glutamate metabolism|Peptidoglycan biosynthesis	UDP-N-acetylmuramoyl-L-alanine:D-glutamate ligase(ADP-forming)	(1) ATP_c0[c2] + (1) D-Glutamate_c0[c2] + (1) UDP-N-acetylmuramoyl-L-alanine_c0[c2] -> (1) ADP_c0[c2] + (1) Phosphate_c0[c2] + (1) H+_c0[c2] + (1) UDP-N-acetylmuramoyl-L-alanyl-D-glutamate_c0[c2]	0.00112792	Yes
rxn05345	Fatty acid biosynthesis	dodecanoyl-[acyl-carrier-protein]:malonyl-[acyl-carrier-protein] C-acyltransferase (decarboxylating)	(1) Dodecanoyl-ACP_c0[c2] + (1) Malonyl-acyl-carrierprotein-_c0[c2] -> (1) CO2_c0[c2] + (1) 3-oxotetradecanoyl-acp_c0[c2] + (1) ACP_c0[c2]	0.0045117	Yes
rxn05465	Fatty acid biosynthesis	Malonyl-CoA:[acyl-carrier-protein] S-malonyltransferase	(1) H+_c0[c2] + (1) Malonyl-CoA_c0[c2] + (1) ACP_c0[c2] <-> (1) CoA_c0[c2] + (1) Malonyl-acyl-carrierprotein-_c0[c2]	0.0449716	Yes
rxn05342	Fatty acid biosynthesis	(3R)-3-Hydroxytetradecanoyl-[acyl-carrier-protein]:NADP+ oxidoreductase	(1) NADP_c0[c2] + (1) HMA_c0[c2] <-> (1) NADPH_c0[c2] + (1) 3-oxotetradecanoyl-acp_c0[c2]	-0.0045117	Yes
rxn03245	Fatty acid elongation|Fatty acid degradation	(S)-Hydroxydecanoyl-CoA hydro-lyase	(1) (S)-Hydroxydecanoyl-CoA_c0[c2] <-> (1) H2O_c0[c2] + (1) (2E)-Decenoyl-CoA_c0[c2]	0.0937788	Yes
rxn02911	Fatty acid elongation|Fatty acid degradation	(S)-3-Hydroxydodecanoyl-CoA hydro-lyase	(1) (S)-3-Hydroxydodecanoyl-CoA_c0[c2] <-> (1) H2O_c0[c2] + (1) (2E)-Dodecenoyl-CoA_c0[c2]	0.0937788	Yes
rxn06777	Fatty acid elongation|Fatty acid degradation	(S)-3-Hydroxytetradecanoyl-CoA:NAD+ oxidoreductase	(1) NAD_c0[c2] + (1) (S)-3-Hydroxytetradecanoyl-CoA_c0[c2] <-> (1) NADH_c0[c2] + (1) H+_c0[c2] + (1) 3-Oxotetradecanoyl-CoA_c0[c2]	-0.0937788	Yes
rxn03244	Fatty acid elongation|Fatty acid degradation	(S)-hydroxydecanoyl-CoA:NAD+ oxidoreductase	(1) NAD_c0[c2] + (1) (S)-Hydroxydecanoyl-CoA_c0[c2] <-> (1) NADH_c0[c2] + (1) H+_c0[c2] + (1) 3-Oxodecanoyl-CoA_c0[c2]	-0.0937788	Yes
rxn02680	Fatty acid elongation|Fatty acid degradation	Octanoyl-CoA:acetyl-CoA C-acyltransferase	(1) Acetyl-CoA_c0[c2] + (1) Octanoyl-CoA_c0[c2] <-> (1) CoA_c0[c2] + (1) 3-Oxodecanoyl-CoA_c0[c2]	0.0937788	Yes
rxn03243	Fatty acid elongation|Fatty acid degradation	Decanoyl-CoA:acetyl-CoA C-acyltransferase	(1) Acetyl-CoA_c0[c2] + (1) Decanoyl-CoA_c0[c2] <-> (1) CoA_c0[c2] + (1) 3-Oxododecanoyl-CoA_c0[c2]	0.0937788	Yes
rxn03242	Fatty acid elongation|Fatty acid degradation	(S)-3-hydroxydodecanoyl-CoA:NAD+ oxidoreductase	(1) NAD_c0[c2] + (1) (S)-3-Hydroxydodecanoyl-CoA_c0[c2] <-> (1) NADH_c0[c2] + (1) H+_c0[c2] + (1) 3-Oxododecanoyl-CoA_c0[c2]	-0.0937788	Yes
rxn03241	Fatty acid elongation|Fatty acid degradation	(S)-3-Hydroxytetradecanoyl-CoA hydro-lyase	(1) (S)-3-Hydroxytetradecanoyl-CoA_c0[c2] <-> (1) H2O_c0[c2] + (1) (2E)-Tetradecenoyl-CoA_c0[c2]	0.0937788	Yes
rxn06510	Fatty acid elongation|Fatty acid degradation	Lauroyl-CoA:acetyl-CoA C-acyltransferase	(1) Acetyl-CoA_c0[c2] + (1) Lauroyl-CoA_c0[c2] <-> (1) CoA_c0[c2] + (1) 3-Oxotetradecanoyl-CoA_c0[c2]	0.0937788	Yes
rxn01492	Fructose and mannose metabolism	ATP:D-fructose-1-phosphate 6-phosphotransferase	(1) ATP_c0[c2] + (1) D-fructose-1-phosphate_c0[c2] <-> (1) ADP_c0[c2] + (1) H+_c0[c2] + (1) D-fructose-1,6-bisphosphate_c0[c2]	-0.265088	No
rxn00650	Glutathione metabolism	L-cysteinylglycine dipeptidase	(1) H2O_c0[c2] + (1) Cys-Gly_c0[c2] <-> (1) Glycine_c0[c2] + (1) L-Cysteine_c0[c2]	-0.000139644	Yes
rxn00350	Glutathione metabolism	glutathione gamma-glutamylaminopeptidase	(1) H2O_c0[c2] + (1) GSH_c0[c2] <-> (1) L-Glutamate_c0[c2] + (1) Cys-Gly_c0[c2]	-0.000139644	Yes

rxn00539	Glycerophospholipid metabolism	ethanolamine ammonia-lyase (acetaldehyde-forming)	(1) Aminoethanol_c0[c2] -> (1) NH3_c0[c2] + (1) Acetaldehyde_c0[c2]	0.00538627	Yes
rxn00611	Glycerophospholipid metabolism	sn-Glycerol-3-phosphate:NAD+ 2-oxidoreductase	(1) NAD_c0[c2] + (1) Glycerol-3-phosphate_c0[c2] <-> (1) NADH_c0[c2] + (1) H+_c0[c2] + (1) Glycerone-phosphate_c0[c2]	-0.104373	Yes
rxn01300	Glycine, serine and threonine metabolism	ATP:L-homoserine O-phosphotransferase	(1) ATP_c0[c2] + (1) L-Homoserine_c0[c2] <-> (1) ADP_c0[c2] + (1) H+_c0[c2] + (1) O-Phospho-L-homoserine_c0[c2]	0.052813	Yes
rxn01069	Glycine, serine and threonine metabolism	O-phospho-L-homoserine phosphate-lyase (adding water;L-threonine-forming)	(1) H2O_c0[c2] + (1) O-Phospho-L-homoserine_c0[c2] -> (1) Phosphate_c0[c2] + (1) L-Threonine_c0[c2]	0.052813	Yes
rxn00541	Glycine, serine and threonine metabolism	L-threonine acetaldehyde-lyase (glycine-forming)	(1) L-Threonine_c0[c2] <-> (1) Glycine_c0[c2] + (1) Acetaldehyde_c0[c2]	0.0432941	Yes
rxn00692	Glycine, serine and threonine metabolism|Cyanoamino acid metabolism|Glyoxylate and dicarboxylate metabolism|One carbon pool by folate|Methane metabolism	5,10-Methylenetetrahydrofolate:glycine hydroxymethyltransferase	(1) H2O_c0[c2] + (1) Glycine_c0[c2] + (1) 5-10-Methylenetetrahydrofolate_c0[c2] <-> (1) L-Serine_c0[c2] + (1) Tetrahydrofolate_c0[c2]	0.00205813	Yes
rxn00742	Glycine, serine and threonine metabolism|Cysteine and methionine metabolism	L-cystathionine cysteine-lyase (deaminating; 2-oxobutanoate-forming)	(1) H2O_c0[c2] + (1) Cystathionine_c0[c2] -> (1) NH3_c0[c2] + (1) L-Cysteine_c0[c2] + (1) 2-Oxobutyrate_c0[c2]	0.00385297	Yes
rxn00337	Glycine, serine and threonine metabolism|Cysteine and methionine metabolism|Lysine biosynthesis	ATP:L-aspartate 4-phosphotransferase	(1) ATP_c0[c2] + (1) L-Aspartate_c0[c2] <-> (1) ADP_c0[c2] + (1) 4-Phospho-L-aspartate_c0[c2]	0.052813	Yes
rxn01301	Glycine, serine and threonine metabolism|Cysteine and methionine metabolism|Lysine biosynthesis	L-Homoserine:NAD+ oxidoreductase	(1) NAD_c0[c2] + (1) L-Homoserine_c0[c2] <-> (1) NADH_c0[c2] + (1) H+_c0[c2] + (1) L-Aspartate4-semialdehyde_c0[c2]	-0.052813	Yes
rxn01643	Glycine, serine and threonine metabolism|Cysteine and methionine metabolism|Lysine biosynthesis	L-Aspartate-4-semialdehyde:NADP+ oxidoreductase (phosphorylating)	(1) NADP_c0[c2] + (1) Phosphate_c0[c2] + (1) L-Aspartate4-semialdehyde_c0[c2] <- (1) NADPH_c0[c2] + (1) H+_c0[c2] + (1) 4-Phospho-L-aspartate_c0[c2]	-0.052813	Yes
rxn02342	Glycolysis / Gluconeogenesis|Citrate cycle (TCA cycle)|Pyruvate metabolism	R03270	(1) Lipoamide_c0[c2] + (1) 2-Hydroxyethyl-ThPP_c0[c2] <-> (1) TPP_c0[c2] + (1) S-Acetyldihydrolipoamide_c0[c2]	0.834994	Yes
rxn01871	Glycolysis / Gluconeogenesis|Citrate cycle (TCA cycle)|Pyruvate metabolism	acetyl-CoA:enzyme N6-(dihydrolipoyl)lysine S-acetyltransferase	(1) Acetyl-CoA_c0[c2] + (1) Dihydrolipoamide_c0[c2] <-> (1) CoA_c0[c2] + (1) S-Acetyldihydrolipoamide_c0[c2]	-0.834994	Yes
rxn00011	Glycolysis / Gluconeogenesis|Citrate cycle (TCA cycle)|Valine, leucine and isoleucine biosynthesis|Pyruvate metabolism|Butanoate metabolism	pyruvate:thiamin diphosphate acetaldehydetransferase (decarboxylating)	(1) CO2_c0[c2] + (1) 2-Hydroxyethyl-ThPP_c0[c2] <- (1) Pyruvate_c0[c2] + (1) TPP_c0[c2] + (1) H+_c0[c2]	-0.838847	Yes
rxn00747	Glycolysis / Gluconeogenesis|Fructose and mannose metabolism|Inositol phosphate metabolism|Carbon	D-glyceraldehyde-3-phosphate aldose-ketose-isomerase	(1) Glyceraldehyde3-phosphate_c0[c2] <-> (1) Glycerone-phosphate_c0[c2]	0.465188	Yes

	fixation in photosynthetic organisms				
rxn00704	Glycolysis / Gluconeogenesis|Galactose metabolism|Starch and sucrose metabolism|Amino sugar and nucleotide sugar metabolism	alpha-D-Glucose 1-phosphate 1,6-phosphomutase	(1) Glucose-1-phosphate_c0[c2] <-> (1) D-glucose-6-phosphate_c0[c2]	-0.00563962	Yes
rxn00175	Glycolysis / Gluconeogenesis|Methane metabolism|Carbon fixation pathways in prokaryotes	Acetate:CoA ligase (AMP-forming)	(1) ATP_c0[c2] + (1) CoA_c0[c2] + (1) Acetate_c0[c2] <-> (1) PPi_c0[c2] + (1) AMP_c0[c2] + (1) Acetyl-CoA_c0[c2] + (1) H+_c0[c2]	-0.0044245	Yes
rxn00148	Glycolysis / Gluconeogenesis|Purine metabolism|Pyruvate metabolism|Carbon fixation in photosynthetic organisms	ATP:pyruvate 2-O-phosphotransferase	(1) ATP_c0[c2] + (1) Pyruvate_c0[c2] <-> (1) ADP_c0[c2] + (1) Phosphoenolpyruvate_c0[c2] + (1) H+_c0[c2]	0.994429	Yes
rxn00506	Glycolysis / Gluconeogenesis|Pyruvate metabolism	Acetaldehyde:NAD+ oxidoreductase	(1) H2O_c0[c2] + (1) NAD_c0[c2] + (1) Acetaldehyde_c0[c2] -> (1) NADH_c0[c2] + (1) Acetate_c0[c2] + (2) H+_c0[c2]	0.0486803	Yes
rxn00499	Glycolysis / Gluconeogenesis|Pyruvate metabolism	(S)-Lactate:NAD+ oxidoreductase	(1) NAD_c0[c2] + (1) L-Lactate_c0[c2] <-> (1) NADH_c0[c2] + (1) Pyruvate_c0[c2] + (1) H+_c0[c2]	2.87736	No
rxn00333	Glyoxylate and dicarboxylate metabolism	Glycolate:oxygen 2-oxidoreductase	(1) O2_c0[c2] + (1) Glycolate_c0[c2] -> (1) H2O2_c0[c2] + (1) Glyoxalate_c0[c2]	0.280918	Yes
rxn12822	Glyoxylate and dicarboxylate metabolism|Nitrogen metabolism	L-glutamate:ferredoxin oxidoreductase (transaminating)	(2) L-Glutamate_c0[c2] + (2) Oxidizedferredoxin_c0[c2] <-> (1) 2-Oxoglutarate_c0[c2] + (1) L-Glutamine_c0[c2] + (2) H+_c0[c2] + (2) Reducedferredoxin_c0[c2]	0.471026	No
rxn03181	Lipopolysaccharide biosynthesis	ATP:2,3,2',3'-tetrakis(3-hydroxytetradecanoyl)-D-glucosaminyl-beta-D-1,6-glucosaminyl-alpha-phosphate 4-O'-phosphotransferase	(1) ATP_c0[c2] + (1) Lipid A disaccharide_c0[c2] <-> (1) ADP_c0[c2] + (1) H+_c0[c2] + (1) Lipid IV(A)_c0[c2]	0.00112792	No
rxn02331	Lipopolysaccharide biosynthesis	phosphoenolpyruvate:D-arabinose-5-phosphate C-(1-carboxyvinyl)transferase (phosphate-hydrolysing, 2-carboxy-2-oxoethyl-forming)	(1) Phosphate_c0[c2] + (1) 3-Deoxy-D-manno-octulosonate8-phosphate_c0[c2] <- (1) H2O_c0[c2] + (1) Phosphoenolpyruvate_c0[c2] + (1) D-Arabinose5-phosphate_c0[c2]	-0.00338377	Yes
rxn02404	Lipopolysaccharide biosynthesis	3-Deoxy-D-manno-octulosonate-8-phosphate 8-phosphohydrolase	(1) H2O_c0[c2] + (1) 3-Deoxy-D-manno-octulosonate8-phosphate_c0[c2] -> (1) Phosphate_c0[c2] + (1) KDO_c0[c2]	0.00338377	Yes
rxn03919	Lipopolysaccharide biosynthesis	D-glycero-beta-D-manno-heptose 1,7-bisphosphate 7-phosphohydrolase	(1) H2O_c0[c2] + (1) D-Glycero-D-manno-heptose1-7-bisphosphate_c0[c2] -> (1) Phosphate_c0[c2] + (1) D-Glycero-D-manno-heptose1-phosphate_c0[c2]	0.0045117	No
rxn03159	Lipopolysaccharide biosynthesis	UDP-2,3-bis(3-hydroxytetradecanoyl)glucosamine:2,3-bis-(3-hydroxytetradecanoyl)-alpha-D-glucosaminyl-1-phosphate 2,3-bis(3-hydroxytetradecanoyl)-glucosaminyltransferase	(1) UDP-2,3-bis(3-hydroxytetradecanoyl)glucosamine_c0[c2] + (1) Lipid X_c0[c2] <-> (1) UDP_c0[c2] + (1) Lipid A disaccharide_c0[c2]	0.00112792	Yes

rxn06723	Lipopolysaccharide biosynthesis	(3R)-3-hydroxymyristoyl-[acyl-carrier protein]:UDP-3-O-[(3R)-3-hydroxymyristoyl]-alpha-D-glucosamine N-acetyltransferase	(1) UDP-2,3-bis(3-hydroxytetradecanoyl)glucosamine_c0[c2] + (1) ACP_c0[c2] <- (1) UDP-3-O-(beta-hydroxymyristoyl)-D-glucosamine_c0[c2] + (1) HMA_c0[c2]	-0.00225585	Yes
rxn03146	Lipopolysaccharide biosynthesis	UDP-3-O-[(3R)-3-hydroxymyristoyl]-N-acetylglucosamine amidohydrolase	(1) H2O_c0[c2] + (1) UDP-3-O-(beta-hydroxymyristoyl)-N-acetylglucosamine_c0[c2] <-> (1) Acetate_c0[c2] + (1) UDP-3-O-(beta-hydroxymyristoyl)-D-glucosamine_c0[c2]	0.00225585	Yes
rxn02405	Lipopolysaccharide biosynthesis	CTP:3-deoxy-D-manno-octulosonate cytidylyltransferase	(1) CTP_c0[c2] + (1) KDO_c0[c2] -> (1) PPi_c0[c2] + (1) H+_c0[c2] + (1) CMP-KDO_c0[c2]	0.00338377	Yes
rxn06865	Lipopolysaccharide biosynthesis	R05146	(1) Lauroyl-KDO2-lipid IV(A)_c0[c2] + (1) ACP_c0[c2] <- (1) kdo2-lipid iva_c0[c2] + (1) Dodecanoyl-ACP_c0[c2]	-0.00112792	Yes
rxn06729	Lipopolysaccharide biosynthesis	(R)-3-Hydroxytetradecanoly-[acyl-carrier-protein]:UDP-N-acetyl-glucosamine 3-O-(3-hydroxytetradecanoyl)transferase	(1) UDP-N-acetylglucosamine_c0[c2] + (1) H+_c0[c2] + (1) HMA_c0[c2] -> (1) UDP-3-O-(beta-hydroxymyristoyl)-N-acetylglucosamine_c0[c2] + (1) ACP_c0[c2]	0.00225585	Yes
rxn03130	Lipopolysaccharide biosynthesis	UDP-2,3-bis[(3R)-3-hydroxymyristoyl]-alpha-D-glucosamine 2,3-bis[(3R)-3-hydroxymyristoyl]-beta-D-glucosaminyl 1-phosphate phosphohydrolase	(1) H2O_c0[c2] + (1) UDP-2,3-bis(3-hydroxytetradecanoyl)glucosamine_c0[c2] -> (2) H+_c0[c2] + (1) UMP_c0[c2] + (1) Lipid X_c0[c2]	0.00112792	Yes
rxn03916	Lipopolysaccharide biosynthesis	ATP:D-glycero-beta-D-manno-heptose 1-phosphate adenylyltransferase	(1) ATP_c0[c2] + (1) D-Glycero-D-manno-heptose1-phosphate_c0[c2] -> (1) PPi_c0[c2] + (1) ADP-D-glycero-D-manno-heptose[c2]	0.0045117	No
rxn03182	Lipopolysaccharide biosynthesis	CMP-3-deoxy-D-manno-oct-2-ulosonate:lipid IVA 3-deoxy-D-manno-oct-2-ulosonate transferase	(1) CMP-KDO_c0[c2] + (1) Lipid IV(A)_c0[c2] -> (1) CMP_c0[c2] + (1) H+_c0[c2] + (1) KDO-lipid IV(A)[c2]	0.00112792	No
rxn06848	Lipopolysaccharide biosynthesis	R05075	(1) kdo2-lipid a_c0[c2] + (1) ACP_c0[c2] <- (1) Lauroyl-KDO2-lipid IV(A)_c0[c2] + (1) Myristoyl-ACP_c0[c2]	-0.00112792	No
rxn03439	Lipopolysaccharide biosynthesis	CMP-3-deoxy-D-manno-oct-2-ulosonate:(KDO)-lipid IVA 3-deoxy-D-manno-oct-2-ulosonate transferase	(1) CMP-KDO_c0[c2] + (1) KDO-lipid IV(A)[c2] -> (1) CMP_c0[c2] + (1) H+_c0[c2] + (1) kdo2-lipid iva_c0[c2]	0.00112792	No
rxn03511	Lipopolysaccharide biosynthesis	ADP-D-glycero-D-manno-heptose 6-epimerase	(1) ADP-D-glycero-D-manno-heptose[c2] -> (1) ADP-L-glycero-D-manno-heptose_c0[c2]	0.0045117	No
rxn03917	Lipopolysaccharide biosynthesis	D-glycero-beta-D-manno-heptose-7-phosphate aldose-ketose-isomerase	(1) Sedoheptulose7-phosphate_c0[c2] -> (1) D-Glycero-D-manno-heptose7-phosphate[c2]	0.0045117	No
rxn03918	Lipopolysaccharide biosynthesis	ATP:D-glycero-beta-D-manno-heptose 7-phosphate 1-phosphotransferase	(1) ATP_c0[c2] + (1) D-Glycero-D-manno-heptose7-phosphate[c2] -> (1) ADP_c0[c2] + (1) H+_c0[c2] + (1) D-Glycero-D-manno-heptose1-7-bisphosphate_c0[c2]	0.0045117	No

rxn00313	Lysine biosynthesis	meso-2,6-diaminoheptanedioate carboxy-lyase (L-lysine-forming)	(1) H+_c0[c2] + (1) meso-2,6-Diaminopimelate_c0[c2] <-> (1) CO2_c0[c2] + (1) L-Lysine_c0[c2]	-0.00112792	Yes
rxn02011	Lysine biosynthesis|Peptidoglycan biosynthesis	UDP-N-acetylmuramoyl-L-alanyl-D-glutamate:(L)-meso-2,6-diaminoheptanedioate gamma-ligase (ADP-forming)	(1) ATP_c0[c2] + (1) meso-2,6-Diaminopimelate_c0[c2] + (1) UDP-N-acetylmuramoyl-L-alanyl-D-glutamate_c0[c2] -> (1) ADP_c0[c2] + (1) Phosphate_c0[c2] + (1) H+_c0[c2] + (1) UDP-N-acetylmuramoyl-L-alanyl-D-gamma-glutamyl-meso-2-6-diaminopimelate_c0[c2]	0.00112792	Yes
rxn03164	Lysine biosynthesis|Peptidoglycan biosynthesis	UDP-N-acetylmuramoyl-L-alanyl-D-glutamyl-meso-2,6-diaminoheptanedioate:D-alanyl-D-alanine ligase(ADP-forming)	(1) ATP_c0[c2] + (1) Ala-Ala[c2] + (1) UDP-N-acetylmuramoyl-L-alanyl-D-gamma-glutamyl-meso-2-6-diaminopimelate_c0[c2] -> (1) ADP_c0[c2] + (1) Phosphate_c0[c2] + (1) H+_c0[c2] + (1) UDP-N-acetylmuramoyl-L-alanyl-D-glutamyl-6-carboxy-L-lysyl-D-alanyl- D-alanine_c0[c2]	0.00112792	No
rxn00549	Methane metabolism|Carbon fixation in photosynthetic organisms	D-Fructose-1,6-bisphosphate 1-phosphohydrolase	(1) H2O_c0[c2] + (1) D-fructose-1,6-bisphosphate_c0[c2] -> (1) Phosphate_c0[c2] + (1) D-fructose-6-phosphate_c0[c2]	0.0954481	Yes
rxn00786	Methane metabolism|Carbon fixation in photosynthetic organisms	D-fructose-1,6-bisphosphate D-glyceraldehyde-3-phosphate-lyase (glycerone-phosphate-forming)	(1) D-fructose-1,6-bisphosphate_c0[c2] <-> (1) Glycerone-phosphate_c0[c2] + (1) Glyceraldehyde3-phosphate_c0[c2]	-0.360536	Yes
rxn02988	Nicotinate and nicotinamide metabolism	glycerone phosphate:iminosuccinate alkyltransferase (cyclizing)	(2) H2O_c0[c2] + (1) Phosphate_c0[c2] + (1) Quinolinate_c0[c2] <- (1) Glycerone-phosphate_c0[c2] + (1) Iminoaspartate_c0[c2]	-0.000279288	Yes
rxn02402	Nicotinate and nicotinamide metabolism	Nicotinate-nucleotide:pyrophosphate phosphoribosyltransferase (carboxylating)	(1) CO2_c0[c2] + (1) PPi_c0[c2] + (1) Nicotinate ribonucleotide_c0[c2] <- (1) H+_c0[c2] + (1) PRPP_c0[c2] + (1) Quinolinate_c0[c2]	-0.000279288	Yes
rxn02155	Nicotinate and nicotinamide metabolism	ATP:nicotinamide-nucleotide adenylyltransferase	(1) ATP_c0[c2] + (1) Nicotinate ribonucleotide_c0[c2] <-> (1) PPi_c0[c2] + (1) Deamido-NAD_c0[c2]	0.000279288	Yes
rxn00083	Nicotinate and nicotinamide metabolism	NADPH:NAD+ oxidoreductase	(1) NAD_c0[c2] + (1) NADPH_c0[c2] <-> (1) NADH_c0[c2] + (1) NADP_c0[c2]	-0.352761	Yes
rxn01265	Nicotinate and nicotinamide metabolism	Nicotinate D-ribonucleotide:diphosphate phosphoribosyltransferase	(1) PPi_c0[c2] + (1) Nicotinate ribonucleotide_c0[c2] <-> (1) PRPP_c0[c2] + (1) Niacin_c0[c2]	-0.000139644	Yes
rxn00077	Nicotinate and nicotinamide metabolism	ATP:NAD+ 2'-phosphotransferase	(1) ATP_c0[c2] + (1) NAD_c0[c2] <-> (1) NADP_c0[c2] + (1) ADP_c0[c2] + (1) H+_c0[c2]	0.000139644	Yes
rxn00338	Nicotinate and nicotinamide metabolism	L-aspartate:oxygen oxidoreductase	(1) O2_c0[c2] + (1) L-Aspartate_c0[c2] -> (1) H2O2_c0[c2] + (1) H+_c0[c2] + (1) Iminoaspartate_c0[c2]	0.000279288	Yes
rxn00138	Nicotinate and nicotinamide metabolism	deamido-NAD+:ammonia ligase (AMP-forming)	(1) ATP_c0[c2] + (1) NH3_c0[c2] + (1) Deamido-NAD_c0[c2] -> (1) NAD_c0[c2] + (1) PPi_c0[c2] + (1) AMP_c0[c2] + (2) H+_c0[c2]	0.000279288	Yes
rxn00102	Nitrogen metabolism	carbonate hydro-lyase (carbon-dioxide-forming)	(1) H+_c0[c2] + (1) H2CO3_c0[c2] <-> (1) H2O_c0[c2] + (1) CO2_c0[c2]	-0.470844	Yes
rxn01602	One carbon pool by folate|Folate biosynthesis	dihydrofolate:NADP+ oxidoreductase	(1) NADP_c0[c2] + (1) Dihydrofolate_c0[c2] <-> (1)	-0.000418932	Yes

			NADPH_c0[c2] + (1) H+_c0[c2] + (1) Folate_c0[c2]		
rxn00686	One carbon pool by folate|Folate biosynthesis	5,6,7,8-tetrahydrofolate:NADP+ oxidoreductase	(1) NADP_c0[c2] + (1) Tetrahydrofolate_c0[c2] <-> (1) NADPH_c0[c2] + (1) H+_c0[c2] + (1) Dihydrofolate_c0[c2]	-0.000956977	Yes
rxn00910	One carbon pool by folate|Methane metabolism	5-methyltetrahydrofolate:NADP+ oxidoreductase	(1) NADP_c0[c2] + (1) 5-Methyltetrahydrofolate_c0[c2] <-> (1) NADPH_c0[c2] + (1) H+_c0[c2] + (1) 5-10-Methylenetetrahydrofolate_c0[c2]	0.00287547	Yes
rxn12512	Pantothenate and CoA biosynthesis	(R)-4'-Phosphopantothenate:L-cysteine ligase	(1) ATP_c0[c2] + (1) L-Cysteine_c0[c2] + (1) 4-phosphopantothenate_c0[c2] -> (1) PPi_c0[c2] + (1) AMP_c0[c2] + (2) H+_c0[c2] + (1) (R)-4'-Phosphopantothenoyl-L-cysteine_c0[c2]	0.000279288	Yes
rxn00100	Pantothenate and CoA biosynthesis	ATP:dephospho-CoA 3'-phosphotransferase	(1) ATP_c0[c2] + (1) Dephospho-CoA_c0[c2] -> (1) ADP_c0[c2] + (1) CoA_c0[c2] + (1) H+_c0[c2]	0.000279288	Yes
rxn02175	Pantothenate and CoA biosynthesis	ATP:pantetheine-4'-phosphate adenylyltransferase	(1) ATP_c0[c2] + (1) Phosphopantetheine_c0[c2] <-> (1) PPi_c0[c2] + (1) Dephospho-CoA_c0[c2]	0.000279288	Yes
rxn06023	Pantothenate and CoA biosynthesis	CoA:apo-[acyl-carrier-protein] pantetheinephosphotransferase	(1) CoA_c0[c2] + (1) apo-ACP_c0[c2] <-> (1) Adenosine 3-5-bisphosphate_c0[c2] + (1) ACP_c0[c2]	0.000139644	Yes
rxn12510	Pantothenate and CoA biosynthesis	ATP:pantothenate 4'-phosphotransferase	(1) ATP_c0[c2] + (1) PAN_c0[c2] <-> (1) ADP_c0[c2] + (1) H+_c0[c2] + (1) 4-phosphopantothenate_c0[c2]	0.000279288	Yes
rxn01790	Pantothenate and CoA biosynthesis	(R)-Pantoate:NADP+ 2-oxidoreductase	(1) NADP_c0[c2] + (1) Pantoate_c0[c2] <-> (1) NADPH_c0[c2] + (1) H+_c0[c2] + (1) 2-Dehydropantoate_c0[c2]	-0.000279288	Yes
rxn00912	Pantothenate and CoA biosynthesis	5,10-Methylenetetrahydrofolate: 3-methyl-2-oxobutanoate hydroxymethyltransferase	(1) H2O_c0[c2] + (1) 3-Methyl-2-oxobutanoate_c0[c2] + (1) 5-10-Methylenetetrahydrofolate_c0[c2] <-> (1) Tetrahydrofolate_c0[c2] + (1) 2-Dehydropantoate_c0[c2]	0.000279288	Yes
rxn02341	Pantothenate and CoA biosynthesis	N-[(R)-4'-Phosphopantothenoyl]-L-cysteine carboxy-lyase	(1) H+_c0[c2] + (1) (R)-4'-Phosphopantothenoyl-L-cysteine_c0[c2] -> (1) CO2_c0[c2] + (1) Phosphopantetheine_c0[c2]	0.000279288	Yes
rxn00213	Pentose and glucuronate interconversions|Galactose metabolism|Starch and sucrose metabolism|Amino sugar and nucleotide sugar metabolism	UTP:alpha-D-glucose-1-phosphate uridylyltransferase	(1) UTP_c0[c2] + (1) Glucose-1-phosphate_c0[c2] <-> (1) PPi_c0[c2] + (1) UDP-glucose_c0[c2]	0.0045117	Yes
rxn00772	Pentose phosphate pathway	ATP:D-ribose 5-phosphotransferase	(1) ATP_c0[c2] + (1) D-Ribose_c0[c2] <-> (1) ADP_c0[c2] + (1) H+_c0[c2] + (1) ribose-5-phosphate_c0[c2]	0.000837864	No
rxn01477	Pentose phosphate pathway	6-Phospho-D-gluconate hydro-lyase(2-dehydro-3-deoxy-6-phospho-D-gluconate-forming)	(1) 6-Phospho-D-gluconate_c0[c2] -> (1) H2O_c0[c2] + (1) 2-Keto-3-deoxy-6-phosphogluconate_c0[c2]	1.27405	Yes
rxn01333	Pentose phosphate pathway	sedoheptulose-7-phosphate:D-glyceraldehyde-3-phosphate glyceronetransferase	(1) Glyceraldehyde3-phosphate_c0[c2] + (1) Sedoheptulose7-phosphate_c0[c2] <-> (1) D-fructose-6-phosphate_c0[c2] + (1) D-Erythrose4-phosphate_c0[c2]	-0.436384	Yes

rxn00777	Pentose phosphate pathway|Carbon fixation in photosynthetic organisms	D-ribose-5-phosphate aldose-ketose-isomerase	(1) ribose-5-phosphate_c0[c2] <-> (1) D-Ribulose5-phosphate_c0[c2]	0.408622	Yes
rxn01200	Pentose phosphate pathway|Carbon fixation in photosynthetic organisms	Sedoheptulose-7-phosphate:D-glyceraldehyde-3-phosphate glycolaldehyde transferase	(1) Glyceraldehyde3-phosphate_c0[c2] + (1) Sedoheptulose7-phosphate_c0[c2] <-> (1) ribose-5-phosphate_c0[c2] + (1) D-Xylulose5-phosphate_c0[c2]	0.431872	Yes
rxn03884	Pentose phosphate pathway|Pentose and glucuronate interconversions	2-dehydro-3-deoxy-D-gluconate-6-phosphate D-glyceraldehyde-3-phosphate-lyase	(1) 2-Keto-3-deoxy-6-phosphogluconate_c0[c2] <-> (1) Pyruvate_c0[c2] + (1) Glyceraldehyde3-phosphate_c0[c2]	1.27405	Yes
rxn01116	Pentose phosphate pathway|Pentose and glucuronate interconversions|Carbon fixation in photosynthetic organisms	D-Ribulose-5-phosphate 3-epimerase	(1) D-Ribulose5-phosphate_c0[c2] <-> (1) D-Xylulose5-phosphate_c0[c2]	-0.868815	Yes
rxn00770	Pentose phosphate pathway|Purine metabolism	ATP:D-ribose-5-phosphate diphosphotransferase	(1) ATP_c0[c2] + (1) ribose-5-phosphate_c0[c2] <-> (1) AMP_c0[c2] + (1) H+_c0[c2] + (1) PRPP_c0[c2]	0.0231109	Yes
rxn03408	Peptidoglycan biosynthesis	UDP-N-acetyl-D-glucosamine:undecaprenyl-diphospho-N-acetylmuramoyl-L-alanyl-gamma-D-glutamyl-meso-2,6-diaminopimeloyl-D-alanyl-D-alanine 4-beta-N-acetylglucosaminlytransferase	(1) UDP-N-acetylglucosamine_c0[c2] + (1) Undecaprenyl-diphospho-N-acetylmuramoyl-L-alanyl-D-glutamyl-meso-2-6-diaminopimeloyl-D-alanyl-D-alanine_c0[c2] <-> (1) UDP_c0[c2] + (1) Undecaprenyl-diphospho-N-acetylmuramoyl--N-acetylglucosamine-L-ala-D-glu-meso-2-6-diaminopimeloyl-D-ala-D-ala_c0[c2]	0.00112792	No
rxn03904	Peptidoglycan biosynthesis	UDP-N-acetylmuramoyl-L-alanyl-gamma-D-glutamyl-meso-2,6-diaminopimeloyl-D-alanyl-D-alanine:undecaprenyl-phosphate phospho-N-acetylmuramoyl-pentapeptide-transferase	(1) Undecaprenylphosphate_c0[c2] + (1) UDP-N-acetylmuramoyl-L-alanyl-D-glutamyl-6-carboxy-L-lysyl-D-alanyl- D-alanine_c0[c2] <-> (1) UMP_c0[c2] + (1) Undecaprenyl-diphospho-N-acetylmuramoyl-L-alanyl-D-glutamyl-meso-2-6-diaminopimeloyl-D-alanyl-D-alanine_c0[c2]	0.00112792	Yes
rxn03901	Peptidoglycan biosynthesis	undecaprenyl-diphosphate phosphohydrolase	(1) H2O_c0[c2] + (1) Bactoprenyl diphosphate_c0[c2] -> (1) Phosphate_c0[c2] + (2) H+_c0[c2] + (1) Undecaprenylphosphate_c0[c2]	0.00112792	No
rxn01739	Phenylalanine, tyrosine and tryptophan biosynthesis	ATP:shikimate 3-phosphotransferase	(1) ATP_c0[c2] + (1) Shikimate_c0[c2] <-> (1) ADP_c0[c2] + (1) H+_c0[c2] + (1) 3-phosphoshikimate_c0[c2]	0.000418932	Yes
rxn02212	Phenylalanine, tyrosine and tryptophan biosynthesis	2-Dehydro-3-deoxy-D-arabino-heptonate 7-phosphate phosphate-lyase (cyclyzing)	(1) DAHP_c0[c2] -> (1) Phosphate_c0[c2] + (1) 5-Dehydroquinate_c0[c2]	0.000418932	Yes
rxn01740	Phenylalanine, tyrosine and tryptophan biosynthesis	Shikimate:NADP+ 3-oxidoreductase	(1) NADP_c0[c2] + (1) Shikimate_c0[c2] <-> (1) NADPH_c0[c2] + (1) H+_c0[c2] + (1) 3-Dehydroshikimate_c0[c2]	-0.000418932	Yes
rxn01255	Phenylalanine, tyrosine and tryptophan biosynthesis	5-O-(1-Carboxyvinyl)-3-phosphoshikimate phosphate-lyase (chorismate-forming)	(1) 5-O--1-Carboxyvinyl-3-phosphoshikimate_c0[c2] -> (1) Phosphate_c0[c2] + (1) Chorismate_c0[c2]	0.000418932	Yes
rxn02476	Phenylalanine, tyrosine and tryptophan biosynthesis	Phosphoenolpyruvate:3-phosphoshikimate 5-O-(1-carboxyvinyl)-transferase	(1) Phosphoenolpyruvate_c0[c2] + (1) 3-phosphoshikimate_c0[c2] <-> (1) Phosphate_c0[c2] + (1) 5-O--1-Carboxyvinyl-3-phosphoshikimate_c0[c2]	0.000418932	Yes

rxn02213	Phenylalanine, tyrosine and tryptophan biosynthesis	3-Dehydroquinate hydro-lyase	(1) 5-Dehydroquinate_c0[c2] -> (1) H2O_c0[c2] + (1) 3-Dehydroshikimate_c0[c2]	0.000418932	Yes
rxn01332	Phenylalanine, tyrosine and tryptophan biosynthesis	Phosphoenolpyruvate:D-erythrose-4-phosphate C-(1-carboxyvinyl)transferase (phosphate hydrolysing, 2-carboxy-2-oxoethyl-forming)	(1) H2O_c0[c2] + (1) Phosphoenolpyruvate_c0[c2] + (1) D-Erythrose4-phosphate_c0[c2] -> (1) Phosphate_c0[c2] + (1) DAHP_c0[c2]	0.000418932	Yes
rxn00060	Porphyrin and chlorophyll metabolism	porphobilinogen:(4-[2-carboxyethyl]-3-[carboxymethyl]pyrrol-2-yl)methyltransferase (hydrolysing)	(1) H2O_c0[c2] + (4) Porphobilinogen_c0[c2] -> (4) NH3_c0[c2] + (1) Hydroxymethylbilane_c0[c2]	0.000279288	Yes
rxn02264	Porphyrin and chlorophyll metabolism	Hydroxymethylbilane hydro-lyase(cyclizing)	(1) Hydroxymethylbilane_c0[c2] <-> (1) H2O_c0[c2] + (1) UroporphyrinogenIII_c0[c2]	0.000279288	No
rxn00029	Porphyrin and chlorophyll metabolism	5-aminolevulinate hydro-lyase (adding 5-aminolevulinate and cyclizing; porphobilinogen-forming)	(2) 5-Aminolevulinate_c0[c2] -> (2) H2O_c0[c2] + (1) H+_c0[c2] + (1) Porphobilinogen_c0[c2]	0.00111715	Yes
rxn01629	Porphyrin and chlorophyll metabolism	(S)-4-Amino-5-oxopentanoate 4,5-aminomutase	(1) 5-Aminolevulinate_c0[c2] <-> (1) L-Glutamate1-semialdehyde_c0[c2]	-0.0022343	Yes
rxn03537	Porphyrin and chlorophyll metabolism	R05222	(1) GTP_c0[c2] + (1) Adenosyl cobinamide phosphate_c0[c2] <-> (1) PPi_c0[c2] + (1) H+_c0[c2] + (1) Adenosylcobinamide-GDP_c0[c2]	0.000139644	No
rxn04413	Porphyrin and chlorophyll metabolism	R06558	(1) GTP_c0[c2] + (1) Adenosyl cobinamide_c0[c2] <-> (1) GDP_c0[c2] + (1) H+_c0[c2] + (1) Adenosyl cobinamide phosphate_c0[c2]	0.000139644	No
rxn03150	Porphyrin and chlorophyll metabolism	R04594	(1) H2O_c0[c2] + (1) alpha-Ribazole 5'-phosphate_c0[c2] -> (1) Phosphate_c0[c2] + (1) alpha-Ribazole_c0[c2]	0.000139644	Yes
rxn05029	Porphyrin and chlorophyll metabolism	ATP:cobinamide Cobeta-adenosyltransferase	(1) ATP_c0[c2] + (1) H+_c0[c2] + (1) Cobinamide_c0[c2] <-> (1) Triphosphate_c0[c2] + (1) Adenosyl cobinamide_c0[c2]	0.000139644	Yes
rxn02303	Porphyrin and chlorophyll metabolism	Coproporphyrinogen:oxyge n oxidoreductase(decarboxyla ting)	(1) O2_c0[c2] + (2) H+_c0[c2] + (1) CoproporphyrinogenIII_c0[c2] <-> (2) H2O_c0[c2] + (2) CO2_c0[c2] + (1) ProtoporphyrinogenIX_c0[c2]	0.000139644	Yes
rxn03538	Porphyrin and chlorophyll metabolism	R05223	(1) alpha-Ribazole_c0[c2] + (1) Adenosylcobinamide-GDP_c0[c2] <-> (1) H+_c0[c2] + (1) GMP_c0[c2] + (1) Calomide_c0[c2]	0.000139644	No
rxn00056	Porphyrin and chlorophyll metabolism	Fe(II):oxygen oxidoreductase	(1) O2_c0[c2] + (4) H+_c0[c2] + (4) Fe2+_c0[c2] <-> (2) H2O_c0[c2] + (4) fe3_c0[c2]	-0.000104733	Yes
rxn02897	Porphyrin and chlorophyll metabolism	Nicotinate-nucleotide:dimethylbenzimi dazole phospho-D-ribosyltransferase	(1) Nicotinate ribonucleotide_c0[c2] + (1) Dimethylbenzimidazole_c0[c2] <-> (1) H+_c0[c2] + (1) Niacin_c0[c2] + (1) alpha-Ribazole 5'-phosphate_c0[c2]	0.000139644	Yes
rxn02056	Porphyrin and chlorophyll metabolism	S-Adenosyl-L-methionine:uroporphyrin-III C-methyltransferase	(2) H+_c0[c2] + (1) Siroheme_c0[c2] <-> (1) Sirohydrochlorin_c0[c2] + (1) Fe2+_c0[c2]	-0.000139644	Yes
rxn02288	Porphyrin and chlorophyll metabolism	Uroporphyrinogen-III carboxy-lyase	(4) H+_c0[c2] + (1) UroporphyrinogenIII_c0[c2] <-> (4)	0.000139644	Yes

			CO2_c0[c2] + (1) CoproporphyrinogenIII_c0[c2]		
rxn06591	Porphyrin and chlorophyll metabolism	L-glutamate-semialdehyde: NADP+ oxidoreductase(L-glutamyl-tRNA(Glu)-forming)	(1) NADPH_c0[c2] + (1) H+_c0[c2] + (1) L-Glutamyl-tRNA-Glu_c0[c2] <-> (1) NADP_c0[c2] + (1) L-Glutamate1-semialdehyde_c0[c2] + (1) tRNA-Glu_c0[c2]	0.0022343	Yes
rxn00224	Porphyrin and chlorophyll metabolism	protoheme ferro-lyase (protoporphyrin-forming)	(1) Protoporphyrin_c0[c2] + (1) Fe2+_c0[c2] <-> (1) Heme_c0[c2] + (2) H+_c0[c2]	0.000139644	Yes
rxn02304	Porphyrin and chlorophyll metabolism	protoporphyrinogen-IX:oxygen oxidoreductase	(3) O2_c0[c2] + (2) ProtoporphyrinogenIX_c0[c2] -> (6) H2O_c0[c2] + (2) Protoporphyrin_c0[c2]	0.000069822	No
rxn06937	Porphyrin and chlorophyll metabolism|Aminoacyl-tRNA biosynthesis	L-glutamate:tRNA(Glu) ligase (AMP-forming)	(1) ATP_c0[c2] + (1) L-Glutamate_c0[c2] + (1) tRNA-Glu_c0[c2] -> (1) PPi_c0[c2] + (1) AMP_c0[c2] + (1) H+_c0[c2] + (1) L-Glutamyl-tRNA-Glu_c0[c2]	0.0022343	Yes
rxn00836	Purine metabolism	IMP:diphosphate phospho-D-ribosyltransferase	(1) PPi_c0[c2] + (1) H+_c0[c2] + (1) IMP_c0[c2] <- (1) PRPP_c0[c2] + (1) HYXN_c0[c2]	-0.0063446	Yes
rxn01297	Purine metabolism	hypoxanthine:NAD+ oxidoreductase	(1) H2O_c0[c2] + (1) NAD_c0[c2] + (1) HYXN_c0[c2] <-> (1) NADH_c0[c2] + (1) H+_c0[c2] + (1) XAN_c0[c2]	-0.0063446	Yes
rxn00097	Purine metabolism	ATP:AMP phosphotransferase	(1) ATP_c0[c2] + (1) AMP_c0[c2] + (1) H+_c0[c2] <-> (2) ADP_c0[c2]	0.0442194	Yes
rxn00131	Purine metabolism	AMP phosphoribohydrolase	(1) H2O_c0[c2] + (1) AMP_c0[c2] <-> (1) ribose-5-phosphate_c0[c2] + (1) Adenine_c0[c2]	-0.000977508	No
rxn05234	Purine metabolism	2'-Deoxyguanosine 5'-triphosphate:oxydized-thioredoxin 2'-oxidoreductase	(1) GTP_c0[c2] + (1) trdrd_c0[c2] -> (1) H2O_c0[c2] + (1) dGTP_c0[c2] + (1) trdox_c0[c2]	0.00090676	Yes
rxn00927	Purine metabolism	Adenosine ribohydrolase	(1) H2O_c0[c2] + (1) Adenosine_c0[c2] <-> (1) D-Ribose_c0[c2] + (1) Adenine_c0[c2]	0.000837864	Yes
rxn00915	Purine metabolism	GMP:diphosphate 5-phospho-alpha-D-ribosyltransferase	(1) PPi_c0[c2] + (1) H+_c0[c2] + (1) GMP_c0[c2] <- (1) PRPP_c0[c2] + (1) Guanine_c0[c2]	-0.00701331	Yes
rxn01225	Purine metabolism	Guanine aminohydrolase	(1) H2O_c0[c2] + (1) H+_c0[c2] + (1) Guanine_c0[c2] <-> (1) NH3_c0[c2] + (1) XAN_c0[c2]	-0.00701331	Yes
rxn00239	Purine metabolism	ATP:GMP phosphotransferase	(1) ATP_c0[c2] + (1) H+_c0[c2] + (1) GMP_c0[c2] <-> (1) ADP_c0[c2] + (1) GDP_c0[c2]	0.00715296	Yes
rxn05232	Purine metabolism	2'-Deoxyadenosine 5'-triphosphate:oxydized-thioredoxin 2'-oxidoreductase	(1) ATP_c0[c2] + (1) trdrd_c0[c2] -> (1) H2O_c0[c2] + (1) dATP_c0[c2] + (1) trdox_c0[c2]	0.000538045	Yes
rxn00800	Purine metabolism|Alanine, aspartate and glutamate metabolism	N6-(1,2-dicarboxyethyl)AMP AMP-lyase (fumarate-forming)	(1) Adenylosuccinate_c0[c2] <-> (1) AMP_c0[c2] + (1) Fumarate_c0[c2]	0.0063446	Yes
rxn00838	Purine metabolism|Alanine, aspartate and glutamate metabolism	IMP:L-aspartate ligase (GDP-forming)	(1) GTP_c0[c2] + (1) L-Aspartate_c0[c2] + (1) IMP_c0[c2] -> (1) Phosphate_c0[c2] + (1) GDP_c0[c2] + (2) H+_c0[c2] + (1) Adenylosuccinate_c0[c2]	0.0063446	Yes
rxn03004	Purine metabolism|One carbon pool by folate	10-Formyltetrahydrofolate:5'-	(1) 10-Formyltetrahydrofolate_c0[c2] + (1) GAR_c0[c2] <-> (1) H+_c0[c2] +	-0.000139644	Yes

		phosphoribosylglycinamide formyltransferase	(1) Tetrahydrofolate_c0[c2] + (1) N-Formyl-GAR_c0[c2]		
rxn00379	Purine metabolism|Sulfur metabolism	ATP:sulfate adenylyltransferase	(1) ATP_c0[c2] + (1) Sulfate_c0[c2] <-> (1) PPi_c0[c2] + (1) APS_c0[c2]	0.0136372	Yes
rxn00364	Pyrimidine metabolism	ATP:CMP phosphotransferase	(1) ATP_c0[c2] + (1) CMP_c0[c2] + (1) H+_c0[c2] <-> (1) ADP_c0[c2] + (1) CDP_c0[c2]	0.0249048	Yes
rxn01673	Pyrimidine metabolism	ATP:dCDP phosphotransferase	(1) ATP_c0[c2] + (1) dCDP_c0[c2] <-> (1) ADP_c0[c2] + (1) dCTP_c0[c2]	0.00090676	Yes
rxn00711	Pyrimidine metabolism	UMP:diphosphate phospho-alpha-D-ribosyltransferase	(1) PPi_c0[c2] + (1) H+_c0[c2] + (1) UMP_c0[c2] <- (1) Uracil_c0[c2] + (1) PRPP_c0[c2]	-0.00933402	Yes
rxn00412	Pyrimidine metabolism	UTP:L-glutamine amido-ligase (ADP-forming)	(1) H2O_c0[c2] + (1) ATP_c0[c2] + (1) L-Glutamine_c0[c2] + (1) UTP_c0[c2] -> (1) ADP_c0[c2] + (1) Phosphate_c0[c2] + (1) L-Glutamate_c0[c2] + (1) CTP_c0[c2] + (2) H+_c0[c2]	0.00469965	Yes
rxn05236	Pyrimidine metabolism	2'-Deoxyuridine 5'-triphosphate:oxydized-thioredoxin 2'-oxidoreductase	(1) UTP_c0[c2] + (1) trdrd_c0[c2] -> (1) H2O_c0[c2] + (1) dUTP_c0[c2] + (1) trdox_c0[c2]	0.000538045	Yes
rxn01512	Pyrimidine metabolism	ATP:dTDP phosphotransferase	(1) ATP_c0[c2] + (1) dTDP_c0[c2] <-> (1) ADP_c0[c2] + (1) TTP_c0[c2]	0.00166597	Yes
rxn06076	Pyrimidine metabolism	2'-Deoxycytidine diphosphate:oxidized-thioredoxin 2'-oxidoreductase	(1) H2O_c0[c2] + (1) dCDP_c0[c2] + (1) trdox_c0[c2] <- (1) CDP_c0[c2] + (1) trdrd_c0[c2]	-0.00090676	Yes
rxn00409	Pyrimidine metabolism	ATP:CDP phosphotransferase	(1) ATP_c0[c2] + (1) CDP_c0[c2] <-> (1) ADP_c0[c2] + (1) CTP_c0[c2]	0.0239981	Yes
rxn01519	Pyrimidine metabolism	dUTP nucleotidohydrolase	(1) H2O_c0[c2] + (1) dUTP_c0[c2] -> (1) PPi_c0[c2] + (2) H+_c0[c2] + (1) dUMP_c0[c2]	0.000538045	Yes
rxn01513	Pyrimidine metabolism	ATP:dTMP phosphotransferase	(1) ATP_c0[c2] + (1) H+_c0[c2] + (1) dTMP_c0[c2] <-> (1) ADP_c0[c2] + (1) dTDP_c0[c2]	0.000538045	Yes
rxn05289	Pyrimidine metabolism	NADPH:oxidized-thioredoxin oxidoreductase	(1) NADPH_c0[c2] + (1) H+_c0[c2] + (1) trdox_c0[c2] <-> (1) NADP_c0[c2] + (1) trdrd_c0[c2]	0.00288961	Yes
rxn00117	Pyrimidine metabolism	ATP:UDP phosphotransferase	(1) ATP_c0[c2] + (1) UDP_c0[c2] <-> (1) ADP_c0[c2] + (1) UTP_c0[c2]	0.00676754	Yes
rxn00654	Pyrimidine metabolism|beta-Alanine metabolism|Pantothenate and CoA biosynthesis	N-Carbamoyl-beta-alanine amidohydrolase	(1) H2O_c0[c2] + (2) H+_c0[c2] + (1) 3-Ureidopropanoate_c0[c2] -> (1) CO2_c0[c2] + (1) NH3_c0[c2] + (1) beta-Alanine_c0[c2]	0.354751	Yes
rxn01626	Pyrimidine metabolism|beta-Alanine metabolism|Pantothenate and CoA biosynthesis	5,6-Dihydrouracil amidohydrolase	(1) H2O_c0[c2] + (1) Hydrouracil_c0[c2] -> (1) H+_c0[c2] + (1) 3-Ureidopropanoate_c0[c2]	0.354751	Yes
rxn00720	Pyrimidine metabolism|beta-Alanine metabolism|Pantothenate and CoA biosynthesis	5,6-Dihydrouracil:NADP+ oxidoreductase	(1) NADP_c0[c2] + (1) Hydrouracil_c0[c2] <-> (1) NADPH_c0[c2] + (1) H+_c0[c2] + (1) Uracil_c0[c2]	-0.354751	Yes
rxn01520	Pyrimidine metabolism|One carbon pool by folate	5,10-Methylenetetrahydrofolate:dUMP C-methyltransferase	(1) 5-10-Methylenetetrahydrofolate_c0[c2] + (1) dUMP_c0[c2] -> (1) dTMP_c0[c2] + (1) Dihydrofolate_c0[c2]	0.000538045	Yes
rxn00145	Pyruvate metabolism	(S)-Lactate:ferricytochrome-c 2-oxidoreductase	(2) Cytochrome c3+_c0[c2] + (1) L-Lactate_c0[c2] <-> (1) Pyruvate_c0[c2] + (2) H+_c0[c2] + (2) Cytochrome c2+_c0[c2]	-2.87736	No

rxn00251	Pyruvate metabolism|Methane metabolism|Carbon fixation in photosynthetic organisms|Carbon fixation pathways in prokaryotes	phosphate:oxaloacetate carboxy-lyase (adding phosphate;phosphoenolpyruvate-forming)	(1) Phosphate_c0[c2] + (1) Oxaloacetate_c0[c2] + (1) H+_c0[c2] <-> (1) H2O_c0[c2] + (1) CO2_c0[c2] + (1) Phosphoenolpyruvate_c0[c2]	-0.391694	Yes
rxn00392	Riboflavin metabolism	ATP:riboflavin 5'-phosphotransferase	(1) ATP_c0[c2] + (1) Riboflavin_c0[c2] <-> (1) ADP_c0[c2] + (1) FMN_c0[c2] + (1) H+_c0[c2]	0.000139644	Yes
rxn00122	Riboflavin metabolism	ATP:FMN adenylyltransferase	(1) ATP_c0[c2] + (1) FMN_c0[c2] -> (1) PPi_c0[c2] + (1) FAD_c0[c2]	0.000139644	Yes
rxn00606	Starch and sucrose metabolism	alpha,alpha-Trehalose-6-phosphate phosphoglucohydrolase	(1) H2O_c0[c2] + (1) Trehalose 6-phosphate_c0[c2] <-> (1) D-Glucose_c0[c2] + (1) D-glucose-6-phosphate_c0[c2]	-0.074443	Yes
rxn00216	Streptomycin biosynthesis|Butirosin and neomycin biosynthesis	ATP:D-glucose 6-phosphotransferase	(1) ATP_c0[c2] + (1) D-Glucose_c0[c2] <-> (1) ADP_c0[c2] + (1) H+_c0[c2] + (1) D-glucose-6-phosphate_c0[c2]	-0.074443	Yes
rxn02000	Streptomycin biosynthesis|Polyketide sugar unit biosynthesis	dTDP-4-dehydro-6-deoxy-D-glucose 3,5-epimerase	(1) dTDP-4-oxo-6-deoxy-D-glucose_c0[c2] <-> (1) dTDP-4-oxo-L-rhamnose_c0[c2]	0.00112792	Yes
rxn01675	Streptomycin biosynthesis|Polyketide sugar unit biosynthesis	dTTP:alpha-D-glucose-1-phosphate thymidylyltransferase	(1) Glucose-1-phosphate_c0[c2] + (1) TTP_c0[c2] <-> (1) PPi_c0[c2] + (1) dTDPglucose_c0[c2]	0.00112792	Yes
rxn02003	Streptomycin biosynthesis|Polyketide sugar unit biosynthesis	dTDP-6-deoxy-L-mannose:NADP+ 4-oxidoreductase	(1) NADP_c0[c2] + (1) dTDP-rhamnose_c0[c2] <-> (1) NADPH_c0[c2] + (1) H+_c0[c2] + (1) dTDP-4-oxo-L-rhamnose_c0[c2]	-0.00112792	Yes
rxn01997	Streptomycin biosynthesis|Polyketide sugar unit biosynthesis|Biosynthesis of vancomycin group antibiotics	dTDPglucose 4,6-hydro-lyase	(1) dTDPglucose_c0[c2] -> (1) H2O_c0[c2] + (1) dTDP-4-oxo-6-deoxy-D-glucose_c0[c2]	0.00112792	Yes
rxn03958	Terpenoid backbone biosynthesis	1-Deoxy-D-xylulose-5-phosphate isomeroreductase	(1) NADP_c0[c2] + (1) 2-C-methyl-D-erythritol4-phosphate_c0[c2] <-> (1) NADPH_c0[c2] + (1) H+_c0[c2] + (1) 1-deoxy-D-xylulose5-phosphate_c0[c2]	-0.0157586	No
rxn03910	Terpenoid backbone biosynthesis	2-Phospho-4-(cytidine 5'-diphospho)-2-C-methyl-D-erythritol CMP-lyase (cyclizing)	(1) 2-phospho-4--cytidine5-diphospho-2-C-methyl-D-erythritol_c0[c2] <-> (1) CMP_c0[c2] + (1) 2-C-methyl-D-erythritol2-4-cyclodiphosphate_c0[c2]	0.0157586	Yes
rxn03908	Terpenoid backbone biosynthesis	ATP:4-(Cytidine 5'-diphospho)-2-C-methyl-D-erythritol 2-phosphotransferase	(1) ATP_c0[c2] + (1) 4--cytidine5-diphospho-2-C-methyl-D-erythritol_c0[c2] <-> (1) ADP_c0[c2] + (1) H+_c0[c2] + (1) 2-phospho-4--cytidine5-diphospho-2-C-methyl-D-erythritol_c0[c2]	0.0157586	Yes
rxn08352	Terpenoid backbone biosynthesis	R08210	(1) NADH_c0[c2] + (1) H+_c0[c2] + (1) 1-Hydroxy-2-methyl-2-butenyl 4-diphosphate_c0[c2] -> (1) H2O_c0[c2] + (1) NAD_c0[c2] + (1) DMAPP_c0[c2]	0.00154686	Yes
rxn08756	Terpenoid backbone biosynthesis	isopentenyl-diphosphate:NAD+ oxidoreductase	(1) NADH_c0[c2] + (1) H+_c0[c2] + (1) 1-Hydroxy-2-methyl-2-butenyl 4-diphosphate_c0[c2] -> (1) H2O_c0[c2] + (1) NAD_c0[c2] + (1) Isopentenyldiphosphate_c0[c2]	0.0142118	Yes
rxn03907	Terpenoid backbone biosynthesis	CTP: 2-C-Methyl-D-erythritol 4-phosphate cytidylyltransferase	(1) CTP_c0[c2] + (1) 2-C-methyl-D-erythritol4-phosphate_c0[c2] <-> (1) PPi_c0[c2] + (1) 4--cytidine5-diphospho-2-C-methyl-D-erythritol_c0[c2]	0.0157586	No

rxn01213	Terpenoid backbone biosynthesis	GPPSYN-RXN	(1) Isopentenyldiphosphate_c0[c2] + (1) DMAPP_c0[c2] -> (1) PPi_c0[c2] + (1) H+_c0[c2] + (1) Geranyldiphosphate_c0[c2]	0.00154686	Yes
rxn03909	Terpenoid backbone biosynthesis	1-Deoxy-D-xylulose-5-phosphate pyruvate-lyase (carboxylating)	(1) Pyruvate_c0[c2] + (1) H+_c0[c2] + (1) Glyceraldehyde3-phosphate_c0[c2] -> (1) CO2_c0[c2] + (1) 1-deoxy-D-xylulose5-phosphate_c0[c2]	0.0158983	Yes
rxn01466	Terpenoid backbone biosynthesis	Geranyl-diphosphate:isopentenyl-diphosphate geranyltrans-transferase	(1) Isopentenyldiphosphate_c0[c2] + (1) Geranyldiphosphate_c0[c2] -> (1) PPi_c0[c2] + (1) H+_c0[c2] + (1) Farnesyldiphosphate_c0[c2]	0.00154686	Yes
rxn13477	Terpenoid backbone biosynthesis	(2E,6E)-farnesyl-diphosphate:isopentenyl-diphosphate farnesyltranstransferase (adding 3 isopentenyl units)	(3) Isopentenyldiphosphate_c0[c2] + (1) Farnesyldiphosphate_c0[c2] -> (3) PPi_c0[c2] + (3) H+_c0[c2] + (1) all-trans-Hexaprenyl diphosphate[c2]	0.000418932	No
rxn00533	Tetracycline biosynthesis|Pyruvate metabolism|Propanoate metabolism|Carbon fixation pathways in prokaryotes	Acetyl-CoA:carbon-dioxide ligase (ADP-forming)	(1) ATP_c0[c2] + (1) Acetyl-CoA_c0[c2] + (1) H2CO3_c0[c2] <-> (1) ADP_c0[c2] + (1) Phosphate_c0[c2] + (1) H+_c0[c2] + (1) Malonyl-CoA_c0[c2]	0.470844	No
rxn00438	Thiamine metabolism	ATP:thiamin-phosphate phosphotransferase	(1) ATP_c0[c2] + (1) H+_c0[c2] + (1) Thiamine phosphate_c0[c2] <-> (1) ADP_c0[c2] + (1) TPP_c0[c2]	0.000139644	No
rxn11946	Ubiquinone and other terpenoid-quinone biosynthesis	R05614	(1) S-Adenosyl-L-methionine_c0[c2] + (1) 2-Octaprenyl-3-methyl-5-hydroxy-6-methoxy-1,4-benzoquinone_c0[c2] <-> (1) S-Adenosyl-homocysteine_c0[c2] + (1) H+_c0[c2] + (1) Ubiquinone-8_c0[c2]	0.000139644	Yes
rxn00966	Ubiquinone and other terpenoid-quinone biosynthesis	chorismate pyruvate-lyase (4-hydroxybenzoate-forming)	(1) Pyruvate_c0[c2] + (1) 4-Hydroxybenzoate_c0[c2] <- (1) Chorismate_c0[c2]	-0.000139644	No
rxn04139	Ubiquinone and other terpenoid-quinone biosynthesis	2-Octaprenyl-3-methyl-6-methoxy-1,4-benzoquinone ,NADPH2:oxygen oxidoreductase	(1) NADPH_c0[c2] + (1) O2_c0[c2] + (1) H+_c0[c2] + (1) 2-Octaprenyl-3-methyl-6-methoxy-1,4-benzoquinone_c0[c2] -> (1) H2O_c0[c2] + (1) NADP_c0[c2] + (1) 2-Octaprenyl-3-methyl-5-hydroxy-6-methoxy-1,4-benzoquinone_c0[c2]	0.000139644	Yes
rxn03893	Ubiquinone and other terpenoid-quinone biosynthesis	all-trans-octaprenyl-diphosphate:4-hydroxybenzoate 3-octaprenyltransferase	(1) 4-Hydroxybenzoate_c0[c2] + (1) Farnesylfarnesylgeraniol_c0[c2] -> (1) PPi_c0[c2] + (1) H+_c0[c2] + (1) 3-Octaprenyl-4-hydroxybenzoate_c0[c2]	0.000139644	Yes
rxn03393	Ubiquinone and other terpenoid-quinone biosynthesis	3-octaprenyl-4-hydroxybenzoate carboxy-lyase	(1) H+_c0[c2] + (1) 3-Octaprenyl-4-hydroxybenzoate_c0[c2] -> (1) CO2_c0[c2] + (1) 2-Octaprenylphenol_c0[c2]	0.000139644	Yes
rxn03394	Ubiquinone and other terpenoid-quinone biosynthesis	R04987	(1) NADPH_c0[c2] + (1) O2_c0[c2] + (1) H+_c0[c2] + (1) 2-Octaprenylphenol_c0[c2] -> (1) H2O_c0[c2] + (1) NADP_c0[c2] + (1) 2-Octaprenyl-6-hydroxyphenol_c0[c2]	0.000139644	Yes
rxn03395	Ubiquinone and other terpenoid-quinone biosynthesis	S-adenosyl-L-methionine:3-(all-trans-octaprenyl)benzene-1,2-diol 2-O-methyltransferase	(1) S-Adenosyl-L-methionine_c0[c2] + (1) 2-Octaprenyl-6-hydroxyphenol_c0[c2] <-> (1) S-Adenosyl-homocysteine_c0[c2] + (1) H+_c0[c2] + (1) 2-Octaprenyl-6-methoxyphenol_c0[c2]	0.000139644	Yes

rxn02831	Ubiquinone and other terpenoid-quinone biosynthesis	O-Succinylbenzoate:CoA ligase (AMP-forming)	(1) ATP_c0[c2] + (1) CoA_c0[c2] + (1) Succinylbenzoate[c2] -> (1) PPi_c0[c2] + (1) AMP_c0[c2] + (1) H+_c0[c2] + (1) Succinylbenzoyl-CoA[c2]	0.000279288	No
rxn02832	Ubiquinone and other terpenoid-quinone biosynthesis	(1R,6R)-6-hydroxy-2-succinylcyclohexa-2,4-diene-1-carboxylate hydrolyase (2-succinylbenzoate-forming)	(1) H2O_c0[c2] + (1) Succinylbenzoate[c2] <- (1) SHCHC[c2]	-0.000279288	No
rxn03397	Ubiquinone and other terpenoid-quinone biosynthesis	UDP-L-rhamnose:flavonol-3-O-D-glucoside L-rhamnosyltransferase	(1) S-Adenosyl-L-methionine_c0[c2] + (1) 2-Octaprenyl-6-methoxy-1,4-benzoquinone_c0[c2] -> (1) S-Adenosyl-homocysteine_c0[c2] + (1) H+_c0[c2] + (1) 2-Octaprenyl-3-methyl-6-methoxy-1,4-benzoquinone_c0[c2]	0.000139644	No
rxn01258	Ubiquinone and other terpenoid-quinone biosynthesis|Biosynthesis of siderophore group nonribosomal peptides	chorismate hydroxymutase	(1) Chorismate_c0[c2] -> (1) Isochorismate_c0[c2]	0.000279288	No
rxn03436	Valine, leucine and isoleucine biosynthesis	(S)-2-Aceto-2-hydroxybutanoate:NADP+ oxidoreductase (isomerizing)	(1) 2-Aceto-2-hydroxybutanoate_c0[c2] <-> (1) (R)-3-Hydroxy-3-methyl-2-oxopentanoate_c0[c2]	0.00385297	Yes
rxn03435	Valine, leucine and isoleucine biosynthesis	(R)-2,3-Dihydroxy-3-methylpentanoate:NADP+ oxidoreductase (isomerizing)	(1) NADP_c0[c2] + (1) 2,3-Dihydroxy-3-methylvalerate_c0[c2] <-> (1) NADPH_c0[c2] + (1) H+_c0[c2] + (1) (R)-3-Hydroxy-3-methyl-2-oxopentanoate_c0[c2]	-0.00385297	Yes
rxn00904	Valine, leucine and isoleucine biosynthesis	L-Valine:pyruvate aminotransferase	(1) Pyruvate_c0[c2] + (1) L-Valine_c0[c2] <-> (1) L-Alanine_c0[c2] + (1) 3-Methyl-2-oxobutanoate_c0[c2]	-0.331789	Yes
rxn01573	Valine, leucine and isoleucine biosynthesis	L-Isoleucine:NAD+ oxidoreductase(deaminating)	(1) H2O_c0[c2] + (1) NAD_c0[c2] + (1) L-Isoleucine_c0[c2] <-> (1) NADH_c0[c2] + (1) NH3_c0[c2] + (1) H+_c0[c2] + (1) 3MOP_c0[c2]	-1.13446E-05	Yes
rxn03437	Valine, leucine and isoleucine biosynthesis	(R)-2,3-Dihydroxy-3-methylpentanoate hydro-lyase	(1) 2,3-Dihydroxy-3-methylvalerate_c0[c2] -> (1) H2O_c0[c2] + (1) 3MOP_c0[c2]	0.00385297	Yes
rxn03194	Valine, leucine and isoleucine biosynthesis	(S)-2-Aceto-2-hydroxybutanoate pyruvate-lyase (carboxylating)	(1) 2-Oxobutyrate_c0[c2] + (1) 2-Hydroxyethyl-ThPP_c0[c2] <-> (1) TPP_c0[c2] + (1) 2-Aceto-2-hydroxybutanoate_c0[c2]	0.00385297	Yes
rxn07434	Valine, leucine and isoleucine degradation	R07603	(1) TPP_c0[c2] + (1) H+_c0[c2] + (1) 3MOP_c0[c2] -> (1) CO2_c0[c2] + (1) 2-Methyl-1-hydroxybutyl-TPP_c0[c2]	0.00384163	Yes
rxn07433	Valine, leucine and isoleucine degradation	R07602	(1) Lipoamide_c0[c2] + (1) 3-Methyl-1-hydroxybutyl-TPP_c0[c2] <-> (1) TPP_c0[c2] + (1) S-(3-Methylbutanoyl)-dihydrolipoamide-E_c0[c2]	0.00384163	Yes
rxn07435	Valine, leucine and isoleucine degradation	R07604	(1) Lipoamide_c0[c2] + (1) 2-Methyl-1-hydroxybutyl-TPP_c0[c2] <-> (1) TPP_c0[c2] + (1) S-(2-Methylbutanoyl)-dihydrolipoamide-E_c0[c2]	0.00384163	Yes
rxn06586	Valine, leucine and isoleucine degradation	3-methylbutanoyl-CoA:enzyme N6-(dihydrolipoyl)lysine S-(3-methylbutanoyl)transferase	(1) Dihydrolipoamide_c0[c2] + (1) Isovaleryl-CoA_c0[c2] <-> (1) CoA_c0[c2] + (1) S-(3-Methylbutanoyl)-dihydrolipoamide-E_c0[c2]	-0.00384163	Yes

rxn07432	Valine, leucine and isoleucine degradation	R07601	(1) TPP_c0[c2] + (1) H+_c0[c2] + (1) 4MOP_c0[c2] -> (1) CO2_c0[c2] + (1) 3-Methyl-1-hydroxybutyl-TPP_c0[c2]	0.00384163	Yes
rxn06335	Valine, leucine and isoleucine degradation	(S)-2-methylbutanoyl-CoA:enzyme N6-(dihydrolipoyl)lysine S-(2-methylbutanoyl)transferase	(1) Dihydrolipoamide_c0[c2] + (1) 2-Methylbutyryl-CoA_c0[c2] <-> (1) CoA_c0[c2] + (1) S-(2-Methylbutanoyl)-dihydrolipoamide-E_c0[c2]	-0.00384163	Yes
rxn00806	Valine, leucine and isoleucine degradation|Valine, leucine and isoleucine biosynthesis|Glucosinolate biosynthesis	L-Leucine:2-oxoglutarate aminotransferase	(1) 2-Oxoglutarate_c0[c2] + (1) L-Leucine_c0[c2] <-> (1) L-Glutamate_c0[c2] + (1) 4MOP_c0[c2]	0.00384163	Yes
rxn00903	Valine, leucine and isoleucine degradation|Valine, leucine and isoleucine biosynthesis|Pantothenate and CoA biosynthesis|Glucosinolate biosynthesis	L-Valine:2-oxoglutarate aminotransferase	(1) 2-Oxoglutarate_c0[c2] + (1) L-Valine_c0[c2] <-> (1) L-Glutamate_c0[c2] + (1) 3-Methyl-2-oxobutanoate_c0[c2]	0.332068	Yes
rxn01331	Vitamin B6 metabolism	D-erythrose 4-phosphate:NAD+ oxidoreductase	(1) H2O_c0[c2] + (1) NAD_c0[c2] + (1) D-Erythrose4-phosphate_c0[c2] -> (1) NADH_c0[c2] + (2) H+_c0[c2] + (1) 4-Phosphoerythronate_c0[c2]	0.000139644	Yes
rxn02939	Vitamin B6 metabolism	4-phospho-D-erythronate:NAD+ 2-oxidoreductase	(1) NAD_c0[c2] + (1) 4-Phosphoerythronate_c0[c2] <-> (1) NADH_c0[c2] + (1) H+_c0[c2] + (1) 2-Oxo-3-hydroxy-4-phosphobutanoate_c0[c2]	0.000139644	Yes
rxn00209	Vitamin B6 metabolism	Pyridoxine 5-phosphate:oxygen oxidoreductase	(1) O2_c0[c2] + (1) Pyridoxine phosphate_c0[c2] -> (1) Pyridoxal phosphate_c0[c2] + (1) H2O2_c0[c2]	0.000139644	Yes
rxn03445	Vitamin B6 metabolism	O-Phospho-4-hydroxy-L-threonine:2-oxoglutarate aminotransferase	(1) 2-Oxoglutarate_c0[c2] + (1) 4-(Phosphonooxy)-threonine_c0[c2] <-> (1) L-Glutamate_c0[c2] + (1) 2-Oxo-3-hydroxy-4-phosphobutanoate_c0[c2]	-0.000139644	Yes

***Sphingobium* sp. AP49, community in R2A medium**

rxn00555	Alanine, aspartate and glutamate metabolism|Amino sugar and nucleotide sugar metabolism	L-glutamine:D-fructose-6-phosphate isomerase (deaminating)	(1) L-Glutamine_c0[c1] + (1) D-fructose-6-phosphate_c0[c1] <-> (1) L-Glutamate_c0[c1] + (1) D-Glucosamine phosphate_c0[c1]	-0.0534271	No
rxn00802	Alanine, aspartate and glutamate metabolism|Arginine and proline metabolism	2-(Nomega-L-arginino)succinate arginine-lyase (fumarate-forming)	(1) L-Argininosuccinate_c0[c1] <-> (1) L-Arginine_c0[c1] + (1) Fumarate_c0[c1]	0.00476807	No
rxn00503	Alanine, aspartate and glutamate metabolism|Arginine and proline metabolism	(S)-1-pyrroline-5-carboxylate:NAD+ oxidoreductase	(2) H2O_c0[c1] + (1) NAD_c0[c1] + (1) 1-Pyrroline-5-carboxylate_c0[c1] <-> (1) NADH_c0[c1] + (1) L-Glutamate_c0[c1] + (1) H+_c0[c1]	-0.00356314	No
rxn01434	Alanine, aspartate and glutamate metabolism|Arginine and proline metabolism	L-Citrulline:L-aspartate ligase (AMP-forming)	(1) ATP_c0[c1] + (1) L-Aspartate_c0[c1] + (1) Citrulline_c0[c1] <-> (1) PPi_c0[c1] + (1) AMP_c0[c1] + (2) H+_c0[c1] + (1) L-Argininosuccinate_c0[c1]	0.00476807	Yes
rxn00182	Alanine, aspartate and glutamate metabolism|Arginine and proline metabolism|Taurine and hypotaurine metabolism|D-Glutamine and D-glutamate	L-glutamate:NAD+ oxidoreductase (deaminating)	(1) H2O_c0[c1] + (1) NAD_c0[c1] + (1) L-Glutamate_c0[c1] <-> (1) NADH_c0[c1] + (1) NH3_c0[c1] + (1) 2-Oxoglutarate_c0[c1] + (1) H+_c0[c1]	-0.298593	Yes

	metabolism|Nitrogen metabolism				
rxn00260	Alanine, aspartate and glutamate metabolism|Carbon fixation in photosynthetic organisms	L-Aspartate:2-oxoglutarate aminotransferase	(1) 2-Oxoglutarate_c0[c1] + (1) L-Aspartate_c0[c1] <-> (1) L-Glutamate_c0[c1] + (1) Oxaloacetate_c0[c1]	-0.166214	No
rxn00085	Alanine, aspartate and glutamate metabolism|Nitrogen metabolism	L-Glutamate:NADP+ oxidoreductase (transaminating)	(1) NADP_c0[c1] + (2) L-Glutamate_c0[c1] <-> (1) NADPH_c0[c1] + (1) 2-Oxoglutarate_c0[c1] + (1) L-Glutamine_c0[c1] + (1) H+_c0[c1]	0.104599	No
rxn00278	Alanine, aspartate and glutamate metabolism|Taurine and hypotaurine metabolism	L-Alanine:NAD+ oxidoreductase (deaminating)	(1) H2O_c0[c1] + (1) NAD_c0[c1] + (1) L-Alanine_c0[c1] <-> (1) NADH_c0[c1] + (1) NH3_c0[c1] + (1) Pyruvate_c0[c1] + (1) H+_c0[c1]	-0.00972115	Yes
rxn01484	Amino sugar and nucleotide sugar metabolism	N-Acetyl-D-glucosamine-6-phosphate amidohydrolase	(1) H2O_c0[c1] + (1) N-Acetyl-D-glucosamine 6-phosphate_c0[c1] <-> (1) Acetate_c0[c1] + (1) D-Glucosamine phosphate_c0[c1]	0.0553607	No
rxn03638	Amino sugar and nucleotide sugar metabolism	Acetyl-CoA:D-glucosamine-1-phosphate N-acetyltransferase	(1) Acetyl-CoA_c0[c1] + (1) D-Glucosamine1-phosphate_c0[c1] -> (1) CoA_c0[c1] + (1) H+_c0[c1] + (1) N-Acetyl-D-glucosamine1-phosphate_c0[c1]	0.00193358	Yes
rxn01485	Amino sugar and nucleotide sugar metabolism	D-Glucosamine 1-phosphate 1,6-phosphomutase	(1) D-Glucosamine1-phosphate_c0[c1] <-> (1) D-Glucosamine phosphate_c0[c1]	-0.00193358	Yes
rxn00293	Amino sugar and nucleotide sugar metabolism	UTP:N-acetyl-alpha-D-glucosamine-1-phosphate uridylyltransferase	(1) UTP_c0[c1] + (1) N-Acetyl-D-glucosamine1-phosphate_c0[c1] <-> (1) PPi_c0[c1] + (1) UDP-N-acetylglucosamine_c0[c1]	-0.143369	Yes
rxn02285	Amino sugar and nucleotide sugar metabolism|Peptidoglycan biosynthesis	UDP-N-acetylmuramate:NADP+ oxidoreductase	(1) NADP_c0[c1] + (1) UDP-MurNAc_c0[c1] <-> (1) NADPH_c0[c1] + (1) H+_c0[c1] + (1) UDP-N-acetylglucosamine enolpyruvate_c0[c1]	-0.000483396	No
rxn00461	Amino sugar and nucleotide sugar metabolism|Peptidoglycan biosynthesis	Phosphoenolpyruvate:UDP-N-acetyl-D-glucosamine 1-carboxyvinyl-transferase	(1) UDP-N-acetylglucosamine_c0[c1] + (1) Phosphoenolpyruvate_c0[c1] <-> (1) Phosphate_c0[c1] + (1) UDP-N-acetylglucosamine enolpyruvate_c0[c1]	0.000483396	Yes
rxn01636	Arginine and proline metabolism	N2-Acetyl-L-ornithine:L-glutamate N-acetyltransferase	(1) L-Glutamate_c0[c1] + (1) N-Acetylornithine_c0[c1] <-> (1) Ornithine_c0[c1] + (1) N-Acetyl-L-glutamate_c0[c1]	0.00488776	No
rxn01637	Arginine and proline metabolism	N2-Acetyl-L-ornithine:2-oxoglutarate aminotransferase	(1) 2-Oxoglutarate_c0[c1] + (1) N-Acetylornithine_c0[c1] <-> (1) L-Glutamate_c0[c1] + (1) 2-Acetamido-5-oxopentanoate_c0[c1]	-0.00488776	Yes
rxn01917	Arginine and proline metabolism	ATP:N-acetyl-L-glutamate 5-phosphotransferase	(1) ATP_c0[c1] + (1) N-Acetyl-L-glutamate_c0[c1] <-> (1) ADP_c0[c1] + (1) n-acetylglutamyl-phosphate_c0[c1]	0.00488776	Yes
rxn00931	Arginine and proline metabolism	L-Proline:NADP+ 5-oxidoreductase	(1) NADP_c0[c1] + (1) L-Proline_c0[c1] <-> (1) NADPH_c0[c1] + (2) H+_c0[c1] + (1) 1-Pyrroline-5-carboxylate_c0[c1]	-0.00356314	No
rxn02465	Arginine and proline metabolism	N-acetyl-L-glutamate-5-semialdehyde:NADP+ 5-oxidoreductase (phosphrylating)	(1) NADP_c0[c1] + (1) Phosphate_c0[c1] + (1) 2-Acetamido-5-oxopentanoate_c0[c1] <- (1) NADPH_c0[c1] + (1) H+_c0[c1] + (1) n-acetylglutamyl-phosphate_c0[c1]	-0.00488776	No

rxn01019	Arginine and proline metabolism	Carbamoyl-phosphate:L-ornithine carbamoyltransferase	(1) Ornithine_c0[c1] + (1) Carbamoylphosphate_c0[c1] -> (1) Phosphate_c0[c1] + (1) H+_c0[c1] + (1) Citrulline_c0[c1]	0.00476807	No
rxn01791	beta-Alanine metabolism|Pantothenate and CoA biosynthesis	(R)-Pantoate:beta-alanine ligase (AMP-forming)	(1) ATP_c0[c1] + (1) beta-Alanine_c0[c1] + (1) Pantoate_c0[c1] -> (1) PPi_c0[c1] + (1) AMP_c0[c1] + (2) H+_c0[c1] + (1) PAN_c0[c1]	0.000119695	No
rxn02185	Butanoate metabolism	2-Acetolactate pyruvate-lyase (carboxylating)	(1) TPP_c0[c1] + (1) ALCTT_c0[c1] <-> (1) Pyruvate_c0[c1] + (1) 2-Hydroxyethyl-ThPP_c0[c1]	-0.00692753	No
rxn00785	Carbon fixation in photosynthetic organisms	D-Fructose 6-phosphate:D-glyceraldehyde-3-phosphate glycolaldehyde transferase	(1) D-fructose-6-phosphate_c0[c1] + (1) Glyceraldehyde3-phosphate_c0[c1] <-> (1) D-Xylulose5-phosphate_c0[c1] + (1) D-Erythrose4-phosphate_c0[c1]	0.0537017	Yes
rxn00285	Citrate cycle (TCA cycle)|Propanoate metabolism|Carbon fixation pathways in prokaryotes	Succinate:CoA ligase (ADP-forming)	(1) ATP_c0[c1] + (1) CoA_c0[c1] + (1) Succinate_c0[c1] <-> (1) ADP_c0[c1] + (1) Phosphate_c0[c1] + (1) Succinyl-CoA_c0[c1]	0.0187199	Yes
rxn00126	Cysteine and methionine metabolism	ATP:L-methionine S-adenosyltransferase	(1) H2O_c0[c1] + (1) ATP_c0[c1] + (1) L-Methionine_c0[c1] -> (1) Phosphate_c0[c1] + (1) PPi_c0[c1] + (1) S-Adenosyl-L-methionine_c0[c1] + (1) H+_c0[c1]	0.000418932	Yes
rxn00952	Cysteine and methionine metabolism	O-acetyl-L-homoserine:hydrogen sulfide S-(3-amino-3-carboxypropyl)transferase	(1) H2S_c0[c1] + (1) O-Acetyl-L-homoserine_c0[c1] -> (1) Acetate_c0[c1] + (1) Homocysteine_c0[c1]	0.00252995	No
rxn00141	Cysteine and methionine metabolism	S-Adenosyl-L-homocysteine hydrolase	(1) H2O_c0[c1] + (1) S-Adenosyl-homocysteine_c0[c1] <-> (1) Homocysteine_c0[c1] + (1) Adenosine_c0[c1]	0.000359084	Yes
rxn01406	Cysteine and methionine metabolism|Arginine and proline metabolism|Glutathione metabolism	S-adenosylmethioninamine:putrescine 3-aminopropyltransferase	(1) Putrescine_c0[c1] + (1) S-Adenosylmethioninamine_c0[c1] -> (1) H+_c0[c1] + (1) 5-Methylthioadenosine_c0[c1] + (1) Spermidine_c0[c1]	5.98474E-05	No
rxn00693	Cysteine and methionine metabolism|One carbon pool by folate	5-Methyltetrahydrofolate:L-homocysteine S-methyltransferase	(1) Homocysteine_c0[c1] + (1) 5-Methyltetrahydrofolate_c0[c1] <-> (1) L-Methionine_c0[c1] + (1) Tetrahydrofolate_c0[c1]	0.00288903	No
rxn00423	Cysteine and methionine metabolism|Sulfur metabolism	acetyl-CoA:L-serine O-acetyltransferase	(1) Acetyl-CoA_c0[c1] + (1) L-Serine_c0[c1] -> (1) CoA_c0[c1] + (1) O-Acetyl-L-serine_c0[c1]	0.00165127	No
rxn00649	Cysteine and methionine metabolism|Sulfur metabolism	O3-acetyl-L-serine:hydrogen-sulfide 2-amino-2-carboxyethyltransferase	(1) H2S_c0[c1] + (1) O-Acetyl-L-serine_c0[c1] -> (1) Acetate_c0[c1] + (1) L-Cysteine_c0[c1]	0.00165127	No
rxn01303	Cysteine and methionine metabolism|Sulfur metabolism	Acetyl-CoA:L-homoserine O-acetyltransferase	(1) Acetyl-CoA_c0[c1] + (1) L-Homoserine_c0[c1] -> (1) CoA_c0[c1] + (1) O-Acetyl-L-homoserine_c0[c1]	0.00252995	No
rxn00283	D-Alanine metabolism	alanine racemase	(1) L-Alanine_c0[c1] <-> (1) D-Alanine_c0[c1]	0.000966792	No
rxn00193	D-Glutamine and D-glutamate metabolism	glutamate racemase	(1) L-Glutamate_c0[c1] <-> (1) D-Glutamate_c0[c1]	0.000483396	No
rxn02286	D-Glutamine and D-glutamate metabolism|Peptidoglycan biosynthesis	UDP-N-acetylmuramate:L-alanine ligase (ADP-forming)	(1) ATP_c0[c1] + (1) L-Alanine_c0[c1] + (1) UDP-MurNAc_c0[c1] -> (1) ADP_c0[c1] + (1) Phosphate_c0[c1] + (1) H+_c0[c1] + (1) UDP-N-acetylmuramoyl-L-alanine_c0[c1]	0.000483396	Yes

rxn02008	D-Glutamine and D-glutamate metabolism|Peptidoglycan biosynthesis	UDP-N-acetylmuramoyl-L-alanine:D-glutamate ligase(ADP-forming)	(1) ATP_c0[c1] + (1) D-Glutamate_c0[c1] + (1) UDP-N-acetylmuramoyl-L-alanine_c0[c1] -> (1) ADP_c0[c1] + (1) Phosphate_c0[c1] + (1) H+_c0[c1] + (1) UDP-N-acetylmuramoyl-L-alanyl-D-glutamate_c0[c1]	0.000483396	No
rxn05345	Fatty acid biosynthesis	dodecanoyl-[acyl-carrier-protein]:malonyl-[acyl-carrier-protein] C-acyltransferase (decarboxylating)	(1) Dodecanoyl-ACP_c0[c1] + (1) Malonyl-acyl-carrierprotein-_c0[c1] -> (1) CO2_c0[c1] + (1) 3-oxotetradecanoyl-acp_c0[c1] + (1) ACP_c0[c1]	0.00193358	Yes
rxn05465	Fatty acid biosynthesis	Malonyl-CoA:[acyl-carrier-protein] S-malonyltransferase	(1) H+_c0[c1] + (1) Malonyl-CoA_c0[c1] + (1) ACP_c0[c1] <-> (1) CoA_c0[c1] + (1) Malonyl-acyl-carrierprotein-_c0[c1]	0.0192736	No
rxn05342	Fatty acid biosynthesis	(3R)-3-Hydroxytetradecanoyl-[acyl-carrier-protein]:NADP+ oxidoreductase	(1) NADP_c0[c1] + (1) HMA_c0[c1] <-> (1) NADPH_c0[c1] + (1) 3-oxotetradecanoyl-acp_c0[c1]	-0.00193358	Yes
rxn02504	Folate biosynthesis	2-amino-4-hydroxy-6-(D-erythro-1,2,3-trihydroxypropyl)-7,8-dihydropteridine glycolaldehyde-lyase	(1) Dihydroneopterin_c0[c1] <-> (1) Glycolaldehyde_c0[c1] + (1) 6-hydroxymethyl dihydropterin_c0[c1]	0.000179542	No
rxn03167	Folate biosynthesis	2-Amino-4-hydroxy-6-(erythro-1,2,3-trihydroxypropyl) dihydropteridine triphosphate phosphohydrolase (alkaline optimum)	(3) H2O_c0[c1] + (1) 7,8-Dihydroneopterin 3'-triphosphate_c0[c1] -> (3) Phosphate_c0[c1] + (3) H+_c0[c1] + (1) Dihydroneopterin_c0[c1]	0.000179542	No
rxn02200	Folate biosynthesis	2-amino-4-hydroxy-6-hydroxymethyl-7,8-dihydropteridine:4-aminobenzoate 2-amino-4-hydroxydihydropteridine-6-methenyltransferase	(1) ABEE_c0[c1] + (1) 6-hydroxymethyl dihydropterin_c0[c1] <-> (1) H2O_c0[c1] + (1) Dihydropteroate_c0[c1]	0.000179542	No
rxn01314	Folate biosynthesis	5,6,7,8-tetrahydrobiopterin:NADP+ oxidoreductase	(1) NADPH_c0[c1] + (1) H+_c0[c1] + (1) Dihydrobiopterin_c0[c1] <-> (1) NADP_c0[c1] + (1) Tetrahydrobiopterin_c0[c1]	0.00233239	No
rxn03841	Folate biosynthesis	4-amino-4-deoxychorismate pyruvate-lyase	(1) ADC_c0[c1] -> (1) Pyruvate_c0[c1] + (1) H+_c0[c1] + (1) ABEE_c0[c1]	0.000179542	No
rxn01257	Folate biosynthesis	chorismate:L-glutamine aminotransferase	(1) L-Glutamine_c0[c1] + (1) Chorismate_c0[c1] <-> (1) L-Glutamate_c0[c1] + (1) ADC_c0[c1]	0.000179542	No
rxn01603	Folate biosynthesis	7,8-dihydropteroate:L-glutamate ligase (ADP-forming)	(1) ATP_c0[c1] + (1) L-Glutamate_c0[c1] + (1) Dihydropteroate_c0[c1] -> (1) ADP_c0[c1] + (1) Phosphate_c0[c1] + (1) H+_c0[c1] + (1) Dihydrofolate_c0[c1]	0.000179542	No
rxn00650	Glutathione metabolism	L-cysteinylglycine dipeptidase	(1) H2O_c0[c1] + (1) Cys-Gly_c0[c1] <-> (1) Glycine_c0[c1] + (1) L-Cysteine_c0[c1]	-5.98474E-05	Yes
rxn00350	Glutathione metabolism	glutathione gamma-glutamylaminopeptidase	(1) H2O_c0[c1] + (1) GSH_c0[c1] <-> (1) L-Glutamate_c0[c1] + (1) Cys-Gly_c0[c1]	-5.98474E-05	No

rxn00615	Glycerolipid metabolism	ATP:glycerol 3-phosphotransferase	(1) ATP_c0[c1] + (1) Glycerol_c0[c1] <-> (1) ADP_c0[c1] + (1) H+_c0[c1] + (1) Glycerol-3-phosphate_c0[c1]	0.000617405	No
rxn00612	Glycerophospholipid metabolism	sn-Glycerol-3-phosphate:NADP+ 2-oxidoreductase	(1) NADP_c0[c1] + (1) Glycerol-3-phosphate_c0[c1] <-> (1) NADPH_c0[c1] + (1) H+_c0[c1] + (1) Glycerone-phosphate_c0[c1]	-0.00370443	No
rxn06493	Glycine, serine and threonine metabolism	dihydrolipoylprotein:NAD+ oxidoreductase	(1) NAD_c0[c1] + (1) Dihydrolipolprotein_c0[c1] <-> (1) NADH_c0[c1] + (1) H+_c0[c1] + (1) Lipoylprotein_c0[c1]	0.00594534	Yes
rxn01300	Glycine, serine and threonine metabolism	ATP:L-homoserine O-phosphotransferase	(1) ATP_c0[c1] + (1) L-Homoserine_c0[c1] <-> (1) ADP_c0[c1] + (1) H+_c0[c1] + (1) O-Phospho-L-homoserine_c0[c1]	0.0103993	No
rxn01069	Glycine, serine and threonine metabolism	O-phospho-L-homoserine phosphate-lyase (adding water;L-threonine-forming)	(1) H2O_c0[c1] + (1) O-Phospho-L-homoserine_c0[c1] -> (1) Phosphate_c0[c1] + (1) L-Threonine_c0[c1]	0.0103993	No
rxn06377	Glycine, serine and threonine metabolism	glycine:lipoylprotein oxidoreductase (decarboxylating and acceptor-aminomethylating)	(1) Glycine_c0[c1] + (1) H+_c0[c1] + (1) Lipoylprotein_c0[c1] -> (1) CO2_c0[c1] + (1) S-Aminomethyldihydrolipoylprotein_c0[c1]	0.00594534	Yes
rxn00692	Glycine, serine and threonine metabolism|Cyanoamino acid metabolism|Glyoxylate and dicarboxylate metabolism|One carbon pool by folate|Methane metabolism	5,10-Methylenetetrahydrofolate:glycine hydroxymethyltransferase	(1) H2O_c0[c1] + (1) Glycine_c0[c1] + (1) 5-10-Methylenetetrahydrofolate_c0[c1] <-> (1) L-Serine_c0[c1] + (1) Tetrahydrofolate_c0[c1]	0.00128494	No
rxn00337	Glycine, serine and threonine metabolism|Cysteine and methionine metabolism|Lysine biosynthesis	ATP:L-aspartate 4-phosphotransferase	(1) ATP_c0[c1] + (1) L-Aspartate_c0[c1] <-> (1) ADP_c0[c1] + (1) 4-Phospho-L-aspartate_c0[c1]	0.0129891	No
rxn01643	Glycine, serine and threonine metabolism|Cysteine and methionine metabolism|Lysine biosynthesis	L-Aspartate-4-semialdehyde:NADP+ oxidoreductase (phosphorylating)	(1) NADP_c0[c1] + (1) Phosphate_c0[c1] + (1) L-Aspartate4-semialdehyde_c0[c1] <- (1) NADPH_c0[c1] + (1) H+_c0[c1] + (1) 4-Phospho-L-aspartate_c0[c1]	-0.0129891	No
rxn01302	Glycine, serine and threonine metabolism|Cysteine and methionine metabolism|Lysine biosynthesis	L-homoserine:NADP+ oxidoreductase	(1) NADP_c0[c1] + (1) L-Homoserine_c0[c1] <-> (1) NADPH_c0[c1] + (1) H+_c0[c1] + (1) L-Aspartate4-semialdehyde_c0[c1]	-0.0129891	No
rxn00420	Glycine, serine and threonine metabolism|Methane metabolism	O-phospho-L-serine phosphohydrolase	(1) H2O_c0[c1] + (1) phosphoserine_c0[c1] -> (1) Phosphate_c0[c1] + (1) L-Serine_c0[c1]	0.00536451	Yes
rxn01101	Glycine, serine and threonine metabolism|Methane metabolism	3-Phospho-D-glycerate:NAD+ 2-oxidoreductase	(1) NAD_c0[c1] + (1) 3-Phosphoglycerate_c0[c1] <-> (1) NADH_c0[c1] + (1) H+_c0[c1] + (1) 3-Phosphonooxypyruvate_c0[c1]	0.00536451	Yes
rxn02914	Glycine, serine and threonine metabolism|Methane metabolism	3-Phosphoserine:2-oxoglutarate aminotransferase	(1) 2-Oxoglutarate_c0[c1] + (1) phosphoserine_c0[c1] <-> (1) L-Glutamate_c0[c1] + (1) 3-Phosphonooxypyruvate_c0[c1]	-0.00536451	Yes
rxn06600	Glycine, serine and threonine metabolism|One carbon pool by folate	S-aminomethyldihydrolipoylprotein:(6S)-tetrahydrofolate aminomethyltransferase (ammonia-forming)	(1) Tetrahydrofolate_c0[c1] + (1) S-Aminomethyldihydrolipoylprotein_c0[c1] -> (1) NH3_c0[c1] + (1) 5-10-Methylenetetrahydrofolate_c0[c1] + (1) Dihydrolipolprotein_c0[c1]	0.00594534	Yes

rxn01964	Glycine, serine and threonine metabolism|Phenylalanine, tyrosine and tryptophan biosynthesis	L-serine hydro-lyase [adding 1-C-(indol-3-yl)glycerol 3-phosphate; L-tryptophan and glyceraldehyde-3-phosphate-forming]	(1) L-Serine_c0[c1] + (1) Indoleglycerol phosphate_c0[c1] -> (1) H2O_c0[c1] + (1) L-Tryptophan_c0[c1] + (1) Glyceraldehyde3-phosphate_c0[c1]	0.000912302	No
rxn00599	Glycine, serine and threonine metabolism|Porphyrin and chlorophyll metabolism	succinyl-CoA:glycine C-succinyltransferase (decarboxylating)	(1) Glycine_c0[c1] + (1) H+_c0[c1] + (1) Succinyl-CoA_c0[c1] -> (1) CoA_c0[c1] + (1) CO2_c0[c1] + (1) 5-Aminolevulinate_c0[c1]	0.000957559	No
rxn00737	Glycine, serine and threonine metabolism|Valine, leucine and isoleucine biosynthesis	L-threonine ammonia-lyase (2-oxobutanoate-forming)	(1) L-Threonine_c0[c1] -> (1) NH3_c0[c1] + (1) 2-Oxobutyrate_c0[c1]	0.00631981	No
rxn00781	Glycolysis / Gluconeogenesis|Carbon fixation in photosynthetic organisms	D-glyceraldehyde-3-phosphate:NAD+ oxidoreductase (phosphorylating)	(1) NAD_c0[c1] + (1) Phosphate_c0[c1] + (1) Glyceraldehyde3-phosphate_c0[c1] <-> (1) NADH_c0[c1] + (1) H+_c0[c1] + (1) 1,3-Bisphospho-D-glycerate_c0[c1]	0.0749697	Yes
rxn01100	Glycolysis / Gluconeogenesis|Carbon fixation in photosynthetic organisms	ATP:3-phospho-D-glycerate 1-phosphotransferase	(1) ATP_c0[c1] + (1) 3-Phosphoglycerate_c0[c1] <-> (1) ADP_c0[c1] + (1) 1,3-Bisphospho-D-glycerate_c0[c1]	-0.0749697	Yes
rxn00011	Glycolysis / Gluconeogenesis|Citrate cycle (TCA cycle)|Valine, leucine and isoleucine biosynthesis|Pyruvate metabolism|Butanoate metabolism	pyruvate:thiamin diphosphate acetaldehydetransferase (decarboxylating)	(1) CO2_c0[c1] + (1) 2-Hydroxyethyl-ThPP_c0[c1] <- (1) Pyruvate_c0[c1] + (1) TPP_c0[c1] + (1) H+_c0[c1]	-0.0132473	Yes
rxn00747	Glycolysis / Gluconeogenesis|Fructose and mannose metabolism|Inositol phosphate metabolism|Carbon fixation in photosynthetic organisms	D-glyceraldehyde-3-phosphate aldose-ketose-isomerase	(1) Glyceraldehyde3-phosphate_c0[c1] <-> (1) Glycerone-phosphate_c0[c1]	0.00382412	No
rxn00704	Glycolysis / Gluconeogenesis|Galactose metabolism|Starch and sucrose metabolism|Amino sugar and nucleotide sugar metabolism	alpha-D-Glucose 1-phosphate 1,6-phosphomutase	(1) Glucose-1-phosphate_c0[c1] <-> (1) D-glucose-6-phosphate_c0[c1]	-0.000483396	No
rxn01106	Glycolysis / Gluconeogenesis|Glycine, serine and threonine metabolism|Methane metabolism	2-Phospho-D-glycerate 2,3-phosphomutase	(1) 2-Phospho-D-glycerate_c0[c1] <-> (1) 3-Phosphoglycerate_c0[c1]	-0.0696052	Yes
rxn00459	Glycolysis / Gluconeogenesis|Methane metabolism	2-phospho-D-glycerate hydro-lyase (phosphoenolpyruvate-forming)	(1) 2-Phospho-D-glycerate_c0[c1] <-> (1) H2O_c0[c1] + (1) Phosphoenolpyruvate_c0[c1]	0.0696052	Yes
rxn00175	Glycolysis / Gluconeogenesis|Methane metabolism|Carbon fixation pathways in prokaryotes	Acetate:CoA ligase (AMP-forming)	(1) ATP_c0[c1] + (1) CoA_c0[c1] + (1) Acetate_c0[c1] <-> (1) PPi_c0[c1] + (1) AMP_c0[c1] + (1) Acetyl-CoA_c0[c1] + (1) H+_c0[c1]	0.0605087	No
rxn00148	Glycolysis / Gluconeogenesis|Purine metabolism|Pyruvate metabolism|Carbon fixation in photosynthetic organisms	ATP:pyruvate 2-O-phosphotransferase	(1) ATP_c0[c1] + (1) Pyruvate_c0[c1] <-> (1) ADP_c0[c1] + (1) Phosphoenolpyruvate_c0[c1] + (1) H+_c0[c1]	0.458469	Yes
rxn00006	Glyoxylate and dicarboxylate metabolism	hydrogen-peroxide:hydrogen-peroxide oxidoreductase	(2) H2O2_c0[c1] -> (2) H2O_c0[c1] + (1) O2_c0[c1]	5.98474E-05	No

rxn00371	Glyoxylate and dicarboxylate metabolism|Methane metabolism	Formate:NAD+ oxidoreductase	(1) NAD_c0[c1] + (1) Formate_c0[c1] -> (1) NADH_c0[c1] + (1) CO2_c0[c1]	0.000897711	No
rxn02320	Histidine metabolism	5-Amino-2-oxopentanoate:2-oxoglutarate aminotransferase	(1) 2-Oxoglutarate_c0[c1] + (1) L-histidinol-phosphate_c0[c1] <-> (1) L-Glutamate_c0[c1] + (1) imidazole acetol-phosphate_c0[c1]	-0.00153198	No
rxn00789	Histidine metabolism	1-(5-phospho-D-ribosyl)-ATP:diphosphate phospho-alpha-D-ribosyl-transferase	(1) PPi_c0[c1] + (1) H+_c0[c1] + (1) Phosphoribosyl-ATP_c0[c1] <- (1) ATP_c0[c1] + (1) PRPP_c0[c1]	-0.00153198	No
rxn02160	Histidine metabolism	L-Histidinol-phosphate phosphohydrolase	(1) H2O_c0[c1] + (1) L-histidinol-phosphate_c0[c1] -> (1) Phosphate_c0[c1] + (1) L-Histidinol_c0[c1]	0.00153198	No
rxn02159	Histidine metabolism	L-Histidinol:NAD+ oxidoreductase	(1) NAD_c0[c1] + (1) L-Histidinol_c0[c1] <-> (1) NADH_c0[c1] + (1) H+_c0[c1] + (1) L-Histidinal_c0[c1]	0.00153198	No
rxn02835	Histidine metabolism	1-(5-phospho-D-ribosyl)-AMP 1,6-hydrolase	(1) H2O_c0[c1] + (1) Phosphoribosyl-AMP_c0[c1] <-> (1) phosphoribosylformiminoaicar-phosphate_c0[c1]	0.00153198	No
rxn03175	Histidine metabolism	N-(5'-Phospho-D-ribosylformimino)-5-amino-1- (5"-phospho-D-ribosyl)-4-imidazolecarboxamide ketol-isomerase	(1) H+_c0[c1] + (1) phosphoribosylformiminoaicar-phosphate_c0[c1] <-> (1) phosphoribulosylformimino-AICAR-phosphate_c0[c1]	0.00153198	No
rxn03135	Histidine metabolism	R04558	(1) L-Glutamate_c0[c1] + (2) H+_c0[c1] + (1) D-erythro-imidazol-glycerol-phosphate_c0[c1] + (1) AICAR_c0[c1] <- (1) L-Glutamine_c0[c1] + (1) phosphoribulosylformimino-AICAR-phosphate_c0[c1]	-0.00153198	No
rxn00863	Histidine metabolism	L-histidinal:NAD+ oxidoreductase	(1) H2O_c0[c1] + (1) NAD_c0[c1] + (1) L-Histidinal_c0[c1] -> (1) NADH_c0[c1] + (2) H+_c0[c1] + (1) L-Histidine_c0[c1]	0.00153198	No
rxn02834	Histidine metabolism	Phosphoribosyl-ATP pyrophosphohydrolase	(1) H2O_c0[c1] + (1) Phosphoribosyl-ATP_c0[c1] -> (1) PPi_c0[c1] + (2) H+_c0[c1] + (1) Phosphoribosyl-AMP_c0[c1]	0.00153198	No
rxn02473	Histidine metabolism	D-erythro-1-(Imidazol-4-yl)glycerol 3-phosphate hydro-lyase	(1) D-erythro-imidazol-glycerol-phosphate_c0[c1] -> (1) H2O_c0[c1] + (1) imidazole acetol-phosphate_c0[c1]	0.00153198	No
rxn02331	Lipopolysaccharide biosynthesis	phosphoenolpyruvate:D-arabinose-5-phosphate C-(1-carboxyvinyl)transferase (phosphate-hydrolysing, 2-carboxy-2-oxoethyl-forming)	(1) Phosphate_c0[c1] + (1) 3-Deoxy-D-manno-octulosonate8-phosphate_c0[c1] <- (1) H2O_c0[c1] + (1) Phosphoenolpyruvate_c0[c1] + (1) D-Arabinose5-phosphate_c0[c1]	-0.00145019	No
rxn02405	Lipopolysaccharide biosynthesis	CTP:3-deoxy-D-manno-octulosonate cytidylyltransferase	(1) CTP_c0[c1] + (1) KDO_c0[c1] -> (1) PPi_c0[c1] + (1) H+_c0[c1] + (1) CMP-KDO_c0[c1]	0.00145019	No
rxn06865	Lipopolysaccharide biosynthesis	R05146	(1) Lauroyl-KDO2-lipid IV(A)_c0[c1] + (1) ACP_c0[c1] <- (1) kdo2-lipid iva_c0[c1] + (1) Dodecanoyl-ACP_c0[c1]	-0.000483396	No
rxn00313	Lysine biosynthesis	meso-2,6-diaminoheptanedioate	(1) H+_c0[c1] + (1) meso-2,6-Diaminopimelate_c0[c1] <-> (1) CO2_c0[c1] + (1) L-Lysine_c0[c1]	-0.000483396	No

		carboxy-lyase (L-lysine-forming)			
rxn02011	Lysine biosynthesis|Peptidoglycan biosynthesis	UDP-N-acetylmuramoyl-L-alanyl-D-glutamate:(L)-meso-2,6-diaminoheptanedioate gamma-ligase (ADP-forming)	(1) ATP_c0[c1] + (1) meso-2,6-Diaminopimelate_c0[c1] + (1) UDP-N-acetylmuramoyl-L-alanyl-D-glutamate_c0[c1] -> (1) ADP_c0[c1] + (1) Phosphate_c0[c1] + (1) H+_c0[c1] + (1) UDP-N-acetylmuramoyl-L-alanyl-D-gamma-glutamyl-meso-2-6-diaminopimelate_c0[c1]	0.000483396	Yes
rxn02988	Nicotinate and nicotinamide metabolism	glycerone phosphate:iminosuccinate alkyltransferase (cyclizing)	(2) H2O_c0[c1] + (1) Phosphate_c0[c1] + (1) Quinolinate_c0[c1] <- (1) Glycerone-phosphate_c0[c1] + (1) Iminoaspartate_c0[c1]	-0.000119695	No
rxn02402	Nicotinate and nicotinamide metabolism	Nicotinate-nucleotide:pyrophosphate phosphoribosyltransferase (carboxylating)	(1) CO2_c0[c1] + (1) PPi_c0[c1] + (1) Nicotinate ribonucleotide_c0[c1] <- (1) H+_c0[c1] + (1) PRPP_c0[c1] + (1) Quinolinate_c0[c1]	-0.000119695	No
rxn02155	Nicotinate and nicotinamide metabolism	ATP:nicotinamide-nucleotide adenylyltransferase	(1) ATP_c0[c1] + (1) Nicotinate ribonucleotide_c0[c1] <-> (1) PPi_c0[c1] + (1) Deamido-NAD_c0[c1]	0.000119695	No
rxn00077	Nicotinate and nicotinamide metabolism	ATP:NAD+ 2'-phosphotransferase	(1) ATP_c0[c1] + (1) NAD_c0[c1] <-> (1) NADP_c0[c1] + (1) ADP_c0[c1] + (1) H+_c0[c1]	5.98474E-05	No
rxn00338	Nicotinate and nicotinamide metabolism	L-aspartate:oxygen oxidoreductase	(1) O2_c0[c1] + (1) L-Aspartate_c0[c1] -> (1) H2O2_c0[c1] + (1) H+_c0[c1] + (1) Iminoaspartate_c0[c1]	0.000119695	No
rxn00138	Nicotinate and nicotinamide metabolism	deamido-NAD+:ammonia ligase (AMP-forming)	(1) ATP_c0[c1] + (1) NH3_c0[c1] + (1) Deamido-NAD_c0[c1] -> (1) NAD_c0[c1] + (1) PPi_c0[c1] + (1) AMP_c0[c1] + (2) H+_c0[c1]	0.000119695	No
rxn00102	Nitrogen metabolism	carbonate hydro-lyase (carbon-dioxide-forming)	(1) H+_c0[c1] + (1) H2CO3_c0[c1] <-> (1) H2O_c0[c1] + (1) CO2_c0[c1]	-0.198951	No
rxn00907	One carbon pool by folate|Carbon fixation pathways in prokaryotes	5,10-methylenetetrahydrofolate:NADP+ oxidoreductase	(1) NADP_c0[c1] + (1) 5-10-Methylenetetrahydrofolate_c0[c1] <-> (1) NADPH_c0[c1] + (1) 5-10-Methenyltetrahydrofolate_c0[c1]	0.00159183	Yes
rxn01211	One carbon pool by folate|Carbon fixation pathways in prokaryotes	5,10-Methenyltetrahydrofolate 5-hydrolase (decyclizing)	(1) H2O_c0[c1] + (1) 5-10-Methenyltetrahydrofolate_c0[c1] <-> (1) H+_c0[c1] + (1) 10-Formyltetrahydrofolate_c0[c1]	0.00159183	Yes
rxn00686	One carbon pool by folate|Folate biosynthesis	5,6,7,8-tetrahydrofolate:NADP+ oxidoreductase	(1) NADP_c0[c1] + (1) Tetrahydrofolate_c0[c1] <-> (1) NADPH_c0[c1] + (1) H+_c0[c1] + (1) Dihydrofolate_c0[c1]	-0.000179542	No
rxn00910	One carbon pool by folate|Methane metabolism	5-methyltetrahydrofolate:NADP+ oxidoreductase	(1) NADP_c0[c1] + (1) 5-Methyltetrahydrofolate_c0[c1] <-> (1) NADPH_c0[c1] + (1) H+_c0[c1] + (1) 5-10-Methylenetetrahydrofolate_c0[c1]	-0.00294888	Yes
rxn09177	Pantothenate and CoA biosynthesis	(R)-4'-Phosphopantothenate:L-cysteine ligase	(1) CTP_c0[c1] + (1) L-Cysteine_c0[c1] + (1) 4-phosphopantothenate_c0[c1] -> (1) PPi_c0[c1] + (1) CMP_c0[c1] + (2) H+_c0[c1] + (1) (R)-4'-Phosphopantothenoyl-L-cysteine_c0[c1]	0.000119695	No
rxn00898	Pantothenate and CoA biosynthesis	2,3-Dihydroxy-3-methylbutanoate hydro-lyase	(1) 2,3-Dihydroxy-isovalerate_c0[c1] -> (1) H2O_c0[c1] + (1) 3-Methyl-2-oxobutanoate_c0[c1]	0.00692753	No
rxn00100	Pantothenate and CoA biosynthesis	ATP:dephospho-CoA 3'-phosphotransferase	(1) ATP_c0[c1] + (1) Dephospho-CoA_c0[c1] -> (1) ADP_c0[c1] + (1) CoA_c0[c1] + (1) H+_c0[c1]	0.000119695	No

rxn02175	Pantothenate and CoA biosynthesis	ATP:pantetheine-4'-phosphate adenylyltransferase	(1) ATP_c0[c1] + (1) Phosphopantetheine_c0[c1] <-> (1) PPi_c0[c1] + (1) Dephospho-CoA_c0[c1]	0.000119695	Yes
rxn06023	Pantothenate and CoA biosynthesis	CoA:apo-[acyl-carrier-protein] pantetheinephosphotransferase	(1) CoA_c0[c1] + (1) apo-ACP_c0[c1] <-> (1) Adenosine 3-5-bisphosphate_c0[c1] + (1) ACP_c0[c1]	5.98474E-05	No
rxn12510	Pantothenate and CoA biosynthesis	ATP:pantothenate 4'-phosphotransferase	(1) ATP_c0[c1] + (1) PAN_c0[c1] <-> (1) ADP_c0[c1] + (1) H+_c0[c1] + (1) 4-phosphopantothenate_c0[c1]	0.000119695	No
rxn01790	Pantothenate and CoA biosynthesis	(R)-Pantoate:NADP+ 2-oxidoreductase	(1) NADP_c0[c1] + (1) Pantoate_c0[c1] <-> (1) NADPH_c0[c1] + (1) H+_c0[c1] + (1) 2-Dehydropantoate_c0[c1]	-0.000119695	No
rxn00912	Pantothenate and CoA biosynthesis	5,10-Methylenetetrahydrofolate: 3-methyl-2-oxobutanoate hydroxymethyltransferase	(1) H2O_c0[c1] + (1) 3-Methyl-2-oxobutanoate_c0[c1] + (1) 5-10-Methylenetetrahydrofolate_c0[c1] <-> (1) Tetrahydrofolate_c0[c1] + (1) 2-Dehydropantoate_c0[c1]	0.000119695	No
rxn02341	Pantothenate and CoA biosynthesis	N-[(R)-4'-Phosphopantothenoyl]-L-cysteine carboxy-lyase	(1) H+_c0[c1] + (1) (R)-4'-Phosphopantothenoyl-L-cysteine_c0[c1] -> (1) CO2_c0[c1] + (1) Phosphopantetheine_c0[c1]	0.000119695	No
rxn02186	Pantothenate and CoA biosynthesis	2,3-Dihydroxy-3-methylbutanoate:NADP+ oxidoreductase (isomerizing)	(1) NADPH_c0[c1] + (1) H+_c0[c1] + (1) ALCTT_c0[c1] <-> (1) NADP_c0[c1] + (1) 2,3-Dihydroxy-isovalerate_c0[c1]	0.00692753	No
rxn01477	Pentose phosphate pathway	6-Phospho-D-gluconate hydro-lyase(2-dehydro-3-deoxy-6-phospho-D-gluconate-forming)	(1) 6-Phospho-D-gluconate_c0[c1] -> (1) H2O_c0[c1] + (1) 2-Keto-3-deoxy-6-phosphogluconate_c0[c1]	0.136463	No
rxn01476	Pentose phosphate pathway	6-Phospho-D-glucono-1,5-lactone lactonohydrolase	(1) H2O_c0[c1] + (1) 6-phospho-D-glucono-1-5-lactone_c0[c1] -> (1) H+_c0[c1] + (1) 6-Phospho-D-gluconate_c0[c1]	0.136463	No
rxn01333	Pentose phosphate pathway	sedoheptulose-7-phosphate:D-glyceraldehyde-3-phosphate glyceronetransferase	(1) Glyceraldehyde3-phosphate_c0[c1] + (1) Sedoheptulose7-phosphate_c0[c1] <-> (1) D-fructose-6-phosphate_c0[c1] + (1) D-Erythrose4-phosphate_c0[c1]	-0.0524304	Yes
rxn00777	Pentose phosphate pathway|Carbon fixation in photosynthetic organisms	D-ribose-5-phosphate aldose-ketose-isomerase	(1) ribose-5-phosphate_c0[c1] <-> (1) D-Ribulose5-phosphate_c0[c1]	-0.102449	No
rxn01200	Pentose phosphate pathway|Carbon fixation in photosynthetic organisms	Sedoheptulose-7-phosphate:D-glyceraldehyde-3-phosphate glycolaldehyde transferase	(1) Glyceraldehyde3-phosphate_c0[c1] + (1) Sedoheptulose7-phosphate_c0[c1] <-> (1) ribose-5-phosphate_c0[c1] + (1) D-Xylulose5-phosphate_c0[c1]	0.0504968	Yes
rxn03884	Pentose phosphate pathway|Pentose and glucuronate interconversions	2-dehydro-3-deoxy-D-gluconate-6-phosphate D-glyceraldehyde-3-phosphate-lyase	(1) 2-Keto-3-deoxy-6-phosphogluconate_c0[c1] <-> (1) Pyruvate_c0[c1] + (1) Glyceraldehyde3-phosphate_c0[c1]	0.136463	Yes
rxn01116	Pentose phosphate pathway|Pentose and glucuronate interconversions|Carbon fixation in photosynthetic organisms	D-Ribulose-5-phosphate 3-epimerase	(1) D-Ribulose5-phosphate_c0[c1] <-> (1) D-Xylulose5-phosphate_c0[c1]	-0.104199	No
rxn00770	Pentose phosphate pathway|Purine metabolism	ATP:D-ribose-5-phosphate diphosphotransferase	(1) ATP_c0[c1] + (1) ribose-5-phosphate_c0[c1] <-> (1) AMP_c0[c1] + (1) H+_c0[c1] + (1) PRPP_c0[c1]	0.148344	No

rxn03408	Peptidoglycan biosynthesis	UDP-N-acetyl-D-glucosamine:undecaprenyl-diphospho-N-acetylmuramoyl-L-alanyl-gamma-D-glutamyl-meso-2,6-diaminopimeloyl-D-alanyl-D-alanine 4-beta-N-acetylglucosaminlytransferase	(1) UDP-N-acetylglucosamine_c0[c1] + (1) Undecaprenyl-diphospho-N-acetylmuramoyl-L-alanyl-D-glutamyl-meso-2-6-diaminopimeloyl-D-alanyl-D-alanine_c0[c1] <-> (1) UDP_c0[c1] + (1) Undecaprenyl-diphospho-N-acetylmuramoyl--N-acetylglucosamine-L-ala-D-glu-meso-2-6-diaminopimeloyl-D-ala-D-ala_c0[c1]	0.000483396	Yes
rxn03904	Peptidoglycan biosynthesis	UDP-N-acetylmuramoyl-L-alanyl-gamma-D-glutamyl-meso-2,6-diaminopimeloyl-D-alanyl-D-alanine:undecaprenyl-phosphate phospho-N-acetylmuramoyl-pentapeptide-transferase	(1) Undecaprenylphosphate_c0[c1] + (1) UDP-N-acetylmuramoyl-L-alanyl-D-glutamyl-6-carboxy-L-lysyl-D-alanyl- D-alanine_c0[c1] <-> (1) UMP_c0[c1] + (1) Undecaprenyl-diphospho-N-acetylmuramoyl-L-alanyl-D-glutamyl-meso-2-6-diaminopimeloyl-D-alanyl-D-alanine_c0[c1]	0.000483396	No
rxn03901	Peptidoglycan biosynthesis	undecaprenyl-diphosphate phosphohydrolase	(1) H2O_c0[c1] + (1) Bactoprenyl diphosphate_c0[c1] -> (1) Phosphate_c0[c1] + (2) H+_c0[c1] + (1) Undecaprenylphosphate_c0[c1]	0.000483396	No
rxn01315	Phenylalanine metabolism|Phenylalanine, tyrosine and tryptophan biosynthesis	L-Phenylalanine,tetrahydrobiopterin:oxygen oxidoreductase (4-hydroxylating)	(1) O2_c0[c1] + (1) L-Phenylalanine_c0[c1] + (1) Tetrahydrobiopterin_c0[c1] -> (1) H2O_c0[c1] + (1) L-Tyrosine_c0[c1] + (1) Dihydrobiopterin_c0[c1]	0.00233239	No
rxn01739	Phenylalanine, tyrosine and tryptophan biosynthesis	ATP:shikimate 3-phosphotransferase	(1) ATP_c0[c1] + (1) Shikimate_c0[c1] <-> (1) ADP_c0[c1] + (1) H+_c0[c1] + (1) 3-phosphoshikimate_c0[c1]	0.00127139	No
rxn02212	Phenylalanine, tyrosine and tryptophan biosynthesis	2-Dehydro-3-deoxy-D-arabino-heptonate 7-phosphate phosphate-lyase (cyclyzing)	(1) DAHP_c0[c1] -> (1) Phosphate_c0[c1] + (1) 5-Dehydroquinate_c0[c1]	0.00127139	No
rxn01740	Phenylalanine, tyrosine and tryptophan biosynthesis	Shikimate:NADP+ 3-oxidoreductase	(1) NADP_c0[c1] + (1) Shikimate_c0[c1] <-> (1) NADPH_c0[c1] + (1) H+_c0[c1] + (1) 3-Dehydroshikimate_c0[c1]	-0.00127139	No
rxn01255	Phenylalanine, tyrosine and tryptophan biosynthesis	5-O-(1-Carboxyvinyl)-3-phosphoshikimate phosphate-lyase (chorismate-forming)	(1) 5-O--1-Carboxyvinyl-3-phosphoshikimate_c0[c1] -> (1) Phosphate_c0[c1] + (1) Chorismate_c0[c1]	0.00127139	No
rxn02508	Phenylalanine, tyrosine and tryptophan biosynthesis	N-(5-Phospho-beta-D-ribosyl)anthranilate ketol-isomerase	(1) N-5-phosphoribosyl-anthranilate_c0[c1] <-> (1) 1-(2-carboxyphenylamino)-1-deoxyribulose 5-phosphate_c0[c1]	0.000912302	No
rxn00726	Phenylalanine, tyrosine and tryptophan biosynthesis	chorismate pyruvate-lyase (amino-accepting; anthranilate-forming)	(1) NH3_c0[c1] + (1) Chorismate_c0[c1] -> (1) H2O_c0[c1] + (1) Pyruvate_c0[c1] + (1) H+_c0[c1] + (1) Anthranilate_c0[c1]	0.000912302	No
rxn00791	Phenylalanine, tyrosine and tryptophan biosynthesis	N-(5-Phospho-D-ribosyl)anthranilate:pyrophosphate phosphoribosyl-transferase	(1) PPi_c0[c1] + (1) H+_c0[c1] + (1) N-5-phosphoribosyl-anthranilate_c0[c1] <- (1) Anthranilate_c0[c1] + (1) PRPP_c0[c1]	-0.000912302	No
rxn02476	Phenylalanine, tyrosine and tryptophan biosynthesis	Phosphoenolpyruvate:3-phosphoshikimate 5-O-(1-carboxyvinyl)-transferase	(1) Phosphoenolpyruvate_c0[c1] + (1) 3-phosphoshikimate_c0[c1] <-> (1) Phosphate_c0[c1] + (1) 5-O--1-Carboxyvinyl-3-phosphoshikimate_c0[c1]	0.00127139	No
rxn02213	Phenylalanine, tyrosine and tryptophan biosynthesis	3-Dehydroquinate hydro-lyase	(1) 5-Dehydroquinate_c0[c1] -> (1) H2O_c0[c1] + (1) 3-Dehydroshikimate_c0[c1]	0.00127139	No

rxn01332	Phenylalanine, tyrosine and tryptophan biosynthesis	Phosphoenolpyruvate:D-erythrose-4-phosphate C-(1-carboxyvinyl)transferase (phosphate hydrolysing, 2-carboxy-2-oxoethyl-forming)	(1) H2O_c0[c1] + (1) Phosphoenolpyruvate_c0[c1] + (1) D-Erythrose4-phosphate_c0[c1] -> (1) Phosphate_c0[c1] + (1) DAHP_c0[c1]	0.00127139	No
rxn02507	Phenylalanine, tyrosine and tryptophan biosynthesis	1-(2-Carboxyphenylamino)-1-deoxy-D-ribulose-5-phosphate carboxy-lyase(cyclizing)	(1) H+_c0[c1] + (1) 1-(2-carboxyphenylamino)-1-deoxyribulose 5-phosphate_c0[c1] -> (1) H2O_c0[c1] + (1) CO2_c0[c1] + (1) Indoleglycerol phosphate_c0[c1]	0.000912302	No
rxn00060	Porphyrin and chlorophyll metabolism	porphobilinogen:(4-[2-carboxyethyl]-3-[carboxymethyl]pyrrol-2-yl)methyltransferase (hydrolysing)	(1) H2O_c0[c1] + (4) Porphobilinogen_c0[c1] -> (4) NH3_c0[c1] + (1) Hydroxymethylbilane_c0[c1]	0.000119695	No
rxn00029	Porphyrin and chlorophyll metabolism	5-aminolevulinate hydro-lyase (adding 5-aminolevulinate and cyclizing; porphobilinogen-forming)	(2) 5-Aminolevulinate_c0[c1] -> (2) H2O_c0[c1] + (1) H+_c0[c1] + (1) Porphobilinogen_c0[c1]	0.000478779	No
rxn03537	Porphyrin and chlorophyll metabolism	R05222	(1) GTP_c0[c1] + (1) Adenosyl cobinamide phosphate_c0[c1] <-> (1) PPi_c0[c1] + (1) H+_c0[c1] + (1) Adenosylcobinamide-GDP_c0[c1]	5.98474E-05	No
rxn04413	Porphyrin and chlorophyll metabolism	R06558	(1) GTP_c0[c1] + (1) Adenosyl cobinamide_c0[c1] <-> (1) GDP_c0[c1] + (1) H+_c0[c1] + (1) Adenosyl cobinamide phosphate_c0[c1]	5.98474E-05	No
rxn03150	Porphyrin and chlorophyll metabolism	R04594	(1) H2O_c0[c1] + (1) alpha-Ribazole 5'-phosphate_c0[c1] -> (1) Phosphate_c0[c1] + (1) alpha-Ribazole_c0[c1]	5.98474E-05	No
rxn05029	Porphyrin and chlorophyll metabolism	ATP:cobinamide Cobeta-adenosyltransferase	(1) ATP_c0[c1] + (1) H+_c0[c1] + (1) Cobinamide_c0[c1] <-> (1) Triphosphate_c0[c1] + (1) Adenosyl cobinamide_c0[c1]	5.98474E-05	No
rxn02303	Porphyrin and chlorophyll metabolism	Coproporphyrinogen:oxygen oxidoreductase(decarboxylating)	(1) O2_c0[c1] + (2) H+_c0[c1] + (1) CoproporphyrinogenIII_c0[c1] <-> (2) H2O_c0[c1] + (2) CO2_c0[c1] + (1) ProtoporphyrinogenIX_c0[c1]	5.98474E-05	No
rxn03538	Porphyrin and chlorophyll metabolism	R05223	(1) alpha-Ribazole_c0[c1] + (1) Adenosylcobinamide-GDP_c0[c1] <-> (1) H+_c0[c1] + (1) GMP_c0[c1] + (1) Calomide_c0[c1]	5.98474E-05	No
rxn02897	Porphyrin and chlorophyll metabolism	Nicotinate-nucleotide:dimethylbenzimidazole phospho-D-ribosyltransferase	(1) Nicotinate ribonucleotide_c0[c1] + (1) Dimethylbenzimidazole_c0[c1] <-> (1) H+_c0[c1] + (1) Niacin_c0[c1] + (1) alpha-Ribazole 5'-phosphate_c0[c1]	5.98474E-05	No
rxn02056	Porphyrin and chlorophyll metabolism	S-Adenosyl-L-methionine:uroporphyrin-III C-methyltransferase	(2) H+_c0[c1] + (1) Siroheme_c0[c1] <-> (1) Sirohydrochlorin_c0[c1] + (1) Fe2+_c0[c1]	-5.98474E-05	No
rxn02288	Porphyrin and chlorophyll metabolism	Uroporphyrinogen-III carboxy-lyase	(4) H+_c0[c1] + (1) UroporphyrinogenIII_c0[c1] <-> (4) CO2_c0[c1] + (1) CoproporphyrinogenIII_c0[c1]	5.98474E-05	Yes
rxn00224	Porphyrin and chlorophyll metabolism	protoheme ferro-lyase (protoporphyrin-forming)	(1) Protoporphyrin_c0[c1] + (1) Fe2+_c0[c1] <-> (1) Heme_c0[c1] + (2) H+_c0[c1]	5.98474E-05	No
rxn01297	Purine metabolism	hypoxanthine:NAD+ oxidoreductase	(1) H2O_c0[c1] + (1) NAD_c0[c1] + (1) HYXN_c0[c1] <-> (1)	0.00138435	No

			NADH_c0[c1] + (1) H+_c0[c1] + (1) XAN_c0[c1]		
rxn00097	Purine metabolism	ATP:AMP phosphotransferase	(1) ATP_c0[c1] + (1) AMP_c0[c1] + (1) H+_c0[c1] <-> (2) ADP_c0[c1]	0.117428	No
rxn00131	Purine metabolism	AMP phosphoribohydrolase	(1) H2O_c0[c1] + (1) AMP_c0[c1] <-> (1) ribose-5-phosphate_c0[c1] + (1) Adenine_c0[c1]	-0.00460213	No
rxn00917	Purine metabolism	Xanthosine-5'-phosphate:L-glutamine amido-ligase (AMP-forming)	(1) H2O_c0[c1] + (1) ATP_c0[c1] + (1) L-Glutamine_c0[c1] + (1) XMP_c0[c1] -> (1) PPi_c0[c1] + (1) AMP_c0[c1] + (1) L-Glutamate_c0[c1] + (3) H+_c0[c1] + (1) GMP_c0[c1]	0.00291633	Yes
rxn00927	Purine metabolism	Adenosine ribohydrolase	(1) H2O_c0[c1] + (1) Adenosine_c0[c1] <-> (1) D-Ribose_c0[c1] + (1) Adenine_c0[c1]	0.00460213	No
rxn01352	Purine metabolism	dGTP triphosphohydrolase	(1) H2O_c0[c1] + (1) dGTP_c0[c1] <-> (1) Deoxyguanosine_c0[c1] + (1) Triphosphate_c0[c1]	-0.000396649	No
rxn01544	Purine metabolism	XMP:pyrophosphate phosphoribosyltransferase	(1) PPi_c0[c1] + (1) H+_c0[c1] + (1) XMP_c0[c1] <- (1) PRPP_c0[c1] + (1) XAN_c0[c1]	-0.00138435	No
rxn00832	Purine metabolism	IMP 1,2-hydrolase (decyclizing)	(1) H2O_c0[c1] + (1) IMP_c0[c1] <-> (1) FAICAR_c0[c1]	-0.00153198	No
rxn00515	Purine metabolism	ATP:IDP phosphotransferase	(1) ATP_c0[c1] + (1) IDP_c0[c1] <-> (1) ADP_c0[c1] + (1) ITP_c0[c1]	-0.140558	Yes
rxn05231	Purine metabolism	2'-Deoxyadenosine 5'-diphosphate:oxidized-thioredoxin 2'-oxidoreductase	(1) ADP_c0[c1] + (1) trdrd_c0[c1] -> (1) H2O_c0[c1] + (1) dADP_c0[c1] + (1) trdox_c0[c1]	0.000222544	No
rxn01299	Purine metabolism	Inosine ribohydrolase	(1) H2O_c0[c1] + (1) Inosine_c0[c1] <-> (1) D-Ribose_c0[c1] + (1) HYXN_c0[c1]	-0.00460213	No
rxn00834	Purine metabolism	IMP:NAD+ oxidoreductase	(1) H2O_c0[c1] + (1) NAD_c0[c1] + (1) IMP_c0[c1] <-> (1) NADH_c0[c1] + (1) H+_c0[c1] + (1) XMP_c0[c1]	0.00153198	Yes
rxn00239	Purine metabolism	ATP:GMP phosphotransferase	(1) ATP_c0[c1] + (1) H+_c0[c1] + (1) GMP_c0[c1] <-> (1) ADP_c0[c1] + (1) GDP_c0[c1]	0.00297618	No
rxn00839	Purine metabolism	ATP:dADP phosphotransferase	(1) ATP_c0[c1] + (1) dADP_c0[c1] <-> (1) ADP_c0[c1] + (1) dATP_c0[c1]	0.000222544	Yes
rxn03137	Purine metabolism|One carbon pool by folate	10-Formyltetrahydrofolate:5'-phosphoribosyl-5-amino-4-imidazolecarboxamide formyltransferase	(1) 10-Formyltetrahydrofolate_c0[c1] + (1) AICAR_c0[c1] <-> (1) Tetrahydrofolate_c0[c1] + (1) FAICAR_c0[c1]	0.00153198	No
rxn00361	Purine metabolism|Sulfur metabolism	ATP:adenylylsulfate 3'-phosphotransferase	(1) ATP_c0[c1] + (1) APS_c0[c1] <-> (1) ADP_c0[c1] + (1) 3-phosphoadenylylsulfate_c0[c1] + (1) H+_c0[c1]	-5.98474E-05	No
rxn00379	Purine metabolism|Sulfur metabolism	ATP:sulfate adenylyltransferase	(1) ATP_c0[c1] + (1) Sulfate_c0[c1] <-> (1) PPi_c0[c1] + (1) APS_c0[c1]	0.00721725	No
rxn00364	Pyrimidine metabolism	ATP:CMP phosphotransferase	(1) ATP_c0[c1] + (1) CMP_c0[c1] + (1) H+_c0[c1] <-> (1) ADP_c0[c1] + (1) CDP_c0[c1]	-0.129765	No
rxn01673	Pyrimidine metabolism	ATP:dCDP phosphotransferase	(1) ATP_c0[c1] + (1) dCDP_c0[c1] <-> (1) ADP_c0[c1] + (1) dCTP_c0[c1]	0.000396649	Yes
rxn00710	Pyrimidine metabolism	orotidine-5'-phosphate carboxy-lyase (UMP-forming)	(1) H+_c0[c1] + (1) Orotidylic acid_c0[c1] -> (1) CO2_c0[c1] + (1) UMP_c0[c1]	0.144336	No

rxn01362	Pyrimidine metabolism	Orotidine-5'-phosphate:diphosphate phospho-alpha-D-ribosyl-transferase	(1) PPi_c0[c1] + (1) H+_c0[c1] + (1) Orotidylic acid_c0[c1] <- (1) PRPP_c0[c1] + (1) Orotate_c0[c1]	-0.144336	No
rxn01465	Pyrimidine metabolism	(S)-dihydroorotate amidohydrolase	(1) H2O_c0[c1] + (1) S-Dihydroorotate_c0[c1] <-> (1) H+_c0[c1] + (1) N-Carbamoyl-L-aspartate_c0[c1]	-0.144336	Yes
rxn00707	Pyrimidine metabolism	ITP:cytidine 5'-phosphotransferase	(1) ITP_c0[c1] + (1) Cytidine_c0[c1] <-> (1) CMP_c0[c1] + (1) H+_c0[c1] + (1) IDP_c0[c1]	-0.140558	No
rxn00410	Pyrimidine metabolism	UTP:ammonia ligase (ADP-forming)	(1) ATP_c0[c1] + (1) NH3_c0[c1] + (1) UTP_c0[c1] <-> (1) ADP_c0[c1] + (1) Phosphate_c0[c1] + (1) CTP_c0[c1] + (2) H+_c0[c1]	0.14258	Yes
rxn01143	Pyrimidine metabolism	ATP:thymidine 5'-phosphotransferase	(1) ATP_c0[c1] + (1) Thymidine_c0[c1] <-> (1) ADP_c0[c1] + (1) H+_c0[c1] + (1) dTMP_c0[c1]	0.000222544	No
rxn01512	Pyrimidine metabolism	ATP:dTDP phosphotransferase	(1) ATP_c0[c1] + (1) dTDP_c0[c1] <-> (1) ADP_c0[c1] + (1) TTP_c0[c1]	0.00070594	Yes
rxn06076	Pyrimidine metabolism	2'-Deoxycytidine diphosphate:oxidized-thioredoxin 2'-oxidoreductase	(1) H2O_c0[c1] + (1) dCDP_c0[c1] + (1) trdox_c0[c1] <- (1) CDP_c0[c1] + (1) trdrd_c0[c1]	-0.000396649	No
rxn00409	Pyrimidine metabolism	ATP:CDP phosphotransferase	(1) ATP_c0[c1] + (1) CDP_c0[c1] <-> (1) ADP_c0[c1] + (1) CTP_c0[c1]	-0.130161	Yes
rxn01513	Pyrimidine metabolism	ATP:dTMP phosphotransferase	(1) ATP_c0[c1] + (1) H+_c0[c1] + (1) dTMP_c0[c1] <-> (1) ADP_c0[c1] + (1) dTDP_c0[c1]	0.000222544	No
rxn05289	Pyrimidine metabolism	NADPH:oxidized-thioredoxin oxidoreductase	(1) NADPH_c0[c1] + (1) H+_c0[c1] + (1) trdox_c0[c1] <-> (1) NADP_c0[c1] + (1) trdrd_c0[c1]	0.00480042	No
rxn00117	Pyrimidine metabolism	ATP:UDP phosphotransferase	(1) ATP_c0[c1] + (1) UDP_c0[c1] <-> (1) ADP_c0[c1] + (1) UTP_c0[c1]	0.000966792	Yes
rxn01018	Pyrimidine metabolism|Alanine, aspartate and glutamate metabolism	carbamoyl-phosphate:L-aspartate carbamoyltransferase	(1) L-Aspartate_c0[c1] + (1) Carbamoylphosphate_c0[c1] -> (1) Phosphate_c0[c1] + (1) H+_c0[c1] + (1) N-Carbamoyl-L-aspartate_c0[c1]	0.144336	Yes
rxn00414	Pyrimidine metabolism|Alanine, aspartate and glutamate metabolism	hydrogen-carbonate:L-glutamine amido-ligase (ADP-forming, carbamate-phosphorylating)	(1) H2O_c0[c1] + (2) ATP_c0[c1] + (1) L-Glutamine_c0[c1] + (1) H2CO3_c0[c1] -> (2) ADP_c0[c1] + (1) Phosphate_c0[c1] + (1) L-Glutamate_c0[c1] + (2) H+_c0[c1] + (1) Carbamoylphosphate_c0[c1]	0.149104	Yes
rxn00146	Pyruvate metabolism	(R)-Lactate:ferricytochrome-c 2-oxidoreductase	(2) Cytochrome c3+_c0[c1] + (1) D-Lactate_c0[c1] <-> (1) Pyruvate_c0[c1] + (2) H+_c0[c1] + (2) Cytochrome c2+_c0[c1]	-0.0552484	No
rxn00500	Pyruvate metabolism	(R)-Lactate:NAD+ oxidoreductase	(1) NAD_c0[c1] + (1) D-Lactate_c0[c1] <-> (1) NADH_c0[c1] + (1) Pyruvate_c0[c1] + (1) H+_c0[c1]	0.0552484	No
rxn00151	Pyruvate metabolism|Carbon fixation in photosynthetic organisms	ATP:pyruvate,phosphate phosphotransferase	(1) ATP_c0[c1] + (1) Phosphate_c0[c1] + (1) Pyruvate_c0[c1] <-> (1) PPi_c0[c1] + (1) AMP_c0[c1] + (1) Phosphoenolpyruvate_c0[c1] + (2) H+_c0[c1]	-0.112371	No
rxn00251	Pyruvate metabolism|Methane metabolism|Carbon fixation in photosynthetic organisms|Carbon fixation pathways in prokaryotes	phosphate:oxaloacetate carboxy-lyase (adding phosphate;phosphoenolpyruvate-forming)	(1) Phosphate_c0[c1] + (1) Oxaloacetate_c0[c1] + (1) H+_c0[c1] <-> (1) H2O_c0[c1] + (1) CO2_c0[c1] + (1) Phosphoenolpyruvate_c0[c1]	-0.166214	Yes

rxn05040	Riboflavin metabolism	D-ribulose 5-phosphate formate-lyase (L-3,4-dihydroxybutan-2-one 4-phosphate-forming)	(1) D-Ribulose5-phosphate_c0[c1] -> (1) Formate_c0[c1] + (1) H+_c0[c1] + (1) 3-4-dihydroxy-2-butanone4-phosphate_c0[c1]	0.00023939	No
rxn00300	Riboflavin metabolism	GTP 7,8-8,9-dihydrolase (diphosphate-forming)	(3) H2O_c0[c1] + (1) GTP_c0[c1] -> (1) PPi_c0[c1] + (1) Formate_c0[c1] + (3) H+_c0[c1] + (1) 2,5-Diamino-6-(5'-phosphoribosylamino)-4-pyrimidineone_c0[c1]	0.000119695	No
rxn00392	Riboflavin metabolism	ATP:riboflavin 5'-phosphotransferase	(1) ATP_c0[c1] + (1) Riboflavin_c0[c1] <-> (1) ADP_c0[c1] + (1) FMN_c0[c1] + (1) H+_c0[c1]	5.98474E-05	No
rxn03080	Riboflavin metabolism	5-amino-6-(D-ribitylamino)uracil butanedionetransferase	(1) 4--1-D-Ribitylamino-5-aminouracil_c0[c1] + (1) 3-4-dihydroxy-2-butanone4-phosphate_c0[c1] <-> (2) H2O_c0[c1] + (1) Phosphate_c0[c1] + (1) H+_c0[c1] + (1) 6-7-Dimethyl-8--1-D-ribityllumazine_c0[c1]	0.00023939	No
rxn00048	Riboflavin metabolism	6,7-Dimethyl-8-(1-D-ribityl)lumazine:6,7-dimethyl-8-(1-D-ribityl)lumazine 2,3-butanediyltransferase	(1) H+_c0[c1] + (2) 6-7-Dimethyl-8--1-D-ribityllumazine_c0[c1] -> (1) Riboflavin_c0[c1] + (1) 4--1-D-Ribitylamino-5-aminouracil_c0[c1]	0.000119695	No
rxn02475	Riboflavin metabolism	2,5-Diamino-6-hydroxy-4-(5-phosphoribosylamino)-pyrimidine 2-aminohydrolase	(1) H2O_c0[c1] + (1) H+_c0[c1] + (1) 2,5-Diamino-6-(5'-phosphoribosylamino)-4-pyrimidineone_c0[c1] -> (1) NH3_c0[c1] + (1) 5-Amino-6--5-phosphoribosylaminouracil_c0[c1]	0.000119695	No
rxn00122	Riboflavin metabolism	ATP:FMN adenylyltransferase	(1) ATP_c0[c1] + (1) FMN_c0[c1] -> (1) PPi_c0[c1] + (1) FAD_c0[c1]	5.98474E-05	No
rxn02474	Riboflavin metabolism	5-amino-6-(5-phosphoribitylamino)uracil:NADP+ 1'-oxidoreductase	(1) NADP_c0[c1] + (1) 5-Amino-6--5-phosphoribitylaminouracil_c0[c1] <-> (1) NADPH_c0[c1] + (1) H+_c0[c1] + (1) 5-Amino-6--5-phosphoribosylaminouracil_c0[c1]	-0.000119695	No
rxn01997	Streptomycin biosynthesis|Polyketide sugar unit biosynthesis|Biosynthesis of vancomycin group antibiotics	dTDPglucose 4,6-hydro-lyase	(1) dTDPglucose_c0[c1] -> (1) H2O_c0[c1] + (1) dTDP-4-oxo-6-deoxy-D-glucose_c0[c1]	0.000483396	No
rxn05239	Sulfur metabolism	adenosine 3',5'-bisphosphate,sulfite:oxidized-thioredoxin oxidoreductase (3'-phosphoadenosine-5'-phosphosulfate -forming)	(1) 3-phosphoadenylylsulfate_c0[c1] + (1) trdrd_c0[c1] <-> (1) Adenosine 3-5-bisphosphate_c0[c1] + (1) H+_c0[c1] + (1) Sulfite_c0[c1] + (1) trdox_c0[c1]	-5.98474E-05	No
rxn00623	Sulfur metabolism	hydrogen-sulfide:NADP+ oxidoreductase	(3) H2O_c0[c1] + (3) NADP_c0[c1] + (1) H2S_c0[c1] <-> (3) NADPH_c0[c1] + (3) H+_c0[c1] + (1) Sulfite_c0[c1]	-0.00418122	No
rxn04996	Terpenoid backbone biosynthesis	dimethyallyl diphosphate:NADP+ oxidoreductase	(1) H2O_c0[c1] + (1) NADP_c0[c1] + (1) DMAPP_c0[c1] <- (1) NADPH_c0[c1] + (1) H+_c0[c1] + (1) 1-Hydroxy-2-methyl-2-butenyl 4-diphosphate_c0[c1]	-0.000662938	No
rxn03958	Terpenoid backbone biosynthesis	1-Deoxy-D-xylulose-5-phosphate isomeroreductase	(1) NADP_c0[c1] + (1) 2-C-methyl-D-erythritol4-phosphate_c0[c1] <-> (1) NADPH_c0[c1] + (1) H+_c0[c1] + (1) 1-deoxy-D-xylulose5-phosphate_c0[c1]	-0.00675369	No
rxn03910	Terpenoid backbone biosynthesis	2-Phospho-4-(cytidine 5'-diphospho)-2-C-methyl-D-	(1) 2-phospho-4--cytidine5-diphospho-2-C-methyl-D-erythritol_c0[c1] <-> (1)	0.00675369	No

		erythritol CMP-lyase (cyclizing)	CMP_c0[c1] + (1) 2-C-methyl-D-erythritol2-4-cyclodiphosphate_c0[c1]		
rxn03908	Terpenoid backbone biosynthesis	ATP:4-(Cytidine 5'-diphospho)-2-C-methyl-D-erythritol 2-phosphotransferase	(1) ATP_c0[c1] + (1) 4--cytidine5-diphospho-2-C-methyl-D-erythritol_c0[c1] <-> (1) ADP_c0[c1] + (1) H+_c0[c1] + (1) 2-phospho-4--cytidine5-diphospho-2-C-methyl-D-erythritol_c0[c1]	0.00675369	No
rxn03907	Terpenoid backbone biosynthesis	CTP: 2-C-Methyl-D-erythritol 4-phosphate cytidylyltransferase	(1) CTP_c0[c1] + (1) 2-C-methyl-D-erythritol4-phosphate_c0[c1] <-> (1) PPi_c0[c1] + (1) 4--cytidine5-diphospho-2-C-methyl-D-erythritol_c0[c1]	0.00675369	No
rxn01213	Terpenoid backbone biosynthesis	GPPSYN-RXN	(1) Isopentenyldiphosphate_c0[c1] + (1) DMAPP_c0[c1] -> (1) PPi_c0[c1] + (1) H+_c0[c1] + (1) Geranyldiphosphate_c0[c1]	0.000662938	No
rxn04113	Terpenoid backbone biosynthesis	isopentenyl-diphosphate:NAD(P)+ oxidoreductase	(1) NADPH_c0[c1] + (1) H+_c0[c1] + (1) 1-Hydroxy-2-methyl-2-butenyl 4-diphosphate_c0[c1] -> (1) H2O_c0[c1] + (1) NADP_c0[c1] + (1) Isopentenyldiphosphate_c0[c1]	0.00609076	No
rxn03909	Terpenoid backbone biosynthesis	1-Deoxy-D-xylulose-5-phosphate pyruvate-lyase (carboxylating)	(1) Pyruvate_c0[c1] + (1) H+_c0[c1] + (1) Glyceraldehyde3-phosphate_c0[c1] -> (1) CO2_c0[c1] + (1) 1-deoxy-D-xylulose5-phosphate_c0[c1]	0.00675369	No
rxn01466	Terpenoid backbone biosynthesis	Geranyl-diphosphate:isopentenyl-diphosphate geranyltrans-transferase	(1) Isopentenyldiphosphate_c0[c1] + (1) Geranyldiphosphate_c0[c1] -> (1) PPi_c0[c1] + (1) H+_c0[c1] + (1) Farnesyldiphosphate_c0[c1]	0.000662938	No
rxn00533	Tetracycline biosynthesis|Pyruvate metabolism|Propanoate metabolism|Carbon fixation pathways in prokaryotes	Acetyl-CoA:carbon-dioxide ligase (ADP-forming)	(1) ATP_c0[c1] + (1) Acetyl-CoA_c0[c1] + (1) H2CO3_c0[c1] <-> (1) ADP_c0[c1] + (1) Phosphate_c0[c1] + (1) H+_c0[c1] + (1) Malonyl-CoA_c0[c1]	0.0498471	No
rxn02484	Thiamine metabolism	ATP:4-amino-5-hydroxymethyl-2-methylpyrimidine 5-phosphotransferase	(1) ATP_c0[c1] + (1) Toxopyrimidine_c0[c1] <-> (1) ADP_c0[c1] + (1) H+_c0[c1] + (1) 4-Amino-5-phosphomethyl-2-methylpyrimidine_c0[c1]	-0.000179542	No
rxn00438	Thiamine metabolism	ATP:thiamin-phosphate phosphotransferase	(1) ATP_c0[c1] + (1) H+_c0[c1] + (1) Thiamine phosphate_c0[c1] <-> (1) ADP_c0[c1] + (1) TPP_c0[c1]	5.98474E-05	No
rxn11946	Ubiquinone and other terpenoid-quinone biosynthesis	R05614	(1) S-Adenosyl-L-methionine_c0[c1] + (1) 2-Octaprenyl-3-methyl-5-hydroxy-6-methoxy-1,4-benzoquinone_c0[c1] <-> (1) S-Adenosyl-homocysteine_c0[c1] + (1) H+_c0[c1] + (1) Ubiquinone-8_c0[c1]	5.98474E-05	No
rxn04139	Ubiquinone and other terpenoid-quinone biosynthesis	2-Octaprenyl-3-methyl-6-methoxy-1,4-benzoquinone ,NADPH2:oxygen oxidoreductase	(1) NADPH_c0[c1] + (1) O2_c0[c1] + (1) H+_c0[c1] + (1) 2-Octaprenyl-3-methyl-6-methoxy-1,4-benzoquinone_c0[c1] -> (1) H2O_c0[c1] + (1) NADP_c0[c1] + (1) 2-Octaprenyl-3-methyl-5-hydroxy-6-methoxy-1,4-benzoquinone_c0[c1]	5.98474E-05	No
rxn03893	Ubiquinone and other terpenoid-quinone biosynthesis	all-trans-octaprenyl-diphosphate:4-hydroxybenzoate 3-octaprenyltransferase	(1) 4-Hydroxybenzoate_c0[c1] + (1) Farnesylfarnesylgeraniol_c0[c1] -> (1) PPi_c0[c1] + (1) H+_c0[c1] + (1) 3-Octaprenyl-4-hydroxybenzoate_c0[c1]	5.98474E-05	No

rxn03394	Ubiquinone and other terpenoid-quinone biosynthesis	R04987	(1) NADPH_c0[c1] + (1) O2_c0[c1] + (1) H+_c0[c1] + (1) 2-Octaprenylphenol_c0[c1] -> (1) H2O_c0[c1] + (1) NADP_c0[c1] + (1) 2-Octaprenyl-6-hydroxyphenol_c0[c1]	5.98474E-05	No
rxn03395	Ubiquinone and other terpenoid-quinone biosynthesis	S-adenosyl-L-methionine:3-(all-trans-octaprenyl)benzene-1,2-diol 2-O-methyltransferase	(1) S-Adenosyl-L-methionine_c0[c1] + (1) 2-Octaprenyl-6-hydroxyphenol_c0[c1] <-> (1) S-Adenosyl-homocysteine_c0[c1] + (1) H+_c0[c1] + (1) 2-Octaprenyl-6-methoxyphenol_c0[c1]	5.98474E-05	No
rxn03436	Valine, leucine and isoleucine biosynthesis	(S)-2-Aceto-2-hydroxybutanoate:NADP+ oxidoreductase (isomerizing)	(1) 2-Aceto-2-hydroxybutanoate_c0[c1] <-> (1) (R)-3-Hydroxy-3-methyl-2-oxopentanoate_c0[c1]	0.00631981	No
rxn03435	Valine, leucine and isoleucine biosynthesis	(R)-2,3-Dihydroxy-3-methylpentanoate:NADP+ oxidoreductase (isomerizing)	(1) NADP_c0[c1] + (1) 2,3-Dihydroxy-3-methylvalerate_c0[c1] <-> (1) NADPH_c0[c1] + (1) H+_c0[c1] + (1) (R)-3-Hydroxy-3-methyl-2-oxopentanoate_c0[c1]	-0.00631981	No
rxn01045	Valine, leucine and isoleucine biosynthesis	L-Valine:NAD+ oxidoreductase(deaminating)	(1) H2O_c0[c1] + (1) NAD_c0[c1] + (1) L-Valine_c0[c1] <-> (1) NADH_c0[c1] + (1) NH3_c0[c1] + (1) H+_c0[c1] + (1) 3-Methyl-2-oxobutanoate_c0[c1]	-0.00680784	No
rxn01573	Valine, leucine and isoleucine biosynthesis	L-Isoleucine:NAD+ oxidoreductase(deaminating)	(1) H2O_c0[c1] + (1) NAD_c0[c1] + (1) L-Isoleucine_c0[c1] <-> (1) NADH_c0[c1] + (1) NH3_c0[c1] + (1) H+_c0[c1] + (1) 3MOP_c0[c1]	-0.0046734	No
rxn03437	Valine, leucine and isoleucine biosynthesis	(R)-2,3-Dihydroxy-3-methylpentanoate hydro-lyase	(1) 2,3-Dihydroxy-3-methylvalerate_c0[c1] -> (1) H2O_c0[c1] + (1) 3MOP_c0[c1]	0.00631981	No
rxn03194	Valine, leucine and isoleucine biosynthesis	(S)-2-Aceto-2-hydroxybutanoate pyruvate-lyase (carboxylating)	(1) 2-Oxobutyrate_c0[c1] + (1) 2-Hydroxyethyl-ThPP_c0[c1] <-> (1) TPP_c0[c1] + (1) 2-Aceto-2-hydroxybutanoate_c0[c1]	0.00631981	No
rxn07434	Valine, leucine and isoleucine degradation	R07603	(1) TPP_c0[c1] + (1) H+_c0[c1] + (1) 3MOP_c0[c1] -> (1) CO2_c0[c1] + (1) 2-Methyl-1-hydroxybutyl-TPP_c0[c1]	0.00164641	No
rxn07433	Valine, leucine and isoleucine degradation	R07602	(1) Lipoamide_c0[c1] + (1) 3-Methyl-1-hydroxybutyl-TPP_c0[c1] <-> (1) TPP_c0[c1] + (1) S-(3-Methylbutanoyl)-dihydrolipoamide-E_c0[c1]	0.00164641	No
rxn07435	Valine, leucine and isoleucine degradation	R07604	(1) Lipoamide_c0[c1] + (1) 2-Methyl-1-hydroxybutyl-TPP_c0[c1] <-> (1) TPP_c0[c1] + (1) S-(2-Methylbutanoyl)-dihydrolipoamide-E_c0[c1]	0.00164641	No
rxn06586	Valine, leucine and isoleucine degradation	3-methylbutanoyl-CoA:enzyme N6-(dihydrolipoyl)lysine S-(3-methylbutanoyl)transferase	(1) Dihydrolipoamide_c0[c1] + (1) Isovaleryl-CoA_c0[c1] <-> (1) CoA_c0[c1] + (1) S-(3-Methylbutanoyl)-dihydrolipoamide-E_c0[c1]	-0.00164641	No
rxn07432	Valine, leucine and isoleucine degradation	R07601	(1) TPP_c0[c1] + (1) H+_c0[c1] + (1) 4MOP_c0[c1] -> (1) CO2_c0[c1] + (1) 3-Methyl-1-hydroxybutyl-TPP_c0[c1]	0.00164641	No
rxn06335	Valine, leucine and isoleucine degradation	(S)-2-methylbutanoyl-CoA:enzyme N6-(dihydrolipoyl)lysine S-(2-methylbutanoyl)transferase	(1) Dihydrolipoamide_c0[c1] + (1) 2-Methylbutyryl-CoA_c0[c1] <-> (1) CoA_c0[c1] + (1) S-(2-Methylbutanoyl)-dihydrolipoamide-E_c0[c1]	-0.00164641	No

rxn00806	Valine, leucine and isoleucine degradation|Valine, leucine and isoleucine biosynthesis|Glucosinolate biosynthesis	L-Leucine:2-oxoglutarate aminotransferase	(1) 2-Oxoglutarate_c0[c1] + (1) L-Leucine_c0[c1] <-> (1) L-Glutamate_c0[c1] + (1) 4MOP_c0[c1]	0.00164641	No
